# Structural deviations of the posterior fossa and the cerebellum and their cognitive links in a neurodevelopmental deletion syndrome

**DOI:** 10.1038/s41380-024-02584-8

**Published:** 2024-05-14

**Authors:** Esra Sefik, Kuaikuai Duan, Yiheng Li, Brittney Sholar, Lindsey Evans, Jordan Pincus, Zeena Ammar, Melissa M. Murphy, Cheryl Klaiman, Celine A. Saulnier, Stormi L. Pulver, Adam E. Goldman-Yassen, Ying Guo, Elaine F. Walker, Longchuan Li, Jennifer G. Mulle, Sarah Shultz

**Affiliations:** 1 Department of Human Genetics, Emory University School of Medicine, Atlanta, GA USA; 2 Department of Psychology, Emory University, Atlanta, GA USA; 3 Department of Pediatrics, Emory University School of Medicine, Atlanta, GA USA; 4 Tri-institutional Center for Translational Research in Neuroimaging and Data Science, Georgia State University, Georgia Institute of Technology, Emory University, Atlanta, GA USA; 5 Department of Biostatistics and Bioinformatics, Rollins School of Public Health, Emory University, Atlanta, GA USA; 6 Marcus Autism Center, Children’s Healthcare of Atlanta and Emory University School of Medicine, Atlanta, GA USA; 7 Neurodevelopmental Assessment & Consulting Services, Atlanta, GA USA; 8 Department of Radiology, Children’s Healthcare of Atlanta, Atlanta, GA USA; 9 Department of Radiology and Imaging Sciences, Emory University School of Medicine, Atlanta, GA USA; 10 Department of Psychiatry, Robert Wood Johnson Medical School, Rutgers University, New Brunswick, NJ USA

**Keywords:** Neuroscience, Genetics, Psychology

## Abstract

High-impact genetic variants associated with neurodevelopmental disorders provide biologically-defined entry points for mechanistic investigation. The 3q29 deletion (3q29Del) is one such variant, conferring a 40-100-fold increased risk for schizophrenia, as well as high risk for autism and intellectual disability. However, the mechanisms leading to neurodevelopmental disability remain largely unknown. Here, we report the first in vivo quantitative neuroimaging study in individuals with 3q29Del (*N* = 24) and neurotypical controls (*N* = 1608) using structural MRI. Given prior radiology reports of posterior fossa abnormalities in 3q29Del, we focused our investigation on the cerebellum and its tissue-types and lobules. Additionally, we compared the prevalence of cystic/cyst-like malformations of the posterior fossa between 3q29Del and controls and examined the association between neuroanatomical findings and quantitative traits to probe gene-brain-behavior relationships. 3q29Del participants had smaller cerebellar cortex volumes than controls, before and after correction for intracranial volume (ICV). An anterior-posterior gradient emerged in finer grained lobule-based and voxel-wise analyses. 3q29Del participants also had larger cerebellar white matter volumes than controls following ICV-correction and displayed elevated rates of posterior fossa arachnoid cysts and mega cisterna magna findings independent of cerebellar volume. Cerebellar white matter and subregional gray matter volumes were associated with visual-perception and visual-motor integration skills as well as IQ, while cystic/cyst-like malformations yielded no behavioral link. In summary, we find that abnormal development of cerebellar structures may represent neuroimaging-based biomarkers of cognitive and sensorimotor function in 3q29Del, adding to the growing evidence identifying cerebellar pathology as an intersection point between syndromic and idiopathic forms of neurodevelopmental disabilities.

## Introduction

Copy number variation (CNV) of DNA sequences (gain/loss of  >1-Kb genomic material) represents a significant source of genetic diversity [1–5] and a far more important substrate for human evolution and adaptation than previously recognized [6]. Accumulating findings indicate the existence of rare CNVs that increase susceptibility to multiple neurodevelopmental disorders trans-diagnostically [7–13]. Systematic investigation of these pathogenic CNVs can greatly further our understanding of quantitative traits and biological markers underlying this shared vulnerability for multiple neurodevelopmental conditions, including schizophrenia (SZ), autism spectrum disorder (ASD), intellectual disability (ID), and attention deficit hyperactivity disorder (ADHD), which exhibit considerable symptomatic and genetic overlap [14]. Noninvasive neuroimaging technologies, such as high-resolution structural magnetic resonance imaging (MRI), can be applied in combination with dimensional phenotyping of genetically-defined cohorts to bridge the gap between molecular/cellular mechanisms directly downstream of disease loci and behavioral endpoints that may traverse diagnostic categories. The integration of genomics and imaging is especially promising for high-impact CNVs with large effect sizes on disease risk, as these variants are expected to produce highly disruptive and relatively consistent deviations in brain structure with greater etiological salience. Such investigations can help disentangle the extensive heterogeneity observed across studies of brain structure in clinical populations defined solely based on diagnostic criteria [15–17].

The 3q29 deletion (3q29Del) is a relatively recently discovered CNV associated with an exceptionally increased burden of neurodevelopmental disability. 3q29Del has a prevalence of ~1 in 30,000 and usually arises de novo due to the hemizygous deletion of a 1.6-Mb locus, spanning 21 protein-coding genes [18, 19]. Cognitive ability in individuals with 3q29Del is typically in the range of mild-to-moderate ID, with deficits of variable severity observed in both non-verbal and verbal reasoning, a complex profile characterized by relative strengths in verbal compared to non-verbal IQ, and significant correlations identified between cognitive disability and adaptive functioning [18, 20]. Besides cognitive deficits, clinically significant deficits in visual-motor integration skills have been documented in 78% of individuals with this CNV [18], pointing to an intriguing co-occurrence of cognitive and sensorimotor dysfunction, congruent with the previously theorized “multiple deficit model” of neurodevelopmental disorders [21, 22]. Additionally, 3q29Del is now recognized as one of eight known CNVs associated with SZ at genome-wide significance [7], with an estimated >40-fold increased risk, amounting to one of the highest known effect sizes in the genetic landscape of SZ [7, 23–27]. This deletion also confers a > 30-fold increased risk for ASD, and exhibits pleiotropy for ADHD, anxiety disorders and a variety of multi-systemic phenotypes [18, 20, 28–30]. Although several genes have been proposed as putative drivers [31, 32], it is only beginning to be understood how genetic variation is translated to abnormal neurodevelopmental phenotypes via systems-level brain function.

In a recent study, our group performed deep-phenotyping in the largest known sample of 3q29Del participants, using gold-standard instruments and structural MRI [18]. Neuroradiological inspection revealed abnormal posterior fossa structures severe enough to be qualitatively detectable by eye in over 70% of participants, including cerebellar hypoplasia and cystic/cyst-like malformations, suggesting that development of the posterior fossa may be particularly vulnerable to the impact of 3q29Del. However, the etiopathologic significance of these neuroanatomical deviations has not yet been investigated.

Here, we report the first in vivo quantitative structural MRI study in individuals with 3q29Del using case-control analysis and quantitative investigation of brain morphology. Motivated by previous radiological findings on marked posterior fossa abnormalities in this syndrome [18], we focused our investigation on volumetric properties of the cerebellum, its primary tissue-types and lobules. Additionally, we expanded our previous radiological investigation to the whole brain to evaluate whether the distribution of radiologically diagnosable anomalies shows enrichment for the posterior fossa over other brain regions. We further tested whether the prevalence of cystic/cyst-like malformations of the posterior fossa, which are typically classified as “incidental findings” in the clinic [33–35], are enriched in 3q29Del as compared to controls. Finally, given accumulating evidence implicating the cerebellum in higher-order functions besides sensorimotor control [36–39], we tested the hypothesis that neuroanatomical findings are related to cognitive and sensorimotor deficits in 3q29Del by employing a dimensional approach to probing gene-brain-behavior relationships consistent with the Research Domain Criteria (RDoC) framework [40].

The findings reported in the present study have important implications for neuroimaging-based biomarker discovery in 3q29Del with links to quantitative neurodevelopmental traits, and open novel directions for focusing cellular and molecular studies on target brain regions and circuits. These data also add to our knowledge of genetic loci that subserve the normative development of the posterior fossa, particularly the cerebellum, which is receiving growing attention in relation to neurodevelopmental disability and cognitive dysfunction.

## Methods and materials

### Participants

#### 3q29Del

3q29Del participants were ascertained from the 3q29 Registry [29] and traveled to Atlanta, GA for evaluation with standardized tools and structural MRI, as described previously [18, 41]. A total of 25 participants with 3q29Del consented to undergo MRI. Data from 24 individuals with 3q29Del, ages 4–39 years (mean ± SD = 14.71 ± 9.21 years, 62.50% male) were included in this study. One participant was retained for radiological examination but excluded from volumetric analyses due to inability to extract reliable measures. Hence, success rate for acquiring high quality volumetric data was 92% (23 out of 25). 3q29Del status was confirmed via clinical genetics reports and/or medical records. Participants provided informed consent/assent; for minors, a parent/legal guardian additionally provided consent. All procedures were approved by the Emory University Institutional Review Board (IRB000088012). See Fig. S1 for major neurodevelopmental and psychiatric diagnoses identified in 3q29Del participants.

#### Neurotypical controls

To achieve reliable estimates for the structural variability observed in typically developing brains, we used MRI data from the largest available, open-access sample of neurotypical controls from the Human Connectome Project (HCP; *N* = 1765), with cross-sectional continuity over a wide age-range (5–37 years) overlapping with the age-range of the 3q29Del sample. In recent years, efforts to integrate large-scale MRI datasets across multiple sites and scanners have enabled the generation of normative “brain charts” that allow age-normed quantification of MRI metrics, with demonstrated robustness to technical differences [42]. This affords increased sensitivity to detect genetic effects on brain structure. In keeping with this framework, we leveraged the HCP dataset to establish normative benchmarks around our study metrics, while accounting for linear and non-linear growth trajectories and sex differences. Based on reported family relationships, one sibling from each monozygotic twin-pair was removed to minimize bias in standard errors [43, 44]; hence, 1608 controls (mean ± SD = 22.73 ± 8.21 years, 46.46% male) were included. See [45, 46] for HCP eligibility criteria.

**Fig. 1:**
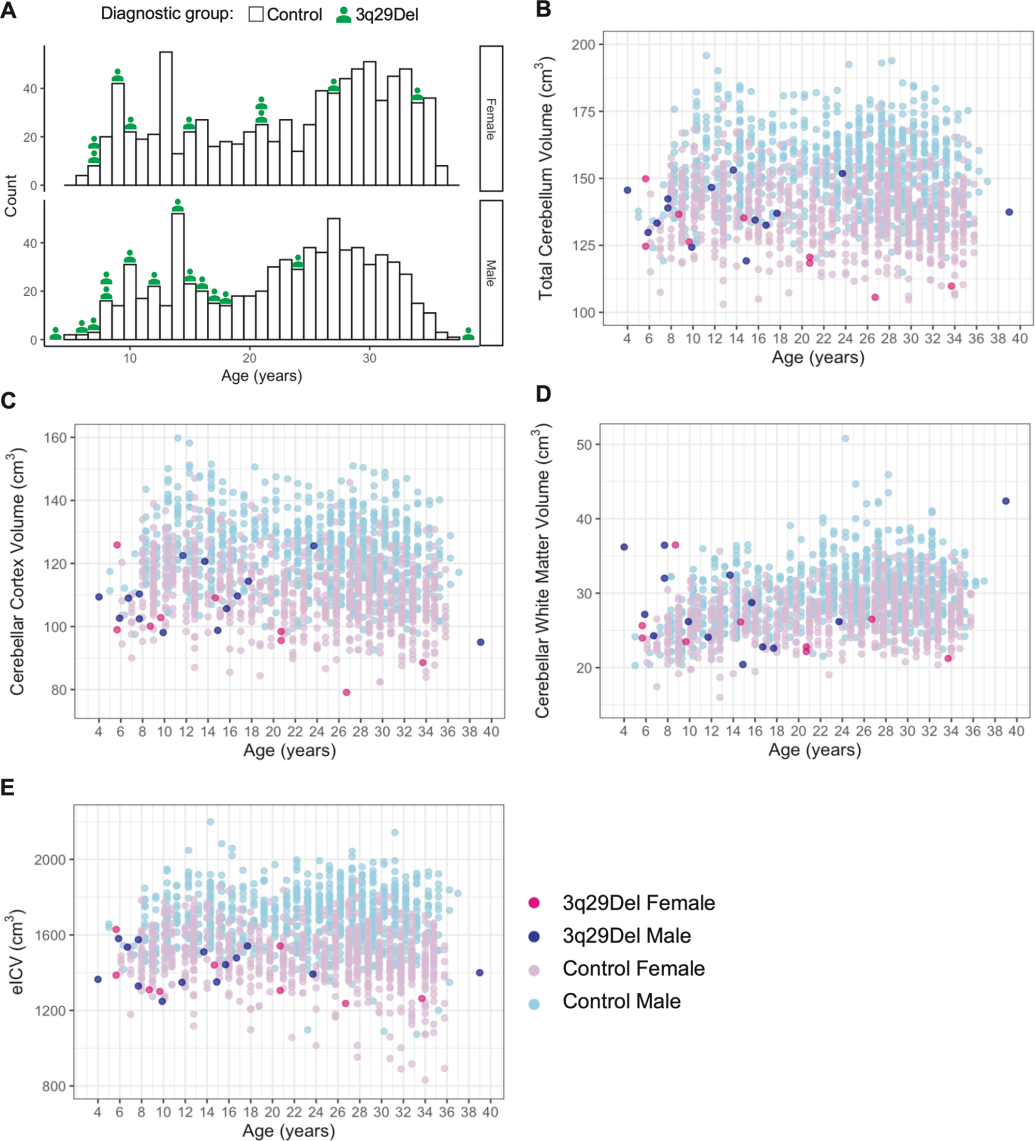
Structural magnetic resonance imaging of 3q29Del participants and neurotypical controls. **A** Histogram showing the age distribution of study participants in volumetric analyses, stratified by sex and diagnostic group. To define reliable estimates for the normative trajectory of cerebellar volumetric change across age, we used the largest available open-access sample of neurotypical controls from the Human Connectome Project, which cross-sectionally covers a wide age-range [5–37 years] with near complete overlap with the age-range of the 3q29Del sample [4–39 years]. Each histogram bin represents one year. Green icons above each bar symbolize the number of 3q29Del participants included in volumetric analyses for a given age and sex. **B–E** Scatter plots showing the distribution of total cerebellum volume, cerebellar cortex volume, cerebellar white matter volume, and eICV as a function of age among male and female participants in each diagnostic group. A slight jitter was added systematically to all panels to minimize overplotting. Data reflect FreeSurfer-based absolute volumes. Control *N* = 1608 (Female *N* = 861, Male *N* = 747), 3q29Del *N* = 23 (Female *N* = 9, Male *N* = 14). 3q29Del 3q29 deletion syndrome, eICV estimated total intracranial volume.

There were no significant differences in the sex, ethnicity, or race compositions of the two groups (*p’s* > 0.05). While there was a near complete overlap between age-ranges, the 3q29Del group was relatively younger than controls (*p* ≤ 0.001) (Table 1, Fig. 1A, Table S1); hence, we control for age in downstream analyses.

**Table 1:** Demographic characteristics of the study sample in volumetric analyses, stratified by diagnostic group.

Demographic variables	Control *N* = 1608	3q29Del *N* = 23	Test statistics
Age (in years)			
Mean ± SD	22.73 ± 8.21	15.09 ± 9.22	*r* = 0.10 (small effect size), *W* = 9429.5, *p* value^*a*^ = 5.26E-05***
Median	25.00	14.00	
Range	5–37	4–39	
Sex, *N* (%)			
Male	747 (46.46%)	14 (60.87%)	*X*^*2*^ = 1.36, DF = 1, *p* value^*b*^ = 0.24
Female	861 (53.54%)	9 (39.13%)	
Ethnicity, *N* (%)^*#*^			
Non-Hispanic / Latino	1397 (88.03%)	22 (95.65%)	*X*^*2*^ = 0.64, DF = 1, *p* value^*b*^ = 0.42
Hispanic / Latino	190 (11.97%)	1 (4.35%)	
Race, *N* (%)^*##*^			
White	1112 (70.66%)	21 (91.30%)	*X*^*2*^ = 6.25, DF = 4, *p* value^*b*^ = 0.18
Black / African American	222 (14.10%)	0 (0%)	
Asian / Native Hawaiian / Other Pacific Islander	108 (6.86%)	0 (0%)	
American Indian / Alaskan Native	4 (0.25%)	0 (0%)	
More Than One Race	128 (8.13%)	2 (8.70%)	

*3q29Del* 3q29 deletion syndrome, *SD* standard deviation, *DF* degrees of freedom.

There were no significant differences in the sex, ethnicity, or race compositions of the two diagnostic groups (*p’s* > 0.05). While there was a near complete overlap between the age-ranges of the two groups, there was a significant age difference between 3q29Del participants and controls on average (*p* ≤ 0.001). Effect sizes are reported for significant test results only. Non-parametric statistics are reported in cases where the data do not meet parametric assumptions. ^*#*^Control *N* = 1587 for the ethnicity variable due to missing data. ^*##*^Control *N* = 1574 for the race variable due to missing data. Corresponding percentages reflect the fraction of controls with complete data. ^*a*^Wilcoxon rank sum test, ^*b*^Pearson’s chi-squared test. *p* value ≤ 0.001 ‘***’, *p* value ≤ 0.01 ‘**’, *p* value ≤ 0.05 ‘*’, *p* value ≤ 0.1.

### Structural MRI acquisition

High-resolution structural MRI data were collected from 3q29Del participants on a 3T Siemens Magnetom Prisma scanner, using a 32-channel head coil and an 80mT/m gradient. T1-weighted 3D images were acquired in the sagittal plane using a single-echo MPRAGE sequence [47] with the following parameters: TE = 2.24 ms, TR = 2400 ms, TI = 1000 ms, bandwidth = 210 Hz/pixel, FOV = 256x256mm, Resolution = 0.8 mm isotropic. T2-weighted 3D images were acquired in the sagittal plane using a SPACE sequence [48] with the following parameters: TE = 563 ms, TR = 3200 ms, bandwidth = 745 Hz/pixel, FOV = 256x256 mm, Resolution = 0.8 mm isotropic.

Control participants were imaged using a 3T Siemens Prisma or Skyra scanner by the HCP Development and Young Adult consortiums, using a 32-channel head coil with 80 or 100 mT/m gradients. T1- and T2-weighted 3D images were acquired in the sagittal plane using single- or multi-echo MPRAGE and SPACE sequences, respectively [46, 49, 50]. Comparison of 3q29Del and control protocols indicate that fundamental aspects of the hardware and acquisition parameters are identical or highly comparable for combined testing (Table S2).

### Image processing

For volumetrics, all 3q29Del scans were first processed with the HCP “minimal pre-processing” pipeline to remove spatial artifacts/distortions, align images to MNI space and obtain brain masks/parcellations [51]. Image processing for controls was performed by the HCP using this common framework. FreeSurfer (https://surfer.nmr.mgh.harvard.edu) was used for automated segmentations as a widely-used and well-validated pipeline that uses probabilistic information estimated from a manually labeled training set [52]. This algorithm is especially suitable for multi-center data, as it demonstrates low sensitivity to noise and variable image quality [53, 54]. Using this approach, we began our investigation by extracting tissue-specific volumetric measures for cerebellar cortex (mainly comprised of gray matter) and cerebellar white matter (WM) to distinguish the two main structural divisions within the cerebellum; total cerebellar volume was defined as the sum of these metrics.

For quality control (QC), two trained evaluators (ES, LL) inspected all FreeSurfer segmentations obtained from 3q29Del participants to determine whether technical problems or notable pathology interfere with registration or segmentation quality. Detailed QC for the HCP dataset is available at https://wiki.humanconnectome.org/. To facilitate joint analysis, we additionally cross-compared the image quality, tissue contrast and segmentation masks of age- and sex-matched case-control pairs and found no systematic irregularities; no manual intervention was performed.

Next, we took a finer grained approach, and divided the cerebellar cortex into smaller subregions, using a deep learning-based convolutional neural network algorithm, called the Automatic Cerebellum Anatomical Parcellation using U-Net Locally Constrained Optimization (ACAPULCO) [55, 56]. Manual corrections were required during QC of subregional volumes, likely due to the more severe nature of posterior fossa abnormalities seen in 3q29Del, compared to the original samples used for model training, which failed to generalize effectively. To ensure the level of structural precision necessary for in-depth subregional analyses and to reduce bias in the distribution of parcellation errors encountered in case-control comparisons, we incorporated a slice-by-slice manual correction step in 2D axial, coronal, and sagittal planes, along with a 3D reconstruction-based manual correction step with ITK-SNAP (https://itksnap.org/). The following 17 subregions were extracted: hemispheric lobules I-V, lobule VI, Crus I, Crus II/lobule VIIB, lobule VIII, lobule IX, lobule X, and vermal lobules I-V, VI-VII, VIII-X.

Finally, the Spatially Unbiased Infratentorial Template (SUIT) toolbox [57, 58] was used to perform a supplemental voxel-based morphometry (VBM) analysis to achieve enhanced flexibility in capturing group differences in cerebellar gray matter, without reliance on predefined boundaries between lobules. The preprocessing steps for SUIT-VBM included tissue segmentation, gray matter normalization using non-linear deformation, modulation, and spatial smoothing (using a 6 mm full width at half maximum Gaussian kernel), as described in [59]. Cerebellum was masked with gray matter probability >0.2 (171,796 voxels in the mask). Correct warping to the template was confirmed for each participant.

We note that presenting results from ACAPULCO, SUIT-VBM and FreeSurfer reinforces the validity of our results and allows for comparison with findings from a larger range of studies using either method. See Supplemental Materials and Figs. S2–4 for extended QC and methodological details.

### Normalization of cerebellar volumes

An important consideration in deciding whether and how regional brain volumes should be adjusted for head size, is the potential relevance of this variable to altered neurodevelopmental processes [60, 61]. Given prior reports of microcephaly in 3q29Del [30], this question is particularly germane to this study. We used the estimated total intracranial volume (eICV) generated by FreeSurfer as a proxy for head size [62], and evaluated the relationship between eICV and tissue-specific volumetric measures for cerebellar cortex and WM in each group. Most cerebellar volumes did not scale proportionally with eICV, and the relationship between cerebellar WM volume and eICV changed as a function of diagnostic group (*p* = 0.03) (Fig. S5). Consequently, all cerebellar volumes were adjusted for eICV using the “residual” method (Fig. S6), based on normative regression slopes derived from controls, as described in [60, 63, 64].

### Radiological evaluation of MRI scans

Prior work by our group using the same 3q29Del scans as in the present study has shown that posterior fossa arachnoid cysts (PFAC) are common among 3q29Del carriers [18]. However, our original report did not explicitly evaluate regions outside the posterior fossa. To probe for patterns of regional specificity, all 3q29Del scans (*N* = 24) were reviewed qualitatively by a board-certified neuroradiologist (AEGY) at the whole-brain level using Horos (https://horosproject.org). Given well-established challenges in differentiating PFAC from mega cisterna magna (MCM) [65, 66], their prevalence rates were considered jointly. We also pulled radiographic data on incidental findings in neurotypical controls (*N* = 1608) to define normative prevalence estimates for cystic/cyst-like malformations. Prior to group comparisons, the neuroradiologist who evaluated the 3q29Del scans independently confirmed the control findings for consistency.

### Standardized behavioral measures

Case-control studies typically focus on a single psychiatric diagnosis despite evidence of trans-diagnostically shared impairments in multiple behavioral domains. For quantitative characterization of the latter, 3q29Del participants completed a standardized, norm-referenced battery of cognitive, motor and sensory tests administered by trained professionals [18, 20, 41]. Cognitive abilities (composite, verbal, and non-verbal IQ) were assessed using the *Differential Ability Scales* (DAS, 2^nd^ edition) among participants aged 4-17 years [67], and the *Wechsler Abbreviated Scale of Intelligence* (WASI, 2^nd^ edition) among participants aged 18-39 years [68]. The *Beery-Buktenica Developmental Test of Visual-Motor Integration* (VMI, 6^th^ edition) was administered to assess the ability to integrate fine motor skills with visual information in geometric design-copying tasks, and supplemental tests were used to measure visual perception and fine motor abilities separately [69]. Behavioral measures were available for 3q29Del participants only.

### Statistical analyses

#### Case-control comparison of volumetric measures

All statistical analyses were performed using *R* version 4.0.3 [70]. Demographic characteristics were compared using Wilcoxon rank sum test and Pearson’s chi-squared test. Multiple linear regression was used to test for group differences in both absolute and eICV-adjusted total cerebellum, cerebellar cortex and WM volumes, as well as in eICV, while correcting for sex and age. The distributions of eICV, and absolute and eICV-adjusted cerebellar volumes tested in tissue-specific analyses are visualized in Fig. 1B-E, and Fig. S7. Analyses of variance (ANOVA) were performed to identify the best-fitting polynomial function of age for each of these volumes of interest (VOIs) (see Table S3 for details). Multiple comparisons correction was applied (7 tests) to control the false discovery rate (FDR) using the Benjamini-Hochberg procedure. In cases where violations were observed for ordinary least squares regression (see Fig. S8), heteroscedasticity-robust estimates were calculated using the HC1 robust standard error estimator [71]. Since reliance on asymptotic theory can be problematic in small-to-moderate sample sizes, exact *p* values were additionally calculated using marginal permutation tests (10,000 random permutations).

To evaluate whether these main effects change as a function of sex or age, we also performed exploratory analyses of diagnostic group by sex, and diagnostic group by age interactions. When a significant product term was identified (*p* ≤ 0.05), males and females were subsequently tested separately and the interaction effect with age was visualized for inspection of underlying trends. Given the exploratory nature of these analyses, FDR correction was not applied.

In supplemental analyses, non-parametric spline modeling was adopted to build volumetric trajectories with increased flexibility and to estimate normative percentile curves for total cerebellum, cerebellar cortex, cerebellar WM, and eICV akin to growth curves. Since splines can capture a wide range of nonlinear trends, penalized cubic spline and quantile spline methods were incorporated to improve the statistical rigor of our central analyses. Finally, we performed a matched case-control analysis of these VOIs for additional confirmation of our main findings in the full study sample, using exact match on sex and nearest available match on age, with a 1:4 case-control ratio.

For finer-grained lobule-specific analyses, both absolute and eICV-adjusted group differences in 17 subregions of the cerebellar cortex were tested in the same age-and sex-matched case-control sample described above, using two-sample Student’s t-tests. Multiple comparisons correction was applied (34 tests) using the Benjamini-Hochberg procedure. Furthermore, supplemental VBM analyses were additionally carried out in this age-and sex-matched sample, using two-sample t-tests for voxel-wise group comparisons of pre-processed cerebellar gray matter volumes, with and without adjustment for eICV. Since these data are spatially smooth, and voxels are correlated with their neighbors, the Gaussian random field theory with Family-Wise Error (FWE) at *p* ≤ 0.05 was used to account for multiple dependent comparisons at the voxel level. To determine the anatomical position of the peak voxels of significant clusters identified through VBM, the SUIT cerebellar atlas viewer (https://www.diedrichsenlab.org/imaging/AtlasViewer/) was used. Interaction effects with sex and age were not assessed in this subsample due to limited statistical power.

#### Case-control comparison of radiological findings

The prevalence of PFAC/MCM findings was compared between 3q29Del and control groups using Fisher’s exact test. Sex-specific differences were evaluated within each group using the same procedure. Demographic characteristics of 3q29Del participants with versus without cystic/cyst-like malformations were compared using Student’s two sample t-test and Fisher’s exact test. To rule out conceivable secondary (acquired) etiologies [72–74], differences in the distribution of past head injuries, maternal complications during pregnancy and neonatal complications during delivery were tested between these groups using Fisher’s exact test. To determine whether the likelihood of cystic/cyst-like malformations covary with volumetric measures among 3q29Del participants, relationships between PFAC/MCM findings and VOIs were modeled using multiple linear regression, with sex, age, and eICV considered as covariates.

#### Tests of brain-behavior relationships in 3q29Del

To probe the functional relevance of our tissue-specific MRI findings, we first investigated the association of tissue-specific cerebellar cortex and WM volumes with composite IQ and VMI scores among 3q29Del participants in separate linear regression models, with adjustment for age and sex. Secondary analyses were conducted to investigate which cognitive (verbal / non-verbal) and sensorimotor (visual perception / fine motor coordination) subprocesses may be associated with cerebellar volumes. Multiple comparisons correction was applied at the behavioral domain level (6 metrics). In models where a significant relationship was identified (*p* ≤ 0.05), eICV was subsequently added as an additional covariate to test whether results reflect a link with the cerebellum beyond global variability in head size. Lobule-specific relationships between behavioral measures and 17 subregions of the cerebellar cortex were additionally tested using the same statistical framework.

Finally, to examine the functional relevance of radiological findings, standardized test scores of 3q29Del participants with versus without cystic/cyst-like malformations were compared using Student’s two sample t-test and Wilcoxon rank sum test. Standard diagnostics were performed to check all statistical assumptions. If parametric assumptions were not met, non-parametric alternatives were used. All analyses were two-tailed. Detailed methods are provided in Supplemental Materials.

## Results

### Case-control differences in tissue-specific volumetric measures

**Fig. 2:**
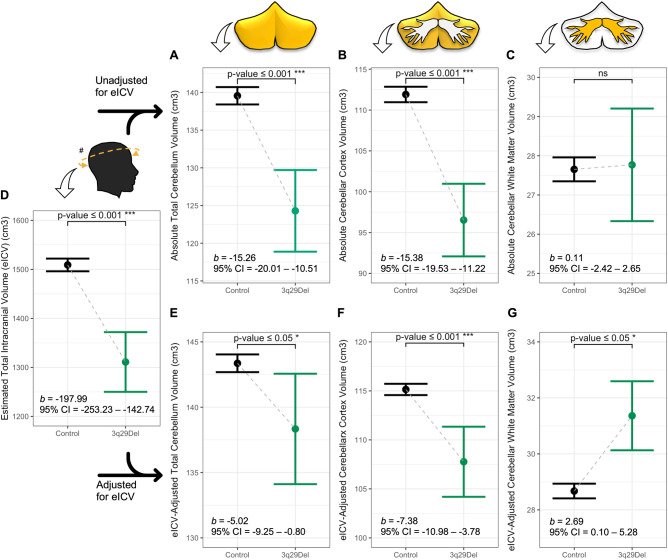
Predictor effect plots showing the effect of diagnostic group on tissue-specific cerebellar volumes and eICV. **A–G** Predicted values of FreeSurfer-based VOIs across the 3q29Del and control groups were computed from the best-fitting multiple linear regression models reported in Table S3, while covariates (sex, age, age^2^ (when applicable)) were held fixed. Error bars indicate the 95% confidence interval. *P* values for the main effect of diagnostic group are indicated on each plot and reflect heteroskedasticity-robust estimates. A schematic of each VOI (yellow) is presented above the corresponding plot for clarity. Regression results indicate a significant difference between 3q29Del participants and neurotypical controls in (**A**) total cerebellum volume (*p* ≤ 0.001), (**B**) cerebellar cortex volume (*p* ≤ 0.001), and (**D**) eICV (*p* ≤ 0.001), with smaller volumes observed in 3q29Del participants compared with controls. This effect remained significant after (**E**) total cerebellum volume (*p* ≤ 0.05) and (**F**) cerebellar cortex volume (*p* ≤ 0.001) were adjusted for eICV. There was no significant effect of diagnostic group on (**C**) absolute cerebellar white matter volume (*p* > 0.05), however (**G**) after eICV-adjustment, 3q29Del participants had significantly larger cerebellar white matter volumes than controls (*p* ≤ 0.05). All findings survived multiple comparisons correction (*FDR-adjusted* *p* value ≤ 0.05). Control *N* = 1608, 3q29Del *N* = 23. ^#^eICV was calculated by FreeSurfer’s atlas-based spatial normalization procedure. *p* value ≤ 0.001 ‘***’, *p* value ≤ 0.01 ‘**’, *p* value ≤ 0.05 ‘*’, *p* value ≤ 0.1 *‘*^*†*^*’* 3q29Del 3q29 deletion syndrome, VOI volumetric measure of interest, eICV estimated total intracranial volume, *b* unstandardized coefficient estimate, CI confidence interval, FDR false discovery rate, ns not significant.

Using multiple linear regression, we first tested for diagnostic group differences in our tissue-specific VOIs, while correcting for age and sex (Table S3A-H, Fig. 2A-G). Participants with 3q29Del had significantly smaller eICVs than controls (*b* = −197.99, *p* ≤ 0.001, *FDR-adjusted p* ≤ 0.001); hence, we report case-control comparisons for both absolute and eICV-adjusted volumes while examining regional changes.

The 3q29Del group had significantly smaller total cerebellum volumes than controls (*b* = −15.26, *p* ≤ 0.001, *FDR-adjusted p* ≤ 0.001), and this finding persisted after eICV-adjustment (*b* = −5.02, *p* = 0.02, *FDR-adjusted p* = 0.03). When broken down to tissue-types, cerebellar cortex volumes were significantly smaller in the 3q29Del group (*b* = -15.38, *p* ≤ 0.001, *FDR-adjusted p* ≤ 0.001), while cerebellar WM volumes did not differ between groups (*p* = 0.93). Case-control differences in cerebellar cortex remained significant after eICV-adjustment (*b* = −7.38, *p* ≤ 0.001, *FDR-adjusted p* ≤ 0.001). Unexpectedly, 3q29Del participants also had significantly larger cerebellar WM volumes than controls after eICV-adjustment (*b* = 2.69, *p* = 0.04, *FDR-adjusted p* = 0.05). Results from sensitivity analysis, after a 39-year-old 3q29Del participant falling outside the control age-range was removed, showed consistent patterns with original analyses (Table S4A–G). Considering these findings, we also tested cerebellar cortex to WM volume ratios for group differences (Table S3D), which revealed smaller ratios in 3q29Del (*b* = −0.49, *p* ≤ 0.01).

As an ancillary method, we fit generalized additive models (GAM) with a cubic spline basis to our data to test for group differences with greater flexibly in modeling age. We also repeated these comparisons using a matched case-control study design with a balanced distribution of age and sex across groups to minimize confounding effects. Both GAM results (Table S5, Fig. S9) and matched case-control comparisons (Table S6) support the tissue-specific VOI differences identified between groups using linear regression.

In exploratory analyses, we evaluated whether the effects of diagnostic group change as a function of sex, while correcting for age (Table S7A–G). There was a significant interaction between diagnostic group and sex on eICV (*b* = −149.64, *p* ≤ 0.01). Both male and female 3q29Del participants had smaller eICVs than controls; however, this reduction was greater among male (*b* = −237.09, *p* ≤ 0.001) than female 3q29Del participants (*b* = −119.89, *p* ≤ 0.01) (Fig. S10A, Table S8A). Both OLS-based asymptotic *p* values and exact *p* values by permutation testing were concordant with heteroscedasticity-robust estimates across these analyses. Additionally, OLS regression and permutation testing indicated an interaction between diagnostic group and sex on eICV-adjusted cerebellar WM volumes (*b* = 2.73, *p* = 0.03); however, this effect failed to reach significance when heteroscedasticity-robust estimates were calculated (*p* = 0.29) (see Fig. S10B, Table S8B for sex-stratified results).

We also evaluated whether the effects of diagnostic group change as a function of age, while correcting for sex (Table S9A–G). OLS regression and permutation testing revealed a diagnostic group by age interaction on absolute (*b* = −0.16, *p* = 0.04) and eICV-adjusted cerebellar WM volumes (*b* = −0.13, *p* = 0.05). Visual inspection of the underlying trends showed an aberrant trajectory of volumetric growth, with an earlier developmental peak observed in the cerebellar WM volumes of 3q92Del participants (Fig. S11). However, these interaction effects failed to reach significance when heteroscedasticity-robust estimates were calculated.

Finally, using quantile splines as a supplemental method, we estimated sex-specific normative percentile curves for our tissue-specific VOIs akin to growth charts. Fig. S12 visualizes the cerebellar volumes and eICV of each 3q29Del participant relative to reference standards in controls, showing that many 3q29Del participants fall into extreme percentiles of growth, although within-group variability is observed in individual percentile ranks.

### Case-control differences in lobule-specific and voxel-level volumetric measures

**Fig. 3:**
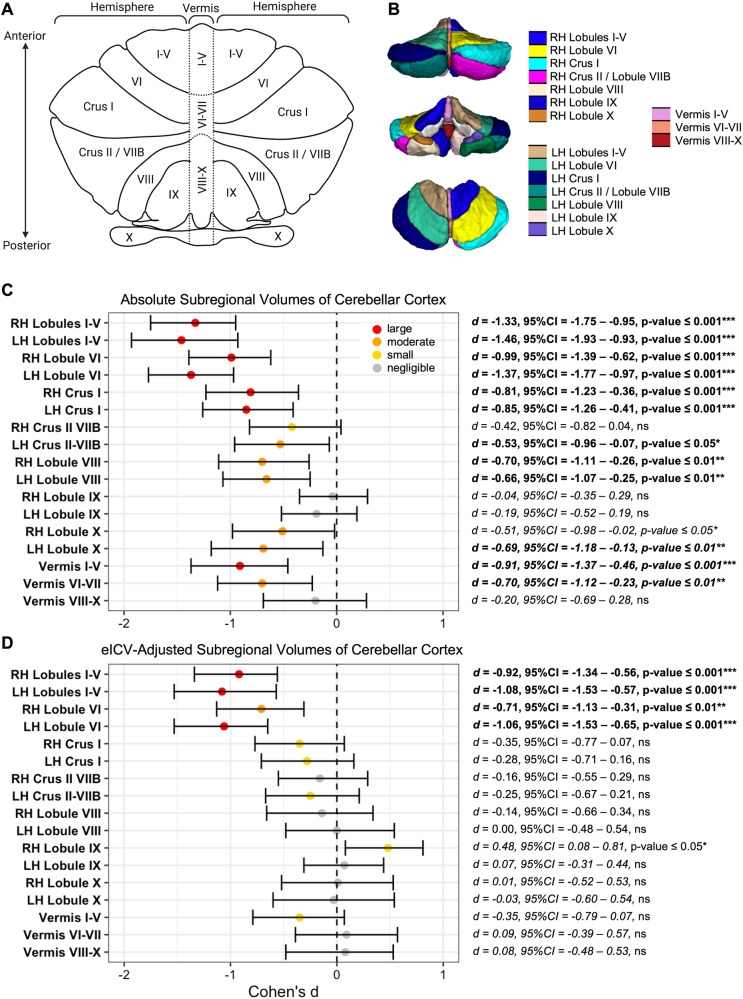
Subregional investigation of the effect of diagnostic group on lobule-specific cerebellar cortex volumes. **A** A flat map diagram of the cerebellum is displayed to visualize the approximate anatomical location of the 17 subregions that the cerebellar cortex was divided into with ACAPULCO. **B** An example 3D reconstruction of an ACAPULCO-based parcellation mask for a representative neurotypical control. **C**, **D** Forest plots of Cohen’s *d* effect sizes and corresponding 95% confidence intervals show the effect of diagnostic group on lobule-specific cerebellar cortex volumes in our age- and sex-matched study sample (case:control ratio = 1:4). Color represents effect size magnitude. Findings that survived multiple comparisons correction (*FDR-adjusted* *p* value ≤ 0.05) are indicated in bold. Tests for absolute volume are shown in (**C**). Tests for eICV-adjusted volumes are shown in (**D**). Detailed summary statistics are reported in Table S10. Control *N* = 92, 3q29Del *N* = 23. *p* value ≤ 0.001 ‘***’, *p* value ≤ 0.01 ‘**’, *p* value ≤ 0.05 ‘*’, *p* value ≤ 0.1 *‘*^*†*^*’*. Thresholds for quantification of effect size magnitude: |d | < 0.2 “negligible”, |d | < 0.5 “small”, |d | < 0.8 “medium”, otherwise “large”. ACAPULCO Automatic Cerebellum Anatomical Parcellation using U-Net Locally Constrained Optimization, 3q29Del 3q29 deletion syndrome, eICV estimated total intracranial volume, *d* Cohen’s d statistic, CI confidence interval, FDR false discovery rate, ns not significant.

The distributions of absolute and eICV-adjusted lobular volumes, derived by ACAPULCO-based finer segmentation of the cerebellar cortex into 17 subregions are visualized in Fig. S13. Approximate location of each lobule is illustrated in Fig. 3A, B. In our age- and sex-matched subsample, we found significant reductions in the absolute hemispheric volumes of bilateral lobules I-V (right Cohen’s *d* = −1.33, left Cohen’s *d* = −1.46), bilateral lobule VI (right Cohen’s *d* = −0.99, left Cohen’s *d* = −1.37), bilateral crus I (right Cohen’s *d* = −0.81, left Cohen’s *d* = −0.85), left crus II / lobule VIIB (Cohen’s *d* = −0.53), bilateral lobule VIII (right Cohen’s *d* = −0.70, left Cohen’s *d* = −0.66), bilateral lobule X (right Cohen’s *d* = −0.51, left Cohen’s *d* = −0.69), as well as in the absolute vermal volumes of lobules I-V (Cohen’s *d* = −0.91) and lobules VI-VII (Cohen’s *d* = −0.70) in the 3q29Del group relative to controls (*p’s* ≤ 0.05) (Table S10, Fig. 3C). All findings, except for the group difference in the absolute volumes of right hemispheric lobule X, survived multiple comparisons correction (*FDR-adjusted p’s* ≤ 0.05). In addition, a trend level reduction was observed in the absolute volumes of right hemispheric crus II / lobule VIIB in the 3q29Del group (Cohen’s *d* = −0.42, *p* ≤ 0.10), however this finding did not reach statistical significance.

After eICV-adjustment, group differences remained significant only in the hemispheric volumes of bilateral lobules I-V (right Cohen’s *d* = −0.92, *p* ≤ 0.001; left Cohen’s *d* = −1.08, *p* ≤ 0.001), and bilateral lobule VI (right Cohen’s *d* = −0.71, *p* ≤ 0.01; left Cohen’s *d* = −1.06, *p* ≤ 0.001), with smaller volumes observed in the 3q29Del group relative to controls (Table S10, Fig. 3D). The effects on the left hemisphere were larger than the right hemisphere in both subregions. All four findings survived multiple comparisons correction (*FDR-adjusted* *p’s* ≤ 0.01). In addition, after eICV-adjustment, larger right hemispheric lobule IX volumes were identified in the 3q29Del group (Cohen’s *d* = 0.48, *p* ≤ 0.05), however this finding did not survive multiple comparisons correction.

Supplemental cerebellar VBM analyses with SUIT yielded T-maps of statistically significant cerebellar gray matter volume differences between groups at the voxel-level, with an anterior-to-posterior gradient in effect sizes, consistent with findings from ACAPULCO (Fig. S14–S16). VBM results without eICV adjustment captured case-control differences in more cerebellar subregions compared to ACAPULCO, which may be driven by localized volumetric differences present within specific lobules that do not generalize to the entire lobule. VBM results after adjustment for eICV highlighted multiple clusters of cerebellar gray matter loss among 3q29Del participants, with peak voxels in regions corresponding to bilateral lobules I-V, VI, Crus I, Crus II, left lobule VIIb, and left lobule X (FWE-adjusted *p’s* ≤ 0.05). To be more stringent, we focus our discussion of subregional case-control differences within the cerebellum on lobules implicated by both ACAPULCO and SUIT-VBM.

### Case-control differences in radiological findings

**Fig. 4:**
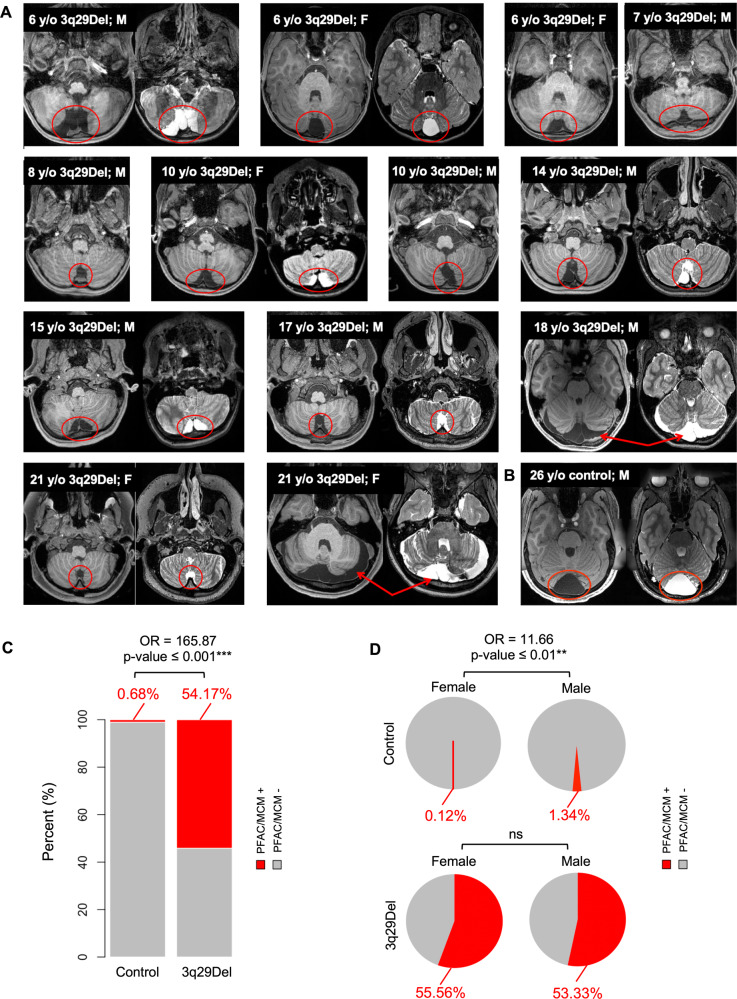
Prevalence of posterior fossa arachnoid cyst and mega cisterna magna findings in structural MRI scans of 3q29Del and control participants. **A** 13 3q29Del participants had a PFAC/MCM finding (marked in red) upon radiological evaluation of structural MRI scans. Representative T1- and/or T2-weighted MR images showing these radiological findings are provided in the axial plane for each 3q29Del participant, in chronological order of age. **B** T1- and T2-weighted MR images of a representative control participant with a PFAC/MCM finding (marked in red). Age and sex information for each participant is indicated on individual images. **C** Bar graphs represent the frequency of PFAC/MCM findings in the 3q29Del and control groups, separately. 3q29Del participants had a significantly elevated rate of PFAC/MCM findings compared with controls (*p* ≤ 0.001). **D** Pie charts represent the sex-stratified frequency of PFAC/MCM findings in the 3q29Del and control groups, separately. Male controls had a significantly elevated rate of PFAC/MCM findings compared with female controls (*p* ≤ 0.01), while there were no sex differences in these rates within the 3q29Del group (*p* > 0.05). Control *N* = 1608 (Female *N* = 861, Male *N* = 747), 3q29Del *N* = 24 (Female *N* = 9, Male *N* = 15). *p* value ≤ 0.001 ‘***’, *p* value ≤ 0.01 ‘**’, *p* value ≤ 0.05 ‘*’, *p* value ≤ 0.1 ‘^†^’. 3q29Del 3q29 deletion syndrome, PFAC posterior fossa arachnoid cyst, MCM mega cisterna magna, MRI magnetic resonance imaging, y/o years-old, M male, F female, OR odds ratio, ns not significant.

Upon radiological evaluation, 0.68% of controls (11 out of 1,608) and 54.17% of 3q29Del participants (13 out of 24) were found to have a PFAC/MCM finding. Comparison of these rates indicated a significantly higher prevalence in 3q29Del than controls (OR = 165.87, *p* ≤ 0.001) (Fig. 4A–C). In sex-stratified analyses, among controls, 0.12% of females (1 out of 861) and 1.34% of males (10 out of 747) had a PFAC/MCM, while 55.56% of females (5 out of 9) and 53.33% of males (8 out of 15) had a PFAC/MCM in 3q29Del (Fig. 4D). Comparison of these sex-specific rates indicated an increase among male compared with female controls (OR = 11.66, *p* ≤ 0.01), but no significant difference between male and female 3q29Del participants (OR = 0.92, *p* > 0.05).

There were no significant differences between 3q29Del participants with versus without PFAC/MCM findings in age, ethnicity, or race, nor in the distribution of past head injuries, maternal complications during pregnancy and/or neonatal complications during delivery (*p’s* > 0.05) (Table S10), suggesting that these findings are most likely of primary (congenital) origin and cannot be readily explained by secondary causes.

Besides PFAC/MCM findings (54.17%, out of 24), radiological examination of the posterior fossa also revealed a formal diagnosis of cerebellar hypoplasia in 11 3q29Del participants (45.83%). Notably, in our whole-brain examination, only one 3q29Del participant (4.17%) had an arachnoid cyst outside the posterior fossa (left parietal lobe), highlighting the increased vulnerability of the posterior fossa to cystic/cyst-like malformations in this syndrome. In addition, whole-brain examination revealed three cases of cavum septum pellucidum (12.5%), two cases of unfused posterior arch of C1 (8.33%), one pituitary hyperplasia (4.17%), one left periatrial WM gliosis (4.17%), one plagiocephaly (4.17%), and one case of mild cerebral volume loss (4.17%) among 3q29Del participants outside the posterior fossa. However, even when considered cumulatively, the prevalence of these anomalies did not reach the same level of localized prevalence as the radiological anomalies seen in the posterior fossa. Lastly, regression results indicated no significant association between the likelihood of PFAC/MCM findings and volumetric variation in the cerebellum or eICV of 3q29Del participants (*p’s* > 0.05) (Table S12, Fig. S17), implying that tissue-specific changes identified in the present study are not simply a byproduct of the cystic/cyst-like malformations enriched in this region.

### Brain-behavior relationships in 3q29Del

**Fig. 5:**
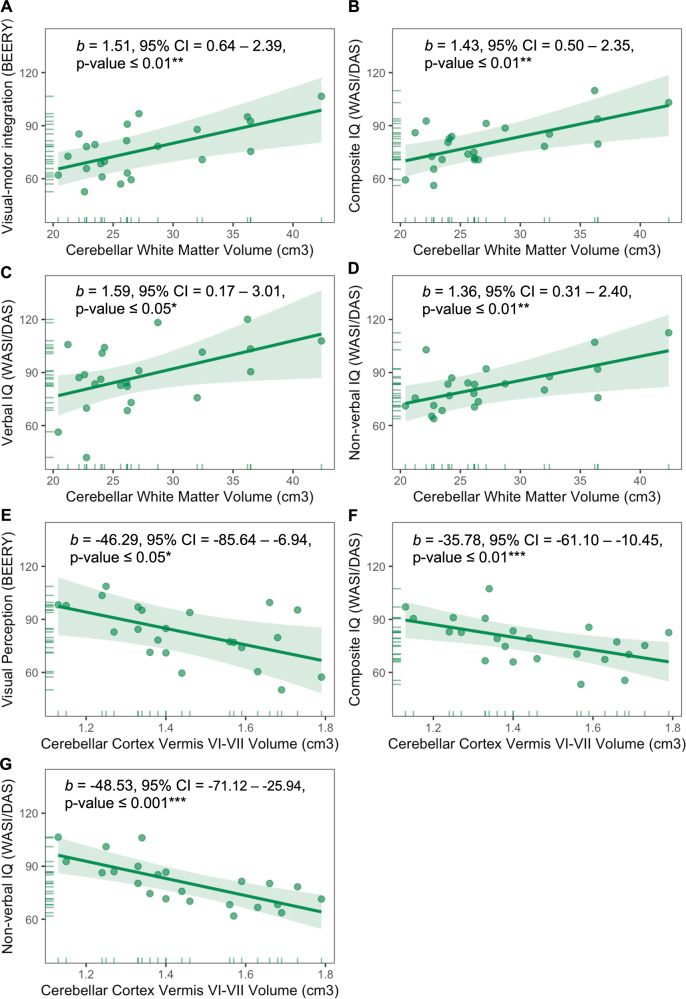
Predictor effect plots showing the relationships between cerebellar white matter and subregional cerebellar cortex volumes and standardized test scores for sensorimotor and cognitive abilities among 3q29Del participants. **A–G** Predicted values for visual-motor integration, visual perception, composite IQ, verbal IQ, and non-verbal IQ scores were computed from the multiple linear regression models reported in Tables S14-16, while covariates (sex, age, eICV) were held fixed. Error bands indicate the 95% confidence interval, data points represent partial residuals, and rug plots show the distribution of the variables. Parameter estimates for the main effect of cerebellar white matter volume and subregional cerebellar cortex volume are indicated on each plot and reflect heteroskedasticity-robust estimates. Regression results indicate significant relationships between cerebellar white matter volume and standardized test scores for (**A**) visual-motor integration skills (*p* ≤ 0.01), (**B**) composite IQ (*p* ≤ 0.01), (**C**) verbal IQ (*p* ≤ 0.05), and (**D**) non-verbal IQ (*p* ≤ 0.01), with larger volumes predicting higher scores among 3q29Del participants. Within cerebellar cortex, significant relationships were identified between vermal lobules VI-VII and standardized test scores for (**E**) visual perception skills (*p* ≤ 0.05), (**F**) composite IQ (*p* ≤ 0.01), and (**G**) non-verbal IQ (*p* ≤ 0.001). 3q29Del *N* = 23. *p* value ≤ 0.001 ‘***’, *p* value ≤ 0.01 ‘**’, *p* value ≤ 0.05 ‘*’, *p* value ≤ 0.1 *‘*^*†*^*’* 3q29Del 3q29 deletion syndrome, IQ intelligence quotient, eICV estimated total intracranial volume, *b* unstandardized coefficient estimate, CI confidence interval.

We next investigated the relevance of these neuroimaging findings to functional deficits in 3q29Del (Fig. S18, Table S13). Both VMI (*b* = 1.38, *p* ≤ 0.01, *FDR-adjusted p* = 0.03) and composite IQ (*b* = 1.33, *p* ≤ 0.01, *FDR-adjusted p* = 0.03) were positively associated with cerebellar WM volumes, while controlling for sex and age (Table S14). In analyses aiming to differentiate sensorimotor and cognitive subprocesses, cerebellar WM volumes were positively associated with both verbal (*b* = 1.51, *p* = 0.03, *FDR-adjusted p* = 0.05) and non-verbal components of IQ (*b* = 1.28, *p* = 0.03, *FDR-adjusted p* = 0.05), but not with visual perception or fine motor coordination (finger and hand movement) skills (*p’s* > 0.05) (Table S14). In confirmatory models that subsequently added eICV as an additional covariate to consider the effects of head size, cerebellar WM volume remained significantly associated with VMI (*b* = 1.51, *p* ≤ 0.01), composite (*b* = 1.43, *p* ≤ 0.01), verbal (*b* = 1.59, *p* = 0.03), and non-verbal IQ (*b* = 1.36, *p* ≤ 0.01) (Table S14, Fig. 5).

We also assessed whether behavioral relationships may have been partially confounded by shared variance (Fig. S19). VMI had a significant positive correlation with composite IQ (*r* = 0.62, *p* ≤ 0.01), which was driven by a significant correlation between VMI and non-verbal IQ (overlapping constructs) (*r* = 0.75, *p* ≤ 0.001). VMI did not correlate with verbal IQ (*p* > 0.05), implying that VMI deficits do not explain the relationship between cerebellar WM volumes and verbal IQ in a significant way, and vice versa.

In tissue-specific analyses, total volume of the cerebellar cortex did not yield significant associations with interrogated behavioral outcomes (*p’s* > 0.05) (Table S14). However, in lobule-specific analyses segmenting the cerebellar cortex into 17 subregions, we found significant associations between subregional gray matter volumes and both sensorimotor and cognitive outcomes (Fig. 5E–G, Tables S15–16). Specifically, an inverse relationship was identified between vermal volumes of lobules VI-VII and visual perception skills among 3q29Del participants, while controlling for age and sex (*b* = −46.35, *p* = 0.03, *FDR-adjusted p* = 0.06) (Tables S15). While this finding did not survive multiple comparisons correction, this effect persisted in confirmatory models where eICV was subsequently added as an additional covariate (*b* = −46.29, *p* = 0.02) (Fig. 5E, Table S15). In addition, a trend level inverse relationship was identified between vermal volumes of lobules VI-VII and visual-motor integration skills; and trend level positive relationships were found between right and left hemispheric volumes of lobule VI and fine motor coordination skills (*p’s* ≤ 0.10) (Table S15); however, these findings did not reach statistical significance.

Furthermore, significant inverse relationships were identified between vermal volumes of lobules VI-VII and both composite IQ (*b* = −35.80, *p* ≤ 0.01, *FDR-adjusted p* = *0.03*) and non-verbal IQ scores (*b* = −48.55, *p* ≤ 0.001, *FDR-adjusted p* ≤ 0.01), while correcting for age and sex (Table S16). These relationships remained persistent in confirmatory models correcting for eICV (composite IQ: *b* = −35.78, *p* ≤ 0.01; non-verbal IQ: *b* = −48.53, *p* ≤ 0.001) (Fig. 5F, G, Table S16), and visual perception skills (composite IQ: *b* = -30.93, *p* = 0.02; non-verbal IQ: *b* = −35.52, *p* ≤ 0.001) (Table S16), accounting for the significant correlations observed between visual perception skills and composite IQ (*r* = 0.43, *p* = 0.04), and visual perception skills and non-verbal IQ (*r* = 0.69, *p* ≤ 0.001) (Fig. S19). In addition, a trend level positive relationship was identified between left Crus II / Lobule VIIB and verbal IQ scores, while correcting for age and sex (*p* ≤ 0.10); however, this finding did not reach statistical significance (Table S16).

Lastly, we found no differences between the standardized test scores of 3q29Del participants with versus without cystic/cyst-like malformations (*p’s* > 0.05) (Table S17), which supports the specificity of behavioral findings to cerebellar changes.

## Discussion

Here we report the first in vivo quantitative neuroimaging study in individuals with 3q29Del, a recurrent CNV that confers exceptionally high genetic risk for neurodevelopmental disability, with effects on multiple functional domains [7, 18, 20, 23–30]. Using high-resolution MRI, standardized behavioral assessment tools, and a hypothesis-driven approach, our study revealed key findings on local alterations of brain structure and brain-behavior relationships in participants with 3q29Del, with a focus on cognitive and sensorimotor outcomes traversing diagnostic categories.

First, by volumetric analysis, we showed that the average size of the cerebellum is significantly smaller among 3q29Del participants compared with neurotypical controls. This difference remained significant after adjustment for eICV, which was itself smaller among 3q29Del participants, indicating a differential effect that was larger in cerebellum than in forebrain. This result corroborates radiological findings from several case reports. Citta et al. (2013) identified cerebellar “atrophy” in a 14-year-old female with 3q29Del, who had a history of ID and psychosis [75]. Sargent et al. (1985) identified “absence” of the cerebellar vermis in a 16-month-old male with a 3q terminal deletion (proximal to 3q29), whose postmortem examination at 26 months also revealed “small” cerebellar hemispheres [76]. Nawa et al. (2022) found atrophy of the cerebellar vermis and hemispheres in a 46-year-old male with 3q29Del presenting with ID, psychosis and autistic features [77]. Hence, our volumetric findings converge with qualitative findings from case reports and for the first time quantitatively highlight the cerebellum as a region of specific pathology in 3q92Del.

For a more nuanced characterization, we next separated the cerebellum into its two primary tissue-types and found that the 3q29Del group had significant reductions in cerebellar cortex volume, which contains more than half of the neurons and well over half the synapses of the entire brain. This finding persisted after eICV-adjustment. Surprisingly, 3q29Del participants also had larger cerebellar WM volumes than controls after eICV-adjustment, implying that cerebellar WM, which mostly contains myelinated axons, exhibits volumetric expansion in 3q29Del relative to what would be expected from controls with the same head size. The volumetric ratio of cerebellar cortex to WM was smaller among 3q29Del participants, confirming that changes in these two structures are nonuniform.

The human cerebellum starts differentiation during the first trimester of gestation, preceding most brain structures [78, 79] and displays a prolonged postnatal developmental course, with cerebellar cortex and WM following distinct trajectories of growth and volumetric peaks at different ages [80–86]. In our study sample, cross-sectional modeling of cerebellar volumes revealed patterns consistent with earlier findings. The typical volume of cerebellar cortex followed an inverted U-shaped trajectory, reflecting a period of rapid growth during early childhood, followed by a slower decline. In contrast, the typical volume of cerebellar WM showed a continuing expansion past adolescence into adulthood. The underlying cellular-level changes that drive these changes in cerebellar cortical volume trajectory are not well defined. A parallel may be found in the neocortex, where developmental changes in gray matter show a transient exuberance in synaptic connections followed by pruning, while changes in white matter involve prolonged increases in myelination and axonal diameter [87]. We conjecture that idiosyncrasies in the rate and type of these neuromaturational processes may account for the trajectory of cerebellar cortex and WM development, and may explain the contrasting volumetric changes observed in these tissues in 3q29Del.

To put forth more localized hypotheses regarding impacted subregions of the cerebellar cortex, we employed a finer-grained, lobule-based segmentation approach with ACAPULCO. Although reductions in absolute volumes of cerebellar regions were widespread in 3q29Del (12 out of 17 regions), adjustment for eICV revealed four specific vulnerable regions, the right and left lobules I-V, representing the anterior lobe (phylogenetically the paleocerebellum), and the right and left lobule VI, representing the superior portion of the posterior lobe (phylogenetically the neocerebellum). Case-control differences in these lobules were also captured in our voxel-level analyses with SUIT-VBM. The emerging pattern suggests the possibility of a susceptibility gradient along the anterior-posterior axis of the cerebellar cortex in individuals with 3q29Del. This may be related to the antero-posterior division documented in ontologic and developmental studies of the cerebellum, which indicate that the origins of distinct cell populations along this axis can be traced to different embryonic brain vesicles [88, 89], and that cerebellar subregions differ in their developmental trajectories, with some findings indicating faster growth in the anterior lobe than most of the posterior lobe during prenatal development [90]. These data, as well as evidence of differences in gene expression and connectivity patterns of the cerebellum across its anterior-posterior axis [91, 92], suggest the possibility that lobules of cerebellar cortex have differential sensitivity to dosage alterations in 3q29 interval genes.

Another key finding was the relationships identified among 3q29Del participants between cerebellar volumes and both sensorimotor and cognitive functions, which exhibit deficits in this population [18, 20] and have been proposed to be pathophysiologically relevant to ID, ADHD, ASD and SZ in a manner more closely related to genetic vulnerability than clinical diagnoses themselves [93–104]. Smaller cerebellar WM volumes were associated with worse VMI (reflecting the ability to integrate sensory information with fine motor commands), and composite, verbal, and non-verbal IQ scores. Importantly, VMI and verbal IQ were not correlated with each other, indicating that these sensorimotor and non-motor associations are not secondary consequences of one another.

Interestingly, although our FreeSurfer-based estimates for total cerebellar cortex volume did not yield significant relationships with our standardized behavioral metrics, lobule-specific analyses with ACAPULCO revealed significant associations between distinct subregions within the cerebellar cortex and both sensorimotor and cognitive skills. Particularly, changes in vermal volumes of lobules VI-VII were related to visual perception skills (reflecting the ability to receive and process visual information from the environment), composite IQ, and non-verbal IQ among 3q29Del participants. Importantly, identified cognitive links persisted after adjustment for eICV and additional control of the variance explained by visual perception scores, indicating that neither the known association between intracranial capacity and cognitive function [105, 106], nor shared variance between the tested behavioral constructs can explain these cognitive correlations.

Posterior vermal lobules VI-VII have long been implicated in visually guided saccadic and smooth pursuit eye movements [107]. Lesions centered on these subregions alter the real-time control and adaptation of various metrics of eye movements in primates [108], which may in part explain the link we observed between these subregions and the capacity to demonstrate age-appropriate visual perception skills in individuals with 3q29Del. Beyond the sensorimotor domain, vermal lobules VI-VII have also been previously linked to performance in a range of cognitive tests in older adults [109]. In experimental mouse work, focal perturbations of vermal lobule VI activity in juvenile life alters the normal development of cognitive functions as well as their adult expression, while gait remains largely unaffected [110]. Acute inhibition of Purkinje cell activity in vermal lobule VI of adult mice impairs cognitive flexibility [111]. Notably, in the mammalian neocortex, lobule VI-VII’s principal targets include not only the somatosensory cortex but also higher-order association regions in the prefrontal and cingulate cortices [110, 112], which are critical for cognitive control. These findings align with significant volumetric associations of these cerebellar subregions with IQ scores in individuals with 3q29Del.

In this context, we highlight that the null finding observed in our analyses of behavioral correlations with total cerebellar cortex volumes likely stems from functional heterogeneity within the cerebellar cortex. Growing evidence indicates the presence of an internal topographical organization within the cerebellum that subserves the multiplicity of behavioral parameters controlled by this hindbrain region [113–115]. It is possible that this diversity may have been similarly masked in prior studies relying on bulk volumetric estimates for the entire cerebellar cortex. We strongly recommend using finer-grained methodological approaches to unveil functional associations in future studies of cerebellar structure.

Cumulatively, our findings indicate that structural cerebellar changes in both WM and subregional gray matter have functional significance for cognitive and sensorimotor phenotypes in 3q29Del. A major shift has begun towards re-conceptualizing the cerebellum as a regulator of both motor and non-motor systems [36–38, 110, 116–124]. The cerebellum and numerous non-motor brain regions are densely connected [110, 112, 116, 125, 126], the cerebellum is specifically activated during cognitive tasks [114, 127, 128], and cerebellar damage produces impairments in linguistic, visuospatial and executive functions [129–132]. A “universal cerebellar transform” theory posits that the cerebellum modulates thought and motor control using the same algorithmic scheme [117]. An alternative “multiple functionality” hypothesis speculates that each functional module of the cerebellum requires a distinct algorithmic scheme [133]; in both cases through bidirectional interactions with the rest of brain, including neocortex.

In recent years, cerebellum has also emerged as a site of renewed interest for a range of neurodevelopmental and psychiatric conditions. Independent studies have shown that structural alterations within the cerebellum are associated with general liability for common psychopathology [134, 135]. Perinatal injury to the cerebellum is now considered the highest known nongenetic risk factor for ASD [37, 136], and a growing number of studies demonstrate that cerebellar growth is particularly susceptible to intra-uterine disruptions such as harmful environmental exposures during pregnancy [137]. Cerebellar abnormalities have also been reported in idiopathic SZ, ASD and ADHD [138], although results in this area show heterogeneity. A recent study found that among patients with psychosis, only individuals with a compromised neuropsychological profile (i.e., low estimated premorbid IQ) exhibit marked cerebellar structural changes, suggesting an early neurodevelopmental link to cerebellar perturbation in a subset of patients with psychosis [139].

Finally, we found that cystic/cyst-like malformations of the posterior fossa show an elevated prevalence among 3q29Del participants. PFAC/MCM prevalence was ~54% among 3q29Del participants, well above the 1% that occurs in controls [34, 35], suggesting a general vulnerability of the posterior fossa to this CNV. Our finding that 3q29Del confers greater influence on risk for these cystic/cyst-like malformations among females confirms our prior identification of reduced male:female bias among participants with 3q29Del in ASD rates [28]. This reduced bias is consistent with the liability threshold model, in which females require a larger genetic burden than males before reaching affection status [140, 141], and the sex ratio in disease prevalence approaches 1:1 as mutation severity increases [142]. 3q29Del is on the more severe end of this genetic spectrum, which may explain why rates of PFAC/MCM are similar among males and females. That said, we found a greater reduction in eICV among male than female 3q29Del participants, consistent with a previous meta-analysis in idiopathic schizophrenia [143]. However, we found no robust sex by diagnostic group interaction effect on cerebellar volumes. These results suggest regional variability in sexual dimorphism patterns in 3q29Del and highlight that sex must be considered an important variable in studies of this CNV. See Supplemental Materials for extended discussion of select findings.

Overall, our results suggest the possibility that 3q29Del sits at the intersection of abnormal cerebellar development and increased risk for neurodevelopmental disability, which converges with evidence from several other genomic variants, including *TSC1*, *FMR1*, *SHANK3*, *MECP2*, *PTEN*, and 22q11.2 deletion, which similarly present with both sensorimotor and cognitive dysfunction [144–150]. The explanatory gap between these loci and disease mechanism has not yet been fully bridged, however altered cerebellar development in early life may be a shared neuroanatomical endophenotype that contributes to disruptions in both sensorimotor and non-motor functions across these variants. Future work should investigate whether the biological mechanisms disrupted in 3q29Del converge onto pathways that are vulnerable to other variants associated with cerebellar abnormalities.

We note that 3q29 genes may have differential effects on cerebellar gray and WM, which if true, would lead to tissue-specific consequences upon their hemizygosity. We queried the Human Protein Atlas (https://www.proteinatlas.org) [151, 152] for the 21 genes located in the 3q29 interval and found that 11 were detected in the cerebellar cortex, four were not, and six were unknown (Fig. S20). Notably, *BDH1*, *DLG1* and *PCYT1A* showed high expression profiles in the granule or Purkinje layers of the cerebellar cortex. *DLG1* and *BDH1* have previously been proposed as drivers of neurodevelopmental phenotypes in this CNV [31, 32]. *DLG1* encodes a synaptic scaffolding protein that interacts with AMPA and NMDA receptors [153], which are key components of glutamatergic synapses and mediators of synaptic plasticity. *BDH1 and PCYT1A* are involved in ketone body metabolism [154] and phosphatidylcholine biosynthesis [155], which are important regulators of bioenergetic homeostasis. Given the high density of excitatory synaptic connections and of neurons in cerebellar cortex, dosage alterations in these genes may be especially detrimental for cerebellar cortex development.

*DLG1* was also detected in cerebellar WM (Fig. S20). In the peripheral nervous system, *DLG1* regulates myelin thickness [156], opening the possibility that 3q29Del may confer a loss in the ability to put a brake on myelination. WM volume can also be affected by axon length and/or axon diameter, and *DLG1* is required for organization of the actin cytoskeleton [157], which plays an important role in axon growth.

Several limitations should be addressed. First, due to its rare prevalence, the 3q29Del sample size was limited, which undermined our ability to reach strong conclusions about interactions involving age and sex. Replication is needed in a larger sample of cases imaged on the same scanner as controls. Large, multi-site studies like the HCP offer an invaluable opportunity to increase statistical power and sensitivity in mapping case-control differences in neuroanatomy; however, variations between scanners can introduce technical confounds, especially when effect sizes are small. Given extensive QC, parallel neuroimaging pipelines, support from radiological data, and previous work showing the resistance of large disease effects to scanner-related artifacts [158], we expect such technical confounds to have produced minimal effects on our findings. We are unable to postulate whether more subtle or distributed deviations in neuroanatomy would be as resistant to scanner effects.

We also note that demands of study participation may have barred individuals with more severe disability from participating; this may have led to underestimation of effect sizes. It is also plausible that cases with milder presentations that did not prompt genetic testing went missed. Analyses should be expanded in future work to psychiatric phenotypes, and finer-grained measures of cognitive and sensorimotor performance for further insights. Importantly, cerebellar connections and volumetric co-variance with cortical and subcortical structures warrant investigation to expand questions to the circuit-level. Finally, future studies will benefit from testing alternative ICV estimation methods, as well as whole-brain VBM.

Finally, cerebellar defects may have impacts on distant brain regions. The “developmental diaschisis” theory argues that early-life cerebellar perturbations can hinder the development of other brain regions [37]. Identifying the origin of pathology in 3q29Del will require the inclusion of earlier timepoints and longitudinal tracking, and the mouse model of 3q29Del [159] may help elucidate these mechanisms.

## Supplementary information

Supplemental Materials

## Data Availability

The 3q29Del data collected from NIH grants R01MH110701 and R01MH118534 are available from the NIMH Data Archive (NDA). NDA is a collaborative informatics system created by the NIH to provide a national resource to support and accelerate research in mental health. Dataset identifiers: 10.15154/kc4f-3v32. The code used for all analyses is available from the corresponding authors on reasonable request. Access to the HCP data is subject to the WU-Minn HCP Consortium’s Open Access and Restricted Data Use Terms for the HCP-Young Adult dataset (1200 subjects data release; structural data available from the Connectome Coordination Facility via: https://humanconnectome.org/study/hcp-young-adult/document/1200-subjects-data-release) and the NDA’s Data Use Certification Agreement for the HCP-Development dataset (HCP-Development Lifespan 2.0 release; structural data available from the NDA via: https://nda.nih.gov/general-query.html?q=query=featured-datasets:HCP%20Aging%20and%20Development). The manuscript reflects the views of the authors and may not reflect the opinions or views of the NIH.
