# Supplementary material for: Structural deviations of the posterior fossa and the cerebellum and their cognitive links in a neurodevelopmental deletion syndrome
Source: Mol Psychiatry. 2024 May 14;29(11):3395–411. doi: 10.1038/s41380-024-02584-8 (PMC11541222; doi:10.1038/s41380-024-02584-8)
Supplement: Supplementary file 1 — Supplemental Materials [file 41380_2024_2584_MOESM1_ESM.pdf]

Supplemental materials

Structural deviations of the posterior fossa and the cerebellum and their cognitive links in a neurodevelopmental deletion syndrome

Table of contents

Supplemental figures and tables……………………………………………………………………………3-74

Table S1. Demographic characteristics of the study sample included in tissue-specific volumetric analyses, stratified by diagnostic group and sex………………………………………………………………………3
Fig. S1. Percent of 3q29Del participants who qualify for neurodevelopmental and psychiatric diagnoses after direct evaluation by our team ……………………………………………………………………………4
Table S2. Structural magnetic resonance imaging (MRI) and data processing protocols…………………5-6
Fig. S2. Example cerebellar segmentation masks of representative age- and sex-matched 3q29Del and neurotypical control pairs included in tissue-specific volumetric analyses……………………………7-8
Fig. S3. Example subregional cerebellar cortex masks derived with ACAPULCO for representative age- and sex-matched 3q29Del and neurotypical control pairs…………………………………………9-10
Fig. S4. Relationship between estimated total intracranial volume, total brain volume and head circumference among 3q29Del participants ……………………………………………………………11
Fig. S5. Relationships between eICV and A) total cerebellum, B) cerebellar cortex, and C) cerebellar white matter volumes among 3q29Del participants versus controls……………………………………………12
Fig. S6. Correction method for adjustment of cerebellar volumes for head size variation in case-control comparisons……………………………………………………………………………………………13
Fig. S7. Scatter plots showing the distribution of eICV-adjusted A) total cerebellum volume, B) cerebellar cortex volume, and C) cerebellar white matter volume as a function of age among male and female participants in each diagnostic group……………………………………………………………………14
Table S3. Extended multiple linear regression results testing the effects of diagnostic group on tissue-specific cerebellar volumes and eICV, with polynomial modeling of age…………………………15-22
Fig. S8. Regression diagnostics: testing the assumptions of ordinary least squares regression for best-fitting models from Table S3…………………………………………………………………………23-24
Table S4. Sensitivity analysis: multiple linear regression results testing the effect of diagnostic group on tissue-specific cerebellar volumes and eICV, after removal of an outlier datapoint from the 3q29Del group……………………………………………………………………………………………………25-26
Table S5. Summary of supplemental results from penalized cubic spline models testing the effect of diagnostic group on tissue-specific cerebellar volumes and eICV ……………………………………27
Fig. S9. Developmental trajectories for tissue-specific cerebellar volumes and eICV, estimated by the penalized cubic spline approach…………………………………………………………………………28
Table S6. Supplemental age- and sex-matched analyses testing for diagnostic group differences in tissue-specific cerebellar volumes and eICV ……………………………………………………………………29
Table S7. Exploratory modeling of diagnostic group by sex interaction effects on tissue-specific cerebellar volumes and eICV……………………………………………………………………………………30-32
Fig. S10. Predictor effect plots visualizing results from sex by diagnostic group interaction models……………………………………………………………………………………………………33
Table S8. *Post hoc* analysis of sex by diagnostic group interaction effects on eICV and eICV-adjusted cerebellar white matter volumes…………………………………………………………………………34
Table S9. Exploratory modeling of diagnostic group by age interaction effects on tissue-specific cerebellar volumes and eICV……………………………………………………………………………………35-37
Fig. S11. Predictor effect plots showing suggestive interaction effects between diagnostic group and age on absolute and eICV-adjusted cerebellar white matter volumes…………………………………………38
Fig. S12. Estimated normative percentile curves for tissue-specific cerebellar volumes and eICV, stratified by sex……………………………………………………………………………………………………39-41

Fig. S13. Distribution of ACAPULCO-based subregional cerebellar cortex volumes across age- and sex-matched 3q29Del and neurotypical control participants……………………………..………………………..42
Table S10. Age- and sex-matched analyses testing for diagnostic group differences in lobule-specific cerebellar cortex volumes……………………………………………………………………………………….43-45
Fig. S14. SUIT-VBM analyses for voxel-level case-control differences in absolute cerebellar gray matter volumes.…………..………………………………………………………………………………………….46
Fig. S15. SUIT-VBM analyses for voxel-level case-control differences in eICV-adjusted cerebellar gray matter volumes.……………..……………………………………………………………………………..….47-48
Fig. S16. Flat maps of voxel-level case-control differences in cerebellar gray matter volume identified through SUIT-VBM analyses in age- and sex-matched 3q29Del and neurotypical control participants………………………………………………………………………………………………….....49
Table S11. Demographic and relevant clinical characteristics of 3q29Del participants with versus without posterior fossa arachnoid cyst or mega cisterna magna findings……………………….………………....50
Fig. S17. Scatter plots showing the distribution of A) total cerebellum volume, B) cerebellar cortex volume, C) cerebellar white matter volume, and D) eICV as a function of age among 3q29Del participants with versus without posterior fossa arachnoid cyst or mega cisterna magna findings…………………...…..51
Table S12. Exploratory analysis of the relationship between posterior fossa arachnoid cyst and mega cisterna magna findings and tissue-specific cerebellar volumes and eICV among 3q29Del participants……………………………………………………………………………………………...52-53
Fig. S18. Violin plots with box plots visualizing the distribution of standardized test scores for sensorimotor and cognitive abilities among 3q29Del participants ………………..……………………………………54
Table. S13. Descriptive statistics for standardized test scores for sensorimotor and cognitive abilities among 3q29Del participants……..…………………………………..………..………………………54
Table S14. Extended multiple linear regression results testing the relationships between tissue-specific cerebellar volumes and sensorimotor and cognitive abilities among 3q29Del participants ………...…55-57
Table S15. Multiple linear regression results testing the relationships between ACAPULCO-based subregional cerebellar cortex volumes and sensorimotor abilities among 3q29Del participants……....58-64
Table S16. Multiple linear regression results testing the relationships between ACAPULCO-based subregional cerebellar cortex volumes and cognitive abilities among 3q29Del participants…………...65-71
Fig. S19. Heatmap visualization of pairwise Pearson's correlations between standardized test scores for sensorimotor and cognitive abilities among 3q29Del participants ……………………………………………72
Table S17. Standardized test scores for sensorimotor and cognitive abilities in 3q29Del participants with versus without posterior fossa arachnoid cyst or mega cisterna magna findings ……………….……...…73
Fig. S20. Tissue and cell-type specific protein expression profiles of 3q29 interval genes annotated by the Human Protein Atlas……………….……………………..……………………………….………..……74

Supplemental methods and extended discussion……………………………………………………………75-86

Extended methods for processing and quality control of structural MRI data…………………………....75-76
Extended methods for radiological evaluation of structural MRI data……………………………...…..76-77
Details on standardized behavioral measures…………………………………………………………........77
Extended statistical methods for penalized cubic spline and quantile spline models……..…………..…77-81
List of R packages used for statistical analyses and diagnostics…………………………………….....81-82
Extended discussion of cerebellar growth trajectories…………………………………………………82-83
Extended discussion of subregional findings with ACAPULCO…………………………………….....83-84
References for supplemental methods and extended discussion…..…………………………..…....84-86

Supplemental figures and tables

Demographic variables	Males			Females		
	**Control** *N* = 747	**3q29Del** *N* = 14	**Test statistics**	**Control** *N* = 861	**3q29Del** *N* = 9	**Test statistics**
**Age** (in years)						
Mean ± SD	22.38 ± 7.74	14.14 ± 9.03	*r* = 0.13 (small effect size), *W* = 8146.5, p-value [a] = 3.40E-04***	23.03 ± 8.60	16.56 ± 9.86	*r* = 0.07 (small effect size), *W* = 5422, p-value [a] = 0.04*
Median	24.00	13.00		25.00	15.00	
[Range]	[5 – 37]	[4 – 39]		[6 – 36]	[6 – 34]	
**Ethnicity**[#], *N (%)*						
Non-Hispanic / Latino	64 (86.60%)	13 (92.86%)	X^2 = 0.08, DF = 1, p-value [b] = 0.78	757 (98.31%)	9 (100%)	X^2 = 0.25, DF = 1, p-value [b] = 0.62
Hispanic / Latino	99 (13.40%)	1 (7.14%)		91 (11.82%)	0 (0%)	
**Race**[##], *N (%)*						
White	52 (71.53%)	13 (92.86%)	X^2 = 3.83, DF = 4, p-value [b] = 0.43	587 (69.88%)	8 (88.89%)	X^2 = 2.43, DF = 4, p-value [b] = 0.66
Black / African American	97 (13.21%)	0 (0%)		125 (14.88%)	0 (0%)	
Asian / Native Hawaiian / Other Pacific Islander	55 (7.49%)	0 (0%)		53 (6.31%)	0 (0%)	
American Indian / Alaskan Native	3 (0.41%)	0 (0%)		1 (0.12%)	0 (0%)	
More than one race	54 (7.36%)	1 (7.14%)		74 (8.81%)	1 (11.11%)	

Table S1. Demographic characteristics of the study sample included in tissue-specific volumetric analyses, stratified by diagnostic group and sex. To supplement the models that explore the sex-specific effects of 3q29Del on tissue-specific VOIs derived with FreeSurfer, we report here the demographic characteristics of the study sample included in corresponding analyses, stratified by diagnostic group and sex. While there was a near complete overlap between the age ranges of the two diagnostic groups in each sex, there was a significant age difference between male 3q29Del participants and male controls ($p \leq 0.001$), and female 3q29Del participants and female controls on average ($p \leq 0.05$). There were no significant differences in the ethnicity or race compositions of the two diagnostic groups in either sex (*p's* > 0.05). Effect sizes are reported for significant test results only. Non-parametric statistics are reported in cases where the data do not meet parametric assumptions. [#]Male control *N* = 739, Female control *N* = 848 for the ethnicity variable due to missing data. [##]Male control *N* = 734, Female control *N* = 840 for the race variable due to missing data. Corresponding percentages reflect the fraction of male or female controls with complete data. [a]Wilcoxon rank sum test, [b]Pearson's chi-squared test. p-value ≤ 0.001 '***', p-value ≤ 0.01 '**', p-value ≤ 0.05 '*', p-value ≤ 0.1 '†' *Abbreviations*: 3q29 deletion syndrome, 3q29Del; volumetric measure of interest, VOI; standard deviation, SD; degrees of freedom, DF.

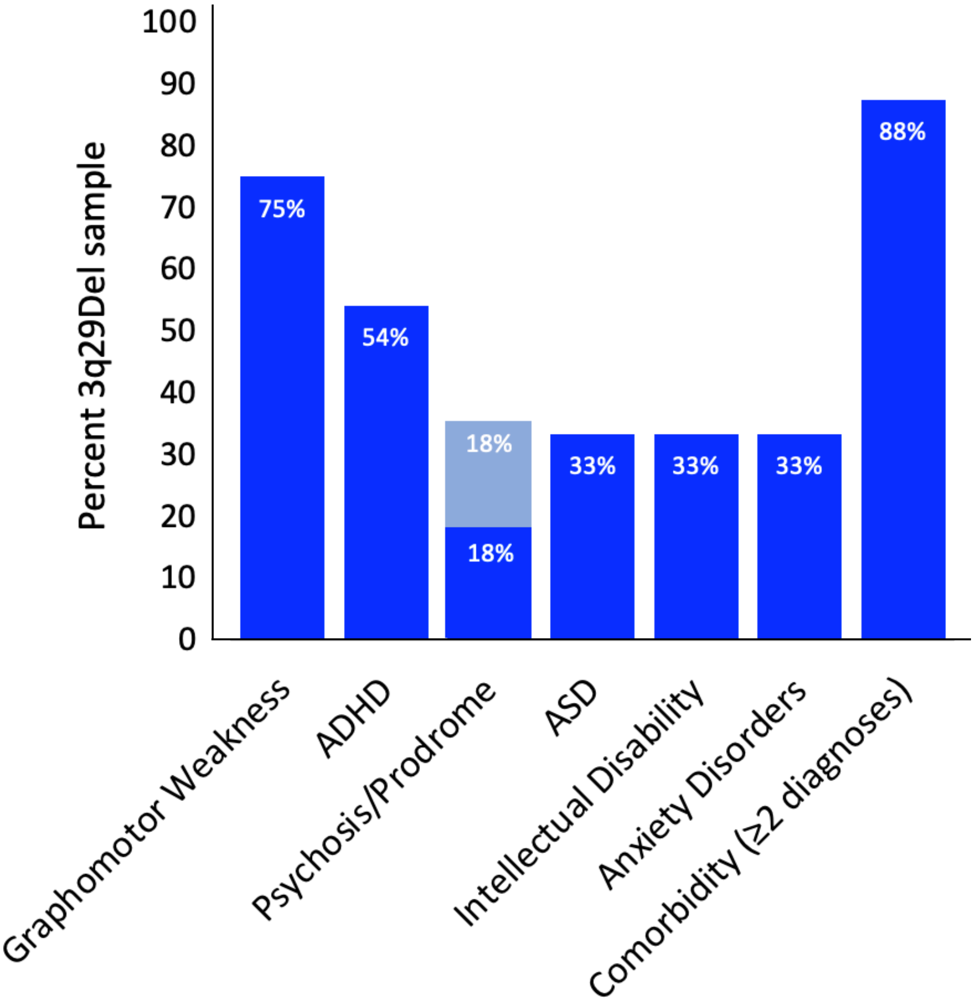

Fig. S1. Percent of 3q29Del participants who qualify for neurodevelopmental and psychiatric diagnoses after direct evaluation by our team. Out of *N* = 24 3q29Del participants included in this study, 18 (75%) qualified for a diagnosis of clinically significant graphomotor weakness, 13 (54%) qualified for ADHD, 8 (33%) qualified for intellectual disability, 8 (33%) qualified for ASD, and 8 (33%) qualified for an anxiety disorder. Psychosis and psychosis prodrome were evaluated only among 3q29Del participants aged 8 years and older, as developmentally appropriate magical thinking cannot be distinguished from true psychosis at younger ages. Out of *N* = 17 3q29Del participants evaluated for these two clinical phenotypes, 3 (18%) qualified for a psychotic disorder diagnosis (1 schizophrenia; 1 schizoaffective disorder, bipolar type; 1 unspecified schizophrenia spectrum and other psychotic disorder), and 3 additional participants (18%) were found to exhibit signs and symptoms consistent with the psychosis prodrome (light blue). Out of *N* = 24 3q29Del participants, 21 (88%) met criteria for two or more of these seven major clinical diagnoses, indicating a substantial burden of neurodevelopmental and psychiatric comorbidities. See Sanchez-Russo et al. (2020) for a comprehensive description of the clinical phenotypes evaluated in the larger 3q29Del sample from which the current 3q29Del neuroimaging sample was drawn, and for the list of standardized diagnostic tools used for these evaluations. *Abbreviations*: 3q29 deletion syndrome, 3q29Del; autism spectrum disorder, ASD; attention-deficit/hyperactivity disorder, ADHD.

Sanchez Russo R, Gambello MJ, Murphy MM, Aberizk K, Black E, Burrell TL, et al. (2021): Deep phenotyping in 3q29 deletion syndrome: recommendations for clinical care. *Genet Med.* 23(5):872-80.

	3q29Del<br>*N* = 24	HCP Young Adult<br>*N* = 1,113	HCP Development<br>*N* = 652
**MRI Hardware**			
Scanner	Siemens Magnetom Prisma 3T	Siemens Magnetom Skyra 3T (Customized 3T "Conectom")	Siemens Prisma 3T
Max gradient strength	80 mT/m gradient coil	100 mT/m gradient coil	80 mT/m gradient coil
Head coil	Siemens 32-channel Prisma head coil	Siemens 32-channel standard head coil	Siemens 32-channel Prisma head coil
**Acquisition parameters | T1- & T2-weighted structurals**			
Slice thickness (mm)	0.8	0.7	0.8
vNavs for prospective motion correction	NO	NO	YES
FOV read (mm)	256	224	256
FOV phase (%)	93.8	100	93.8
Base resolution	320	320	320
Slabs	1	1	1
Slices per slab	208	256	208
Slice orientation	Sagittal	Sagittal	Sagittal
PAT mode	GRAPPA	GRAPPA	GRAPPA
Acceleration Factor PE	2	2	2
Reference lines PE	32	32	32
**Acquisition parameters | T1-weighted structural**			
Pulse sequence	3D single-echo T1-weighted MPRAGE	3D single-echo T1-weighted MPRAGE	3D multi-echo T1-weighted MPRAGE
TR (ms)	2400	2400	2500
TE (ms)	2.2	2.1	1.8 / 3.6 / 5.4 / 7.2
TI (ms)	1000	1000	1000
Flip angle (degree)	8	8	8
Coil elements	HEA; HEP	HEA; HEP	HEA; HEP
Bandwidth (Hz/Px)	220	210	744 / 744 / 744 / 744
Fat suppression	Water excitation fast	Water excitation fast	Water excitation fast
Scan time (min:sec)	6:38	7:40	8:22
**Acquisition parameters | T2-weighted structural**			
Pulse sequence	3D T2-weighted SPACE	3D T2-weighted SPACE	3D T2-weighted SPACE
TR (ms)	3200	3200	3200
TE (ms)	563	565	564
Flip angle mode	variable	variable	variable
Coil elements	HC1-7; NC1,2	HEA; HEP	HEA; HEP
Bandwidth (Hz/Px)	744	744	744
Turbo factor	314	314	314
Fat suppression	None	None	None
Scan time (min:sec)	6:38	7:40	8:22
**Other**			
Data release version	N/A	"1200 subjects data release" - minimally processed format	"HCP-Development Lifespan 2.0 release" - minimally processed format
Structural processing pipeline for tissue-specific segmentations	HCP "minimal pre-processing" pipeline (v4.1.3 with FreeSurfer v6.0) (Glasser et al., 2013, Harms et al., 2018)	HCP "minimal pre-processing" pipeline (v3.21 with FreeSurfer v5.3.0-HCP) (Glasser et al., 2013, Harms et al., 2018)	HCP "minimal pre-processing" pipeline (v4.3.0 with FreeSurfer v6.0) (Glasser et al., 2013, Harms et al., 2018)
Manual editing following QC of tissue-specific segmentations	NO	NO	NO
Structural processing pipeline for lobule-specific analyses	ACAPULCO (v.3.0) (Han et al., 2020)	ACAPULCO (v.3.0) (Han et al., 2020). Applied to the age- and sex-matched subsample.	ACAPULCO (v.3.0) (Han et al., 2020). Applied to the age- and sex-matched subsample.
Structural processing pipeline for voxel-level analyses	SUIT-VBM (v.3.4) (Diedrichsen, 2006; Diedrichsen et al., 2009)	SUIT-VBM (v.3.4) (Diedrichsen, 2006; Diedrichsen et al., 2009) Applied to the age- and sex-matched subsample.	SUIT-VBM. (v.3.4) (Diedrichsen, 2006; Diedrichsen et al., 2009) Applied to the age- and sex-matched subsample.
Manual editing following QC of subregional segmentations	YES	YES	YES

Table S2. Structural magnetic resonance imaging (MRI) and data processing protocols. A detailed report of the imaging protocols and relevant parameters used in the 3q29Del, HCP Young Adult and HCP Development datasets are provided in this table for increased transparency. HCP Young Adult and Development datasets were pooled to derive the control dataset; their corresponding imaging protocols were previously shown to be largely congruent, with most differences rooted in challenges related to scanning developmental populations (Harms et al., 2018). Note that the original "1200 Subjects Release" by the HCP Young Adult project includes structural MRI scans for *N* = 1,113 participants. A subset of these data comes from monozygotic twins, who have been previously reported to exhibit high to moderate correlations in brain morphology (e.g., White et al., 2002). To minimize bias in our standard error estimates, one sibling from each known monozygotic twin-pair that was scanned by the HCP Young Adult project was removed from the original dataset in the present study (*N* = 154 monozygotic twin-pairs based on available genotyping and/or self-report data**;** in cases of discrepancy between genotyping and self-report data, we relied on genetically verified data for final filtering). We additionally removed all participants with unknown zygosity information *(N* = 3) from the HCP Young Adult dataset for stringency. Hence, the final sample size of the HCP Young Adult data included in the present study for tissue-specific analyses was *N* = 956. Note that there were no monozygotic twin-pairs in the 3q29Del dataset. Zygosity information was not publicly available for the HCP Development dataset. Importable imaging protocols for all HCP datasets are available at https://www.humanconnectome.org/hcp-protocols. For SUIT-VBM-based voxel-level analyses and ACAPULCO-based lobule-specific analyses parcellating the cerebellar cortex into smaller subregions, only data from the subset of control participants who were age- and sex-matched to 3q29Del participants with a 1:4 case-control ratio were used. *Abbreviations*: 3q29 deletion syndrome, 3q29Del; Human Connectome Project, HCP; magnetization-prepared rapid gradient-echo, MPRAGE; Sampling perfection with application optimized contrast using different angle evolutions, SPACE; repetition time, TR; echo time, TE; inversion time, TI; volumetric navigators, vNavs; Field-of-view, FOV; parallel acquisition technique, PAT; phase-encoding, PE; generalized auto-calibrating partial parallel acquisition, GRAPPA; quality control, QC; ACAPULCO, Automatic Cerebellum Anatomical Parcellation using U-Net Locally Constrained Optimization; SUIT, Spatially Unbiased Infratentorial Template; VBM, voxel-based morphometry.

Diedrichsen J (2006): A spatially unbiased atlas template of the human cerebellum. *Neuroimage.* 33(1):127-38.

Diedrichsen J, Balsters JH, Flavell J, Cussans E, Ramnani N (2009): A probabilistic MR atlas of the human cerebellum. *Neuroimage*. 46(1):39-46.

Glasser MF, Sotiropoulos SN, Wilson JA, Coalson TS, Fischl B, Andersson JL, et al. (2013): The minimal preprocessing pipelines for the Human Connectome Project. *Neuroimage*. 80:105-124.

Harms MP, Somerville LH, Ances BM, Andersson J, Barch DM, Bastiani M, et al. (2018): Extending the Human Connectome Project across ages: Imaging protocols for the Lifespan Development and Aging projects. *Neuroimage*. 183:972-984.

Han S, Carass A, He Y, & Prince JL (2020): Automatic cerebellum anatomical parcellation using U-Net with locally constrained optimization. *NeuroImage*. 218:116819.

White T, Andreasen NC, Nopoulos P (2002): Brain volumes and surface morphology in monozygotic twins. *Cereb Cortex*. 12:486-493.

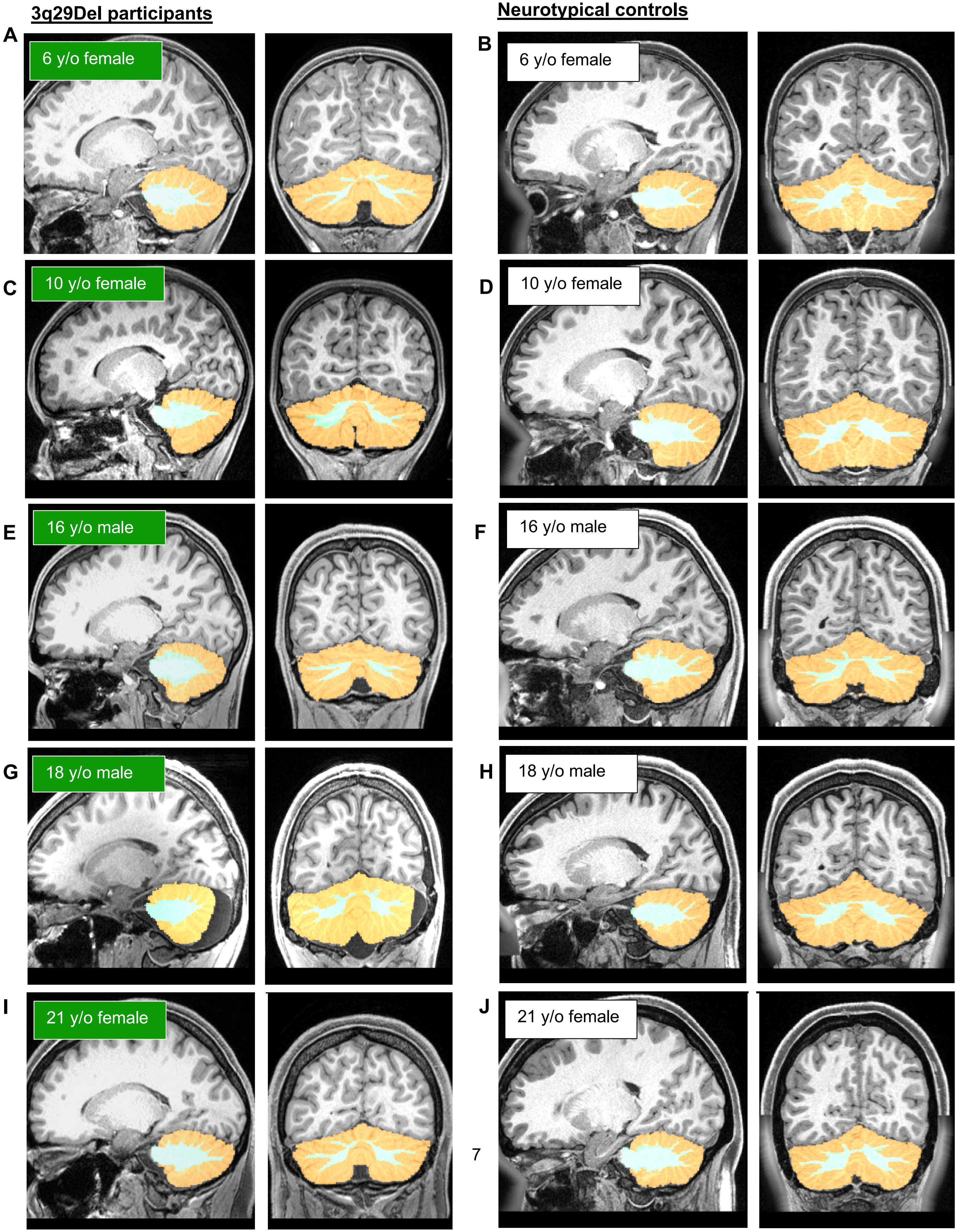
3q29Del participants
Neurotypical controls
A
6 y/o female
B
6 y/o female
C
10 y/o female
D
10 y/o female
E
16 y/o male
F
16 y/o male
G
18 y/o male
H
18 y/o male
I
21 y/o female
J
21 y/o female

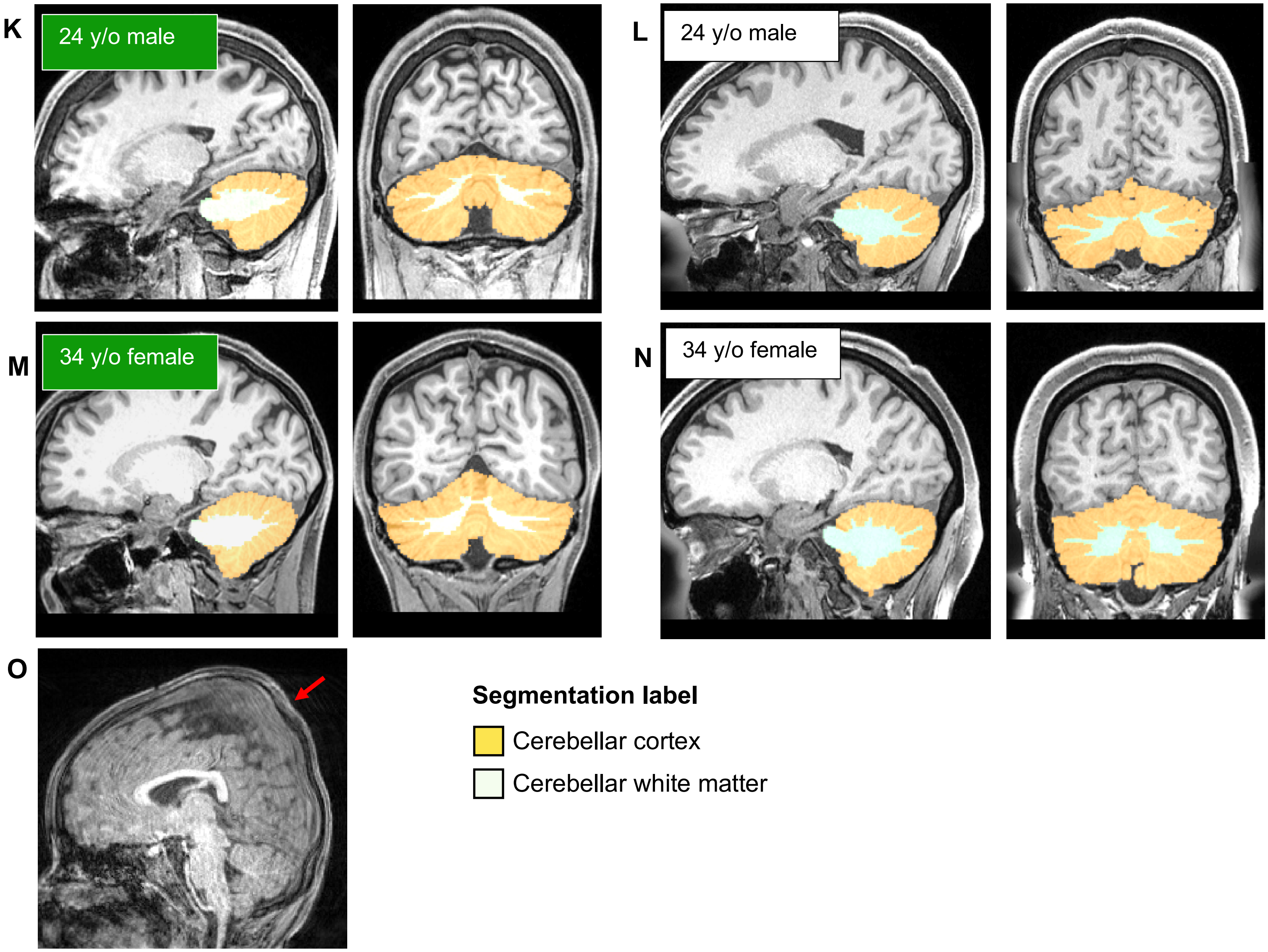

Fig. S2. Example cerebellar segmentation masks of representative age- and sex-matched 3q29Del and neurotypical control pairs included in tissue-specific volumetric analyses. A-N) For quality control, cerebellar cortex and white matter segmentation masks were overlaid on T1-weighted MR images acquired from *N* = 23 age- and sex-matched case-control pairs randomly selected from the dataset. Representative images for seven randomly selected case-control pairs are provided in this figure for illustration in sagittal and coronal planes. 3D volumes were segmented using FreeSurfer's automated subcortical segmentation algorithm in both diagnostic groups, as implemented in the HCP "minimal pre-processing" pipeline (Glasser et al., 2013; Harms et al., 2018). Image quality, tissue contrast and the boundaries of delineation for cerebellar voxel classifications were examined by two independent evaluators using the Connectome Workbench tool (https://www.humanconnectome.org/software/get-connectome-workbench). These outputs were found to be highly consistent within and between diagnostic groups, independent of age, sex, and presence or absence of radiologically observable posterior fossa abnormalities. **O)** A representative T1-weighted sagittal MR image of a 6-year-old male 3q29Del participant who was excluded from volumetric analyses due to severe motion artifacts and a skull deformity interfering with reliable volume estimations. Upon radiological examination, this 3q29Del participant was found to have plagiocephaly (red arrow). An example MR image is provided to illustrate our basis for exclusion. *Abbreviations*: 3q29 deletion syndrome, 3q29Del; years old, y/o.

Harms MP, Somerville LH, Ances BM, Andersson J, Barch DM, Bastiani M, et al. (2018): Extending the Human Connectome Project across ages: Imaging protocols for the Lifespan Development and Aging projects. *Neuroimage*. 183:972-984.

Glasser MF, Sotiropoulos SN, Wilson JA, Coalson TS, Fischl B, Andersson JL, et al. (2013): The minimal preprocessing pipelines for the Human Connectome Project. *Neuroimage*. 80:105-124

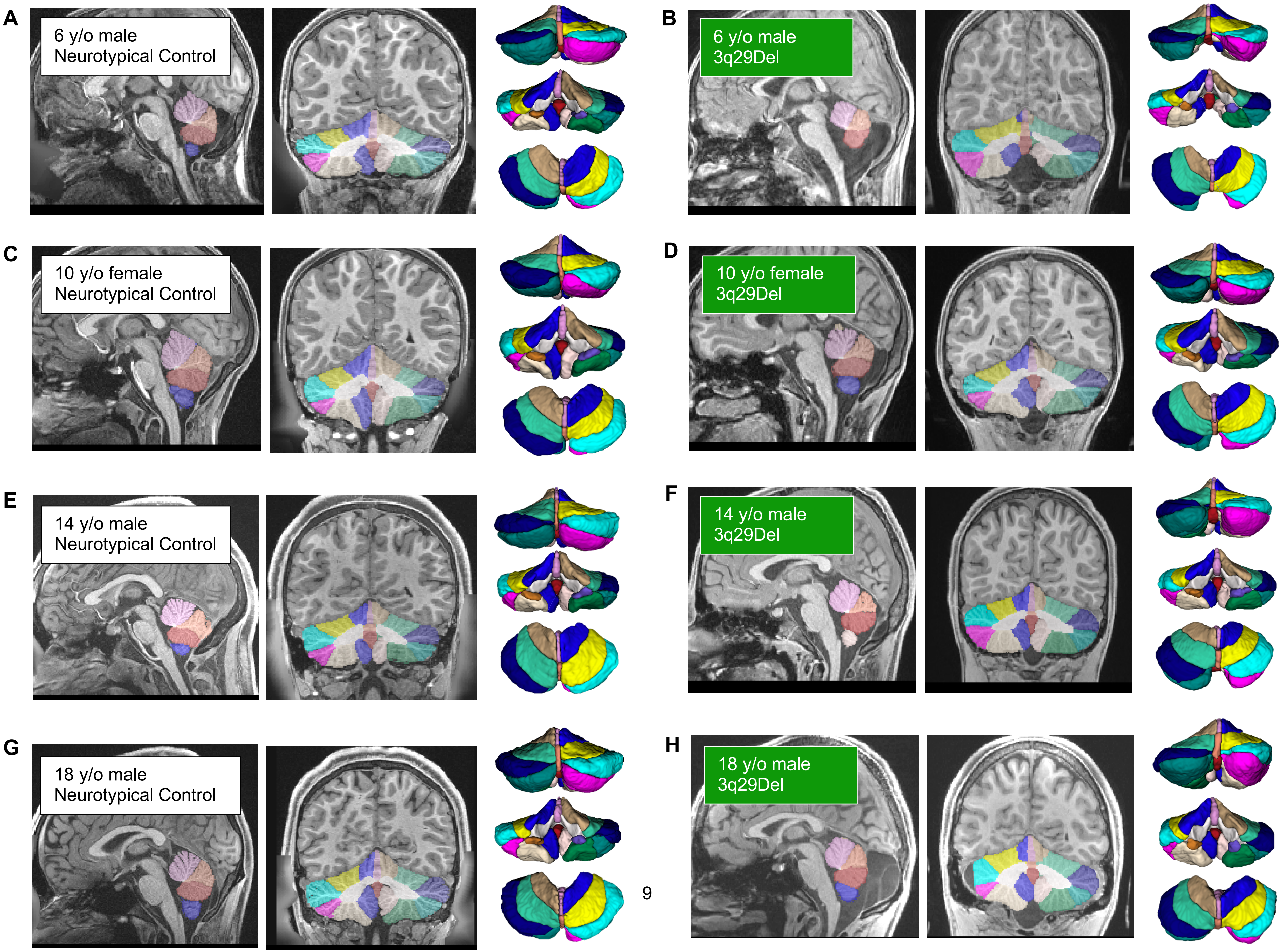
A
6 y/o male
Neurotypical Control
B
6 y/o male
3q29Del
C
10 y/o female
Neurotypical Control
D
10 y/o female
3q29Del
E
14 y/o male
Neurotypical Control
F
14 y/o male
3q29Del
G
18 y/o male
Neurotypical Control
H
18 y/o male
3q29Del

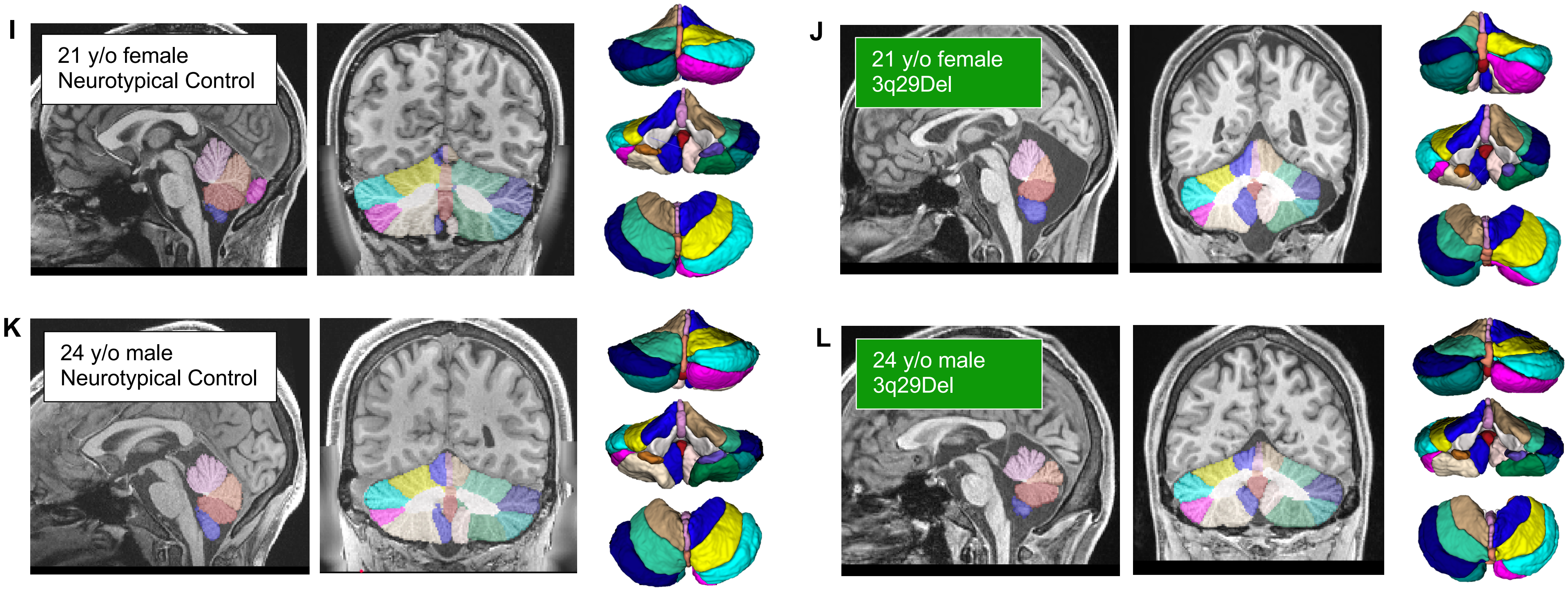

Fig. S3. Example subregional cerebellar cortex masks derived with ACAPULCO for representative age- and sex-matched 3q29Del and neurotypical control pairs. A-L) Representative T1-weighted images for six case-control pairs randomly selected from the age- and sex-matched study sample, with ACAPULCO-based subregional cerebellar cortex parcellations overlaid in sagittal and coronal planes post-QC. 3D reconstructions of resulting volumes viewed from three different angles are provided next to each scan. The segmentation labels for 17 suregions that the cerebellar cortex was divided into are color coded as shown in the legend (14 labels for lateral hemispheres and 3 labels for midline vermis). For quality control, the boundaries of all subregional classifications were examined by two trained evaluators. Mis-parcellations, including errors in the delineation of fissures separating adjacent lobules, over-segmentation of extra-cerebellar tissue, and under-segmentation of cerebellar tissue, were manually corrected slice-by-slice using ITK-SNAP (v. 4.0.1; http://www.itksnap.org). Note that in coronal view, the right side of the cerebellum appears on the left side of the image. *Abbreviations*: Automatic Cerebellum Anatomical Parcellation using U-Net Locally Constrained Optimization, ACAPULCO; 3q29 deletion syndrome, 3q29Del; years old, y/o; right hemisphere, RH; left hemisphere, LH.

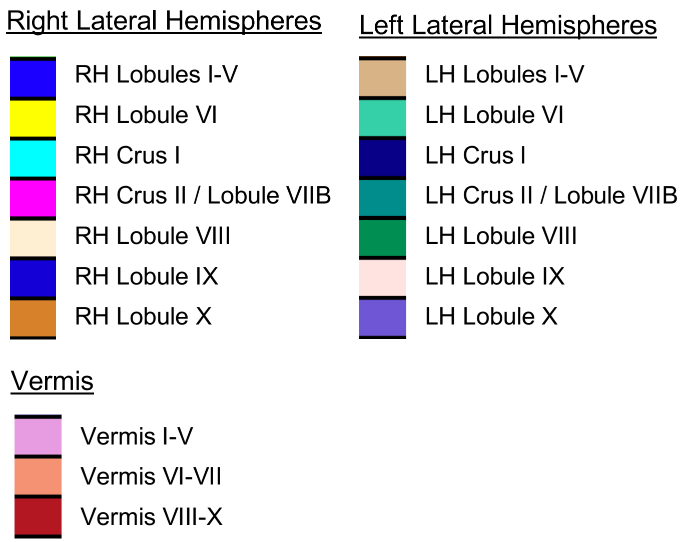

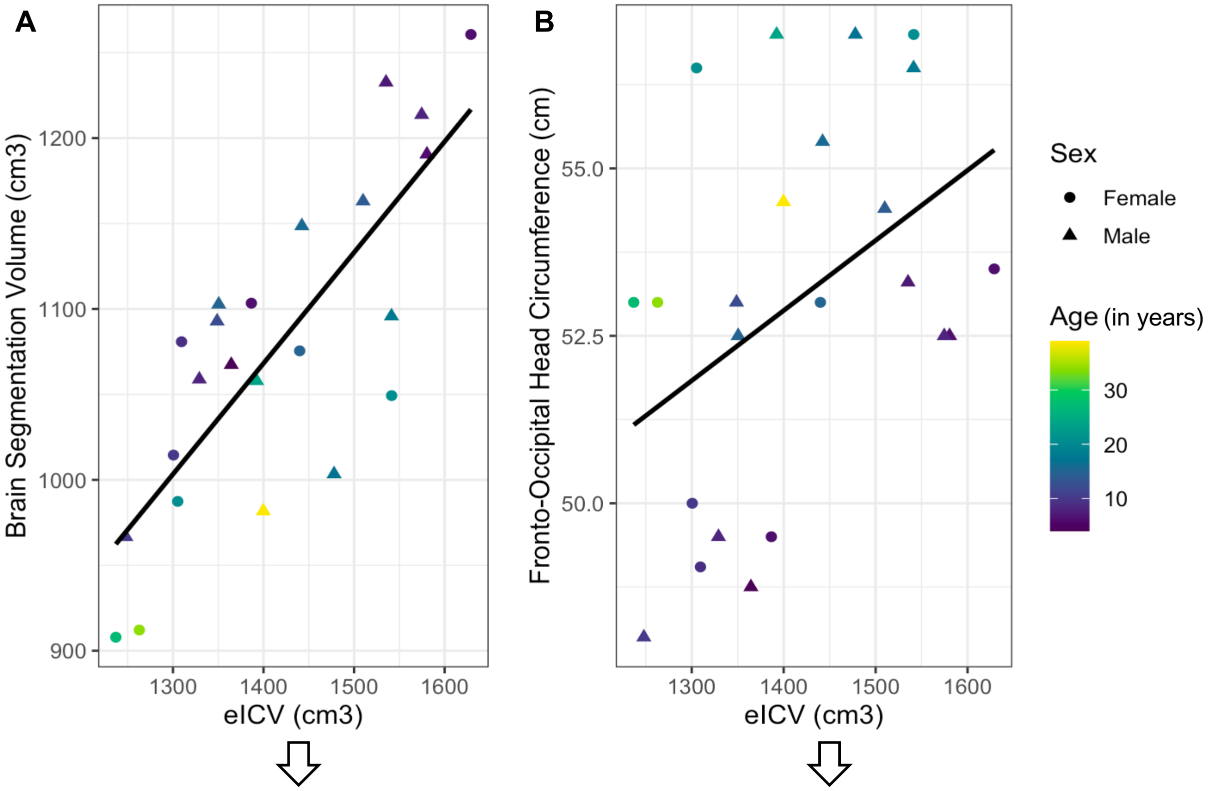

Pearson's *r* = 0.79
p-value = 6.50E-06***

Partial correlation after controlling for age and sex:

Pearson's *r* = 0.82
p-value = 6.55E-06***

Pearson's *r* = 0.43
p-value = 0.04*

Partial correlation after controlling for age and sex:

Pearson's *r* = 0.71
p-value = 2.92E-04***

Fig. S4. Relationships between estimated total intracranial volume, total brain volume and head circumference among 3q29Del participants. Morphometric analysis of regional brain structure confronts the challenge of accounting for head size variation. A widely used automated procedure for head size correction, which has been validated against manually delineated measurements of total intracranial volume (ICV), is the atlas-based head size normalization technique developed by FreeSurfer. This method exploits the relationship between ICV and the linear transform to MNI space to calculate an estimated total intracranial volume (eICV) for each participant, as described in Buckner et al. (2004). Since direct quality control of ICV segmentations is not attainable in this framework (due to lack of available segmentation masks), we assessed the quality of our eICV data by testing the Pearson's correlations between **A)** eICV and total brain volume (i.e., the "brain segmentation volume" label in FreeSurfer: volume of all voxels that are not background / brain stem) and **B)** eICV and fronto-occipital head circumference, which was determined by our team using standardized tape measurement in $N = 23$ 3q29Del participants. Consistent with previous literature (Hshieh et al., 2016; Wolf et al., 2003 and others), these variables showed a significant positive correlation with eICV in our 3q29Del dataset, with moderate to strong correlation coefficients (p-values ≤ 0.05), providing an indirect metric for quality control. Note that visual inspection of the alignments from which eICV values were calculated did not reveal any errors. *Abbreviations*: 3q29 deletion syndrome, 3q29Del.

Buckner RL, Head D, Parker J, Fotenos AF, Marcus D, Morris JC, et al. (2004): A unified approach for morphometric and functional data analysis in young, old, and demented adults using automated atlas-based head size normalization: reliability and validation against manual measurement of total intracranial volume. *Neuroimage*. 23:724-738.

Hshieh TT, Fox ML, Kosar CM, Cavallari M, Guttmann CR, Alsop D, et al. (2016): Head circumference as a useful surrogate for intracranial volume in older adults. *Int Psychogeriatr*. 28:157-162.

Wolf H, Kruggel F, Hensel A, Wahlund LO, Arendt T, Gertz HJ (2003): The relationship between head size and intracranial volume in elderly subjects. *Brain Res*. 973:74-80.

A

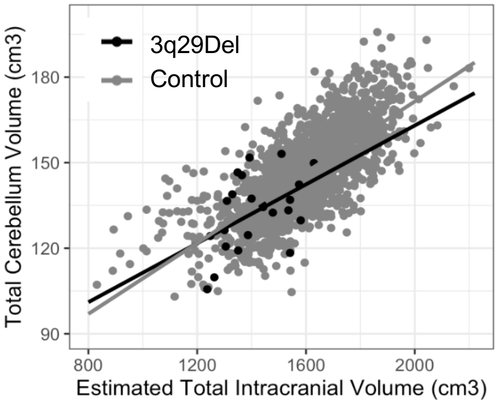

$$\mathbf{3q29Del} \mid Total\ \widehat{Cerebellum\ Volume} = 82.77^{***} - 0.31\,(Age) + 9.68\,(Sex_M)^{\dagger} + 0.03\,(eICV)$$

$$\mathbf{Control} \mid Total\ \widehat{Cerebellum\ Volume} = 60.15^{***} - 0.04\,(Age) + 5.31\,(Sex_M)^{***} + 0.05\,(eICV)^{***}$$

Pooled analysis in 3q29Del & control participants:
eICV X Diagnostic group interaction effect: *b* = -0.01, 95% CI = -0.04 – 0.03, p-value = 0.67

B

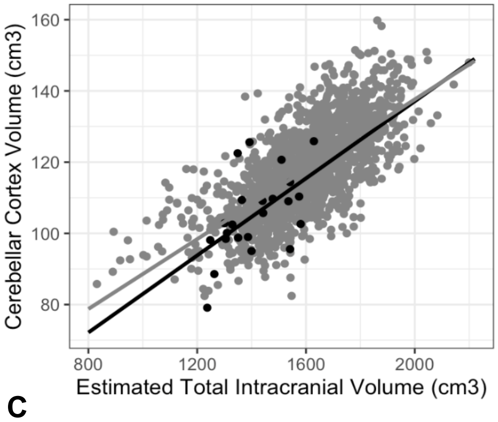

$$\mathbf{3q29Del} \mid Cerebellar\ \widehat{Cortex}\ Volume = 48.77 - 0.31\,(Age) + 5.99\,(Sex_M) + 0.04\,(eICV)^{*}$$

$$\mathbf{Control} \mid Cerebellar\ \widehat{Cortex}\ Volume = 55.61^{***} - 0.21\,(Age)^{***} + 4.75\,(Sex_M)^{***} + 0.04\,(eICV)^{***}$$

Pooled analysis in 3q29Del & control participants:
eICV X Diagnostic group interaction effect: *b* = 0.004, 95% CI = -0.03 – 0.03, p-value = 0.80

C

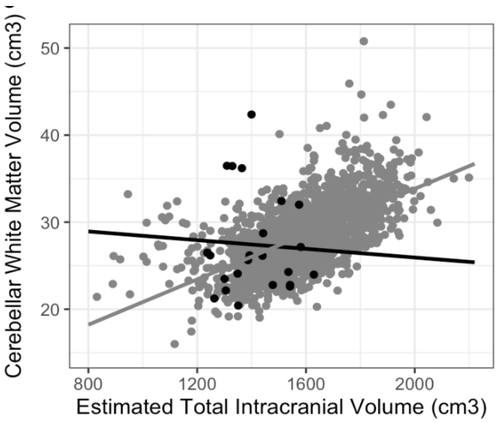

$$\mathbf{3q29Del} \mid Cerebellar\ \widehat{WM}\ Volume = 34.01 + 0.001\,(Age) + 3.69\,(Sex_M) - 0.01\,(eICV)$$

$$\mathbf{Control} \mid Cerebellar\ \widehat{WM}\ Volume = 4.53^{***} + 0.16\,(Age)^{***} + 0.56\,(Sex_M)^{**} + 0.01\,(eICV)^{***}$$

Pooled analysis in 3q29Del & control participants:
eICV X Diagnostic group interaction effect: *b* = -0.01, 95% CI = -0.02 – -0.001, p-value = 0.03*

Fig. S5. Relationships between eICV and A) total cerebellum, B) cerebellar cortex, and C) cerebellar white matter volumes among 3q29Del participants versus controls. Volumes reflect FreeSurfer-based morphometrics. The grey and black lines represent the estimated linear regression lines for control and 3q29Del groups, respectively. Corresponding multiple linear regression equations for each outcome, with age and sex added as covariates, are provided on the right. Four out of six group-specific intercepts were significantly different from zero (*p's* ≤ 0.05), indicating that the data do not meet the assumptions for using the "proportion method" for head size adjustment. When 3q29Del and control data were pooled, a significant interaction effect was identified between eICV and diagnostic group on cerebellar white matter volumes, while correcting for age and sex (*p* ≤ 0.05), with a significant positive slope observed among controls (*b* = 0.01, *p* ≤ 0.05) and a negative but not significant slope observed among 3q29Del participants (*b* = -0.01, *p* > 0.05). These inhomogeneous regression slopes, along with non-zero intercept tests support the implementation of the "residual method" as a statistically appropriate approach for eICV-adjustment of cerebellar volumes in downstream analyses. Refer to Mathalon et al. (1993), O'Brien et al. (2011) and Voevodskaya et al. (2014) for details on statistical considerations for head size adjustment. 3q29Del *N* = 23, Control *N* = 1,608. Contrast coding: reference level for sex in regression models is female. p-value ≤ 0.001 '***', p-value ≤ 0.01 '**', p-value ≤ 0.05 '*', p-value ≤ 0.1 '†'. *Abbreviations*: 3q29 deletion syndrome, 3q29Del; unstandardized coefficient estimate, *b*; confidence interval, CI; white matter, WM; estimated total intracranial volume, eICV; male, M.

A

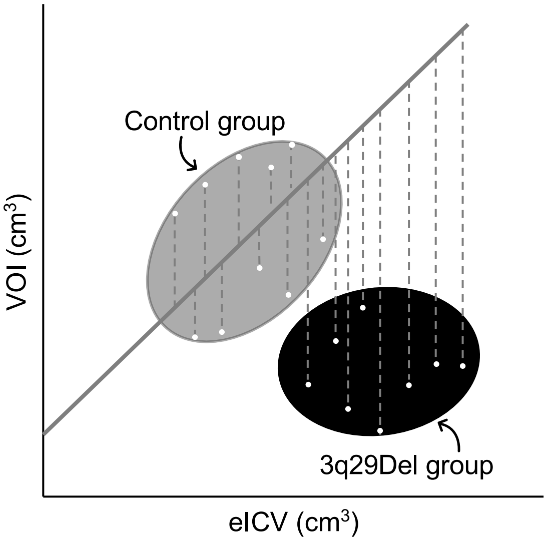

B

Using i to indicate a given participant, obtain parameter estimates using the control data only:

$$Estimated(VOI_i)_{control} = b_{0\,control} + b_{1_{control}}(eICV_i) + b_{2_{control}}(Sex_i) + b_{3_{control}}(Age_i).$$

Generate residuals using the estimates from this model for all participants and calculate eICV-adjusted VOIs:

$$Adjusted(VOI_i) = VOI_i - b_{control} \times (eICV_i - \overline{eICV}_{control})$$

; where $\overline{eICV}_{control}$ is the mean $eICV$ of the control group.

$$Adjusted(VOI_i) = \underbrace{VOI_i - (b_{1_{control}} \times eICV_i)}_{residual_i} + \underbrace{(b_{control} \times \overline{eICV}_{control})}_{\overline{VOI}_{control}},$$

$$Adjusted(VOI_i) = residual_i + \overline{VOI}_{control}.$$

Fig. S6. Correction method for adjustment of cerebellar volumes for head size variation in case-control comparisons. Absolute cerebellar volumes were adjusted for eICV using the residual method for head size correction (statistical justification for methodology is outlined in Fig. S5). **A)** A schematic illustration of the residual method for calculating eICV-adjusted VOIs, modified from O'Brien et al. (2011). The gray and black ellipses denote illustrative scatterplots for control and 3q29Del groups, respectively. The solid gray line represents a least-square derived linear regression between eICV and a given VOI, computed based on data from the control group only. Dashed vertical lines illustrate a sample of residuals calculated from this regression line for both control and 3q29Del participants. **B)** Formulas used for calculating eICV-adjusted VOIs based on the residual approach (disregarding error). We assume that the regression slope, *b1* represents the normative relationship between eICV and cerebellar volumes and that this relationship is not necessarily sustained in the 3q29Del group. Using the residual approach, we regress the VOI of neurotypical controls on the eICV of neurotypical controls, including age and sex as covariates, and use the estimates obtained from this linear regression model to calculate residuals for all participants from both diagnostic groups. Hence, each residual represents the deviation of a given participant's observed VOI from what would be expected of a control participant with the same eICV. Previous work has shown that the residual method is generally robust to systematic and random errors in MRI datasets (Sanfilipo et al., 2004), which provides advantages for detecting true group differences in combined datasets. Refer to Mathalon et al. (1993), O'Brien et al. (2011) and Voevodskaya et al. (2014) for details on statistical considerations for head size adjustment. *Abbreviations*: 3q29 deletion syndrome, 3q29Del; estimated total intracranial volume, eICV; volumetric measure of interest, VOI.

Mathalon DH, Sullivan EV, Rawles JM, Pfefferbaum A (1993): Correction for head size in brain-imaging measurements. *Psychiatry Res*. 50:121-139.

O'Brien LM, Ziegler DA, Deutsch CK, Frazier JA, Herbert MR, Locascio JJ (2011): Statistical adjustments for brain size in volumetric neuroimaging studies: some practical implications in methods. *Psychiatry Res*. 193:113-122.

Sanfilipo MP, Benedict RH, Zivadinov R, Bakshi R (2004): Correction for intracranial volume in analysis of whole brain atrophy in multiple sclerosis: the proportion vs. residual method. *Neuroimage*. 22:1732-1743.

Voevodskaya O, Simmons A, Nordenskjold R, Kullberg J, Ahlstrom H, Lind L, et al. (2014): The effects of intracranial volume adjustment approaches on multiple regional MRI volumes in healthy aging and Alzheimer's disease. *Front Aging Neurosci*. 6:264.

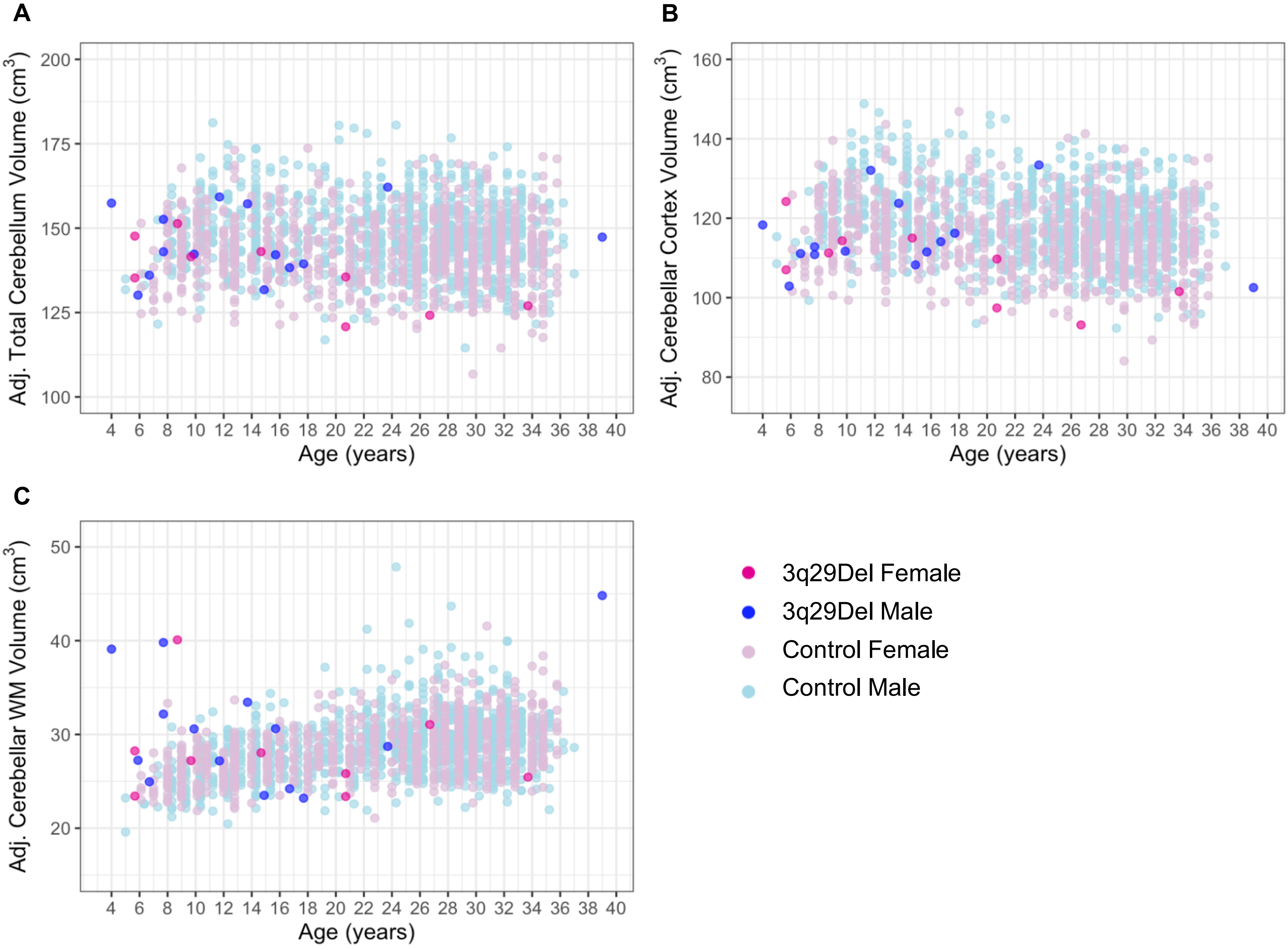

Fig. S7. Scatter plots showing the distribution of eICV-adjusted A) total cerebellum volume, B) cerebellar cortex volume, and C) cerebellar white matter volume as a function of age among male and female participants in each diagnostic group. Volumes reflect FreeSurfer-based morphometrics. A slight jitter was added systematically to all panels to minimize overplotting. Control *N* = 1,608 (Female *N* = 861, Male *N* = 747), 3q29Del *N* = 23 (Female *N* = 9, Male *N* = 14). *Abbreviations*: 3q29 deletion syndrome, 3q29Del; adjusted for estimated total intracranial volume, adj.; white matter, WM.

Table S3. Extended multiple linear regression results testing the effect of diagnostic group on tissue-specific cerebellar volumes and eICV, with polynomial modeling of age. A-H) Volumes reflect FreeSurfer-based morphometrics. Multiple linear regression models include sex, age, age^2 and/or age^3 as covariates. The main effect of diagnostic group is reported in bold for clarity. ANOVAs were performed to sequentially compare simpler models to more complex models to identify the best-fitting polynomial function of age for each VOI (highlighted in blue). The relationship between the best-fitting polynomial function of age and VOIs is plotted in the right bottom corner of each panel using data pooled across diagnostic groups. Final inferences are based on heteroskedasticity-robust estimates, which are provided above non-robust OLS estimates (in grey brackets), along with robust Wald statistics, for the best-fitting models only. We also report exact p-values calculated by non-asymptotic permutation marginal tests. Contrast coding: reference levels for the diagnostic group and sex variables are neurotypical control and female, respectively.

A. VOI: Absolute Total Cerebellum Volume (cm^3)

Degree of polynomial	Explanatory variables	*b*	CI (95%)	p-value	perm. p-value
**Linear (Model 1)**	Intercept	140.44	138.52 – 142.36	< 2.00E-16***	
	Age (years)	-0.10	-0.17 – -0.02	0.01**	0.01**
	Sex [Male]	16.50	15.27 – 17.74	< 2.00E-16***	1.00E-04***
	**Diagnostic Group [3q29Del]**	**-16.25**	**-21.50 – -11.00**	**1.57E-09*** **	**1.00E-04*** **
	R^2 / R^2 adjusted	0.31 / 0.31			
	F-statistic (OLS)	241.7 on 3 and 1627 DF, p-value < 2.20E-16***			
**Quadratic (Model 2)** *Best-fit*	Intercept	133.50	128.97 – 138.03 [129.05 – 137.96]	< 2.00E-16*** [< 2.00E-16***]	
	Age (years)	0.68	0.22 – 1.14 [0.22 – 1.13]	3.72E-03** [3.63E-03**]	3.30E-03***
	Age^2	-0.02	-0.03 – -0.01 [-0.03 – -0.01]	6.22E-04*** [7.31E-04***]	1.00E-03***
	Sex [Male]	16.22	14.97 – 17.48 [14.98 – 17.47]	< 2.00E-16*** [< 2.00E-16***]	1.00E-04***
	**Diagnostic Group [3q29Del]**	**-15.26**	**-20.01 – -10.51 [-20.53 – -10.00]**	**3.76E-10*** [1.53E-08***]**	**1.00E-04*** **
	R^2 / R^2 adjusted	0.31 / 0.31			
	Robust Wald test	184.0 on 4 and 1626 DF, p-value < 2.20E-16***			
	[F-statistic (OLS)	185.3 on 4 and 1626 DF, p-value < 2.20E-16***]			
**Cubic (Model 3)**	Intercept	127.70	117.23 – 138.16	< 2.00E-16***	
	Age (years)	1.69	-0.02 – 3.39	0.06†	0.06†
	Age^2	-0.07	-0.15 – 0.01	0.11	0.11
	Age^3	0.001	-0.001 – 0.002	0.23	0.23
	Sex [Male]	16.25	15.01 – 17.49	< 2.00E-16***	1.00E-04***
	**Diagnostic Group [3q29Del]**	**-15.10**	**-20.37 – -9.83**	**2.25E-08*** **	**1.00E-04*** **
	R^2 / R^2 adjusted	0.31 / 0.31			
	F-statistic (OLS)	148.6 on 5 and 1625 DF, p-value < 2.20E-16***			

Model 1 vs Model 2 – ANOVA Table:

	Resid. DF	Resid. SS	DF	SS	F-value	Pr(>F)
1	1627	260917				
2	1626	259092	1	1824.90	11.45	7.31E-04***

Model 2 vs Model 3 – ANOVA Table:

	Resid. DF	Resid. SS	DF	SS	F-value	Pr(>F)
2	1626	259092				
3	1625	258862	1	230.30	1.45	0.23

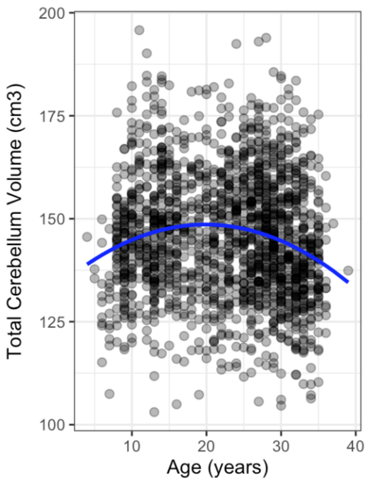

B. VOI: Absolute Cerebellar Cortex Volume (cm^3)

Degree of polynomial	Explanatory variables	*b*	CI (95%)	p-value	perm. p-value
**Linear (Model 1)**	Intercept	116.86	115.29 – 118.43	< 2.00E-16***	
	Age (years)	-0.25	-0.31 – -0.19	3.87E-15***	1.00E-04***
	Sex [Male]	13.27	12.25 – 14.28	< 2.00E-16***	1.00E-04***
	**Diagnostic Group [3q29Del]**	**-15.85**	**-20.15 – -11.55**	**7.27E-13*** **	**1.00E-04*** **
	R^2 / R^2 adjusted	0.32 / 0.32			
	F-statistic (OLS)	257.9 on 3 and 1627 DF, p-value < 2.20E-16***			
**Quadratic (Model 2)** *Best-fit*	Intercept	113.52	109.73 – 117.31 [109.86 – 117.18]	< 2.00E-16*** [< 2.00E-16***]	
	Age (years)	0.13	-0.26 – 0.51 [-0.25 – 0.50]	0.52 [0.51]	0.50
	Age^2	-0.01	-0.02 – -0.0001 [-0.02 – -0.0001]	0.04* [0.04*]	0.04*
	Sex [Male]	13.13	12.10 – 14.17 [12.11 – 14.15]	< 2.00E-16*** [< 2.00E-16***]	1.00E-04***
	**Diagnostic Group [3q29Del]**	**-15.38**	**-19.53 – -11.22 [-19.70 – -11.06]**	**5.86E-13*** [4.28E-12***]**	**1.00E-04*** **
	R^2 / R^2 adjusted	0.32 / 0.32			
	Robust Wald test	193.2 on 4 and 1626 DF, p-value < 2.20E-16***			
	[F-statistic (OLS)	194.8 on 4 and 1626 DF, p-value < 2.20E-16***]			
**Cubic (Model 3)**	Intercept	107.48	98.89 – 116.07	< 2.00E-16 ***	
	Age (years)	1.18	-0.23 – 2.58	0.10†	0.10†
	Age^2	-0.06	-0.13 – 0.01	0.08†	0.08†
	Age^3	0.001	-0.0002 – 0.002	0.13	0.13
	Sex [Male]	13.16	12.14 – 14.17	< 2.00E-16 ***	1.00E-04 ***
	**Diagnostic Group [3q29Del]**	**-15.21**	**-19.53 – -10.88**	**7.57E-12 *** **	**1.00E-04 *** **
	R^2 / R^2 adjusted	0.32 / 0.32			
	F-statistic (OLS)	156.4 on 5 and 1625 DF, p-value < 2.20E-16 ***			

Model 1 vs Model 2 – ANOVA Table:

	Resid. DF	Resid. SS	DF	SS	F-value	Pr(>F)
**1**	1627	174977				
**2**	1626	174554	1	422.96	3.94	0.04 *

Model 2 vs Model 3 – ANOVA Table:

	Resid. DF	Resid. SS	DF	SS	F-value	Pr(>F)
**2**	1626	174554				
**3**	1625	174305	1	249.44	2.33	0.13

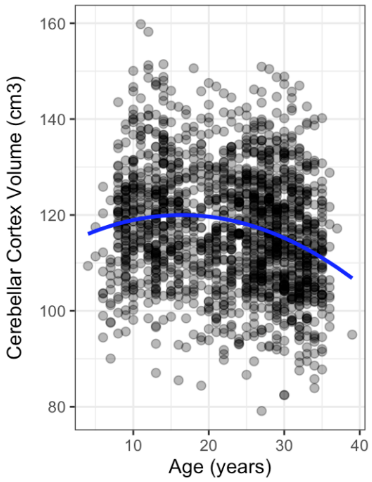

C. VOI: Absolute Cerebellar White Matter Volume (cm^3)

Degree of polynomial	Explanatory variables	*b*	CI (95%)	p-value	perm. p-value
**Linear (Model 1)**	Intercept	23.58	23.07 – 24.10	< 2.00E-16***	
	Age (years)	0.15	0.13 – 0.17	< 2.00E-16***	1.00E-04***
	Sex [Male]	3.24	2.90 – 3.57	< 2.00E-16***	1.00E-04***
	**Diagnostic Group [3q29Del]**	**-0.40**	**-1.80 – 1.01**	**0.58**	**0.57**
	R^2 / R^2 adjusted	0.26 / 0.26			
	F-statistic (OLS)	187.3 on 3 and 1627 DF, p-value < 2.20E-16 ***			
**Quadratic (Model 2)** *Best-fit*	Intercept	19.98	18.82 – 21.15 [18.80 – 21.16]	< 2.00E-16*** [< 2.00E-16***]	
	Age (years)	0.55	0.43 – 0.67 [0.43 – 0.67]	< 2.00E-16*** [< 2.00E-16***]	1.00E-04***
	Age^2	-0.01	-0.01 – -0.01 [-0.01 – -0.01]	8.88E-11*** [4.94E-11***]	1.00E-04***
	Sex [Male]	3.09	2.76 – 3.43 [2.76 – 3.42]	< 2.00E-16*** [< 2.00E-16***]	1.00E-04***
	**Diagnostic Group [3q29Del]**	**0.11**	**-2.42 – 2.65 [-1.28 – 1.51]**	**0.93 [0.87]**	**0.87**
	R^2 / R^2 adjusted	0.28 / 0.27			
	Robust Wald test	155.1 on 4 and 1626 DF, p-value < 2.20E-16***			
	[F-statistic (OLS)	155.1 on 4 and 1626 DF, p-value < 2.20E-16***]			
**Cubic (Model 3)**	Intercept	20.22	17.45 – 23.00	< 2.00E-16***	
	Age (years)	0.51	0.06 – 0.96	0.03*	0.03*
	Age^2	-0.01	-0.03 – 0.02	0.52	0.53
	Age^3	0.00	-0.0004 – 0.0003	0.85	0.85
	Sex [Male]	3.09	2.76 – 3.42	< 2.00E-16***	1.00E-04***
	**Diagnostic Group [3q29Del]**	**0.11**	**-1.29 – 1.51**	**0.88**	**0.87**
	R^2 / R^2 adjusted	0.28 / 0.27			
	F-statistic (OLS)	124.0 on 5 and 1625 DF, p-value < 2.20E-16 ***			

Model 1 vs Model 2 – ANOVA Table:

	Resid. DF	Resid. SS	DF	SS	F-value	Pr(>F)
**1**	1627	18695				
**2**	1626	18205	1	490.30	43.79	4.94E-11***

Model 2 vs Model 3 – ANOVA Table:

	Resid. DF	Resid. SS	DF	SS	F-value	Pr(>F)
**2**	1626	18205				
**3**	1625	18204	1	0.39	0.04	0.85

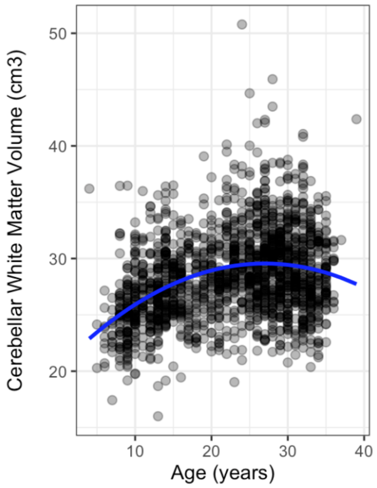

D. VOI: Cerebellar Cortex to White Matter Volume Ratio

Degree of polynomial	Explanatory variables	*b*	CI (95%)	p-value	perm. p-value
**Linear (Model 1)**	Intercept	4.88	4.82 – 4.94	< 2.00E-16***	
	Age (years)	-0.03	-0.03 – -0.03	< 2.00E-16***	1.00E-04***
	Sex [Male]	0.01	-0.03 – 0.05	0.56	0.56
	**Diagnostic Group [3q29Del]**	**-0.42**	**-0.58 – -0.25**	**5.35E-07*** **	**1.00E-04*** **
	R^2 / R^2 adjusted	0.31 / 0.31			
	F-statistic (OLS)	240.4 on 3 and 1627 DF, p-value < 2.20E-16***			
**Quadratic (Model 2)** *Best-fit*	Intercept	5.38	5.22 – 5.53 [5.22 – 5.53]	< 2.00E-16*** [< 2.00E-16***]	
	Age (years)	-0.09	-0.10 – -0.07 [-0.10 – -0.07]	< 2.00E-16*** [< 2.00E-16***]	1.00E-04***
	Age^2	0.001	0.001 – 0.002 [0.001 – 0.002]	9.59E-13*** [2.46E-15***]	1.00E-04***
	Sex [Male]	0.03	-0.01 – 0.07 [-0.01 – 0.07]	0.11 [0.11]	0.11
	**Diagnostic Group [3q29Del]**	**-0.49**	**-0.86 – -0.12 [-0.65 – -0.33]**	**9.82E-03** [2.92E-09***]**	**1.00E-04*** **
	R^2 / R^2 adjusted	0.33 / 0.33			
	Robust Wald test	179.4 on 4 and 1626 DF, p-value < 2.20E-16***			
	[F-statistic (OLS)	203.2 on 4 and 1626 DF, p-value < 2.20E-16***]			
**Cubic (Model 3)**	Intercept	5.30	4.98 – 5.62	< 2.00E-16***	
	Age (years)	-0.07	-0.13 – -0.02	0.01**	0.01**
	Age^2	0.001	-0.002 – 0.003	0.63	0.63
	Age^3	0.00	0.00 – 0.0001	0.60	0.59
	Sex [Male]	0.03	-0.01 – 0.07	0.10†	0.11
	**Diagnostic Group [3q29Del]**	**-0.49**	**-0.65 – -0.33**	**3.61E-09*** **	**1.00E-04*** **
	R^2 / R^2 adjusted	0.33 / 0.33			
	F-statistic (OLS)	162.6 on 5 and 1625 DF, p-value < 2.2E-16 ***			

Model 1 vs Model 2 – ANOVA Table:

	Resid. DF	Resid. SS	DF	SS	F-value	Pr(>F)
**1**	1627	250.16				
**2**	1626	240.70	1	9.46	63.90	2.46E-15***

Model 2 vs Model 3 – ANOVA Table:

	Resid. DF	Resid. SS	DF	SS	F-value	Pr(>F)
**2**	1626	240.70				
**3**	1625	240.66	1	0.04	0.27	0.60

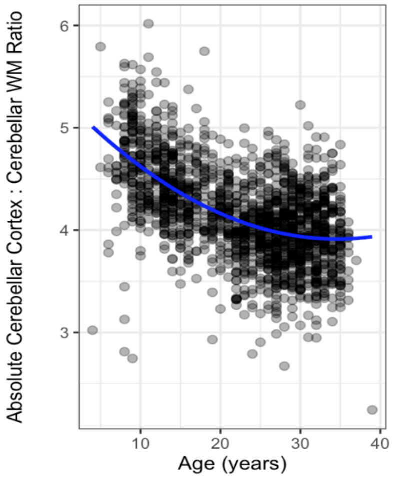

E. VOI: Estimated Total Intracranial Volume (eICV) (cm3)

Degree of polynomial	Explanatory variables	*b*	CI (95%)	p-value	perm. p-value
**Linear (Model 1)**	Intercept	1511.99	1490.34 – 1533.63	< 2.00E-16***	
	Age (years)	-0.97	-1.81 – -0.12	0.03*	0.02*
	Sex [Male]	209.85	195.94 – 223.77	< 2.00E-16***	1.00E-04***
	**Diagnostic Group [3q29Del]**	**-211.79**	**-270.97 – -152.61**	**3.26E-12*** **	**1.00E-04*** **
	R^2 / R^2 adjusted	0.36 / 0.36			
	F-statistic (OLS)	306.8 on 3 and 1627 DF, p-value < 2.20E-16***			
**Quadratic (Model 2)** *Best-fit*	Intercept	1415.02	1367.87 – 1462.17 [1364.89 – 1465.14]	< 2.00E-16*** [< 2.00E-16***]	
	Age (years)	9.89	4.84 – 14.94 [4.75 – 15.03]	1.26E-04*** [1.65E-04***]	2.00E-04***
	Age^2	-0.25	-0.37 – -0.13 [-0.37 – -0.14]	3.66E-05*** [2.79E-05***]	1.00E-04***
	Sex [Male]	206.00	192.06 – 219.93 [192.03 – 219.96]	< 2.00E-16*** [< 2.00E-16***]	1.00E-04***
	**Diagnostic Group [3q29Del]**	**-197.99**	**-253.23 – -142.74 [-257.22 – -138.75]**	**3.05E-12*** [7.39E-11***]**	**1.00E-04*** **
	R^2 / R^2 adjusted	0.37 / 0.37			
	Robust Wald test	232.5 on 4 and 1626 DF, p-value < 2.20E-16***			
	[F-statistic (OLS)	236.9 on 4 and 1626 DF, p-value < 2.20E-16***]			
**Cubic (Model 3)**	Intercept	1419.77	1301.95 – 1537.60	< 2.00E-16***	
	Age (years)	9.06	-10.14 – 28.27	0.36	0.36
	Age^2	-0.21	-1.16 – 0.74	0.66	0.66
	Age^3	-0.001	-0.02 – 0.01	0.93	0.93
	Sex [Male]	205.98	192.01 – 219.95	< 2.00E-16***	1.00E-04***
	**Diagnostic Group [3q29Del]**	**-198.12**	**-257.44 – -138.79**	**7.69E-11*** **	**1.00E-04*** **
	R^2 / R^2 adjusted	0.37 / 0.37			
	F-statistic (OLS)	189.4 on 5 and 1625 DF, p-value < 2.20E-16***			

Model 1 vs Model 2 – ANOVA Table:

	Resid. DF	Resid. SS	DF	SS	F-value	Pr(>F)
1	1627	33159001				
2	1626	32802768	1	356233	17.66	2.79E-05***

Model 2 vs Model 3 – ANOVA Table:

	Resid. DF	Resid. SS	DF	SS	F-value	Pr(>F)
2	1626	32802768				
3	1625	32802613	1	154.45	0.01	0.93

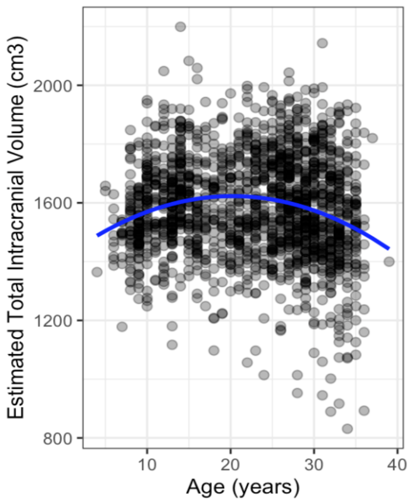

F. VOI: eICV-Adjusted Total Cerebellum Volume (cm³)

Degree of polynomial	Explanatory variables	*b*	CI (95%)	p-value	perm. p-value
**Linear (Model 1)** *Best-fit*	Intercept	144.44	142.97 – 145.92 [142.90 – 145.99]	< 2.00E-16*** [< 2.00E-16***]	
	Age (years)	-0.05	-0.11 – 0.01 [-0.11 – 0.01]	0.12 [0.12]	0.12
	Sex [Male]	5.38	4.38 – 6.38 [4.39 – 6.37]	< 2.00E-16*** [< 2.00E-16***]	1.00E-04***
	**Diagnostic Group [3q29Del]**	**-5.02**	**-9.25 – -0.80** **[-9.24 – -0.81]**	**0.02*** **[0.02*]**	**0.02***
	R^2 / R^2 adjusted	0.07 / 0.07			
	Robust Wald test	39.0 on 3 and 1627 DF, p-value < 2.20E-16***			
	[F-statistic (OLS)	40.1 on 3 and 1627 DF, p-value < 2.20E-16***]			
**Quadratic (Model 2)**	Intercept	142.64	139.06 – 146.23	< 2.00E-16***	
	Age (years)	0.15	-0.21 – 0.52	0.41	0.41
	Age^2	-0.005	-0.01 – 0.004	0.28	0.27
	Sex [Male]	5.31	4.31 – 6.31	< 2.00E-16***	1.00E-04***
	**Diagnostic Group [3q29Del]**	**-4.77**	**-9.01 – -0.53**	**0.03***	**0.03***
	R^2 / R^2 adjusted	0.07 / 0.07			
	F-statistic (OLS)	30.4 on 4 and 1626 DF, p-value < 2.20E-16***			
**Cubic (Model 3)**	Intercept	136.59	128.16 – 145.02	< 2.00E-16***	
	Age (years)	1.21	-0.17 – 2.58	0.09†	0.08†
	Age^2	-0.06	-0.13 – 0.01	0.09†	0.09†
	Age^3	0.001	-0.0002 – 0.002	0.12	0.12
	Sex [Male]	5.33	4.33 – 6.33	< 2.00E-16***	1.00E-04***
	**Diagnostic Group [3q29Del]**	**-4.60**	**-8.84 – -0.35**	**0.03***	**0.03***
	R^2 / R^2 adjusted	0.07 / 0.07			
	F-statistic (OLS)	24.8 on 5 and 1625 DF, p-value < 2.20E-16***			

Model 1 vs Model 2 – ANOVA Table:

	Resid. DF	Resid. SS	DF	SS	F-value	Pr(>F)
**1**	1627	168327				
**2**	1626	168204	1	122.66	1.19	0.28

Model 2 vs Model 3 – ANOVA Table:

	Resid. DF	Resid. SS	DF	SS	F-value	Pr(>F)
**2**	1626	168204				
**3**	1625	167953	1	250.74	2.43	0.12

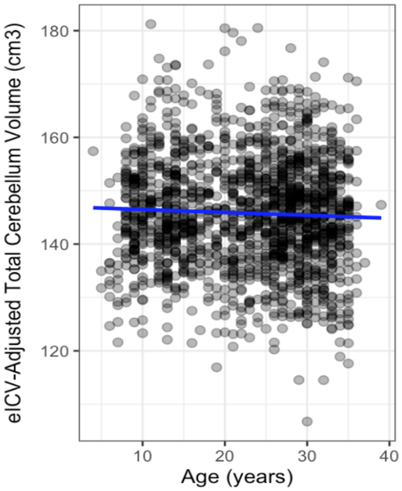

G. VOI: eICV-Adjusted Cerebellar Cortex Volume (cm^3)

Degree of polynomial	Explanatory variables	*b*	CI (95%)	p-value	perm. p-value
**Linear (Model 1)**<br>*Best-fit*	Intercept	119.88	118.60 – 121.17<br>[118.58 – 121.19]	< 2.00E-16***<br>[< 2.00E-16***]	
	Age (years)	-0.21	-0.26 – -0.16<br>[-0.26 – -0.16]	1.37E-15***<br>[1.42E-15***]	1.00E-04***
	Sex [Male]	4.87	4.03 – 5.72<br>[4.03 – 5.71]	< 2.00E-16***<br>[< 2.00E-16***]	1.00E-04***
	**Diagnostic Group [3q29Del]**	**-7.38**	**-10.98 – -3.78**<br>**[-10.95 – -3.81]**	**6.03E-05***<br>**[5.28E-05***]**	**1.00E-04***
	R^2 / R^2 adjusted	0.11 / 0.11			
	Robust Wald test	67.5 on 3 and 1627 DF, p-value < 2.20E-16***			
	[F-statistic (OLS)	70.1 on 3 and 1627 DF, p-value < 2.20E-16***]			
**Quadratic (Model 2)**	Intercept	120.42	117.38 – 123.46	< 2.00E-16***	
	Age (years)	-0.27	-0.58 – 0.04	0.09†	0.09†
	Age^2	0.001	-0.01 – 0.01	0.70	0.70
	Sex [Male]	4.89	4.05 – 5.74	< 2.00E-16***	1.00E-04***
	**Diagnostic Group [3q29Del]**	**-7.46**	**-11.05 – -3.86**	**4.92E-05***	**2.00E-04***
	R^2 / R^2 adjusted	0.11 / 0.11			
	F-statistic (OLS)	52.6 on 4 and 1626 DF, p-value < 2.20E-16 ***			
**Cubic (Model 3)**	Intercept	114.19	107.05 – 121.33	< 2.00E-16***	
	Age (years)	0.81	-0.35 – 1.98	0.17	0.17
	Age^2	-0.05	-0.11 – 0.00	0.07†	0.07†
	Age^3	0.001	0.00 – 0.002	0.06†	0.06†
	Sex [Male]	4.92	4.07 – 5.76	< 2.00E-16***	1.00E-04***
	**Diagnostic Group [3q29Del]**	**-7.28**	**-10.88 – -3.69**	**7.40E-05***	**3.00E-04***
	R^2 / R^2 adjusted	0.12 / 0.11			
	F-statistic (OLS)	2442.8 on 5 and 1625 DF, p-value < 2.20E-16***			

Model 1 vs Model 2 – ANOVA Table:

	Resid. DF	Resid. SS	DF	SS	F-value	Pr(>F)
**1**	1627	120694				
**2**	1626	120683	1	10.95	0.15	0.70

Model 2 vs Model 3 – ANOVA Table:

	Resid. DF	Resid. SS	DF	SS	F-value	Pr(>F)
**2**	1626	120683				
**3**	1625	120418	1	265.34	3.58	0.06†

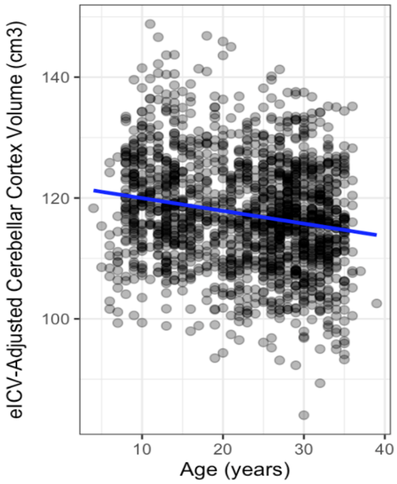

H. VOI: eICV-Adjusted Cerebellar White Matter Volume (cm^3)

Degree of polynomial	Explanatory variables	*b*	CI (95%)	p-value	perm. p-value
**Linear (Model 1)**	Intercept	24.56	24.12 – 25.00	< 2.00E-16***	
	Age (years)	0.16	0.14 – 0.18	< 2.00E-16***	1.00E-04***
	Sex [Male]	0.51	0.23 – 0.79	4.33E-04***	8.00E-04***
	**Diagnostic Group [3q29Del]**	**2.36**	**1.16 – 3.55**	**1.22E-04*** **	**3.00E-04*** **
	R^2 / R^2 adjusted	0.18 / 0.18			
	F-statistic (OLS)	118.2 on 3 and 1627 DF, p-value < 2.20E-16***			
**Quadratic (Model 2)** *Best-fit*	Intercept	22.22	21.22 – 23.23 [21.21 – 23.24]	< 2.00E-16*** [< 2.00E-16***]	
	Age (years)	0.42	0.31 – 0.53 [0.32 – 0.53]	6.39E-14*** [2.53E-15***]	1.00E-04***
	Age^2	-0.01	-0.01 – -0.004 [-0.01 – -0.004]	3.94E-06*** [6.12E-07***]	1.00E-04***
	Sex [Male]	0.41	0.13 – 0.70 [0.13 – 0.70]	4.51E-03** [4.07E-03**]	4.50E-03**
	**Diagnostic Group [3q29Del]**	**2.69**	**0.10 – 5.28 [1.49 – 3.89]**	**0.04* [1.15E-05***]**	**1.00E-04*** **
	R^2 / R^2 adjusted	0.19 / 0.19			
	Robust Wald test	106.7 on 4 and 1626 DF, p-value < 2.20E-16***			
	[F-statistic (OLS)	96.2 on 4 and 1626 DF, p-value < 2.20E-16***]			
**Cubic (Model 3)**	Intercept	22.40	20.02 – 24.78	< 2.00E-16 ***	
	Age (years)	0.39	0.004 – 0.78	0.04*	0.04*
	Age^2	-0.005	-0.02 – 0.01	0.64	0.63
	Age^3	0.00	-0.003 – 0.003	0.87	0.87
	Sex [Male]	0.41	0.13 – 0.70	4.16E-03**	4.50E-03**
	**Diagnostic Group [3q29Del]**	**2.68**	**1.48 – 3.88**	**1.23E-05*** **	**1.00E-04*** **
	R^2 / R^2 adjusted	0.19 / 0.19			
	F-statistic (OLS)	77.0 on 5 and 1625 DF, p-value < 2.20E-16***			

Model 1 vs Model 2 – ANOVA Table:

	Resid. DF	Resid. SS	DF	SS	F-value	Pr(>F)
**1**	1627	13626				
**2**	1626	13420	1	206.93	25.07	6.12E-07***

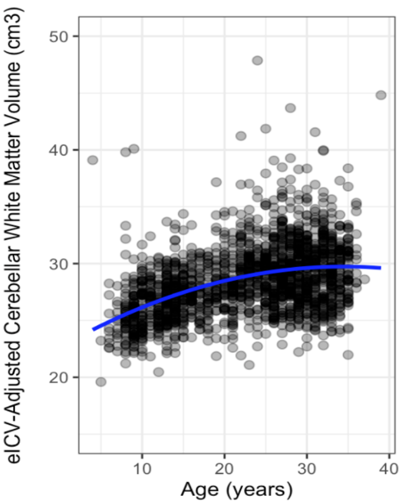

Model 2 vs Model 3 – ANOVA Table:

	Resid. DF	Resid. SS	DF	SS	F-value	Pr(>F)
**2**	1626	13420				
**3**	1625	13419	1	0.21	0.03	0.87

Control *N* = 1,608, 3q29Del *N* = 23. p-value ≤ 0.001 '***', p-value ≤ 0.01 '**', p-value ≤ 0.05 '*', p-value ≤ 0.1 '†'. *Abbreviations*: 3q29 deletion syndrome, 3q29Del; VOI, volumetric measure of interest; eICV, estimated total intracranial volume; unstandardized coefficient estimate, *b*; confidence interval, CI; degrees of freedom, DF; sum of squares, SS; analysis of variance, ANOVA; permutation, perm; ordinary least squares, OLS; residual, resid.

Fig. S8. Regression diagnostics: testing the assumptions of ordinary least squares regression for best-fitting models from Table S3. A-H) Plots of residuals versus predicted values for checking the assumptions of linearity and homoscedasticity in multiple linear regression. The residuals should appear approximately linear and be evenly spread around the red $y = 0$ line (i.e., slope & y-intercept = 0) to assume a linear relationship between the predictors and the outcome variables and to assume homoscedasticity. **I-P)** QQ-plots of residuals for checking the normality assumption. The normal probability plot of residuals should approximately follow a 45-degree straight line to indicate that all errors are normally distributed around zero. Shapiro-Wilk normality tests were performed to additionally check that the residuals are normally distributed. **Q-X)** Scale-location plots for checking the homoscedasticity assumption. Standardized residuals reflect residuals divided by estimated standard error. Variance of the errors should be approximately the same for any combination of values of the independent variables. Studentized Breusch-Pagan tests were performed to additionally check for homoscedasticity. Given several observed violations of necessary linear regression assumptions, we calculated heteroscedasticity-robust estimates for final inferences. p-value ≤ 0.001 '***', p-value ≤ 0.01 '**', p-value ≤ 0.05 '*', p-value ≤ 0.1 '†'. *Abbreviations*: volumetric measure of interest, VOI; estimated total intracranial volume, eICV.

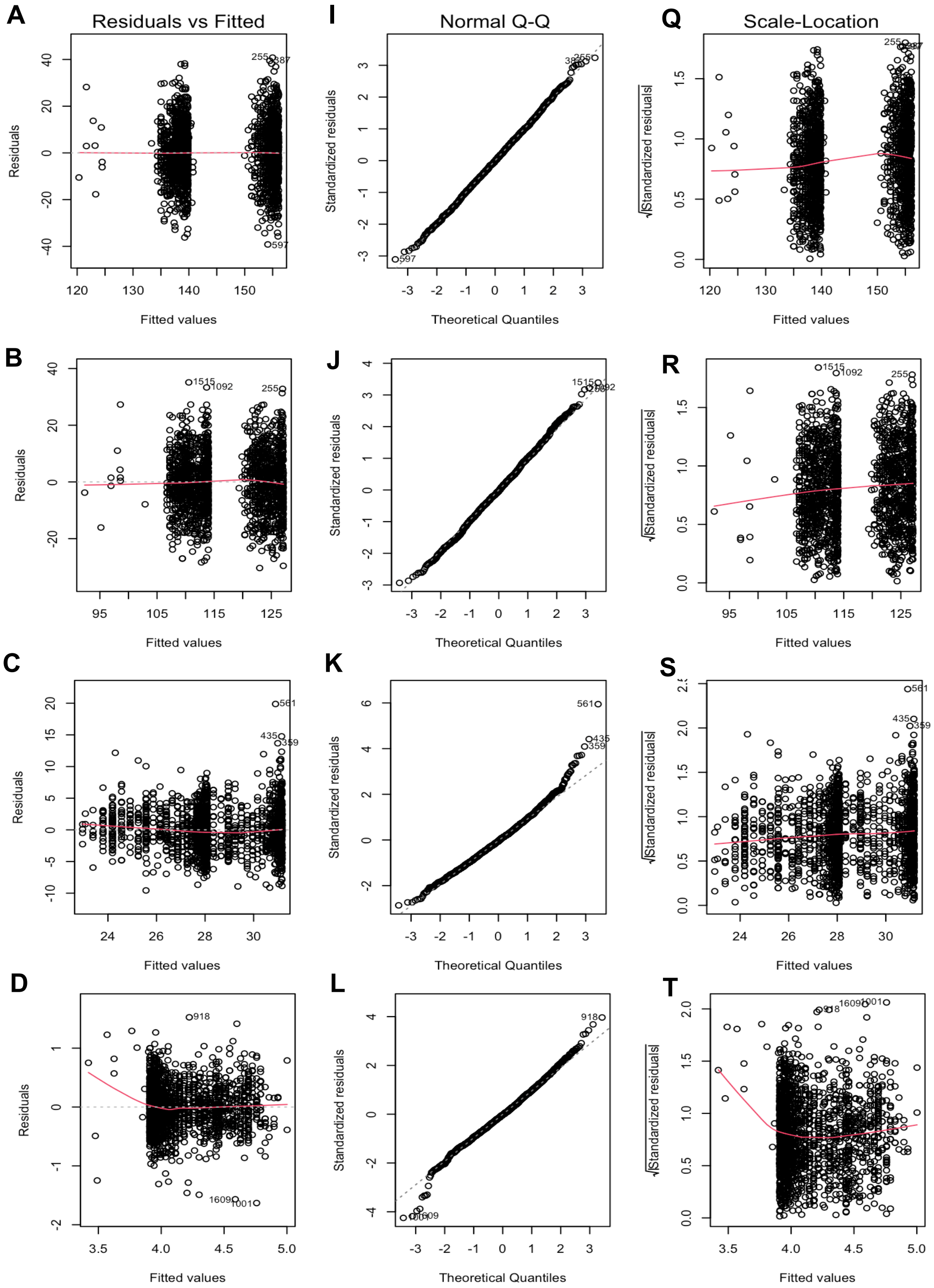

VOI: Absolute Total Cerebellum Volume
Best fit: Model 2 (refer to Table S3A)

Shapiro-Wilk test: W = 1.00, p-value = 0.80
Studentized Breusch-Pagan test: BP = 13.19, p-value = 0.01**.

VOI: Absolute Cerebellar Cortex Volume
Best fit: Model 2 (refer to Table S3B)

Shapiro-Wilk test: W = 1.00, p-value = 0.30
Studentized Breusch-Pagan test: BP = 11.05, p-value = 0.03*.

VOI: Absolute Cerebellar White Matter Volume
Best fit: Model 2 (refer to Table S3C)

Shapiro-Wilk test: W = 0.99, p-value = 2.15E-11***
Studentized Breusch-Pagan test: BP = 71.94, p-value = 8.85E-15***.

VOI: Cerebellar Cortex to White Matter Volume Ratio
Best fit: Model 2 (refer to Table S3D)

Shapiro-Wilk test: W = 0.99, p-value = 1.09E-06***
Studentized Breusch-Pagan test: BP = 180.9, p-value < 2.2E-16***.

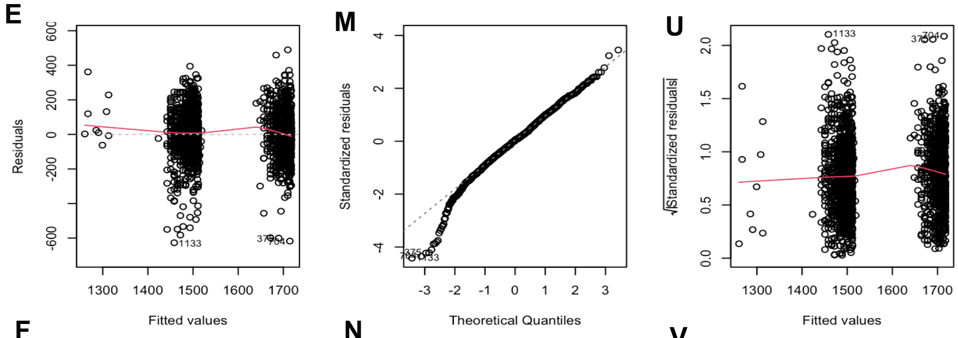

VOI: eICV
Best fit: Model 2 (refer to Table S3E)

Shapiro-Wilk test: W = 0.98, p-value = 1.66E-12***
Studentized Breusch-Pagan test: BP = 16.01, p-value = 3.00E-03**.

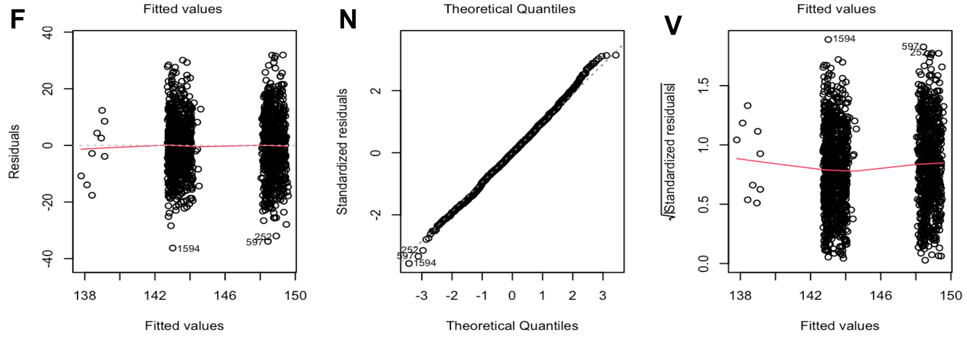

VOI: eICV-Adjusted Total Cerebellum Volume
Best fit: Model 1 (refer to Table S3F)

Shapiro-Wilk test: W = 1.00, p-value = 0.26
Studentized Breusch-Pagan test: BP = 12.86, p-value = 4.95E-03**.

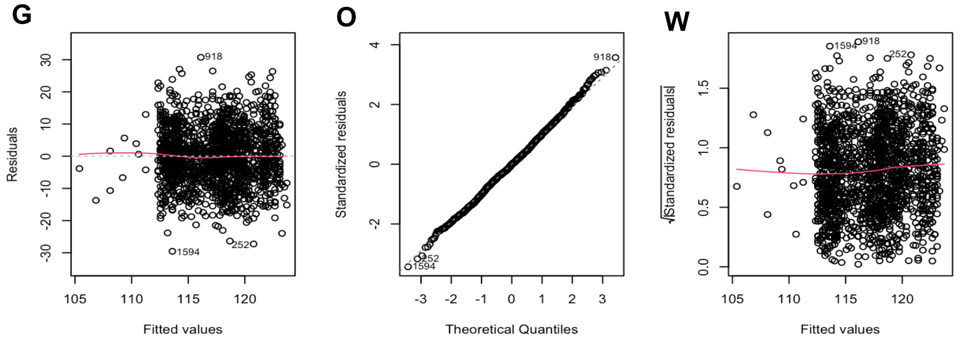

VOI: eICV-Adjusted Cerebellar Cortex Volume
Best fit: Model 1 (refer to Table S3G)

Shapiro-Wilk test: W = 1.00, p-value = 0.29
Studentized Breusch-Pagan test: BP = 6.67, p-value = 0.08†.

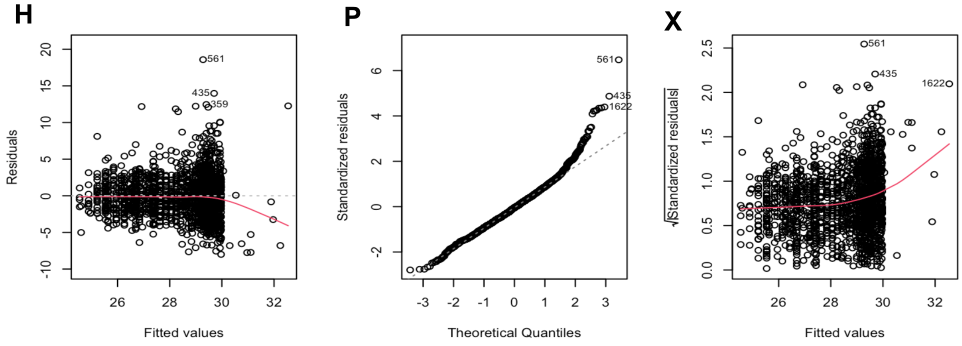

VOI: eICV-Adjusted Cerebellar White Matter Volume
Best fit: Model 2 (refer to Table S3H)

Shapiro-Wilk test: W = 0.97, p-value < 2.2E-16 ***
Studentized Breusch-Pagan test: BP = 121.71, p-value < 2.2E-16 ***.

Table S4. Sensitivity analysis: multiple linear regression results testing the effect of diagnostic group on tissue-specific cerebellar volumes and eICV, after removal of an outlier datapoint from the 3q29Del group. A-G) Volumes reflect FreeSurfer-based morphometrics. A 39-year-old male 3q29Del participant falling outside the age-range of neurotypical controls and the majority of the 3q29Del group was removed from the sample and the best-fitting models from Table S3 were re-run for sensitivity analysis. Models include sex, age and/or age^2 (when appropriate) as covariates. The main effect of diagnostic group is reported in bold for clarity. Final inferences are based on heteroskedasticity-robust estimates provided above OLS estimates (in grey brackets). We also report exact p-values by non-asymptotic permutation marginal tests. Contrast coding: reference levels for diagnostic group and sex are neurotypical control and female, respectively. Results from sensitivity analysis show consistent patterns with results in Table S3 for the full study sample.

VOI	Explanatory variables	*b*	CI (95%)	p-value	perm. p-value
**A. Absolute Total Cerebellum Volume (cm^3)**	Intercept	133.47	[128.93 – 138.01]<br>[129.01 – 137.93]	< 2.00E-16***<br>[< 2.00E-16***]	
	Age (years)	0.68	[0.22 – 1.14]<br>[0.23 – 1.14]	3.60E-03**<br>[3.45E-03**]	3.70E-03**
	Age^2	-0.02	[-0.03 – -0.01]<br>[-0.03 – -0.01]	6.08E-04***<br>[6.92E-04***]	7.00E-04***
	Sex [Male]	16.22	[14.96 – 17.48]<br>[14.98 – 17.46]	< 2.00E-16***<br>[< 2.00E-16***]	1.00E-04***
	**Diagnostic Group [3q29Del]**	**-15.45**	**[-20.39 – -10.51]**<br>**[-20.83 – -10.07]**	**1.06E-09*****<br>**[2.08E-08***]**	**1.00E-04*** **
	R^2 / R^2 adjusted	0.31 / 0.31			
	Robust Wald test	183.7 on 4 and 1625 DF, p-value < 2.20E-16***			
	[F-statistic (OLS)	185.1 on 4 and 1625 DF, p-value < 2.20E-16***]			
**B. Absolute Cerebellar Cortex Volume (cm^3)**	Intercept	113.59	[109.79 – 117.38]<br>[109.93 – 117.25]	< 2.00E-16***<br>[< 2.00E-16***]	
	Age (years)	0.12	[-0.27 – 0.50]<br>[-0.26 – 0.49]	0.56<br>[0.55]	0.54
	Age^2	-0.01	[-0.02 – 0.0003]<br>[-0.02 – 0.0002]	0.06†<br>[0.06†]	0.06†
	Sex [Male]	13.15	[12.11 – 14.18]<br>[12.13 – 14.17]	< 2.00E-16***<br>[< 2.00E-16***]	1.00E-04***
	**Diagnostic Group [3q29Del]**	**-15.02**	**[-19.29 – -10.74]**<br>**[-19.43 – -10.60]**	**7.87E-12*****<br>**[3.44E-11***]**	**1.00E-04*** **
	R^2 / R^2 adjusted	0.32 / 0.32			
	Robust Wald test	192.1 on 4 and 1625 DF, p-value < 2.20E-16***			
	[F-statistic (OLS)	193.7 on 4 and 1625 DF, p-value < 2.20E-16***]			
**C. Absolute Cerebellar White Matter Volume (cm^3)**	Intercept	19.88	18.73 – 21.04<br>[18.71 – 21.06]	< 2.00E-16***<br>[< 2.00E-16***]	
	Age (years)	0.57	0.45 – 0.69<br>[0.45 – 0.69]	< 2.00E-16***<br>[< 2.00E-16***]	1.00E-04***
	Age^2	-0.01	-0.01 – -0.01<br>[-0.01 – -0.01]	2.39E-12***<br>[6.01E-12***]	1.00E-04***
	Sex [Male]	3.07	2.74 – 3.40<br>[2.74 – 3.40]	< 2.00E-16***<br>[< 2.00E-16***]	1.00E-04***
	**Diagnostic Group [3q29Del]**	**-0.43**	**-2.85 – 1.98**<br>**[-1.85 – 0.99]**	**0.73**<br>**[0.55]**	**0.55**
	R^2 / R^2 adjusted	0.28 / 0.27			
	Robust Wald test	151.5 on 4 and 1625 DF, p-value < 2.20E-16***			
	[F-statistic (OLS)	155.3 on 4 and 1625 DF, p-value < 2.20E-16***]			

**D. Estimated Total Intracranial Volume (eICV) (cm^3)**	Intercept	1415.21	1367.90 – 1462.53<br>[1365.02 – 1465.40]	< 2.00E-16***<br>[< 2.00E-16***]	
	Age (years)	9.86	4.77 – 14.94<br>[4.71 – 15.01]	1.48E-04***<br>[1.81E-04***]	5.00E-04***
	Age^2	-0.25	-0.37 – -0.13<br>[-0.37 – -0.13]	4.56E-05***<br>[3.18E-05***]	1.00E-04***
	Sex [Male]	206.04	192.09 – 219.99<br>[192.06 – 220.01]	< 2.00E-16***<br>[< 2.00E-16***]	1.00E-04***
	**Diagnostic Group [3q29Del]**	**-196.91**	**-254.53 – -139.30**<br>**[-257.44 – -136.39]**	**2.80E-11*****<br>**[2.28E-10***]**	**1.00E-04*** **
	R^2 / R^2 adjusted	0.37 / 0.37			
	Robust Wald test	232.1 on 4 and 1625 DF, p-value < 2.20E-16***			
	[F-statistic (OLS)	236.3 on 4 and 1625 DF, p-value < 2.20E-16***]			
**E. eICV-Adjusted Total Cerebellum Volume (cm^3)**	Intercept	144.47	142.99 – 145.95<br>[142.92 – 146.02]	< 2.00E-16***<br>[< 2.00E-16***]	
	Age (years)	-0.05	-0.11 – 0.01<br>[-0.11 – 0.01]	0.11<br>[0.11]	0.11
	Sex [Male]	5.37	4.37 – 6.37<br>[4.38 – 6.37]	< 2.00E-16***<br>[< 2.00E-16***]	1.00E-04***
	**Diagnostic Group [3q29Del]**	**-5.23**	**-9.64 – -0.83**<br>**[-9.55 – -0.92]**	**0.02***<br>**[0.02*]**	**0.02***
	R^2 / R^2 adjusted	0.07 / 0.07			
	Robust Wald test	39.1 on 3 and 1626 DF, p-value < 2.20E-16***			
	[F-statistic (OLS)	40.2 on 3 and 1626 DF, p-value < 2.20E-16***]			
**F. eICV-Adjusted Cerebellar Cortex Volume (cm^3)**	Intercept	119.84	118.55 – 121.13<br>[118.53 – 121.15]	< 2.00E-16***<br>[< 2.00E-16***]	
	Age (years)	-0.21	-0.26 – -0.16<br>[-0.26 – -0.16]	2.87E-15***<br>[2.70E-15***]	1.00E-04***
	Sex [Male]	4.88	4.04 – 5.72<br>[4.04 – 5.72]	< 2.00E-16***<br>[< 2.00E-16***]	1.00E-04***
	**Diagnostic Group [3q29Del]**	**-7.06**	**-10.78 – -3.35**<br>**[-10.72 – -3.41]**	**2.01E-04*****<br>**[1.56E-04***]**	**2.00E-04*** **
	R^2 / R^2 adjusted	0.11 / 0.11			
	Robust Wald test	66.6 on 3 and 1626 DF, p-value < 2.20E-16***			
	[F-statistic (OLS)	69.3 on 3 and 1626 DF, p-value < 2.20E-16***]			
**G. eICV-Adjusted Cerebellar White Matter Volume (cm^3)**	Intercept	22.12	21.12 – 23.12<br>[21.11 – 23.13]	< 2.00E-16***<br>[< 2.00E-16***]	
	Age (years)	0.44	0.33 – 0.55<br>[0.34 – 0.54]	1.09E-15***<br>[< 2.00E-16***]	1.00E-04***
	Age^2	-0.01	-0.01 – -0.004<br>[-0.01 – -0.004]	2.37E-07***<br>[8.06E-08***]	1.00E-04***
	Sex [Male]	0.39	0.11 – 0.68<br>[0.11 – 0.67]	6.57E-03**<br>[6.16E-03**]	4.20E-03**
	**Diagnostic Group [3q29Del]**	**2.13**	**-0.34 – 4.59**<br>**[0.91 – 3.34]**	**0.09†**<br>**[6.19E-04***]**	**1.10E-03** **
	R^2 / R^2 adjusted	0.19 / 0.19			
	Robust Wald test	111.6 on 4 and 1625 DF, p-value < 2.20E-16***			
	[F-statistic (OLS)	94.1 on 4 and 1625 DF, p-value < 2.20E-16***]			

Control *N* = 1,608, 3q29Del *N* = 22. p-value ≤ 0.001 '***', p-value ≤ 0.01 '**', p-value ≤ 0.05 '*', p-value ≤ 0.1 '†'. *Abbreviations*: 3q29 deletion syndrome, 3q29Del; VOI, volumetric measure of interest; eICV, estimated total intracranial volume; unstandardized coefficient estimate, *b*; confidence interval, CI; degrees of freedom, DF; permutation, perm; ordinary least squares, OLS.

VOI	EDF	p-value		
		s(Age) (years)	Sex [Male]	Diagnostic Group [3q29Del]
**Total Cerebellum Volume (cm^3)**				
**Absolute Volume**	5.73	1.47E-06***	< 2.00E-16*** (male > female)	**3.47E-07*** (3q29Del < control)**
**eICV-Adjusted Volume**	4.35	0.09†	< 2.00E-16*** (male > female)	**0.05***
**Cerebellar Cortex Volume (cm^3)**				
**Absolute Volume**	5.98	< 2.00E-16***	< 2.00E-16*** (male > female)	**5.32E-10*** (3q29Del < control)**
**eICV-Adjusted Volume**	5.36	< 2.00E-16***	< 2.00E-16*** (male > female)	**4.67E-04*** (3q29Del < control)**
**Cerebellar White Matter Volume (cm^3)**				
**Absolute Volume**	5.26	< 2.00E-16***	< 2.00E-16*** (male > female)	**0.89**
**eICV-Adjusted Volume**	3.99	< 2.00E-16***	4.87E-03** (male > female)	**2.52E-05*** (3q29Del > control)**
**eICV (cm^3)**	5.00	5.05E-06***	< 2.00E-16*** (male > female)	**5.01E-10*** (3q29Del < control)**

Table S5. Summary of supplemental results from penalized cubic spline models testing the effect of diagnostic group on tissue-specific cerebellar volumes and eICV. To more flexibly account for linear and non-linear trajectories of volumetric change across age without requiring *a priori* selection of candidate models, we fit generalized additive models (GAM) with a cubic spline basis to our data as a supplemental method. Volumes reflect FreeSurfer-based morphometrics. Smoothing parameters were selected by the restricted maximum likelihood (REML) approach. EDF represents how complex the developmental pattern of the estimated volumetric trajectory is across age. EDF = 1 is equivalent to a straight line, EDF = 2 is equivalent to a quadratic curve, etc., with higher EDFs describing increased wiggliness. s(Age) represents the spline term of age. The main effect of diagnostic group is reported in bold for clarity. The p-values for sex and diagnostic group are derived from standard t-tests of whether the indicator of sex/diagnostic group are significant in determining each VOI. The p-values for s(Age) are derived from an approximate F-test of whether the smooth term of age is significant in the penalized cubic spline models for each VOI. Contrast coding: reference levels for the diagnostic group and sex variables are neurotypical control and female, respectively. For categorical variables, the direction of the corresponding effect in significant tests (*p's* ≤ 0.05) is specified by the ">" (greater than) or "<" (less than) symbols. Control *N* = 1,608**,** 3q29Del *N* = 23. p-value ≤ 0.001 '***', p-value ≤ 0.01 '**', p-value ≤ 0.05 '*', p-value ≤ 0.1 '†'. *Abbreviations*: 3q29 deletion syndrome, 3q29Del; estimated total intracranial volume, eICV; volumetric measure of interest, VOI; effective degrees of freedom, EDF.

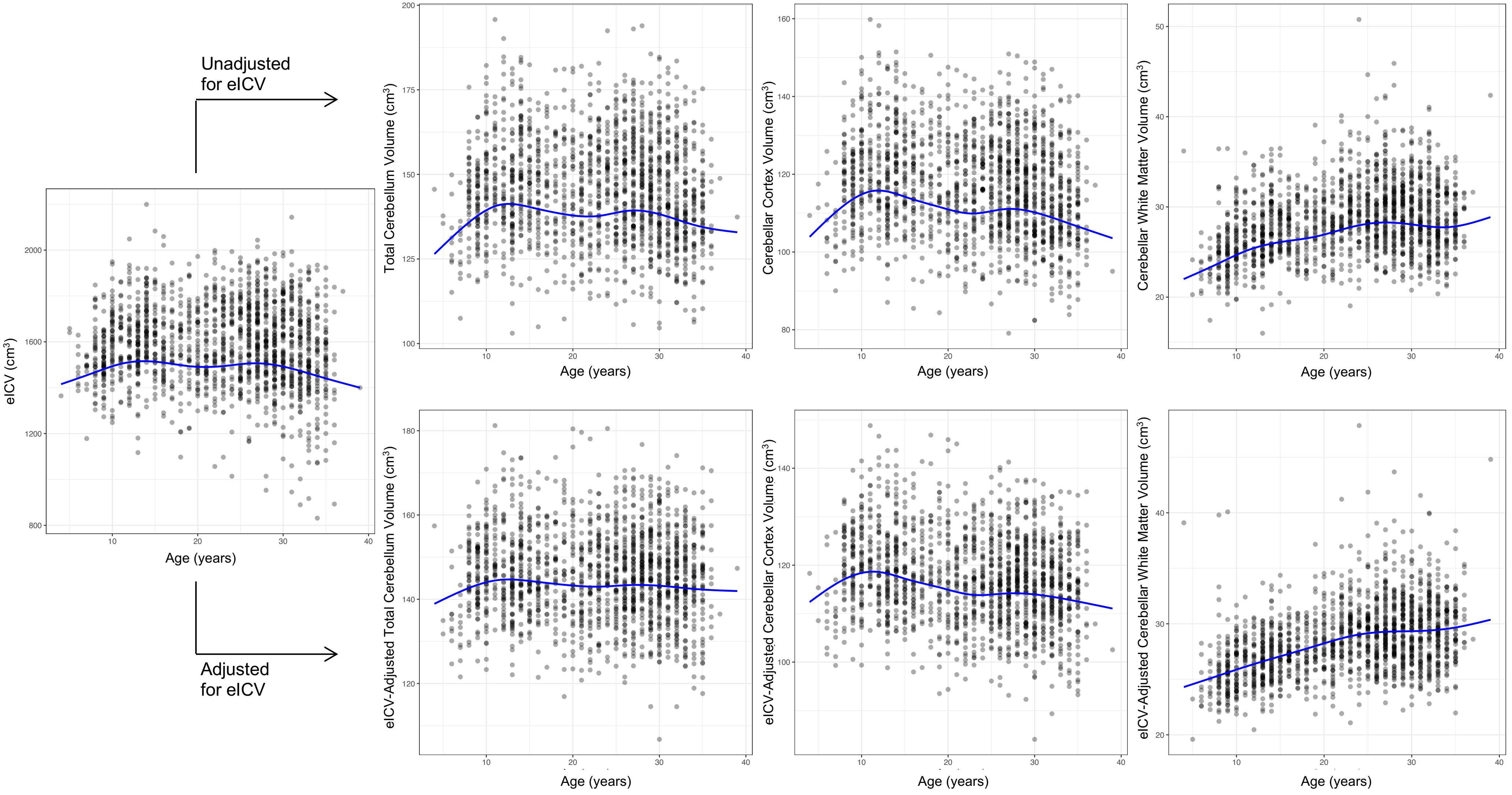

Fig. S9. Developmental trajectories for tissue-specific cerebellar volumes and eICV, estimated by the penalized cubic spline approach. Volumes reflect FreeSurfer-based morphometrics. Each dot represents volumetric data for a single study participant. Blue solid lines represent the estimated mean volumes. An indicator of sex (male / female), an indicator of diagnostic group (3q29Del / neurotypical control) and a spline term of age were included in each generalized additive model (GAM). Control *N* = 1,608**,** 3q29Del *N* = 23. *Abbreviations*: 3q29 deletion syndrome, 3q29Del; estimated total intracranial volume, eICV.

	Age- & sex-matched subsample *case:control ratio = 1:4*		
	**Control** *N* = 92	**3q29Del** *N* = 23	**Test statistics**
**Sex**, *n/N (%)*			
Male<br>Female	56 (60.87%)<br>36 (39.13%)	14 (60.87%)<br>9 (39.13%)	OR = 1,<br>p-value[a] = 1.00
**Age** (in years)			
Mean ± SD<br>Median [Range]	15.17 ± 8.55<br>14.00 [5 – 37]	15.09 ± 9.22<br>14.00 [4 – 39]	W = 1031.50<br>p-value[b] = 0.86
**Total Cerebellum Volume (cm^3)**			
**Absolute Volume**<br>Mean ± SD<br>Median [Range]	147.30 ± 15.13<br>147.52 [107.18 – 190.15]	132.74 ± 12.81<br>134.43 [105.61 – 153.09]	*Cohen's d* = -0.99 *(large ES)*<br>*95% CI = -1.41 – -0.58*<br>p-value[c] = 4.50E-05***
**eICV-Adjusted Volume**<br>Mean ± SD<br>Median [Range]	147.79 ± 10.87<br>147.66 [121.58 – 174.78]	141.97 ± 11.31<br>142.12 [120.79 – 162.13]	*Cohen's d* = -0.53 *(moderate ES)*<br>*95% CI = -0.99 – -0.07*<br>p-value[c] = 0.02*
**Cerebellar Cortex Volume (cm^3)**			
**Absolute Volume**<br>Mean ± SD<br>Median [Range]	120.69 ± 12.57<br>120.29 [85.77 – 158.22]	105.34 ± 11.55<br>102.85 [79.11 – 125.88]	*Cohen's d* = -1.24 *(large ES)*<br>*95% CI = -1.69 – -0.76*<br>p-value[c] = 5.39E-07***
**eICV-Adjusted Volume**<br>Mean ± SD<br>Median [Range]	121.05 ± 9.30<br>120.59 [99.31 – 146.62]	112.31 ± 9.82<br>111.51 [93.13 – 133.41]	*Cohen's d* = -0.93 *(large ES)*<br>*95% CI = -1.38 – -0.38*<br>p-value[c] = 1.19E-04***
**Cerebellar WM Volume (cm^3)**			
**Absolute Volume**<br>Mean ± SD<br>Median [Range]	26.61 ± 3.61<br>25.64 [19.74 – 36.22]	27.40 ± 5.84<br>26.13 [20.42 – 42.37]	*Cohen's d* = 0.19 *(negligible ES)*<br>*95% CI = -0.43 – 0.75*<br>p-value[c] = 0.42
**eICV-Adjusted Volume**<br>Mean ± SD<br>Median [Range]	26.73 ± 3.02<br>26.74 [19.59 – 35.81]	29.66 ± 6.11<br>28.04 [23.21 – 44.81]	*Cohen's d* = 0.77 *(moderate ES)*<br>*95% CI = 0.16 – 1.42*<br>p-value[c] = 1.34E-03**
**eICV (cm^3)**			
Mean ± SD<br>Median [Range]	1578.28 ± 156.63<br>1572.27 [831.21 – 1877.51]	1413.34 ± 116.47<br>1392.33 [1236.90 – 1629.01]	*Cohen's d* = -1.10 *(large ES)*<br>*95% CI = -1.54 – -0.58*<br>p-value[c] = 6.60E-06***

Table S6. Supplemental age- and sex-matched analyses testing for diagnostic group differences in tissue-specific cerebellar volumes and eICV. [a]Fisher's exact test, [b]Wilcoxon rank sum test, [c]Student's two sample t-test. Diagnostic group comparisons on tissue-specific volumes derived with FreeSurfer were repeated using an age- and sex-matched cohort drawn from the larger HCP sample to minimize the influence of demographic confounds. Controls were randomly selected from the larger HCP sample based on exact match for sex and nearest available match for age, with a 1:4 case:control ratio and without replacement. Lower and upper bounds of the Cohen's *d* effect sizes (95% confidence interval) were computed with the bias-corrected and accelerated (BCa) bootstrap method using 1,000 replications. Results from this matched case-control analysis are consistent with the main analyses reported in Table S3 using the full study sample. p-value ≤ 0.001 '***', p-value ≤ 0.01 '**', p-value ≤ 0.05 '*', p-value ≤ 0.1 '†'. Quantification of the effect size magnitude is performed using the thresholds defined in Cohen (1992). |d| < 0.2 "negligible", |d| < 0.5 "small", |d| < 0.8 "medium", otherwise "large". *Abbreviations*: 3q29 deletion syndrome, 3q29Del; Human Connectome Project, HCP; estimated total intracranial volume, eICV*;* white matter, WM; odds ratio, OR; effect size, ES.

Cohen J (1992): A power primer. *Psychological Bulletin*. 112, 155-159.

Table S7. Exploratory modeling of diagnostic group by sex interaction effects on tissue-specific cerebellar volumes and eICV. A-G) Volumes reflect FreeSurfer-based morphometrics. Interaction effects are reported in bold for clarity. Covariates included in each linear regression model reflect the best-fitting models from Table S3. Final inferences are based on heteroskedasticity-robust estimates, which are provided above non-robust OLS estimates (in grey brackets), along with robust Wald statistics for each model. We also report exact p-values calculated by non-asymptotic permutation marginal tests. Contrast coding: reference levels for diagnostic group and sex are neurotypical control and female, respectively.

VOI	Explanatory variables	*b*	CI (95%)	p-value	perm. p-value
**A. Absolute Total Cerebellum Volume (cm^3)**	Intercept	133.53	128.99 – 138.08 [129.08 – 137.99]	< 2.00E-16*** [< 2.00E-16***]	
	Age (years)	0.67	0.21 – 1.13 [0.22 – 1.13]	4.18E-03** [3.96E-03**]	3.10E-03**
	Age^2	-0.02	-0.03 – -0.01 [-0.03 – -0.01]	7.13E-04*** [8.03E-04***]	1.10E-03**
	Sex [Male]	16.28	15.01 – 17.55 [15.03 – 17.53]	< 2.00E-16*** [< 2.00E-16***]	1.00E-04***
	Diagnostic Group [3q29Del]	-12.95	-21.59 – -4.32 [-21.27 – -4.63]	3.30E-03** [2.29E-03**]	2.20E-03**
	**Diagnostic Group x Sex**	**-3.82**	**-13.91 – 6.27 [-14.48 – 6.84]**	**0.46 [0.48]**	**0.48**
	R^2 / R^2 adjusted	0.31 / 0.31			
	Robust Wald test	148.1 on 5 and 1625 DF, p-value < 2.20E-16***			
	[F-statistic (OLS)	148.3 on 5 and 1625 DF, p-value < 2.20E-16***]			
**B. Absolute Cerebellar Cortex Volume (cm^3)**	Intercept	113.56	109.77 – 117.35 [109.90 – 117.22]	< 2.00E-16*** [< 2.00E-16***]	
	Age (years)	0.12	-0.26 – 0.50 [-0.26 – 0.49]	0.54 [0.53]	0.52
	Age^2	-0.01	-0.02 – 0.0001 [-0.02 – 0.0001]	0.06† [0.06†]	0.06†
	Sex [Male]	13.20	12.16 – 14.24 [12.17 – 14.22]	< 2.00E-16*** [< 2.00E-16***]	1.00E-04***
	Diagnostic Group [3q29Del]	-12.59	-19.84 – -5.34 [-19.42 – -5.76]	6.71E-04*** [3.06E-04***]	2.00E-04***
	**Diagnostic Group x Sex**	**-4.61**	**-13.25 – 4.03 [-13.36 – 4.14]**	**0.30 [0.30]**	**0.30**
	R^2 / R^2 adjusted	0.32 / 0.32			
	Robust Wald test	155.6 on 5 and 1625 DF, p-value < 2.20E-16***			
	[F-statistic (OLS)	156.0 on 5 and 1625 DF, p-value < 2.20E-16***]			
**C. Absolute Cerebellar White Matter Volume (cm^3)**	Intercept	19.98	18.82 – 21.14 [18.80 – 21.16]	< 2.00E-16*** [< 2.00E-16***]	
	Age (years)	0.55	0.43 –0.67 [0.43 – 0.67]	< 2.00E-16*** [< 2.00E-16***]	1.00E-04***
	Age^2	-0.01	-0.01 – -0.01 [-0.01 – -0.01]	5.63E-11*** [4.53E-11***]	1.00E-04***
	Sex [Male]	3.08	2.75 – 3.41 [2.75 – 3.41]	< 2.00E-16*** [< 2.00E-16***]	1.00E-04***
	Diagnostic Group [3q29Del]	-0.36	-3.85 – 3.13 [-2.57 – 1.84]	0.84 [0.75]	0.75
	**Diagnostic Group x Sex**	**0.79**	**-4.17 – 5.74 [-2.04 – 3.61]**	**0.76 [0.58]**	**0.58**
	R^2 / R^2 adjusted	0.28 / 0.27			
	Robust Wald test	121.5 on 5 and 1625 DF, p-value < 2.20E-16***			
	[F-statistic (OLS)	124.1 on 5 and 1625 DF, p-value < 2.20E-16***]			

**D. Estimated Total Intracranial Volume (eICV) (cm^3)**	Intercept	1416.29	1369.41 – 1463.16] [1366.23 – 1466.34]	< 2.00E-16*** [< 2.00E-16***]	
	Age (years)	9.66	4.64 – 14.69 [4.53 – 14.79]	1.69E-04*** [2.29E-04***]	3.00E-04***
	Age^2	-0.25	-0.37 – -0.13 [-0.37 – -0.13]	4.86E-05*** [3.92E-05***]	1.00E-04***
	Sex [Male]	208.09	194.04 – 222.14 [194.05 – 222.13]	< 2.00E-16*** [< 2.00E-16***]	1.00E-04***
	Diagnostic Group [3q29Del]	-107.56	-191.51 – -23.61 [-201.01 – -14.11]	0.01** [0.02*]	0.03*
	**Diagnostic Group x Sex**	**-149.64**	**-249.96 – -49.33 [-269.37 – -29.91]**	**3.48E-03** [0.01**]**	**0.01****
	R^2 / R^2 adjusted	0.37 / 0.37			
	Robust Wald test	192.4 on 5 and 1625 DF, p-value < 2.20E-16***			
	[F-statistic (OLS)	191.3 on 5 and 1625 DF, p-value < 2.20E-16***]			
**E. eICV-Adjusted Total Cerebellum Volume (cm^3)**	Intercept	144.46	142.99 – 145.94 [142.92 – 146.00]	< 2.00E-16*** [< 2.00E-16***]	
	Age (years)	-0.05	-0.11 – 0.01 [-0.11 – 0.01]	0.12 [0.12]	0.12
	Sex [Male]	5.33	4.32 – 6.33 [4.33 – 6.32]	< 2.00E-16*** [< 2.00E-16***]	1.00E-04***
	Diagnostic Group [3q29Del]	-7.41	-13.80 – -1.02 [-14.11 – -0.72]	0.02* [0.03*]	0.03*
	**Diagnostic Group x Sex**	**3.94**	**-4.41 – 12.30 [-4.64 – 12.53]**	**0.35 [0.37]**	**0.37**
	R^2 / R^2 adjusted	0.07 / 0.07			
	Robust Wald test	29.6 on 4 and 1626 DF, p-value < 2.20E-16***			
	[F-statistic (OLS)	30.3 on 4 and 1626 DF, p-value < 2.20E-16***]			
**F. eICV-Adjusted Cerebellar Cortex Volume (cm^3)**	Intercept	119.89	118.60 – 121.17 [118.58 – 121.19]	< 2.00E-16*** [< 2.00E-16***]	
	Age (years)	-0.21	-0.26 – -0.16 [-0.26 – -0.16]	1.48E-15*** [1.50E-15***]	1.00E-04***
	Sex [Male]	4.85	4.00 – 5.70 [4.01 – 5.70]	< 2.00E-16*** [< 2.00E-16***]	1.00E-04***
	Diagnostic Group [3q29Del]	-8.24	-13.39 – -3.10 [-13.91 – -2.57]	1.71E-03** [4.42E-03**]	4.30E-03**
	**Diagnostic Group x Sex**	**1.43**	**-5.62 – 8.48 [-5.84 – 8.70]**	**0.69 [0.70]**	**0.70**
	R^2 / R^2 adjusted	0.11 / 0.11			
	Robust Wald test	51.0 on 4 and 1626 DF, p-value < 2.20E-16***			
	[F-statistic (OLS)	52.6 on 4 and 1626 DF, p-value < 2.20E-16***]			
**G. eICV-Adjusted Cerebellar White Matter Volume (cm^3)**	Intercept	22.20	21.21 – 23.19 [21.19 – 23.21]	< 2.00E-16*** [< 2.00E-16***]	
	Age (years)	0.43	0.32 – 0.53 [0.32 – 0.53]	8.45E-15*** [1.32E-15***]	1.00E-04***
	Age^2	-0.01	-0.01 – -0.004 [-0.01 – -0.004]	1.55E-06*** [4.10E-07***]	1.00E-04***
	Sex [Male]	0.38	0.10 – 0.65 [0.09 – 0.66]	8.15E-03** [9.49E-03**]	9.30E-03**
	Diagnostic Group [3q29Del]	1.04	-2.56 – 4.64 [-0.85 – 2.93]	0.57 [0.28]	0.27
	**Diagnostic Group x Sex**	**2.73**	**-2.29 – 7.75 [0.31 – 5.15]**	**0.29 [0.03*]**	**0.03***
	R^2 / R^2 adjusted	0.19 / 0.19			
	Robust Wald test	87.5 on 5 and 1625 DF, p-value < 2.20E-16***			
	[F-statistic (OLS)	78.2 on 5 and 1625 DF, p-value < 2.20E-16***]			

Control *N* = 1,608 (Female *N* = 861, Male *N* = 747), 3q29Del *N* = 23 (Female *N* = 9, Male *N* = 14). p-value ≤ 0.001 '***', p-value ≤ 0.01 '**', p-value ≤ 0.05 '*', p-value ≤ 0.1 '†'. *Abbreviations*: 3q29 deletion syndrome, 3q29Del; VOI, volumetric measure of interest; eICV, estimated total intracranial volume; unstandardized coefficient estimate, *b*; confidence interval, CI; degrees of freedom, DF; permutation, perm; ordinary least squares, OLS.

Fig. S10. Predictor effect plots visualizing results from sex by diagnostic group interaction models. A) Predicted values of eICV across male versus female 3q29Del and control groups were computed from the exploratory interaction model reported in Table S7D, while covariates (age, age2) were held fixed. Heteroskedasticity-robust regression results indicate a significant diagnostic group by sex interaction effect on eICV (p ≤ 0.05). eICV was calculated by FreeSurfer's atlas-based spatial normalization procedure. **B)** Predicted values of eICV-adjusted cerebellar white matter volume across male versus female 3q29Del and control groups were computed from the exploratory interaction model reported in Table S7G, while covariates (age, age^2) were held fixed. Non-robust OLS estimates, and permutation testing from Table S7G suggested a diagnostic group by sex interaction effect on eICV-adjusted cerebellar white matter volumes (non-robust $p \leq 0.05$, permutation $p \leq 0.05$), however this effect was not significant ($p > 0.05$) when robust standard error estimates were calculated to account for the heteroskedasticity in the data. We provide a graphic illustration of this finding for visual inspection of underlying trends, but we consider the evidence in favor of this sex-specific effect to be weaker. Error bars indicate the 95% confidence interval. Control *N* = 1,608 (Female *N* = 861, Male *N* = 747), 3q29Del *N* = 23 (Female *N* = 9, Male *N* = 14). p-value ≤ 0.001 '***', p-value ≤ 0.01 '**', p-value ≤ 0.05 '*', p-value ≤ 0.1 *'†'* *Abbreviations*: 3q29 deletion syndrome, 3q29Del; estimated total intracranial volume, eICV; ordinary least squares, OLS.

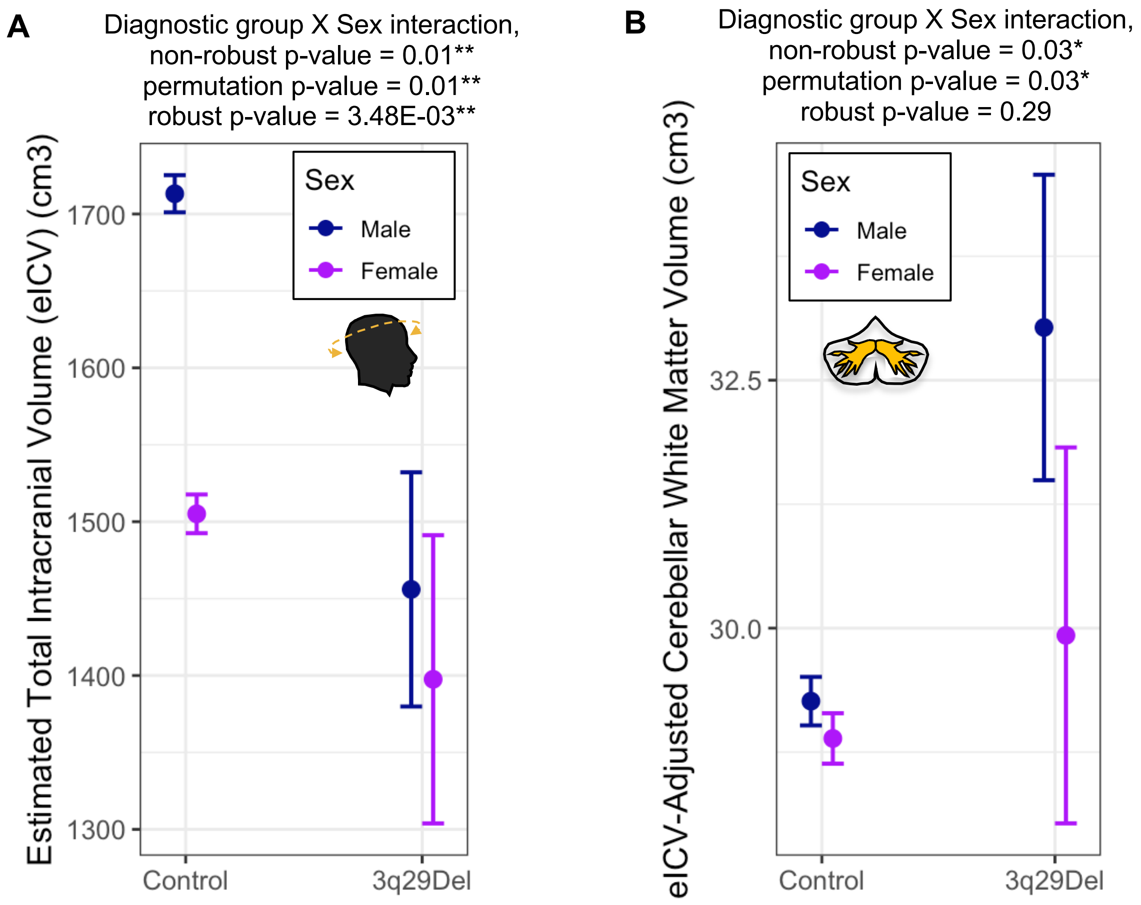

Outcome variable	Explanatory variables	*b*	CI (95%)	p-value	perm. p-value
**A. Estimated Total Intracranial Volume (eICV) (cm^3)**<br><br>**Sex: Male**	Intercept	1573.56	1502.04 – 1645.07<br>[1497.94 – 1649.18]	< 2.00E-16***<br>[< 2.00E-16***]	
	Age (years)	11.48	3.99 – 18.98<br>[3.83 – 19.14]	2.72E-03**<br>[3.34E-03**]	2.60E-03**
	Age^2	-0.23	-0.41 – -0.05<br>[-0.41 – -0.05]	0.01**<br>[0.01**]	0.01**
	**Diagnostic group [3q29Del]**	**-237.09**	**-293.81 – -180.36**<br>**[-312.81 – -161.36]**	**9.87E-16******<br>**[1.28E-09******]**	**1.00E-04*****
	R^2 / R^2 adjusted	0.08 / 0.07			
	Robust Wald test	32.3 on 3 and 757 DF, p-value < 2.00E-16***			
	[F-statistic (OLS)	20.97 on 3 and 757 DF, p-value = 4.67E-13***]			
**Estimated Total Intracranial Volume (eICV) (cm^3)**<br><br>**Sex: Female**	Intercept	1463.47	1403.35 – 1523.58<br>[396.83 – 1530.11]	< 2.00E-16***<br>[< 2.00E-16***]	
	Age (years)	7.28	0.73 – 13.83<br>[0.44 – 14.12]	0.03*<br>[0.04*]	0.03*
	Age^2	-0.24	-0.39 – -0.08<br>[-0.39 – -0.08]	3.14E-03**<br>[3.22E-03**]	3.00E-03**
	**Diagnostic group [3q29Del]**	**-119.89**	**-198.88 – -40.89**<br>**[-213.38 – -26.39]**	**2.98E-03****<br>**[0.01**]**	**0.01****
	R^2 / R^2 adjusted	0.05 / 0.04			
	Robust Wald test	13.00 on 3 and 866 DF, p-value = 2.61E-08***			
	[F-statistic (OLS)	13.62 on 3 and 866 DF, p-value = 1.09E-08]			
**B. eICV-Adjusted Cerebellar White Matter Volume (cm^3)**<br><br>**Sex: Male**	Intercept	21.14	19.36 – 22.93<br>[19.41 – 22.88]	< 2.00E-16***<br>[< 2.00E-16***]	
	Age (years)	0.56	0.36 – 0.75<br>[0.38 – 0.73]	1.94E-08***<br>[8.15E-10***]	1.00E-04***
	Age^2	-0.01	-0.01 – -0.005<br>[-0.01 – -0.004]	1.74E-04***<br>[2.48E-05***]	1.00E-04***
	**Diagnostic group [3q29Del]**	**4.09**	**0.50 – 7.68**<br>**[2.35 – 5.83]**	**0.03***<br>**[4.59E-06***]**	**1.00E-04*****
	R^2 / R^2 adjusted	0.18 / 0.18			
	Robust Wald test	62.1 on 3 and 757 DF, p-value < 2.00E-16***			
	[F-statistic (OLS)	57.0 on 3 and 757 DF, p-value < 2.00E-16***]			
**eICV-Adjusted Cerebellar White Matter Volume (cm^3)**<br><br>**Sex: Female**	Intercept	23.35	22.27 – 24.43<br>[22.16 – 24.54]	< 2.00E-16***<br>[< 2.00E-16***]	
	Age (years)	0.32	0.20 – 0.44<br>[0.20 – 0.44]	9.95E-08***<br>[3.16E-07***]	1.00E-04***
	Age^2	-0.004	-0.01 – -0.001<br>[-0.01 – -0.001]	4.24E-03**<br>[4.83E-03**]	4.90E-03**
	**Diagnostic group [3q29Del]**	**0.88**	**-2.64 – 4.39**<br>**[-0.79 – 2.54]**	**0.62**<br>**[0.30]**	**0.29**
	R^2 / R^2 adjusted	0.21 / 0.20			
	Robust Wald test	89.4 on 3 and 866 DF, p-value < 2.00E-16***			
	[F-statistic (OLS)	75.5 on 3 and 866 DF, p-value < 2.00E-16***]			

Table S8. *Post hoc* analysis of sex by diagnostic group interaction effects on eICV and eICV-adjusted cerebellar white matter volumes. For further inspection of diagnostic group by sex interaction effects reported in Table S7D, here we report results from *post hoc* analyses stratifying the sample into males and females, separately. Main effect of diagnostic group is highlighted in bold for clarity. **A)** Both male and female 3q29Del participants have smaller eICVs than controls (*p's* ≤ 0.01), however this reduction is greater among male 3q29Del participants than females. **B)** Male 3q29Del participants have larger eICV-adjusted cerebellar white matter volumes than male controls ($p \leq 0.05$), whereas this effect is not significant in females ($p > 0.05$). Final inferences are based on heteroskedasticity-robust estimates provided above non-robust OLS estimates (in grey brackets). Robust Wald test statistics are reported to assess the overall significance of each model, along with exact p-values. Male *N* = 761 (Control *N* = 747, 3q29Del *N* = 14), Female *N* = 870 (Control *N* = 861, 3q29Del *N* = 9). Contrast coding: reference level for diagnostic group is neurotypical control. p-value ≤ 0.001 '***', p-value ≤ 0.01 '**', p-value ≤ 0.05 '*', p-value ≤ 0.1 '†'. *Abbreviations*: 3q29 deletion syndrome, 3q29Del; estimated total intracranial volume, eICV; unstandardized coefficient estimate, *b*; confidence interval, CI; degrees of freedom, DF; permutation, perm.

Table S9. Exploratory modeling of diagnostic group by age interaction effects on tissue-specific cerebellar volumes and eICV. A-G) Volumes reflect FreeSurfer-based morphometrics. Interrogated interactions are reported in bold for clarity. Covariates included in each linear regression model reflect the best-fitting models from Table S3. Final inferences are based on heteroskedasticity-robust estimates provided above non-robust OLS estimates (in grey brackets), along with robust Wald statistics for each model. We also report exact p-values calculated by non-asymptotic permutation marginal tests. Contrast coding: reference levels for the diagnostic group and sex variables are neurotypical control and female, respectively.

VOI	Explanatory variables	*b*	CI (95%)	p-value	perm. p-value
**A. Absolute Total Cerebellum Volume (cm^3)**	Intercept	133.22	128.67 – 137.78<br>[128.75 – 137.70]	< 2.00E-16***<br>[< 2.00E-16***]	
	Age (years)	0.70	0.24 – 1.16<br>[0.24 – 1.16]	2.84E-03**<br>[2.74E-03**]	2.60E-03**
	Age^2	-0.02	-0.03 – -0.01<br>[-0.03 – -0.01]	4.97E-04***<br>[5.75E-04***]	7.00E-04***
	Sex [Male]	16.21	14.95 – 17.47<br>[14.97 – 17.45]	< 2.00E-16***<br>[< 2.00E-16***]	1.00E-04***
	Diagnostic Group [3q29Del]	-9.84	-19.10 – -0.59<br>[-20.13 – 0.44]	0.04*<br>[0.06†]	0.06†
	**Diagnostic Group x Age**	**-0.35**	**-0.86 – 0.15**<br>**[-0.93 – 0.22]**	**0.17**<br>**[0.23]**	**0.23**
	R^2 / R^2 adjusted	0.31 / 0.31			
	Robust Wald test	150.9 on 5 and 1625 DF, p-value < 2.20E-16***			
	[F-statistic (OLS)	148.6 on 5 and 1625 DF, p-value < 2.20E-16***]			
**B. Absolute Cerebellar Cortex Volume (cm^3)**	Intercept	113.37	109.59 – 117.15<br>[109.69 – 117.04]	< 2.00E-16***<br>[< 2.00E-16***]	
	Age (years)	0.14	-0.24 – 0.52<br>[-0.24 – 0.51]	0.48<br>[0.47]	0.46
	Age^2	-0.01	-0.02 – 0.0003<br>[-0.02 – -0.0003]	0.04*<br>[0.04*]	0.04*
	Sex [Male]	13.13	12.09 – 14.16<br>[12.11 – 14.14]	< 2.00E-16***<br>[< 2.00E-16***]	1.00E-04***
	Diagnostic Group [3q29Del]	-12.42	-20.50 – -4.35<br>[-20.87 – -3.98]	2.59E-03**<br>[3.96E-03**]	3.00E-03**
	**Diagnostic Group x Age**	**-0.19**	**-0.59 – 0.20**<br>**[-0.67 – 0.28]**	**0.34**<br>**[0.42]**	**0.42**
	R^2 / R^2 adjusted	0.32 / 0.32			
	Robust Wald test	159.7 on 5 and 1625 DF, p-value < 2.20E-16***			
	[F-statistic (OLS)	155.9 on 5 and 1625 DF, p-value < 2.20E-16***]			
**C. Absolute Cerebellar White Matter Volume (cm^3)**	Intercept	19.86	18.61 – 21.10<br>[18.67 – 21.04]	< 2.00E-16***<br>[< 2.00E-16***]	
	Age (years)	0.56	0.43 – 0.69<br>[0.44 – 0.68]	< 2.00E-16***<br>[< 2.00E-16***]	1.00E-04***
	Age^2	-0.01	-0.01 – -0.01<br>[-0.01 – -0.01]	7.32E-10***<br>[2.27E-11***]	1.00E-04***
	Sex [Male]	3.09	2.75 – 3.42<br>[2.76 – 3.41]	< 2.00E-16***<br>[< 2.00E-16***]	1.00E-04***
	Diagnostic Group [3q29Del]	2.58	-3.70 – 8.85<br>[-0.15 – 5.30]	0.42<br>[0.06†]	0.06†
	**Diagnostic Group x Age**	**-0.16**	**-0.60 – 0.28**<br>**[-0.31 – -0.01]**	**0.47**<br>**[0.04*]**	**0.04***
	R^2 / R^2 adjusted	0.28 / 0.28			
	Robust Wald test	125.9 on 5 and 1625 DF, p-value < 2.20E-16***			
	[F-statistic (OLS)	125.2 on 5 and 1625 DF, p-value < 2.20E-16***]			

**D. Estimated Total Intracranial Volume (eICV) (cm^3)**	Intercept	1413.37	1366.47 – 1460.28 [1362.98 – 1463.77]	< 2.00E-16*** [< 2.00E-16***]	
	Age (years)	10.02	5.00 – 15.04 [4.87 – 15.17]	9.50E-05*** [1.42E-04***]	2.00E-04***
	Age^2	-0.26	-0.38 – -0.14 [-0.37 – -0.14]	2.90E-05*** [2.45E-05***]	1.00E-04***
	Sex [Male]	205.92	191.98 – 219.86 [191.95 – 219.89]	< 2.00E-16*** [< 2.00E-16***]	1.00E-04***
	Diagnostic Group [3q29Del]	-166.04	-278.61 – -53.47 [-281.82 – -50.26]	3.87E-03** [4.97E-03**]	5.40E-03**
	**Diagnostic Group x Age**	**-2.09**	**-6.93 – 2.74** **[-8.60 – 4.42]**	**0.40** **[0.53]**	**0.53**
	R^2 / R^2 adjusted	0.37 / 0.37			
	Robust Wald test	194.5 on 5 and 1625 DF, p-value < 2.20E-16***			
	[F-statistic (OLS)	189.5 on 5 and 1625 DF, p-value < 2.20E-16***]			
**E. eICV-Adjusted Total Cerebellum Volume (cm^3)**	Intercept	144.36	142.87 – 145.85 [142.81 – 145.91]	< 2.00E-16*** [< 2.00E-16***]	
	Age (years)	-0.04	-0.10 – 0.02 [-0.10 – 0.02]	0.15 [0.16]	0.16
	Sex [Male]	5.37	4.38 – 6.37 [4.38 – 6.37]	< 2.00E-16*** [< 2.00E-16***]	1.00E-04***
	Diagnostic Group [3q29Del]	-1.56	-9.21 – 6.09 [-9.80 – 6.68]	0.69 [0.71]	0.72
	**Diagnostic Group x Age**	**-0.23**	**-0.69 – 0.23** **[-0.69 – 0.24]**	**0.33** **[0.34]**	**0.33**
	R^2 / R^2 adjusted	0.07 / 0.07			
	Robust Wald test	29.7 on 4 and 1626 DF, p-value < 2.20E-16***			
	[F-statistic (OLS)	30.3 on 4 and 1626 DF, p-value < 2.20E-16***]			
**F. eICV-Adjusted Cerebellar Cortex Volume (cm^3)**	Intercept	119.84	118.55 – 121.13 [118.52 – 121.15]	< 2.00E-16*** [< 2.00E-16***]	
	Age (years)	-0.21	-0.26 – -0.16 [-0.26 – -0.16]	4.27E-15*** [4.34E-15***]	1.00E-04***
	Sex [Male]	4.87	4.03 – 5.71 [4.03 – 5.71]	< 2.00E-16*** [< 2.00E-16***]	1.00E-04***
	Diagnostic Group [3q29Del]	-5.64	-11.69 – 0.40 [-12.63 – 1.34]	0.07† [0.10†]	0.10†
	**Diagnostic Group x Age**	**-0.11**	**-0.47 – 0.24** **[-0.51 – 0.28]**	**0.53** **[0.57]**	**0.57**
	R^2 / R^2 adjusted	0.11 / 0.11			
	Robust Wald test	51.0 on 4 and 1626 DF, p-value < 2.20E-16***			
	[F-statistic (OLS)	52.6 on 4 and 1626 DF, p-value < 2.20E-16***]			
**G. eICV-Adjusted Cerebellar White Matter Volume (cm^3)**	Intercept	22.12	21.04 – 23.20 [21.10 – 23.14]	< 2.00E-16*** [< 2.00E-16***]	
	Age (years)	0.43	0.31 – 0.55 [0.33 – 0.54]	1.31E-12*** [8.34E-16***]	1.00E-04***
	Age^2	-0.01	-0.01 – -0.003 [-0.01 – -0.004]	1.08E-05*** [8.34E-16***]	1.00E-04***
	Sex [Male]	0.41	0.12 – 0.70 [0.13 – 0.69]	5.42E-03** [4.51E-03**]	4.90-03**
	Diagnostic Group [3q29Del]	4.74	-1.78 – 11.25 [2.40 – 7.07]	0.15 [7.48E-05***]	2.00E-04***
	**Diagnostic Group x Sex**	**-0.13**	**-0.58 – 0.31** **[-0.27 – -0.002]**	**0.55** **[0.05*]**	**0.05***
	R^2 / R^2 adjusted	0.19 / 0.19			
	Robust Wald test	99.6 on 5 and 1625 DF, p-value < 2.20E-16***			
	[F-statistic (OLS)	77.9 on 5 and 1625 DF, p-value < 2.20E-16***]			

Control *N* = 1,608, 3q29Del *N* = 23. p-value ≤ 0.001 '***', p-value ≤ 0.01 '**', p-value ≤ 0.05 '*', p-value ≤ 0.1 '†'. *Abbreviations*: 3q29 deletion syndrome, 3q29Del; VOI, volumetric measure of interest; eICV, estimated total intracranial volume; unstandardized coefficient estimate, *b*; confidence interval, CI; degrees of freedom, DF; permutation, perm; ordinary least squares, OLS.

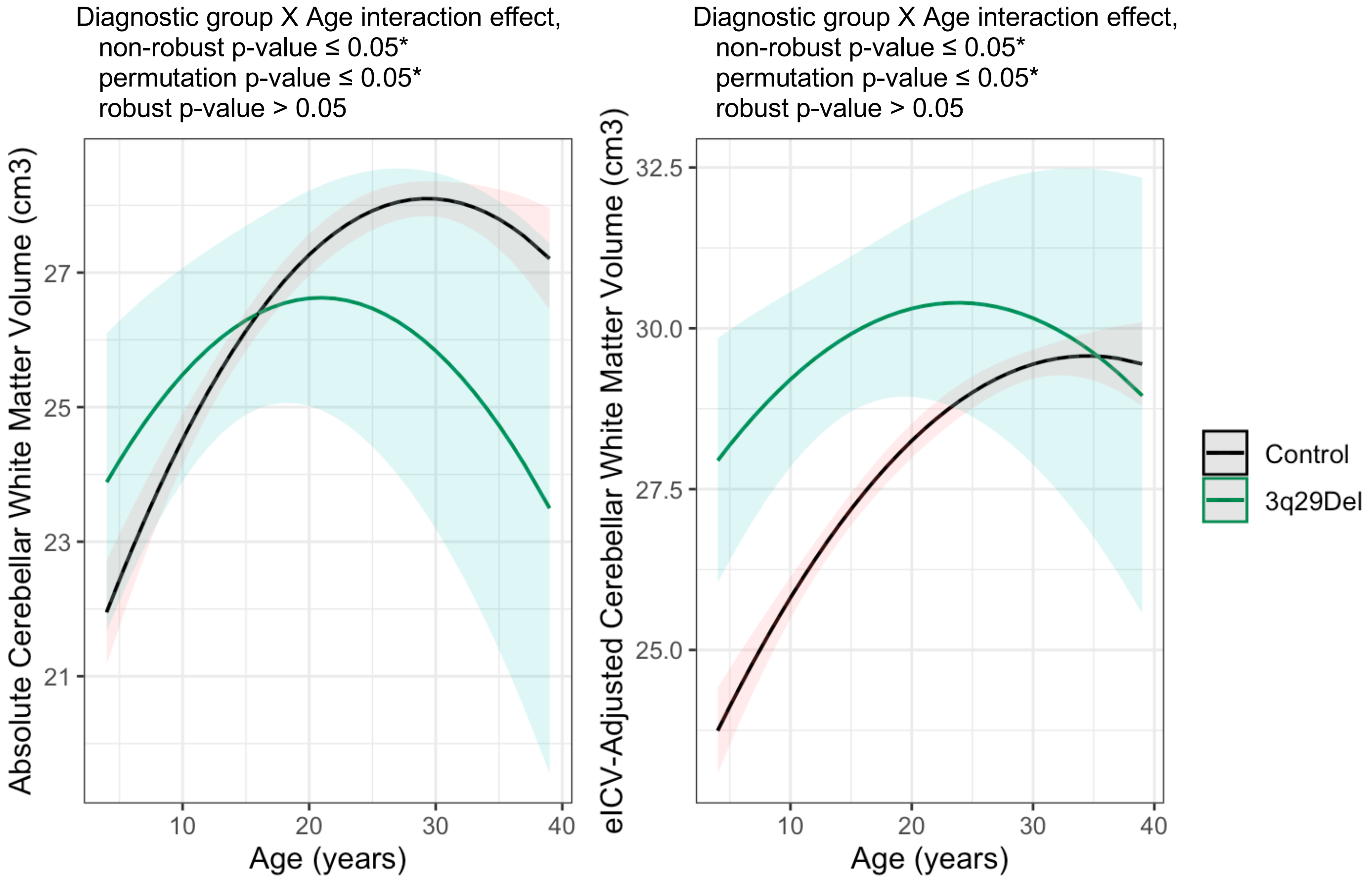

Fig. S11. Predictor effect plots showing suggestive interaction effects between diagnostic group and age on absolute and eICV-adjusted cerebellar white matter volumes. Predicted values of absolute and eICV-adjusted cerebellar white matter volumes in 3q29Del versus control groups were computed as a function of age from the exploratory interaction models reported in Table S9C and Table S9G, respectively, while covariates (age, age^2) were held fixed. Error bands indicate the 95% confidence interval. Non-robust OLS estimates, and permutation testing from Table S9C and Table S9G suggested a diagnostic group by age interaction effect on absolute and eICV-adjusted cerebellar white matter volumes (non-robust *p's* ≤ 0.05, permutation *p's* ≤ 0.05), with peak volumes reached earlier in the 3q92Del group compared with controls. However, these interaction effects were not significant (*p's* > 0.05) when robust standard error estimates were calculated to account for the heteroskedasticity in the data. We provide a graphic illustration of this finding for visual inspection of underlying deviations in neurodevelopmental trends, but we consider the evidence in favor of this interaction effect to be weak. Control *N* = 1,608, 3q29Del *N* = 23. p-value ≤ 0.001 '***', p-value ≤ 0.01 '**', p-value ≤ 0.05 '*', p-value ≤ 0.1 *'†' Abbreviations*: 3q29 deletion syndrome, 3q29Del; estimated total intracranial volume, eICV; ordinary least squares, OLS.

Males | Absolute volumes

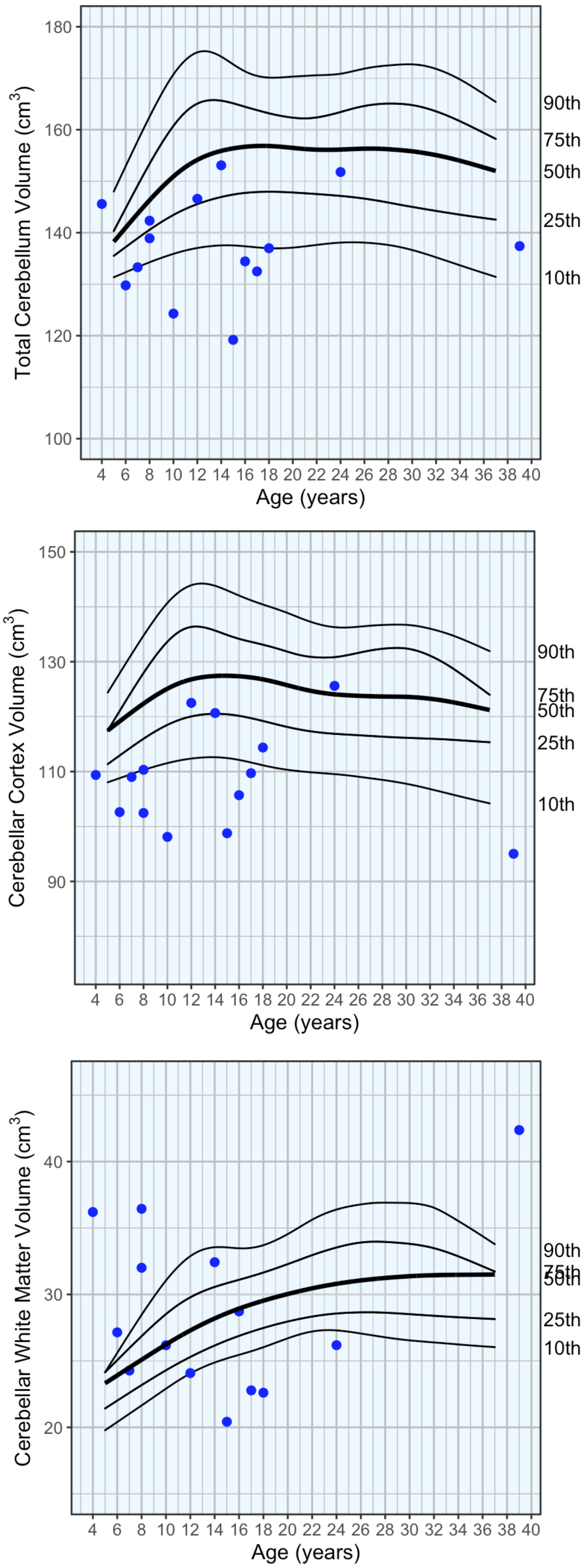

Females | Absolute volumes

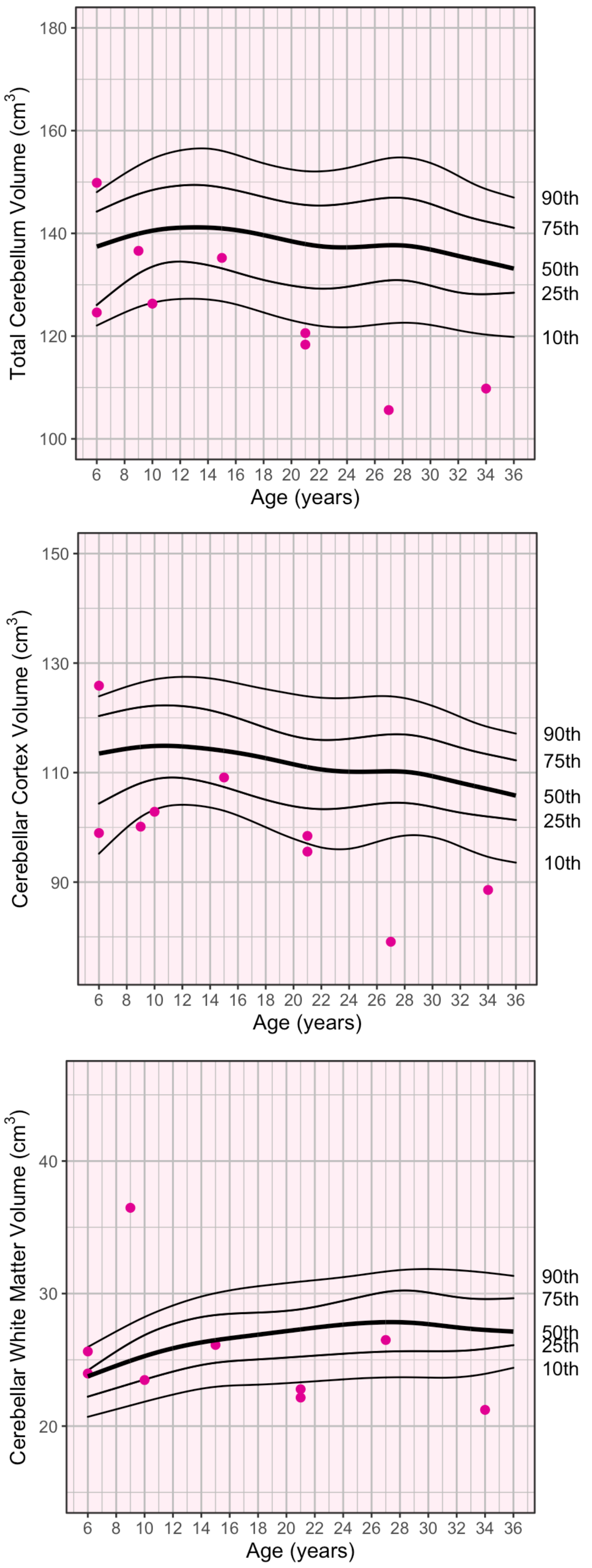

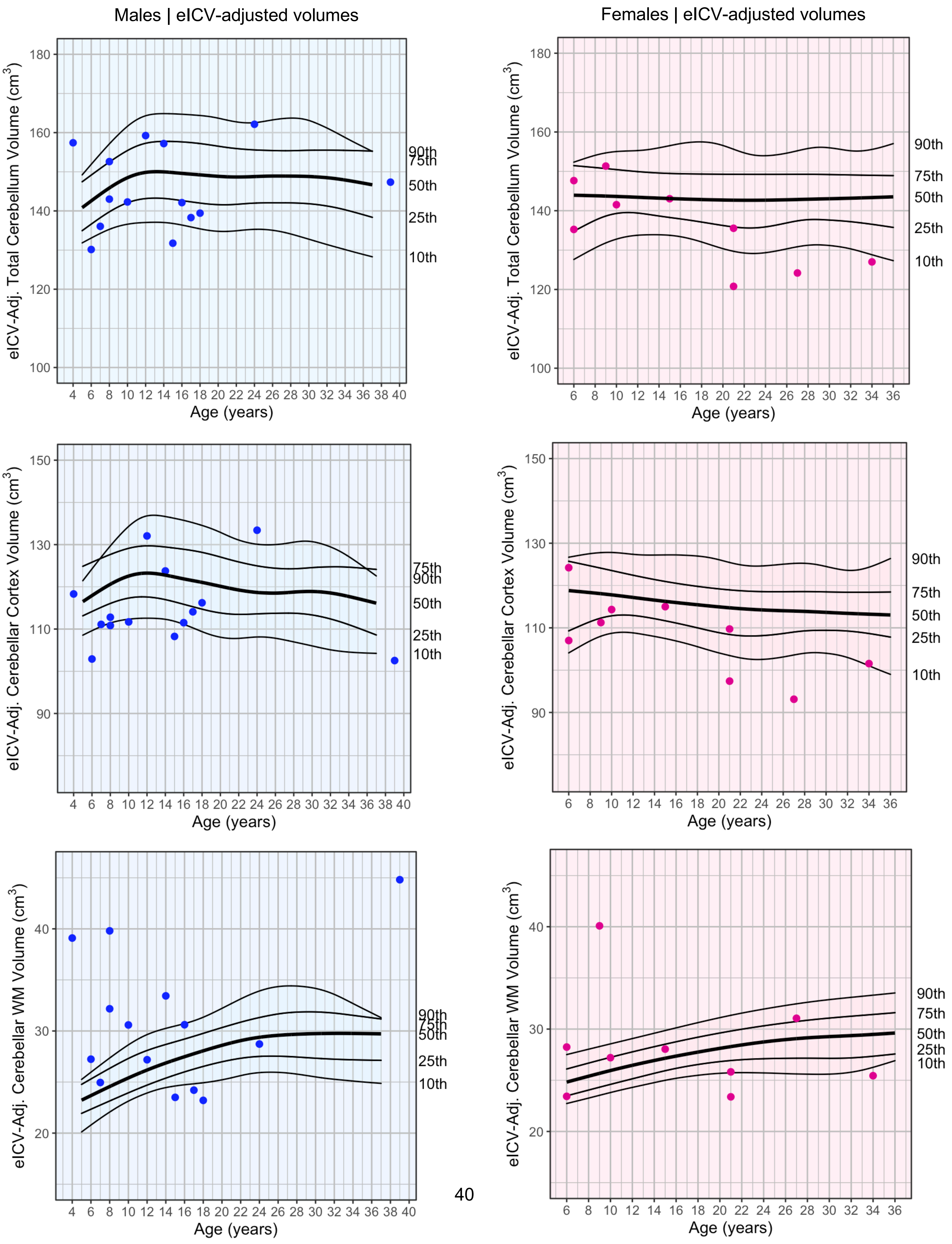
Males | eICV-adjusted volumes
Females | eICV-adjusted volumes
eICV-Adj. Total Cerebellum Volume (cm^3)
eICV-Adj. Cerebellar Cortex Volume (cm^3)
eICV-Adj. Cerebellar WM Volume (cm^3)
Age (years)
90th
75th
50th
25th
10th

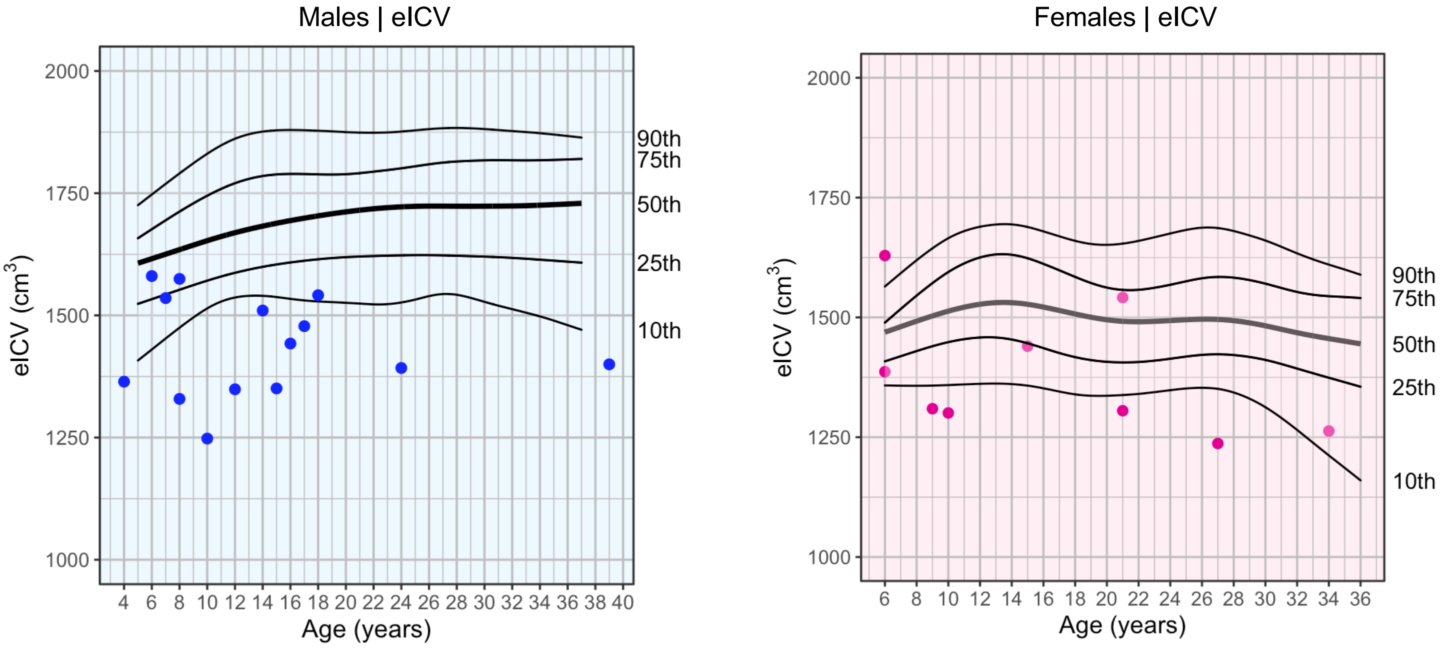

Fig. S12. Estimated normative percentile curves for tissue-specific cerebellar volumes and eICV, stratified by sex. Volumes reflect FreeSurfer-based morphometrics. Black solid lines represent the 10th, 25th, 50th (median, thicker), 75th, and 90th percentiles estimated by fitting quantile splines to volumetric data from *N* = 861 female neurotypical controls and *N* = 747 male neurotypical controls, separately. Pink dots represent data points for *N = 9* 3q29Del female participants *and* blue dots represent data points for *N = 14* 3q29Del male participants, whose volumetric measures are being compared to sex-stratified reference percentiles in neurotypical controls. A spline term of age was included as a covariate in each quantile spline model, as described by Oh et al. (2004). Due to the relative sparsity of volumetric data at the youngest and oldest endpoints of the age range covered in the present study, slight overlaps are observed in percentile bands. *Abbreviations*: 3q29 deletion syndrome, 3q29Del; estimated total intracranial volume, eICV; adjusted, adj.

Oh HS, Nychka D, Brown T, Charbonneau P (2004): Period analysis of variable stars by robust smoothing. *Journal of the Royal Statistical Society: Series C (Applied Statistics)*. *53*(1):15-30.

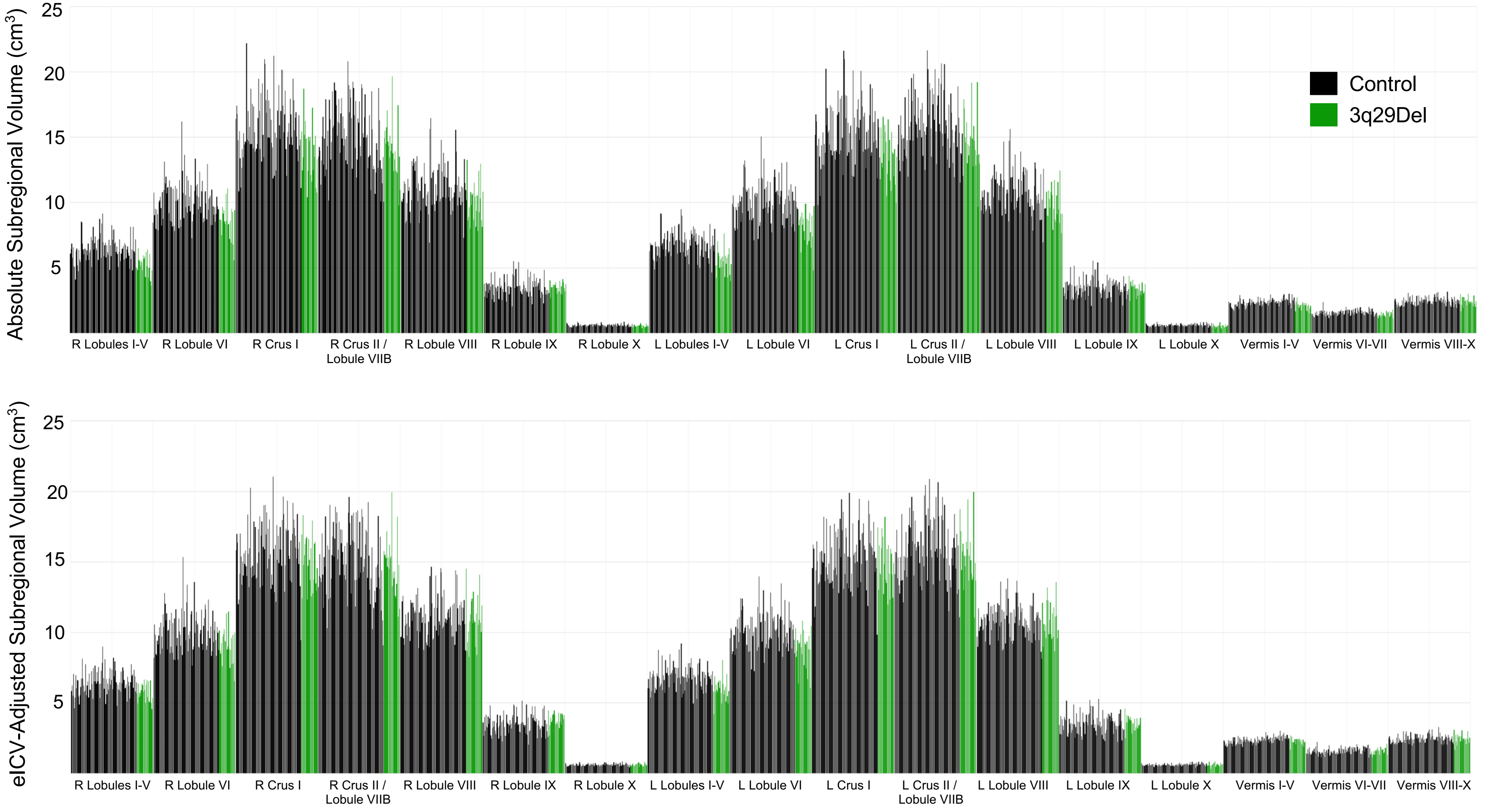

Fig. S13. Distribution of ACAPULCO-based subregional cerebellar cortex volumes across age- and sex-matched 3q29Del and neurotypical control participants. The segmentation labels for 17 subregions that cerebellar cortex was divided into with ACAPULCO are listed on the x axis. Individual bars represent the absolute (top) and eICV-adjusted (bottom) volumes of each participant. Black: control; green: 3q29Del. Control participants were randomly selected from the larger HCP sample based on exact match for sex and nearest available match for age, with a 1:4 case:control ratio. Within each group, participants are sorted in ascending order of age. A flat map diagram of the cerebellum is displayed in the bottom corner to visualize the approximate anatomical location of the interrogated segmentation labels. The illustration was adapted from Larsell (1958). *Abbreviations*: Automatic Cerebellum Anatomical Parcellation using U-Net Locally Constrained Optimization, ACAPULCO; estimated total intracranial volume, eICV; Human Connectome Project, HCP; 3q29 deletion syndrome, 3q29Del; R, right; L, left.

Larsell, O (1958): Lobules of the mammalian and human cerebellum. *Anat Rec*. 130, 329-330.

Table S10. Age- and sex-matched analyses testing for diagnostic group differences in lobule-specific cerebellar cortex volumes. Diagnostic group comparisons on ACAPULCO-based sub-regional cerebellar cortex volumes in the same age- and sex-matched sub-sample as in Table S6 are presented. Results were obtained by Student's two sample t-tests. Lower and upper bounds of the effect size (95% confidence interval) were computed with the bias-corrected and accelerated (BCa) bootstrap method using 1,000 replications.

	**ACAPULCO-based subregional comparisons** *case:control ratio = 1:4*		
	**Control** *N* = 92	**3q29Del** *N* = 23	**Test statistics**
**Lateral Hemispheres | Right Lobules I-V (cm^3)**			
**Absolute Volume**			*Cohen's d* = -1.33 *(large ES)*
Mean ± SD	6.49 ± 0.97	5.25 ± 0.76	*95% CI = -1.75 – -0.95*
Median [Range]	6.43 [4.12 – 9.18]	5.16 [3.63 – 6.51]	p-value = 9.71E-08***
**eICV-Adjusted Volume**			
Mean ± SD	6.49 ± 0.84	5.75 ± 0.63	*Cohen's d* = -0.92 *(large ES)* *95% CI = -1.34 – -0.56*
Median [Range]	6.47 [4.63 – 8.99]	5.73 [4.58 – 6.67]	p-value = 1.32E-04***
**Lateral Hemispheres | Left Lobules I-V (cm^3)**			
**Absolute Volume**			*Cohen's d* = -1.46 *(large ES)*
Mean ± SD	6.87 ± 0.96	5.49 ± 0.90	*95% CI = -1.93 – -0.93*
Median [Range]	6.85 [4.65 – 9.48]	5.50 [4.00 – 7.62]	p-value = 7.78E-09***
**eICV-Adjusted Volume**			
Mean ± SD	6.87 ± 0.83	5.98 ± 0.80	*Cohen's d* = -1.08 *(large ES)* *95% CI = -1.53 – -0.57*
Median [Range]	6.90 [4.96 – 9.21]	5.90 [4.22 – 8.04]	p-value = 9.36E-06***
**Lateral Hemispheres | Right Lobule VI (cm^3)**			
**Absolute Volume**			*Cohen's d* = -0.99 *(large ES)*
Mean ± SD	10.10 ± 1.52	8.64 ± 1.24	*95% CI = -1.39 – -0.62*
Median [Range]	9.98 [7.29 – 16.19]	8.65 [5.58 – 11.08]	p-value = 4.51E-05***
**eICV-Adjusted Volume**			
Mean ± SD	10.10 ± 1.40	9.13 ± 1.20	*Cohen's d* = -0.71 *(moderate ES)* *95% CI = -1.13 – -0.31*
Median [Range]	10.03 [7.64 – 15.37]	9.24 [6.53 – 11.50]	p-value = 3.00E-03**
**Lateral Hemispheres | Left Lobule VI (cm^3)**			
**Absolute Volume**			*Cohen's d* = -1.37 *(large ES)*
Mean ± SD	10.21 ± 1.52	8.19 ± 1.31	*95% CI = -1.77 – -0.97*
Median [Range]	10.16 [7.11 – 15.07]	8.62 [4.82 – 9.91]	p-value = 4.26E-08***
**eICV-Adjusted Volume**			
Mean ± SD	10.21 ± 1.30	8.85 ± 1.28	*Cohen's d* = -1.06 *(large ES)* *95% CI = -1.53 – -0.65*
Median [Range]	10.25 [7.27 – 13.98]	9.16 [6.08 – 10.83]	p-value = 1.45E-05***
**Lateral Hemispheres | Right Crus I (cm^3)**			
**Absolute Volume**			*Cohen's d* = -0.81 *(large ES)*
Mean ± SD	15.80 ± 2.34	13.93 ± 2.09	*95% CI = -1.23 – -0.36*
Median [Range]	15.34 [10.87 – 22.17]	14.41 [10.40 – 18.71]	p-value = 6.87E-04***
**eICV-Adjusted Volume**			
Mean ± SD	15.80 ± 2.07	15.09 ± 1.82	*Cohen's d* = -0.35 *(small ES)* *95% CI = -0.77 – 0.07*
Median [Range]	15.82 [9.44 – 21.05]	15.17 [12.34 – 18.35]	p-value = 0.13
**Lateral Hemispheres | Left Crus I (cm^3)**			
**Absolute Volume**			*Cohen's d* = -0.85 *(large ES)*
Mean ± SD	15.41 ± 2.27	13.54 ± 1.91	*95% CI = -1.26 – -0.41*
Median [Range]	15.33 [9.81 – 21.63]	13.75 [9.96 – 16.57]	p-value = 4.31E-04***

**eICV-Adjusted Volume**			
Mean ± SD	15.41 ± 1.96	14.86 ± 1.77	*Cohen's d* = -0.28 *(small ES)*
Median [Range]	15.38 [9.86 – 19.92]	14.86 [11.98 – 18.21]	*95% CI = -0.71 – 0.16*
			p-value = 0.23
**Lateral Hemispheres | Right Crus II / Lobule VIIB (cm^3)**			
**Absolute Volume**			
Mean ± SD	15.17 ± 2.48	14.16 ± 2.13	*Cohen's d* = -0.42 *(small ES)*
Median [Range]	15.03 [10.05 – 20.82]	13.95 [9.77 – 19.68]	*95% CI = -0.82 – 0.04*
			p-value = 0.07†
**eICV-Adjusted Volume**			
Mean ± SD	15.17 ± 2.39	14.82 ± 2.00	*Cohen's d* = -0.16 *(negligible ES)*
Median [Range]	15.29 [9.08 – 19.62]	14.79 [11.14 – 19.95]	*95% CI = -0.55 – 0.29*
			p-value = 0.51
**Lateral Hemispheres | Left Crus II / Lobule VIIB (cm^3)**			
**Absolute Volume**			
Mean ± SD	15.97 ± 2.27	14.77 ± 2.16	*Cohen's d* = -0.53 *(moderate ES)*
Median [Range]	15.72 [11.53 – 21.66]	14.62 [9.69 – 19.23]	*95% CI = -0.96 – -0.07*
			p-value = 0.02*
**eICV-Adjusted Volume**			
Mean ± SD	15.97 ± 2.14	15.43 ± 2.06	*Cohen's d* = -0.25 *(small ES)*
Median [Range]	15.84 [11.08 – 20.91]	15.42 [11.06 – 19.97]	*95% CI = -0.67 – 0.21*
			p-value = 0.28
**Lateral Hemispheres | Right Lobule VIII (cm^3)**			
**Absolute Volume**			
Mean ± SD	11.17 ± 1.72	9.98 ± 1.56	*Cohen's d* = -0.70 *(moderate ES)*
Median [Range]	10.92 [6.91 – 16.45]	9.79 [7.82 – 13.26]	*95% CI = -1.11 – -0.26*
			p-value = 3.18E-03**
**eICV-Adjusted Volume**			
Mean ± SD	11.17 ± 1.38	10.97 ± 1.68	*Cohen's d* = -0.14 *(negligible ES)*
Median [Range]	11.09 [8.74 – 14.65]	10.81 [8.28 – 14.54]	*95% CI = -0.66 – 0.34*
			p-value = 0.56
**Lateral Hemispheres | Left Lobule VIII (cm^3)**			
**Absolute Volume**			
Mean ± SD	10.83 ± 1.52	9.85 ± 1.31	*Cohen's d* = -0.66 *(moderate ES)*
Median [Range]	10.71 [6.96 – 15.63]	9.53 [7.68 – 12.47]	*95% CI = -1.07 – -0.25*
			p-value = 5.20E-03**
**eICV-Adjusted Volume**			
Mean ± SD	10.83 ± 1.17	10.84 ± 1.33	*Cohen's d* = 0.004 *(negligible ES)*
Median [Range]	10.89 [8.13 – 13.83]	10.61 [8.65 – 13.59]	*95% CI = -0.48 – 0.54*
			p-value = 0.99
**Lateral Hemispheres | Right Lobule IX (cm^3)**			
**Absolute Volume**			
Mean ± SD	3.54 ± 0.71	3.52 ± 0.38	*Cohen's d* = -0.04 *(negligible ES)*
Median [Range]	3.52 [2.06 – 5.50]	3.55 [2.70 – 4.16]	*95% CI = -0.35 – 0.29*
			p-value = 0.88
**eICV-Adjusted Volume**			
Mean ± SD	3.54 ± 0.68	3.85 ± 0.44	*Cohen's d* = 0.48 *(small ES)*
Median [Range]	3.52 [2.04 – 5.17]	3.85 [2.70 – 4.48]	*95% CI = 0.08 – 0.81*
			p-value = 0.04*
**Lateral Hemispheres | Left Lobule IX (cm^3)**			
**Absolute Volume**			
Mean ± SD	3.55 ± 0.70	3.42 ± 0.47	*Cohen's d* = -0.19 *(negligible ES)*
Median [Range]	3.56 [2.08 – 5.54]	3.45 [2.50 – 4.39]	*95% CI = -0.52 – 0.19*
			p-value = 0.43
**eICV-Adjusted Volume**			
Mean ± SD	3.55 ± 0.68	3.59 ± 0.47	*Cohen's d* = 0.07 *(negligible ES)*
Median [Range]	3.49 [2.25 – 5.30]	3.61 [2.50 – 4.60]	*95% CI = -0.31 – 0.44*
			p-value = 0.78
**Lateral Hemispheres | Right Lobule X (cm^3)**			
**Absolute Volume**			
Mean ± SD	0.62 ± 0.09	0.58 ± 0.10	*Cohen's d* = -0.51 *(moderate ES)*
Median [Range]	0.63 [0.44 – 0.88]	0.58 [0.40 – 0.79]	*95% CI = -0.98 – -0.02*
			p-value = 0.03*

**eICV-Adjusted Volume**<br>Mean ± SD<br>Median [Range]	0.62 ± 0.09<br>0.62 [0.45 – 0.87]	0.62 ± 0.10<br>0.62 [0.47 – 0.83]	*Cohen's d* = 0.01 *(negligible ES)*<br>*95% CI = -0.52 – 0.53*<br>p-value = 0.96
**Lateral Hemispheres | Left Lobule X (cm^3)**			
**Absolute Volume**<br>Mean ± SD<br>Median [Range]	0.63 ± 0.10<br>0.62 [0.42 – 0.89]	0.56 ± 0.11<br>0.56 [0.34 – 0.84]	*Cohen's d* = -0.69 *(moderate ES)*<br>*95% CI = -1.18 – -0.13*<br>p-value = 3.87E-03**
**eICV-Adjusted Volume**<br>Mean ± SD<br>Median [Range]	0.63 ± 0.09<br>0.62 [0.42 – 0.84]	0.63 ± 0.12<br>0.62 [0.34 – 0.87]	*Cohen's d* = -0.03 *(negligible ES)*<br>*95% CI = -0.60 – 0.54*<br>p-value = 0.90
**Vermis I-V (cm^3)**			
**Absolute Volume**<br>Mean ± SD<br>Median [Range]	2.37 ± 0.28<br>2.36 [1.70 – 3.01]	2.12 ± 0.27<br>2.17 [1.54 – 2.74]	*Cohen's d* = -0.91 *(large ES)*<br>*95% CI = -1.37 – -0.46*<br>p-value = 1.53E-04***
**eICV-Adjusted Volume**<br>Mean ± SD<br>Median [Range]	2.37 ± 0.25<br>2.36 [1.87 – 3.03]	2.29 ± 0.23<br>2.36 [1.67 – 2.69]	*Cohen's d* = -0.35 *(small ES)*<br>*95% CI = -0.79 – 0.07*<br>p-value = 0.13
**Vermis VI-VII (cm^3)**			
**Absolute Volume**<br>Mean ± SD<br>Median [Range]	1.59 ± 0.21<br>1.58 [1.16 – 2.37]	1.45 ± 0.19<br>1.40 [1.13 – 1.79]	*Cohen's d* = -0.70 *(moderate ES)*<br>*95% CI = -1.12 – -0.23*<br>p-value = 3.52E-03**
**eICV-Adjusted Volume**<br>Mean ± SD<br>Median [Range]	1.60 ± 0.23<br>1.56 [1.11 – 2.25]	1.62 ± 0.23<br>1.66 [1.15 – 2.11]	*Cohen's d* = 0.09 *(negligible ES)*<br>*95% CI = -0.39 – 0.57*<br>p-value = 0.69
**Vermis VIII-X (cm^3)**			
**Absolute Volume**<br>Mean ± SD<br>Median [Range]	2.46 ± 0.30<br>2.44 [1.94 – 3.20]	2.40 ± 0.33<br>2.42 [1.69 – 3.02]	*Cohen's d* = -0.20 *(negligible ES)*<br>*95% CI = -0.69 – 0.28*<br>p-value = 0.41
**eICV-Adjusted Volume**<br>Mean ± SD<br>Median [Range]	2.46 ± 0.29<br>2.43 [1.97 – 3.29]	2.49 ± 0.33<br>2.48 [1.69 – 3.09]	*Cohen's d* = 0.08 *(negligible ES)*<br>*95% CI = -0.48 – 0.53*<br>p-value = 0.74

p-value ≤ 0.001 '***', p-value ≤ 0.01 '**', p-value ≤ 0.05 '*', p-value ≤ 0.1 '†'. Quantification of the effect size magnitude is performed using the thresholds defined in Cohen (1992). |d| < 0.2 "negligible", |d| < 0.5 "small", |d| < 0.8 "medium", otherwise "large". *Abbreviations*: Automatic Cerebellum Anatomical Parcellation using U-Net Locally Constrained Optimization, ACAPULCO; 3q29 deletion syndrome, 3q29Del; estimated total intracranial volume, eICV; Human Connectome Project, HCP; effect size, ES.

Cohen J (1992): A power primer. *Psychological Bulletin*. 112, 155-159.

A Control (*N* = 92) vs 3q29Del (*N* = 23)

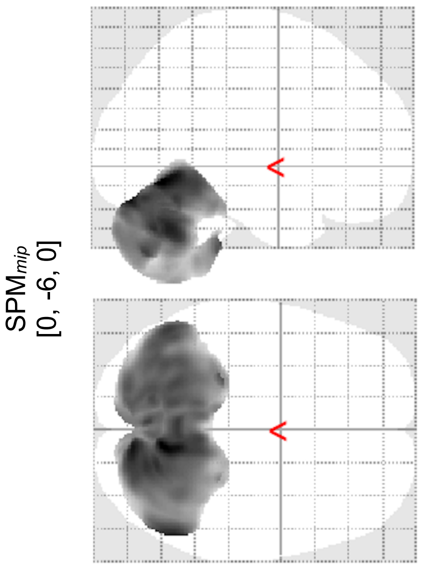

SPM-VBM Results
Height threshold: T = 3.827 {$P_{FWE\text{-}corr}$ ≤ 0.05
Extent threshold: k = 0 voxels

B Contrast

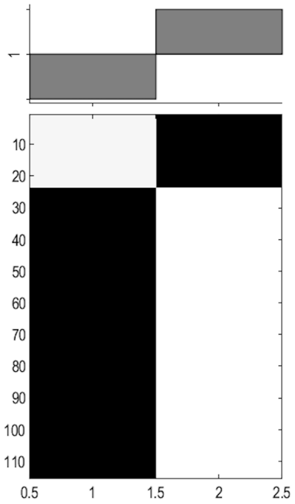

Design matrix

Voxel-level Group Differences in <u>Absolute</u> Cerebellar Gray Matter Volume

C **Statistics: p-values adjusted for the search volume**

**Cluster-level**			**Peak-level**					
$P_{FWE\text{-}corr}$	***k_E***	***P_{uncor}***	***$P_{FWE\text{-}corr}$***	***T***	***(Z_E)***	***P_{uncor}***	***mm mm mm (for local maxima)***	***Cerebellar Lobule (for local maxima)***
0.000	112,372	0.000	0.000	9.44	Inf	0.000	11, -64, -10	Right V
			0.000	8.82	7.67	0.000	11, -81, -20	Right VI
			0.000	8.53	7.47	0.000	56, -60, -34	Right Crus I

Table shows 3 local maxima more than 8.0 mm apart.

Height threshold: T = 3.83, p = 0.000 (0.050)
Extent threshold: k = 0 voxels
Expected voxels per cluster, <k> = 685.937
Expected number of clusters, <c> = 0.05
FWEp: 3.827, FWEc: 112,372

Degrees of freedom = [1.0, 113.0]
FWHM = 23.1 24.1 19.8 mm mm mm; 23.1 24.1 19.8 (voxels)
Volume: 171,796 = 171,796 voxels = 22.5 resels
Voxel size: 1.0 1.0 1.0 mm mm mm; (resel = 11055.57 voxels)

Fig. S14. SUIT-VBM analyses for voxel-level case-control differences in absolute cerebellar gray matter volumes. **A)** Visualizes the absolute cerebellar volume results in standardized space in three orthogonal planes, with the dark spots representing clusters of voxels that passed our Family-Wise Error (FWE) corrected significance threshold (FWE-adjusted *p* ≤ 0.05) to account for multiple dependent comparisons at voxel level. **B)** Displays the design matrix with the selected contrast (a row for each scan, a column for each condition). **C)** The SUIT-VBM results table was generated with the Statistical Parametric Mapping (SPM) software. The coordinates and statistical significance of all clusters above our chosen significance threshold are listed, as well as separate (> 8 mm apart) maxima within a cluster, along with details of significance thresholds and search volume reported underneath. x, y, z (mm): coordinates for each maximum are listed in standardized MNI space. Peak-level: the chance (*p*) of finding (under the null hypothesis) a peak with this or a greater height (*T- or Z-statistic*), corrected (FWE) / uncorrected for the search volume. Cluster level: the chance (*p*) of finding a cluster with this many (k_E) or a greater number of voxels, corrected (FWE) / uncorrected for the search volume. Neurotypical control participants were randomly selected from the larger HCP sample based on exact match for sex and nearest available match for age, with a 1:4 case:control ratio. Given the large number of voxels (112,372 voxels) captured by the identified cluster, the individual cerebellar lobules mapped to the specific coordinates of the local maxima do not represent the full extent of volumetric abnormalities detected in absolute cerebellar volumes. *Abbreviations*: Spatially Unbiased Infratentorial Template, SUIT; Human Connectome Project, HCP; 3q29 deletion syndrome, 3q29Del; full width at half maximum, FWHM.

A Control (*N* = 92) vs 3q29Del (*N* = 23)

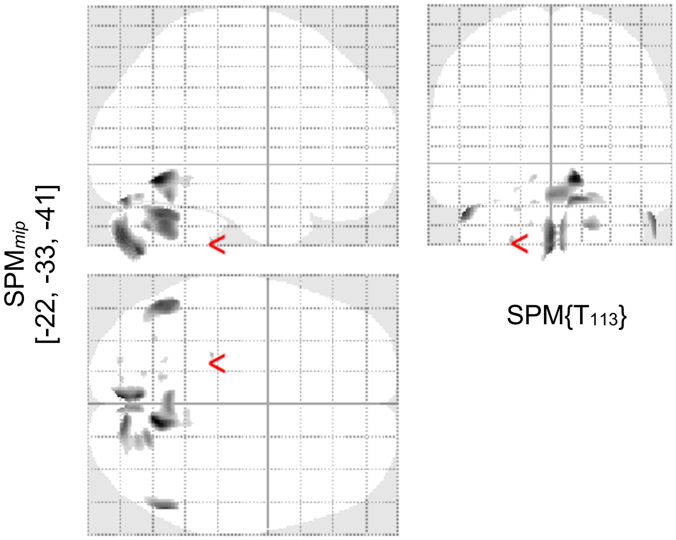

SPM-VBM Results
Height threshold: T = 3.956 {$P_{FWE\text{-}corr} \leq 0.05$
Extent threshold: k = 0 voxels

B Contrast

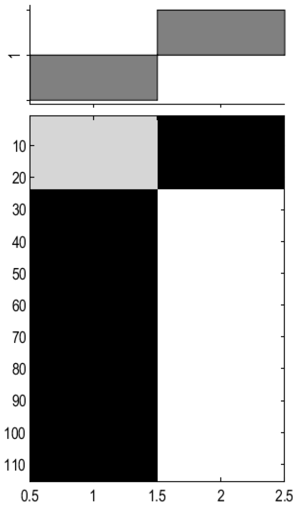

Design matrix

Voxel-level Group Differences in <u>eICV-Adjusted</u> Cerebellar Gray Matter Volume

C **Statistics: p-values adjusted for the search volume**

**Set-level**		**Cluster-level**			**Peak-level**					
P	***c***	***P$_{FWE\text{-}corr}$***	***k$_E$***	***P$_{uncor}$***	***P$_{FWE\text{-}corr}$***	***T***	***(Z$_E$)***	***P$_{uncor}$***	***mm mm mm (for local maxima)***	***Cerebellar Lobule (for local maxima)***
0.000	13	0.002	1,688	0.039	0.000	6.02	5.59	0.000	11, -64, -10	Right V
					0.003	4.85	4.61	0.000	-1, -57, -18	Left I-IV
					0.033	4.09	3.94	0.000	6, -47, -11	Right I-IV
		0.003	1,421	0.056	0.000	5.46	5.13	0.000	-2, -84, -39	Left Crus II
					0.000	5.45	5.13	0.000	-2, -74, -46	Left VIIb
					0.031	4.11	3.96	0.000	6, -82, -28	Right Crus II
		0.010	608	0.195	0.000	5.33	5.03	0.000	-50, -62, -28	Left Crus I
		0.012	500	0.238	0.001	5.25	4.95	0.000	12, -82, -20	Right Crus I
					0.012	4.41	4.22	0.000	21, -74, -18	Right VI
		0.011	576	0.206	0.001	5.19	4.90	0.000	56, -59, -32	Right Crus I
		0.021	233	0.422	0.003	4.78	4.55	0.000	19, -69, -34	Right Crus I
		0.044	14	0.876	0.016	4.32	4.15	0.000	-24, -33, -42	Left X
		0.037	50	0.734	0.023	4.21	4. 05	0.000	-12, -61, -10	Left V
		0.045	11	0.893	0.024	4.19	4.03	0.000	-11, -84, -22	Left Crus I
		0.043	16	0.865	0.031	4.11	3.96	0.000	-14, -71, -34	Left Crus I
		0.042	20	0.845	0.032	4.10	3.95	0.000	-20, -84, -25	Left Crus I
		0.045	10	0.900	0.036	4.06	3.92	0.000	-33, -65, -21	Left VI
		0.045	9	0.906	0.039	4.04	3.90	0.000	-21, -59, -15	Left VI

Table shows 3 local maxima more than 8.0 mm apart.

Height threshold: T = 3.96, p = 0.000 (0.050)
Extent threshold: k = 0 voxels
Expected voxels per cluster, <k> = 385.816
Expected number of clusters, <c> = 0.05
FWEp: 3.956, FWEc: 9

Degrees of freedom = [1.0, 113.0]
FWHM = 19.8 20.7 16.8 mm mm mm; 19.8 20.7 16.8 (voxels)
Volume: 171,796 = 171,796 voxels = 22.5 resels
Voxel size: 1.0 1.0 1.0 mm mm mm; (resel = 6876.50 voxels)

Fig. S15. SUIT-VBM analyses for voxel-level case-control differences in eICV-adjusted cerebellar gray matter volumes. A) Visualizes the eICV-adjusted cerebellar volume results in standardized space in three orthogonal planes, with

the dark spots representing clusters of voxels that passed our Family-Wise Error (FWE) corrected significance threshold (FWE-adjusted $p \leq 0.05$) to account for multiple dependent comparisons at voxel level. **B)** Displays the design matrix with the selected contrast (a row for each scan, a column for each condition). **C)** The SUIT-VBM results table was generated with the Statistical Parametric Mapping (SPM) software. The coordinates and statistical significance of all clusters above our chosen significance threshold are listed, as well as separate (> 8 mm apart) maxima within a cluster, along with details of significance thresholds and search volume reported underneath. x, y, z (mm): coordinates for each maximum are listed in standardized MNI space. Peak-level: the chance *(p)* of finding (under the null hypothesis) a peak with this or a greater height (*T- or Z-statistic*), corrected (FWE) / uncorrected for the search volume. Cluster level: the chance (*p*) of finding a cluster with this many (k_E) or a greater number of voxels, corrected (FWE) / uncorrected for the search volume. Set-level: the chance (*p*) of finding this (*c*) or a greater number of clusters in the search volume. Neurotypical control participants were randomly selected from the larger HCP sample based on exact match for sex and nearest available match for age, with a 1:4 case:control ratio. *Abbreviations*: Spatially Unbiased Infratentorial Template, SUIT; estimated total intracranial volume, eICV; Human Connectome Project, HCP; 3q29 deletion syndrome, 3q29Del; full width at half maximum, FWHM.

A Voxel-level Group Differences in Absolute Cerebellar Gray Matter Volume

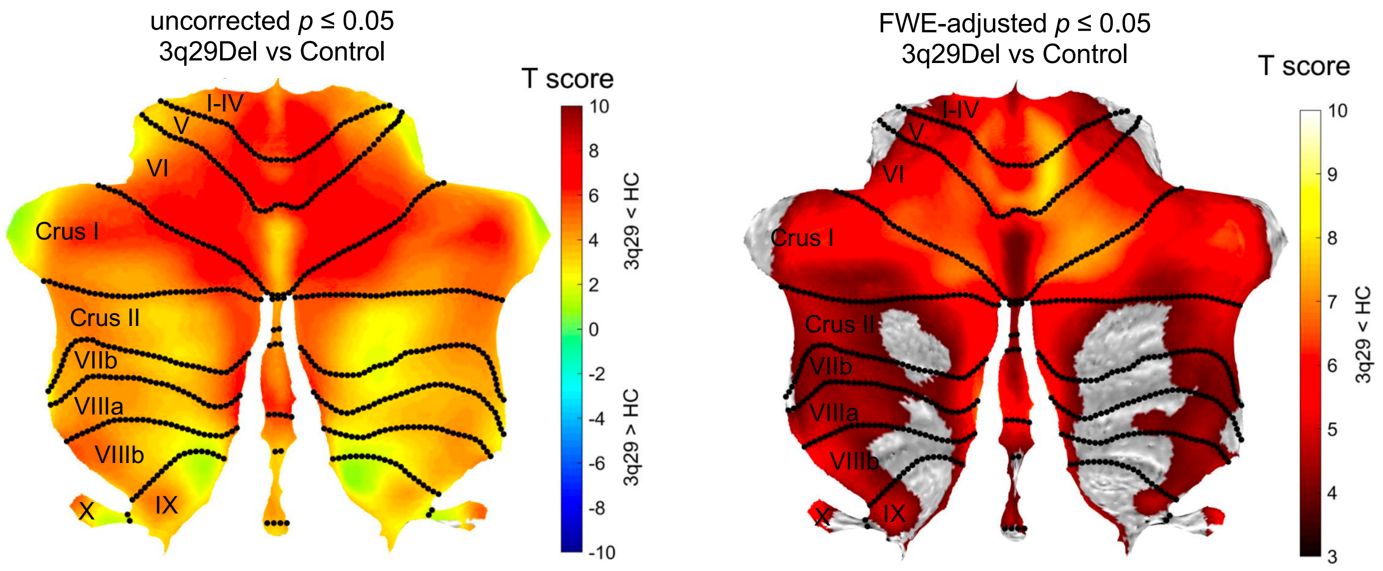

B Voxel-level Group Differences in eICV-Adjusted Cerebellar Gray Matter Volume

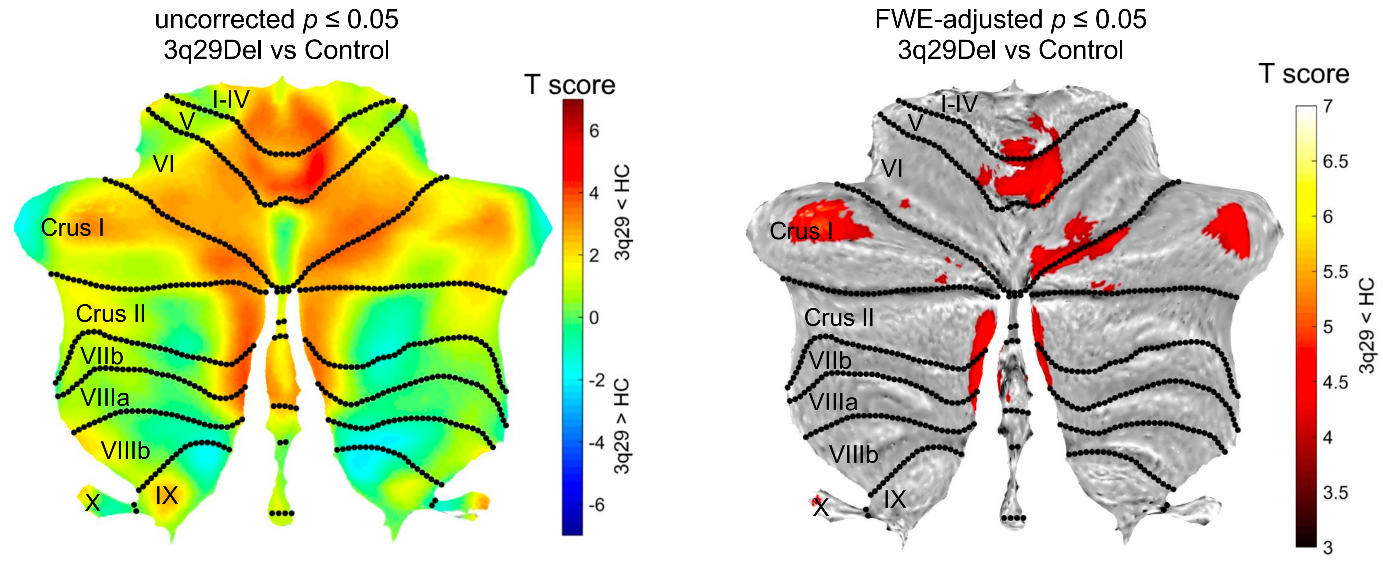

Fig. S16. Flat maps of voxel-level case-control differences in cerebellar gray matter volume identified through SUIT-VBM analyses in age- and sex-matched 3q29Del and neurotypical control participants. A) Group comparison findings from SUIT-VBM (3q29Del vs HC), displayed as a cerebellar flatmap with approximate anatomical lobular boundaries overlaid as dotted lines. Left panel plots nominally significant findings uncorrected for multiple comparisons (*p-value* ≤ 0.05); right panel plots the findings surviving FWE correction for multiple comparisons (FWE-adjusted *p-value* ≤ 0.05). **B)** Same format as A, but after adjustment for eICV. Control participants were randomly selected from the larger HCP sample based on exact match for sex and nearest available match for age, with a 1:4 case:control ratio. The color bars represent T-values. *Abbreviations*: Spatially Unbiased Infratentorial Template, SUIT; estimated total intracranial volume, eICV; Human Connectome Project, HCP; 3q29 deletion syndrome, 3q29Del; R, right; L, left.

	**PFAC/MCM -** (*N* = 11)	**PFAC/MCM +** (*N* = 13)	**Test statistics**
**Sex**, *n/N (%)*			
Male	7/11 (63.64%)	8/13 (61.54%)	OR = 0.92,<br>95% CI = 0.13 – 6.39,<br>p-value [a] = 1
Female	4/11 (36.36%)	5/13 (38.46%)	
**Age** (in years)			
Mean ± SD	17.64 ± 11.76	12.23 ± 5.72	*t = -1.47,* DF = 22,<br>p-value [b] = 0.16[b]
Median [Range]	15 [4 – 39]	10 [6 – 21]	
**Ethnicity**, *n/N (%)*			
Non-Hispanic / Latino	11/11 (100%)	12/13 (92.31%)	OR = INF,<br>95% CI = 0.02 – INF,<br>*p-value* [a] = 1
Hispanic / Latino	0/11 (0%)	1/13 (7.69%)	
**Race**, *n/N (%)*			
White	10/11 (90.91%)	12/13 (92.31%)	OR = 0.84,<br>95% CI = 0.01 – 71.84<br>p-value [a] = 1
More than one race	1/11 (9.09%)	1/13 (7.69%)	
**History of head injury**[#], *n/N (%)*			
Negative	6/8 (75.00%)	11/13 (84.62%)	OR = 0.56,<br>95% CI = 0.03 – 9.62,<br>p-value [a] = 0.62
Positive	2/8 (25.00%)	2/13 (15.38%)	
**History of neonatal complications during delivery**[#], *n/N (%)*<br>*"Did the baby have any trouble at birth?" (e.g., birth injuries, jaundice, fetal hypoxia, respiratory distress syndrome, hemorrhage); "Was the baby premature?"*			
Negative	3/8 (37.50%)	7/13 (53.85%)	OR = 0.53,<br>95% CI = 0.06 – 4.21,<br>p-value [a] = 0.66
Positive	5/8 (62.50%)	6/13 (46.15%)	
**History of maternal complications during pregnancy**[#], *n/N (%)*<br>*"Did the mother have any illness or injury during pregnancy?" (e.g., preeclampsia, infection, physical trauma)*			
Negative	4/8 (50.00%)	9/13 (69.23%)	OR = 0.46,<br>95% CI = 0.05 – 3.90,<br>p-value [a] = 0.65
Positive	4/8 (50.00%)	4/13 (30.77%)	
**Combined history of head injury, neonatal complications during delivery, and/or maternal complications during pregnancy**[#], *n/N (%)*			
Negative	2/8 (25.00%)	4/13 (30.77%)	OR= 0.76,<br>95% CI= 0.05 – 7,55,<br>p-value [a] = 1
Positive	6/8 (75.00%)	9/13 (69.23%)	

Table S11. Demographic and relevant clinical characteristics of 3q29Del participants with versus without posterior fossa arachnoid cyst or mega cisterna magna findings. There was no significant difference between PFAC/MCM positive (+) and negative (-) 3q29Del participants in demographic characteristics or in clinical characteristics that have been proposed to be associated with the etiology of secondary (acquired) cysts in previous literature (*p's* > 0.05). [a]Fisher's exact test, [b]Student's two sample t-test. [#]3q29Del *N* = 21 due to missing data. *Abbreviations:* 3q29 deletion syndrome, 3q29Del; posterior fossa arachnoid cyst, PFAC; mega cisterna magna, MCM; standard deviation, SD; odds ratio, OR; confidence interval, CI.

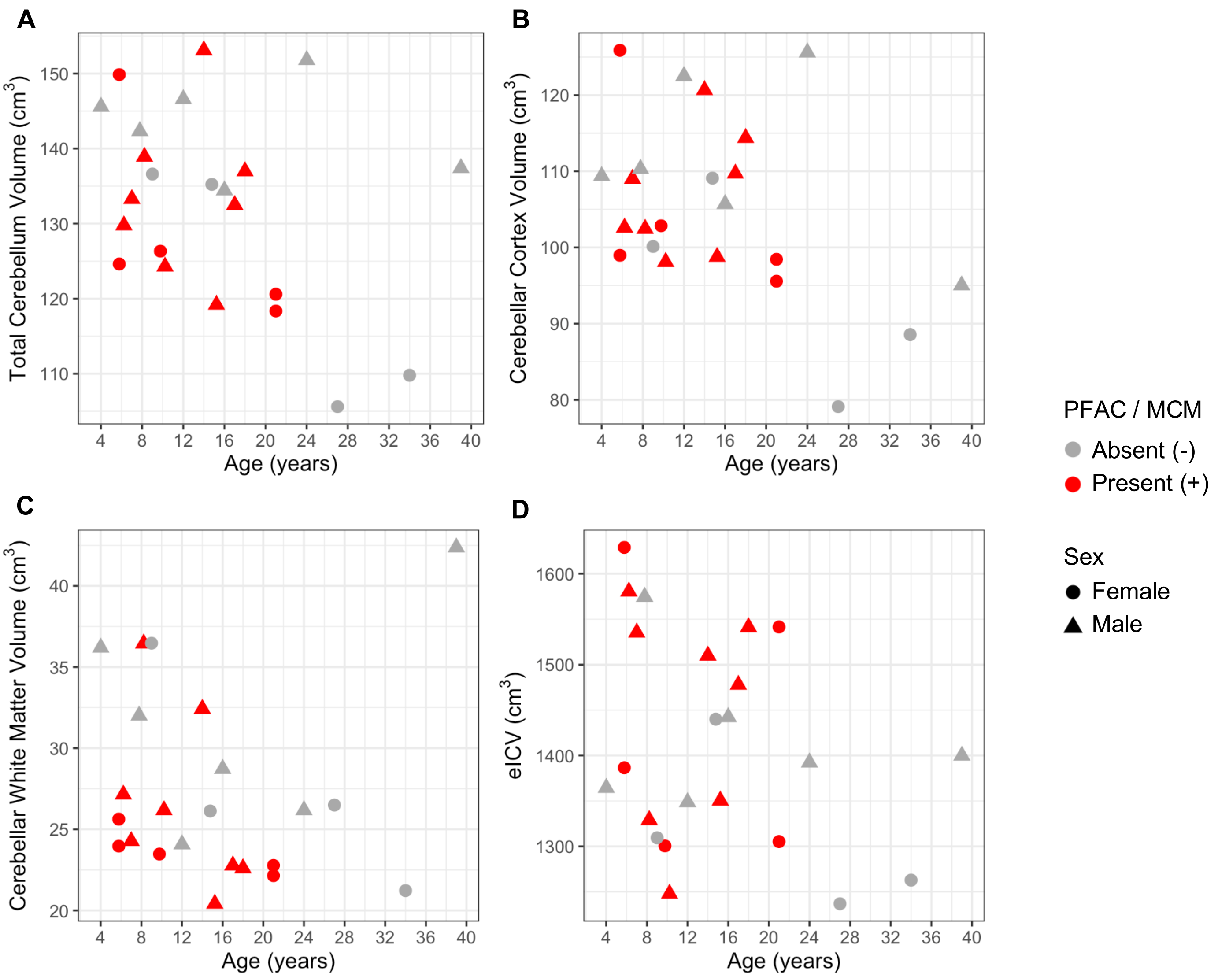

Fig. S17. Scatter plots showing the distribution of A) total cerebellum volume, B) cerebellar cortex volume, C) cerebellar white matter volume, and D) eICV as a function of age among 3q29Del participants with versus without posterior fossa arachnoid cyst or mega cisterna magna findings. Volumes reflect FreeSurfer-based morphometrics. A slight jitter was added systematically to all panels to minimize overplotting. 3q29Del *N* = 23 (Female *N* = 9, Male *N* = 14). *Abbreviations*: 3q29 deletion syndrome, 3q29Del; estimated total intracranial volume, eICV; posterior fossa arachnoid cyst, PFAC; mega cisterna magna, MCM.

		Model 1			Model 2 (with eICV-adjustment)		
**Outcome variables**	**Explanatory variables**	***b***	**CI (95%)**	**p-value**	***b***	**CI (95%)**	**p-value**
**Total Cerebellum Volume (cm³)**	Intercept	138.53	123.93 – 153.12	3.59E-14***	79.17	20.03 – 138.32	0.01**
	Age (years)	-0.57	-1.13 – -0.004	0.04*	-0.46	-0.99 – 0.06	0.08†
	Sex [Male]	11.11	1.41 – 20.81	0.03*	8.99	-0.15 – 18.13	0.05*
	eICV (cm³)				0.04	0.001 – 0.08	0.04*
	**PFAC / MCM [Positive]**	**-7.07**	**-17.22 – 3.09**	**0.16**	**-9.09**	**-18.62 – 0.44**	**0.06†**
	R^2 / R^2 adjusted	0.39 / 0.30			0.52 / 0.41		
	F-statistic	4.1 on 3 and 19 DF, p-value = 0.02*			4.8 on 4 and 18 DF, p-value = 0.01**		
**Cerebellar Cortex Volume (cm³)**	Intercept	108.93	94.64 – 123.22	1.85E-12***	47.17	-9.75 – 104.10	0.10†
	Age (years)	-0.49	-1.04 – 0.07	0.08†	-0.38	-0.89 – 0.13	0.13
	Sex [Male]	7.89	-1.61 – 17.39	0.10†	5.69	-3.11 – 14.49	0.19
	eICV (cm³)				0.04	0.005 – 0.08	0.03*
	**PFAC / MCM [Positive]**	**-1.90**	**-11.84 – 8.04**	**0.69**	**-4.01**	**-13.18 – 5.17**	**0.37**
	R^2 / R^2 adjusted	0.28 / 0.17			0.45 / 0.33		
	F-statistic	2.5 on 3 and 19 DF, p-value = 0.09†			3.7 on 4 and 18 DF, p-value = 0.02*		
**Cerebellar White Matter Volume (cm³)**	Intercept	37.25	28.70 – 45.80	3.40E-08***	41.34	11.67 – 71.02	9.15E-03**
	Age (years)	-1.22	-2.11 – -0.33	9.82E-03**	-1.23	-2.15 – -0.31	0.01**
	Age^2	0.03	0.01 – 0.05	0.01**	0.03	0.01 – 0.05	0.01**
	Sex [Male]	2.93	-1.28 – 7.14	0.16	3.07	-1.38 – 7.53	0.16
	eICV (cm³)				-0.003	-0.02 – 0.02	0.76
	**PFAC / MCM [Positive]**	**-3.97**	**-8.47 – 0.52**	**0.08†**	**-3.83**	**-8.57 – 0.91**	**0.11**
	R^2 / R^2 adjusted	0.48 / 0.37			0.48 / 0.33		
	F-statistic	4.2 on 4 and 18 DF, p-value = 0.01**			3.2 on 5 and 17 DF, p-value = 0.03*		
**Estimated Total Intracranial Volume (eICV) (cm³)**	Intercept	1392.33	1236.74 – 1547.91	1.05E-13***			
	Age (years)	-2.39	-8.39 – 3.61	0.41			
	Sex [Male]	49.68	-53.72 – 153.08	0.33			
	**PFAC / MCM [Positive]**	**47.55**	**-60.68 – 155.79**	**0.37**			
	R^2 / R^2 adjusted	0.16 / 0.03					
	F-statistic	1.2 on 3 and 19 DF, p-value = 0.32					

Table S12. Exploratory analysis of the relationship between posterior fossa arachnoid cyst and mega cisterna magna findings and tissue-specific cerebellar volumes and eICV among 3q29Del participants. Multiple linear regression results for total cerebellum volume, cerebellar cortex volume**,** cerebellar white matter volume and eICV indicate no significant relationship between the likelihood of PFAC/MCM findings and interrogated VOIs among 3q29Del participants (*p's* > 0.05), while correcting for sex, age, age^2 (if appropriate) and eICV (in model 2 only). The main effect of the

binary PFAC/MCM variable is reported in bold for clarity. The age term/s included in each model reflect the best-fitting polynomial expansion of age for a given VOI selected by ANOVA. Major assumptions for ordinary least squares regression were met in the current analysis, hence additional heteroskedasticity-robust estimates were not calculated. F-statistics are reported to assess the overall significance of each model. 3q29Del *N* = 23. Contrast coding: reference levels for the PFAC/MCM and sex variables are negative and female, respectively. p-value ≤ 0.001 '***', p-value ≤ 0.01 '**', p-value ≤ 0.05 '*', p-value ≤ 0.1 '†'. *Abbreviations*: 3q29 deletion syndrome, 3q29Del; volumetric measure of interest, VOI; posterior fossa arachnoid cyst, PFAC; mega cisterna magna, MCM; estimated total intracranial volume, eICV; analysis of variance, ANOVA; unstandardized coefficient estimate, *b*; confidence interval, CI; degrees of freedom, DF.

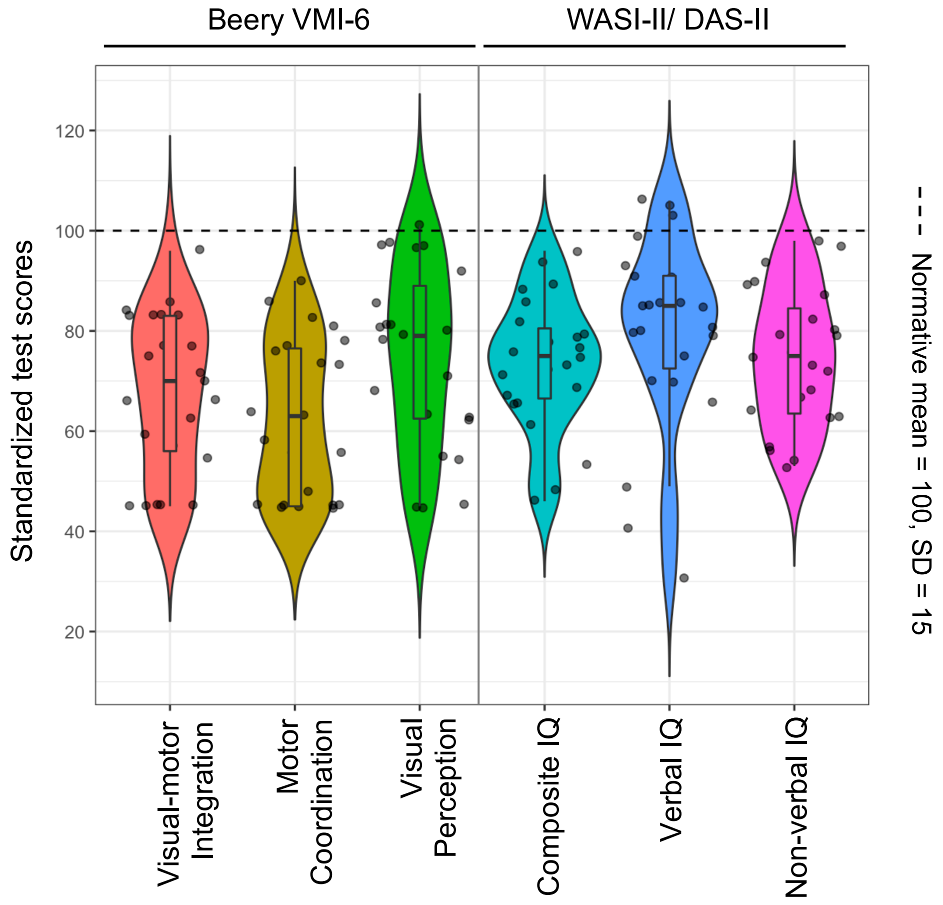

Fig. S18. Violin plots with box plots visualizing the distribution of standardized test scores for sensorimotor and cognitive abilities among 3q29Del participants. Violin plots represent the distribution of standardized test scores (normative mean = 100, SD = 15) for visual-motor integration, motor coordination and visual perception skills measured by the Beery-Buktenica Developmental Test of VMI, and for composite, verbal and non-verbal IQ scores measured by the WASI / DAS. Boxplots visualize the five-number summary statistics for each measure (minimum, lower quartile, median, upper quartile and maximum). Higher scores indicate better performance. 3q29Del *N* = 23. *Abbreviations*: 3q29 deletion syndrome, 3q29Del; intelligence quotient, IQ; Wechsler Abbreviated Scale of Intelligence, WASI; Differential Ability Scales, DAS; visual-motor integration, VMI; standard deviation, SD.

**Standardized test scores**	**3q29Del** (*N* = 23)
**Visual-motor Integration** (*Beery VMI-6)*	
Mean ± SD	67.83 ± 15.87
Median [Range]	70 [45 – 96]
**Supplemental test: Motor Coordination** (*Beery VMI-6)*	
Mean ± SD	62.65 ± 15.68
Median [Range]	63 [45 – 90]
**Supplemental test: Visual Perception** (*Beery VMI-6)*	
Mean ± SD	74.74 ± 18.20
Median [Range]	79 [45 – 101]
**Composite IQ** *(WASI-II/ DAS-II)*	
Mean ± SD	73.48 ± 13.30
Median [Range]	75 [46 – 96]
**Supplemental test: Verbal IQ** *(WASI-II/ DAS-II)*	
Mean ± SD	79.87 ± 19.13
Median [Range]	85 [31 – 106]
**Supplemental test: Non-verbal IQ** *(WASI-II/ DAS-II)*	
Mean ± SD	74.61 ± 13.80
Median [Range]	75 [53 – 98]

Table. S13. Descriptive statistics for standardized test scores for sensorimotor and cognitive abilities among 3q29Del participants. *Abbreviations*: 3q29 deletion syndrome, 3q29Del; intelligence quotient, IQ; Wechsler Abbreviated Scale of Intelligence, WASI; Differential Ability Scales, DAS; visual-motor integration, VMI; standard deviation, SD.

Table S14. Extended multiple linear regression results testing the relationships between tissue-specific cerebellar volumes and sensorimotor and cognitive abilities among 3q29Del participants. Main effects of FreeSurfer-based cerebellar cortex and white matter volumes are reported in bold for clarity. Regression parameters reflect heteroskedasticity-robust estimates. Robust Wald test statistics are reported to assess the overall significance of each model. Results indicate significant relationships between cerebellar white matter volume, visual-motor integration skills ($p \leq 0.01$), composite IQ ($p \leq 0.01$), verbal IQ ($p \leq 0.05$), and non-verbal IQ ($p \leq 0.05$) among 3q29Del participants, while correcting for age and sex. These effects persisted in secondary models including eICV as an additional covariate (*p's* ≤ 0.05). 3q29Del *N* = 23.

Outcome variable	Explanatory variables	*b*	CI (95%)	p-value
**Visual-motor Integration** *(standardized score, Beery VMI-6)*	Intercept	100.65	17.78 – 183.51	0.02*
	Age (years)	-0.63	-1.72 – 0.46	0.24
	Sex [Male]	-4.22	-19.46 – 11.02	0.57
	**Cerebellar Cortex Volume (cm^3)**	**-0.20**	**-0.95 – 0.56**	**0.59**
	R^2 / R^2 adjusted	0.13 / -0.01		
	Robust Wald Test	0.6 on 3 and 19 DF, p-value = 0.63		
	Intercept	44.93	15.79 – 74.07	4.44E-03**
	Age (years)	-0.57	-1.24 – 0.10	0.09†
	Sex [Male]	-10.47	-24.59 – 3.65	0.14
	**Cerebellar WM Volume (cm^3)**	**1.38**	**0.36 – 2.41**	**0.01****
	R^2 / R^2 adjusted	0.35 / 0.25		
	Robust Wald Test	4.0 on 3 and 19 DF, p-value = 0.02*		
**Supplemental test: Motor Coordination** *(standardized score, Beery VMI-6)*	Intercept	44.27	-44.76 – 133.29	0.31
	Age (years)	0.28	-0.71 – 1.28	0.56
	Sex [Male]	-5.78	-20.33 – 8.78	0.42
	**Cerebellar Cortex Volume (cm^3)**	**0.17**	**-0.60 – 0.94**	**0.65**
	R^2 / R^2 adjusted	0.05 / -0.10		
	Robust Wald Test	0.4 on 3 and 19 DF, p-value = 0.77		
	Intercept	46.45	15.79 – 77.11	5.03E-03**
	Age (years)	0.20	-0.53 – 0.92	0.58
	Sex [Male]	-6.58	-21.59 – 8.42	0.37
	**Cerebellar WM Volume (cm^3)**	**0.63**	**-0.45 – 1.71**	**0.24**
	R^2 / R^2 adjusted	0.09 / -0.05		
	Robust Wald Test	0.8 on 3 and 19 DF, p-value = 0.53		
**Supplemental test: Visual Perception** *(standardized score, Beery VMI-6)*	Intercept	109.13	24.51 – 193.76	0.01**
	Age (years)	-0.60	-1.68 – 0.48	0.26
	Sex [Male]	-5.46	-22.30 – 11.38	0.51
	**Cerebellar Cortex Volume (cm^3)**	**-0.21**	**-0.96 – 0.54**	**0.57**
	R^2 / R^2 adjusted	0.10 / -0.04		
	Robust Wald Test	0.6 on 3 and 19 DF, p-value = 0.62		
	Intercept	65.04	30.90 – 99.18	7.88E-04***
	Age (years)	-0.52	-1.34 – 0.29	0.19
	Sex [Male]	-10.06	-30.39 – 10.28	0.31
	**Cerebellar WM Volume (cm^3)**	**0.87**	**-0.83 – 2.56**	**0.30**
	R^2 / R^2 adjusted	0.16 / 0.03		
	Robust Wald Test	0.8 on 3 and 19 DF, p-value = 0.52		

	Intercept	62.45	-4.14 – 129.04	0.06†
	Age (years)	0.06	-0.86 – 0.97	0.90
	Sex [Male]	-5.15	-18.74 – 8.44	0.44
	**Cerebellar Cortex Volume (cm^3)**	**0.13**	**-0.47 – 0.72**	**0.66**
	R^2 / R^2 adjusted	0.03 / -0.12		
**Composite IQ** *(standardized score, WASI-II/ DAS-II)*	Robust Wald Test	0.2 on 3 and 19 DF, p-value = 0.88		
	Intercept	42.69	19.34 – 66.03	1.14E-03**
	Age (years)	-0.03	-0.56 – 0.50	0.90
	Sex [Male]	-8.66	-20.51 – 3.18	0.14
	**Cerebellar WM Volume (cm^3)**	**1.33**	**0.36 – 2.31**	**9.89E-03****
	R^2 / R^2 adjusted	0.34 / 0.23		
	Robust Wald Test	3.1 on 3 and 19 DF, p-value = 0.05*		
	Intercept	46.24	-32.13 – 124.61	0.23
	Age (years)	0.28	-0.72 – 1.29	0.56
	Sex [Male]	-9.09	-25.92 – 7.74	0.27
	**Cerebellar Cortex Volume (cm^3)**	**0.33**	**-0.36 – 1.02**	**0.33**
**Supplemental test: Verbal IQ** *(standardized score, WASI-II/ DAS-II)*	R^2 / R^2 adjusted	0.06 / -0.08		
	Robust Wald Test	0.5 on 3 and 19 DF, p-value = 0.66		
	Intercept	43.93	12.21 – 75.64	9.20E-03**
	Age (years)	0.10	-0.65 – 0.85	0.78
	Sex [Male]	-11.58	-28.35 – 5.18	0.16
	**Cerebellar WM Volume (cm^3)**	**1.51**	**0.13 – 2.89**	**0.03***
	R^2 / R^2 adjusted	0.23 / 0.11		
	Robust Wald Test	2.9 on 3 and 19 DF, p-value = 0.06†		
	Intercept	94.11	33.85 – 154.37	4.04E-03**
	Age (years)	-0.52	-1.55 – 0.50	0.30
	Sex [Male]	-5.40	-19.42 – 8.63	0.43
	**Cerebellar Cortex Volume (cm^3)**	**-0.08**	**-0.64 – 0.48**	**0.77**
**Supplemental test: Non-verbal IQ** *(standardized score, WASI-II/ DAS-II)*	R^2 / R^2 adjusted	0.14 / 0.003		
	Robust Wald Test	0.5 on 3 and 19 DF, p-value = 0.67		
	Intercept	53.59	23.85 – 83.33	1.29E-03**
	Age (years)	-0.52	-1.11 – 0.07	0.08†
	Sex [Male]	-10.37	-22.86 – 2.12	0.10†
	**Cerebellar WM Volume (cm^3)**	**1.28**	**0.17 – 2.39**	**0.03***
	R^2 / R^2 adjusted	0.41 / 0.31		
	Robust Wald Test	3.0 on 3 and 19 DF, p-value = 0.6†		

Secondary models for tissue-specific volumes that were found to have a significant main effect on sensorimotor and cognitive abilities ($p \leq 0.05$), after eICV is included as an additional covariate:

**Outcome variables**	**Explanatory variables**	***b***	**CI (95%)**	**p-value**
	Intercept	-38.13	-122.35 – 46.08	0.35
	Age (years)	-0.39	-0.97 – 0.19	0.18
**Visual-motor Integration** *(standardized score, Beery VMI-6)*	Sex [Male]	-13.59	-25.64 – -1.54	0.03*
	eICV (cm^3)	0.06	0.002 – 0.11	0.04*
	**Cerebellar WM Volume (cm^3)**	**1.51**	**0.64 – 2.39**	**1.87E-03****
	R^2 / R^2 adjusted	0.50 / 0.38		
	Robust Wald Test	5.1 on 4 and 18 DF, p-value = 6.08E-03**		

**Composite IQ** *(standardized score, WASI-II/ DAS-II)*	Intercept	-15.38	-76.16 – 45.39	0.60
	Age (years)	0.10	-0.42 – 0.61	0.70
	Sex [Male]	-10.85	-21.04 – -0.65	0.04*
	eICV (cm^3)	0.04	0.00 – 0.08	0.06†
	**Cerebellar WM Volume (cm^3)**	**1.43**	**0.50 – 2.35**	**4.66E-03****
	R^2 / R^2 adjusted	0.44 / 0.31		
	Robust Wald Test	6.2 on 4 and 18 DF, p-value = 2.57E-03**		
**Verbal IQ** *(standardized score, WASI-II/ DAS-II)*	Intercept	-2.26	-103.80 – 99.28	0.96
	Age (years)	0.20	-0.59 – 1.00	0.60
	Sex [Male]	-13.32	-29.75 – 3.10	0.11
	eICV (cm^3)	0.03	-0.03 – 0.09	0.31
	**Cerebellar WM Volume (cm^3)**	**1.59**	**0.17 – 3.01**	**0.03***
	R^2 / R^2 adjusted	0.26 / 0.10		
	Robust Wald Test	2.5 on 4 and 18 DF, p-value = 0.08†		
**Non-verbal IQ** *(standardized score, WASI-II/ DAS-II)*	Intercept	6.21	-54.98 – 67.40	0.83
	Age (years)	-0.41	-0.96 – 0.13	0.13
	Sex [Male]	-12.15	-22.77 – -1.53	0.03*
	eICV (cm^3)	0.03	-0.004 – 0.07	0.08†
	**Cerebellar WM Volume (cm^3)**	**1.36**	**0.31 – 2.40**	**0.01****
	R^2 / R^2 adjusted	0.47 / 0.35		
	Robust Wald Test	5.6 on 4 and 18 DF, p-value = 4.03E-03**		

Contrast coding: reference level for the sex variable is female. p-value ≤ 0.001 '***', p-value ≤ 0.01 '**', p-value ≤ 0.05 '*', p-value ≤ 0.1 '†'. *Abbreviations*: 3q29 deletion syndrome, 3q29Del; intelligence quotient, IQ; Wechsler Abbreviated Scale of Intelligence, WASI; Differential Ability Scales, DAS; visual-motor integration, VMI; white matter, WM; unstandardized coefficient estimate, *b*; confidence interval, CI; degrees of freedom, DF; estimated total intracranial volume, eICV.

.

Table S15. Multiple linear regression results testing the relationships between ACAPULCO-based subregional cerebellar cortex volumes and sensorimotor abilities among 3q29Del participants. Main effects of ACAPULCO-based subregional cerebellar cortex volumes are reported in bold. Regression parameters reflect heteroskedasticity-robust estimates. Robust Wald test statistics are reported to assess the overall significance of each model. Results indicate a significant inverse relationship between vermis VI-VII volumes and visual perception skills, while correcting for age and sex ($p \leq 0.05$). This effect persisted after eICV was included as an additional covariate in a secondary model ($p \leq 0.05$). In addition, a trend level inverse relationship was identified between vermis VI-VII and visual-motor integration skills; and trend level positive relationships were found between right and left hemispheric lobule VI and motor coordination skills, while correcting for age and sex (*p's* ≤ 0.10). 3q29Del *N* = 23.

Outcome variable	Explanatory variables	*b*	CI (95%)	p-value
**Visual-motor Integration** *(standardized score, Beery VMI-6)*	Intercept	85.40	18.92 – 151.89	0.01**
	Age (years)	-0.57	-1.78 – 0.64	0.33
	Sex [Male]	-5.44	-19.78 – 8.90	0.44
	**Right Lobules I-V (cm^3)**	**-1.07**	**-11.69 – 9.55**	**0.84**
	R^2 / R^2 adjusted	0.12 / -0.02		
	Robust Wald Test	0.4 on 3 and 19 DF, p-value = 0.73		
**Visual-motor Integration** *(standardized score, Beery VMI-6)*	Intercept	72.50	11.32 – 133.69	0.02*
	Age (years)	-0.51	-1.69 – 0.66	0.37
	Sex [Male]	-5.56	-19.72 – 8.59	0.42
	**Left Lobules I-V (cm^3)**	**1.17**	**-7.34 – 9.68**	**0.78**
	R^2 / R^2 adjusted	0.12 / -0.02		
	Robust Wald Test	0.6 on 3 and 19 DF, p-value = 0.61		
**Visual-motor Integration** *(standardized score, Beery VMI-6)*	Intercept	48.97	-14.34 – 112.29	0.12
	Age (years)	-0.40	-1.47 – 0.68	0.45
	Sex [Male]	-6.99	-19.13 – 5.15	0.24
	**Right Lobule VI (cm^3)**	**3.37**	**-2.54 – 9.27**	**0.25**
	R^2 / R^2 adjusted	0.18 / 0.05		
	Robust Wald Test	2.6 on 3 and 19 DF, p-value = 0.08†		
**Visual-motor Integration** *(standardized score, Beery VMI-6)*	Intercept	65.66	9.24 – 122.09	0.02*
	Age (years)	-0.49	-1.59 – 0.61	0.36
	Sex [Male]	-6.98	-19.92 – 5.96	0.27
	**Left Lobule VI (cm^3)**	**1.69**	**-3.87 – 7.25**	**0.53**
	R^2 / R^2 adjusted	0.13 / -0.003		
	Robust Wald Test	1.4 on 3 and 19 DF, p-value = 0.28		
**Visual-motor Integration** *(standardized score, Beery VMI-6)*	Intercept	60.54	0.79 – 120.29	0.05*
	Age (years)	-0.44	-1.62 – 0.75	0.45
	Sex [Male]	-6.27	-19.77 – 7.23	0.34
	**Right Crus I (cm^3)**	**1.27**	**-2.00 – 4.54**	**0.43**
	R^2 / R^2 adjusted	0.14 / 0.004		
	Robust Wald Test	1.1 on 3 and 19 DF, p-value = 0.37		
**Visual-motor Integration** *(standardized score, Beery VMI-6)*	Intercept	59.59	-6.00 – 125.19	0.07†
	Age (years)	-0.43	-1.62 – 0.76	0.46
	Sex [Male]	-6.94	-19.82 – 5.94	0.27
	**Left Crus I (cm^3)**	**1.40**	**-2.48 – 5.28**	**0.46**
	R^2 / R^2 adjusted	0.14 / 0.003		
	Robust Wald Test	1.3 on 3 and 19 DF, p-value = 0.32		
**Visual-motor Integration** *(standardized score, Beery VMI-6)*	Intercept	82.07	29.38 – 134.77	4.12E-03**
	Age (years)	-0.56	-1.60 – 0.48	0.28
	Sex [Male]	-5.62	-20.56 – 9.33	0.44
	**Right Crus II / Lobule VIIB (cm^3)**	**-0.17**	**-3.59 – 3.25**	**0.92**
	R^2 / R^2 adjusted	0.12 / -0.02		
	Robust Wald Test	0.5 on 3 and 19 DF, p-value = 0.68		

**Visual-motor Integration** *(standardized score, Beery VMI-6)*	Intercept	70.43	5.20 – 135.65	0.04*
	Age (years)	-0.50	-1.60 – 0.61	0.36
	Sex [Male]	-6.88	-23.39 – 9.62	0.39
	**Left Crus II / Lobule VIIB (cm^3)**	**0.61**	**-3.72 – 4.95**	**0.77**
	R^2 / R^2 adjusted	0.12 / -0.017		
	Robust Wald Test	0.4 on 3 and 19 DF, p-value = 0.74		
**Visual-motor Integration** *(standardized score, Beery VMI-6)*	Intercept	83.90	33.55 – 134.24	2.46E-03**
	Age (years)	-0.54	-1.64 – 0.56	0.32
	Sex [Male]	-5.32	-19.21 – 8.58	0.43
	**Right Lobule VIII (cm^3)**	**-0.47**	**-4.95 – 4.00**	**0.83**
	R^2 / R^2 adjusted	0.12 / -0.02		
	Robust Wald Test	0.4 on 3 and 19 DF, p-value = 0.73		
**Visual-motor Integration** *(standardized score, Beery VMI-6)*	Intercept	99.32	43.62 – 155.02	1.41E-03**
	Age (years)	-0.61	-1.72 – 0.51	0.27
	Sex [Male]	-4.82	-19.34 – 9.70	0.50
	**Left Lobule VIII (cm^3)**	**-1.97**	**-6.75 – 2.81**	**0.40**
	R^2 / R^2 adjusted	0.14 / 0.004		
	Robust Wald Test	0.7 on 3 and 19 DF, p-value = 0.58		
**Visual-motor Integration** *(standardized score, Beery VMI-6)*	Intercept	62.92	-25.80 – 151.64	0.15
	Age (years)	-0.54	-1.61 – 0.53	0.30
	Sex [Male]	-5.78	-19.84 – 8.27	0.40
	**Right Lobule IX (cm^3)**	**4.71**	**-17.71 – 27.13**	**0.66**
	R^2 / R^2 adjusted	0.13 / -0.01		
	Robust Wald Test	0.7 on 3 and 19 DF, p-value = 0.56		
**Visual-motor Integration** *(standardized score, Beery VMI-6)*	Intercept	50.96	-36.57 – 138.49	0.24
	Age (years)	-0.45	-1.57 – 0.66	0.41
	Sex [Male]	-5.59	-19.36 – 8.19	0.41
	**Left Lobule IX (cm^3)**	**7.91**	**-13.68 – 29.51**	**0.45**
	R^2 / R^2 adjusted	0.17 / 0.04		
	Robust Wald Test	1.2 on 3 and 19 DF, p-value = 0.35		
**Visual-motor Integration** *(standardized score, Beery VMI-6)*	Intercept	103.99	61.95 – 146.03	5.36E-05***
	Age (years)	-0.55	-1.55 – 0.46	0.27
	Sex [Male]	-6.12	-19.49 – 7.26	0.35
	**Right Lobule X (cm^3)**	**-42.01**	**-114.74 – 30.72**	**0.24**
	R^2 / R^2 adjusted	0.18 / 0.05		
	Robust Wald Test	1.7 on 3 and 19 DF, p-value = 0.21		
**Visual-motor Integration** *(standardized score, Beery VMI-6)*	Intercept	98.63	62.57 – 134.68	1.62E-05***
	Age (years)	-0.51	-1.60 – 0.58	0.34
	Sex [Male]	-4.88	-18.65 – 8.88	0.47
	**Left Lobule X (cm^3)**	**-36.00**	**-92.99 – 20.98**	**0.20**
	R^2 / R^2 adjusted	0.18 / 0.05		
	Robust Wald Test	1.1 on 3 and 19 DF, p-value = 0.37		
**Visual-motor Integration** *(standardized score, Beery VMI-6)*	Intercept	96.08	18.82 – 173.35	0.02*
	Age (years)	-0.61	-1.83 – 0.61	0.31
	Sex [Male]	-5.85	-20.31 – 8.61	0.41
	**Vermis I-V (cm^3)**	**-7.31**	**-38.07 – 23.45**	**0.62**
	R^2 / R^2 adjusted	0.13 / -0.01		
	Robust Wald Test	0.4 on 3 and 19 DF, p-value = 0.73		
**Visual-motor Integration** *(standardized score, Beery VMI-6)*	Intercept	128.93	76.52 – 181.35	5.71E-05***
	Age (years)	-0.43	-1.25 – 0.38	0.28
	Sex [Male]	-7.71	-20.65 – 5.22	0.23
	**Vermis VI-VII (cm^3)**	**-34.36**	**-72.89 – 4.17**	**0.08†**
	R^2 / R^2 adjusted	0.28 / 0.16		
	Robust Wald Test	3.2 on 3 and 19 DF, p-value = 0.04*		

**Visual-motor Integration** *(standardized score, Beery VMI-6)*	Intercept	86.17	24.22 – 148.11	8.96E-03**
	Age (years)	-0.55	-1.67 – 0.57	0.32
	Sex [Male]	-5.50	-19.83 – 8.84	0.43
	**Vermis VIII-X (cm^3)**	**-2.80**	**-25.32 – 19.73**	**0.80**
	R^2 / R^2 adjusted	0.12 / -0.02		
	Robust Wald Test	0.4 on 3 and 19 DF, p-value = 0.73		
**Supplemental test: Motor Coordination** *(standardized score, Beery VMI-6)*	Intercept	35.85	-35.75 – 107.46	0.31
	Age (years)	0.36	-0.68 – 1.39	0.48
	Sex [Male]	-5.99	-18.92 – 6.95	0.34
	**Right Lobules I-V (cm^3)**	**4.77**	**-6.65 – 16.19**	**0.39**
	R^2 / R^2 adjusted	0.08 / -0.06		
	Robust Wald Test	0.7 on 3 and 19 DF, p-value = 0.58		
**Supplemental test: Motor Coordination** *(standardized score, Beery VMI-6)*	Intercept	43.34	-17.53 – 104.21	0.15
	Age (years)	0.28	-0.66 – 1.23	0.54
	Sex [Male]	-3.85	-18.18 – 10.49	0.58
	**Left Lobules I-V (cm^3)**	**3.16**	**-5.41 – 11.74**	**0.45**
	R^2 / R^2 adjusted	0.07 / -0.08		
	Robust Wald Test	0.5 on 3 and 19 DF, p-value = 0.69		
**Supplemental test: Motor Coordination** *(standardized score, Beery VMI-6)*	Intercept	31.67	-14.27 – 77.62	0.17
	Age (years)	0.35	-0.46 – 1.17	0.38
	Sex [Male]	-5.66	-19.22 – 7.91	0.39
	**Right Lobule VI (cm^3)**	**3.37**	**-0.77 – 7.51**	**0.09**†
	R^2 / R^2 adjusted	0.10 / -0.04		
	Robust Wald Test	1.4 on 3 and 19 DF, p-value = 0.26		
**Supplemental test: Motor Coordination** *(standardized score, Beery VMI-6)*	Intercept	37.58	-0.87 – 76.02	0.05*
	Age (years)	0.29	-0.51 – 1.09	0.45
	Sex [Male]	-6.57	-20.69 – 7.56	0.34
	**Left Lobule VI (cm^3)**	**3.01**	**-0.84 – 6.86**	**0.10**†
	R^2 / R^2 adjusted	0.10 / -0.05		
	Robust Wald Test	1.2 on 3 and 19 DF, p-value = 0.34		
**Supplemental test: Motor Coordination** *(standardized score, Beery VMI-6)*	Intercept	54.75	-11.52 – 121.01	0.10†
	Age (years)	0.25	-0.73 – 1.22	0.60
	Sex [Male]	-4.64	-19.18 – 9.90	0.51
	**Right Crus I (cm^3)**	**0.50**	**-3.45 – 4.45**	**0.79**
	R^2 / R^2 adjusted	0.04 / -0.11		
	Robust Wald Test	0.3 on 3 and 19 DF, p-value = 0.83		
**Supplemental test: Motor Coordination** *(standardized score, Beery VMI-6)*	Intercept	60.12	-13.16 – 133.40	0.10†
	Age (years)	0.22	-0.79 – 1.23	0.65
	Sex [Male]	-4.57	-19.90 – 10.76	0.54
	**Left Crus I (cm^3)**	**0.15**	**-4.55 – 4.84**	**0.95**
	R^2 / R^2 adjusted	0.04 / -0.11		
	Robust Wald Test	0.3 on 3 and 19 DF, p-value = 0.83		
**Supplemental test: Motor Coordination** *(standardized score, Beery VMI-6)*	Intercept	58.09	-1.79 – 117.97	0.06†
	Age (years)	0.24	-0.67 – 1.15	0.59
	Sex [Male]	-4.72	-19.69 – 10.25	0.52
	**Right Crus II / Lobule VIIB (cm^3)**	**0.27**	**-3.51 – 4.05**	**0.88**
	R^2 / R^2 adjusted	0.04 / -0.11		
	Robust Wald Test	0.3 on 3 and 19 DF, p-value = 0.82		
**Supplemental test: Motor Coordination** *(standardized score, Beery VMI-6)*	Intercept	46.27	-25.91 – 118.45	0.20
	Age (years)	0.29	-0.69 – 1.26	0.55
	Sex [Male]	-6.38	-22.09 – 9.33	0.41
	**Left Crus II / Lobule VIIB (cm^3)**	**1.08**	**-3.53 – 5.69**	**0.63**
	R^2 / R^2 adjusted	0.06 / -0.09		
	Robust Wald Test	0.4 on 3 and 19 DF, p-value = 0.74		

**Supplemental test: Motor Coordination** *(standardized score, Beery VMI-6)*	Intercept	55.14	6.03 – 104.24	0.03*
	Age (years)	0.21	-0.66 – 1.07	0.62
	Sex [Male]	-5.20	-19.81 – 9.41	0.47
	**Right Lobule VIII (cm^3)**	**0.76**	**-3.59 – 5.11**	**0.72**
	R^2 / R^2 adjusted	0.04 / -0.11		
	Robust Wald Test	0.3 on 3 and 19 DF, p-value = 0.82		
**Supplemental test: Motor Coordination** *(standardized score, Beery VMI-6)*	Intercept	71.78	7.87 – 135.69	0.03*
	Age (years)	0.18	-0.76 – 1.12	0.70
	Sex [Male]	-3.98	-18.73 – 10.77	0.58
	**Left Lobule VIII (cm^3)**	**-0.95**	**-6.66 – 4.75**	**0.73**
	R^2 / R^2 adjusted	0.04 / -0.11		
	Robust Wald Test	0.4 on 3 and 19 DF, p-value = 0.77		
**Supplemental test: Motor Coordination** *(standardized score, Beery VMI-6)*	Intercept	49.03	-34.89 – 132.95	0.24
	Age (years)	0.21	-0.66 – 1.07	0.62
	Sex [Male]	-4.45	-18.81 – 9.92	0.52
	**Right Lobule IX (cm^3)**	**3.75**	**-18.24 – 25.73**	**0.72**
	R^2 / R^2 adjusted	0.05 / -0.10		
	Robust Wald Test	0.3 on 3 and 19 DF, p-value = 0.83		
**Supplemental test: Motor Coordination** *(standardized score, Beery VMI-6)*	Intercept	46.48	-24.70 – 117.66	0.19
	Age (years)	0.26	-0.65 – 1.16	0.56
	Sex [Male]	-4.34	-18.66 – 9.98	0.53
	**Left Lobule IX (cm^3)**	**4.36**	**-13.81 – 22.54**	**0.62**
	R^2 / R^2 adjusted	0.06 / -0.09		
	Robust Wald Test	0.3 on 3 and 19 DF, p-value = 0.82		
**Supplemental test: Motor Coordination** *(standardized score, Beery VMI-6)*	Intercept	77.15	36.99 – 117.32	7.31E-04***
	Age (years)	0.21	-0.62 – 1.03	0.61
	Sex [Male]	-4.65	-18.76 – 9.46	0.50
	**Right Lobule X (cm^3)**	**-25.63**	**-91.07 – 39.81**	**0.42**
	R^2 / R^2 adjusted	0.06 / -0.08		
	Robust Wald Test	0.6 on 3 and 19 DF, p-value = 0.63		
**Supplemental test: Motor Coordination** *(standardized score, Beery VMI-6)*	Intercept	71.31	36.53 – 106.09	3.94E-04***
	Age (years)	0.22	-0.65 – 1.10	0.60
	Sex [Male]	-4.02	-18.40 – 10.36	0.56
	**Left Lobule X (cm^3)**	**-17.13**	**-74.68 – 40.42**	**0.54**
	R^2 / R^2 adjusted	0.06 / -0.09		
	Robust Wald Test	0.4 on 3 and 19 DF, p-value = 0.72		
**Supplemental test: Motor Coordination** *(standardized score, Beery VMI-6)*	Intercept	44.55	-38.99 – 128.09	0.28
	Age (years)	0.28	-0.69 – 1.25	0.55
	Sex [Male]	-4.38	-18.63 – 9.86	0.53
	**Vermis I-V (cm^3)**	**7.78**	**-26.21 – 41.77**	**0.64**
	R^2 / R^2 adjusted	0.06 / -0.09		
	Robust Wald Test	0.3 on 3 and 19 DF, p-value = 0.80		
**Supplemental test: Motor Coordination** *(standardized score, Beery VMI-6)*	Intercept	85.44	28.00 – 142.89	5.73E-03**
	Age (years)	0.26	-0.51 – 1.03	0.49
	Sex [Male]	-5.35	-20.13 – 9.42	0.46
	**Vermis VI-VII (cm^3)**	**-16.15**	**-54.75 – 22.45**	**0.39**
	R^2 / R^2 adjusted	0.08 / -0.07		
	Robust Wald Test	0.4 on 3 and 19 DF, p-value = 0.72		
**Supplemental test: Motor Coordination** *(standardized score, Beery VMI-6)*	Intercept	57.65	-8.42 – 123.73	0.08
	Age (years)	0.22	-0.69 – 1.12	0.62
	Sex [Male]	-4.65	-19.51 – 10.22	0.52
	**Vermis VIII-X (cm^3)**	**1.91**	**-22.52 – 26.33**	**0.87**
	R^2 / R^2 adjusted	0.04 / -0.11		
	Robust Wald Test	0.3 on 3 and 19 DF, p-value = 0.83		

**Supplemental test: Visual Perception** *(standardized score, Beery VMI-6)*	Intercept	112.72	45.79 – 179.64	2.26E-03**
	Age (years)	-0.65	-1.71 – 0.41	0.21
	Sex [Male]	-5.60	-22.42 – 11.22	0.49
	**Right Lobules I-V (cm^3)**	**-4.71**	**-16.51 – 7.09**	**0.41**
	R^2 / R^2 adjusted	0.12 / -0.02		
	Robust Wald Test	0.8 on 3 and 19 DF, p-value = 0.51		
**Supplemental test: Visual Perception** *(standardized score, Beery VMI-6)*	Intercept	88.08	22.58 – 153.58	0.01**
	Age (years)	-0.51	-1.55 – 0.53	0.32
	Sex [Male]	-7.17	-24.85 – 10.52	0.41
	**Left Lobules I-V (cm^3)**	**-0.23**	**-10.26 – 9.80**	**0.96**
	R^2 / R^2 adjusted	0.91 / -0.05		
	Robust Wald Test	0.5 on 3 and 19 DF, p-value = 0.65		
**Supplemental test: Visual Perception** *(standardized score, Beery VMI-6)*	Intercept	69.21	1.82 – 136.61	0.04*
	Age (years)	-0.42	-1.36 – 0.51	0.36
	Sex [Male]	-7.82	-24.17 – 8.54	0.33
	**Right Lobule VI (cm^3)**	**1.93**	**-5.21 – 9.07**	**0.58**
	R^2 / R^2 adjusted	0.11 / -0.04		
	Robust Wald Test	0.8 on 3 and 19 DF, p-value = 0.53		
**Supplemental test: Visual Perception** *(standardized score, Beery VMI-6)*	Intercept	66.13	12.05 – 120.22	0.02*
	Age (years)	-0.44	-1.32 – 0.45	0.31
	Sex [Male]	-8.89	-25.04 – 7.26	0.26
	**Left Lobule VI (cm^3)**	**2.51**	**-3.71 – 8.74**	**0.41**
	R^2 / R^2 adjusted	0.12 / -0.02		
	Robust Wald Test	1.0 on 3 and 19 DF, p-value = 0.40		
**Supplemental test: Visual Perception** *(standardized score, Beery VMI-6)*	Intercept	54.09	0.99 – 107.19	0.05*
	Age (years)	-0.33	-1.35 – 0.70	0.51
	Sex [Male]	-7.96	-24.52 – 8.61	0.33
	**Right Crus I (cm^3)**	**2.18**	**-1.13 – 5.49**	**0.18**
	R^2 / R^2 adjusted	0.14 / 0.01		
	Robust Wald Test	1.3 on 3 and 19 DF, p-value = 0.29		
**Supplemental test: Visual Perception** *(standardized score, Beery VMI-6)*	Intercept	60.60	4.36 – 116.83	0.04*
	Age (years)	-0.36	-1.34 – 0.63	0.45
	Sex [Male]	-8.63	-25.59 – 8.33	0.30
	**Left Crus I (cm^3)**	**1.83**	**-2.05 – 5.72**	**0.34**
	R^2 / R^2 adjusted	0.12 / -0.02		
	Robust Wald Test	0.9 on 3 and 19 DF, p-value = 0.44		
**Supplemental test: Visual Perception** *(standardized score, Beery VMI-6)*	Intercept	82.60	-6.61 – 171.81	0.07†
	Age (years)	-0.48	-1.76 – 0.80	0.45
	Sex [Male]	-7.39	-24.59 – 9.80	0.38
	**Right Crus II / Lobule VIIB (cm^3)**	**0.27**	**-5.27 – 5.81**	**0.92**
	R^2 / R^2 adjusted	0.09 / -0.05		
	Robust Wald Test	0.6 on 3 and 19 DF, p-value = 0.63		
**Supplemental test: Visual Perception** *(standardized score, Beery VMI-6)*	Intercept	92.69	12.34 – 173.04	0.03*
	Age (years)	-0.53	-1.65 – 0.58	0.33
	Sex [Male]	-6.40	-25.12 – 12.32	0.48
	**Left Crus II / Lobule VIIB (cm^3)**	**-0.41**	**-5.54 – 4.73**	**0.87**
	R^2 / R^2 adjusted	0.09 / -0.05		
	Robust Wald Test	0.6 on 3 and 19 DF, p-value = 0.64		
**Supplemental test: Visual Perception** *(standardized score, Beery VMI-6)*	Intercept	113.87	66.67 – 161.07	7.12EE-05***
	Age (years)	-0.50	-1.55 – 0.56	0.34
	Sex [Male]	-4.22	-20.56 – 12.12	0.60
	**Right Lobule VIII (cm^3)**	**-2.91**	**-7.72 – 1.90**	**0.22**
	R^2 / R^2 adjusted	0.15 / 0.01		
	Robust Wald Test	1.2 on 3 and 19 DF, p-value = 0.35		

**Supplemental test: Visual Perception** *(standardized score, Beery VMI-6)*	Intercept	116.76	41.30 – 192.22	4.32E-03**
	Age (years)	-0.60	-1.62 – 0.41	0.23
	Sex [Male]	-5.66	-22.01 – 10.70	0.48
	**Left Lobule VIII (cm^3)**	**-2.99**	**-10.40 – 4.42**	**0.41**
	R^2 / R^2 adjusted	0.13 / -0.005		
	Robust Wald Test	0.9 on 3 and 19 DF, p-value = 0.45		
**Supplemental test: Visual Perception** *(standardized score, Beery VMI-6)*	Intercept	106.75	27.53 – 185.97	0.01**
	Age (years)	-0.50	-1.55 – 0.54	0.32
	Sex [Male]	-7.13	-23.95 – 9.70	0.39
	**Right Lobule IX (cm^3)**	**-5.70**	**-26.92 – 15.52**	**0.58**
	R^2 / R^2 adjusted	0.10 / -0.04		
	Robust Wald Test	0.6 on 3 and 19 DF, p-value = 0.62		
**Supplemental test: Visual Perception** *(standardized score, Beery VMI-6)*	Intercept	79.96	4.90 – 155.01	0.04*
	Age (years)	-0.48	-1.54 – 0.57	0.35
	Sex [Male]	-7.08	-24.24 – 10.09	0.40
	**Left Lobule IX (cm^3)**	**1.87**	**-16.63 – 20.36**	**0.84**
	R^2 / R^2 adjusted	0.09 / -0.05		
	Robust Wald Test	0.7 on 3 and 19 DF, p-value = 0.59		
**Supplemental test: Visual Perception** *(standardized score, Beery VMI-6)*	Intercept	112.10	47.32 – 176.89	1.82E-03**
	Age (years)	-0.51	-1.40 – 0.38	0.25
	Sex [Male]	-7.47	-23.95 – 9.02	0.36
	**Right Lobule X (cm^3)**	**-43.56**	**-150.75 – 63.62**	**0.41**
	R^2 / R^2 adjusted	0.14 / 0.01		
	Robust Wald Test	0.8 on 3 and 19 DF, p-value = 0.49		
**Supplemental test: Visual Perception** *(standardized score, Beery VMI-6)*	Intercept	101.09	49.99 – 152.19	5.56E-04***
	Age (years)	-0.48	-1.49 – 0.53	0.33
	Sex [Male]	-6.45	-22.49 – 9.60	0.41
	**Left Lobule X (cm^3)**	**-27.08**	**-112.80 – 58.64**	**0.52**
	R^2 / R^2 adjusted	0.12 / -0.02		
	Robust Wald Test	0.6 on 3 and 19 DF, p-value = 0.61		
**Supplemental test: Visual Perception** *(standardized score, Beery VMI-6)*	Intercept	110.33	38.05 – 182.61	4.77E-03**
	Age (years)	-0.60	-1.73 – 0.53	0.28
	Sex [Male]	-7.21	-24.33 – 9.91	0.39
	**Vermis I-V (cm^3)**	**-10.41**	**-38.47 – 17.65**	**0.45**
	R^2 / R^2 adjusted	0.11 / -0.03		
	Robust Wald Test	0.6 on 3 and 19 DF, p-value = 0.65		
**Supplemental test: Visual Perception** *(standardized score, Beery VMI-6)*	Intercept	153.39	94.97 – 211.82	2.66E-05***
	Age (years)	-0.36	-0.97 – 0.24	0.21
	Sex [Male]	-9.73	-24.96 – 5.51	0.20
	**Vermis VI-VII (cm^3)**	**-46.35**	**-88.20 – -4.50**	**0.03***
	R^2 / R^2 adjusted	0.31 / 0.21		
	Robust Wald Test	3.5 on 3 and 19 DF, p-value = 0.04*		
**Supplemental test: Visual Perception** *(standardized score, Beery VMI-6)*	Intercept	87.53	20.61 – 154.44	0.01**
	Age (years)	-0.51	-1.52 – 0.51	0.31
	Sex [Male]	-7.09	-23.89 – 9.72	0.39
	**Vermis VIII-X (cm^3)**	**-0.35**	**-26.78 – 26.09**	**0.98**
	R^2 / R^2 adjusted	0.09 / -0.05		
	Robust Wald Test	0.6 on 3 and 19 DF, p-value = 0.65		

Secondary models for subregions that were found to have a significant main effect on sensorimotor abilities ($p \leq 0.05$), after eICV is included as an additional covariate:

Outcome variable	Explanatory variables	*b*	CI (95%)	p-value
**Supplemental test: Visual Perception** *(standardized score, Beery VMI-6)*	Intercept	63.00	-61.52 – 187.53	0.30
	Age (years)	-0.15	-0.68 – 0.37	0.55
	Sex [Male]	-12.75	-26.83 – 1.32	0.07†
	eICV (cm^3)	0.06	0.004 – 0.12	0.04*
	**Vermis VI-VII (cm^3)**	**-46.29**	**-85.64 – -6.94**	**0.02***
	R^2 / R^2 adjusted	0.46 / 0.33		
	Robust Wald Test	7.1 on 3 and 19 DF, p-value = 1.31E-03**		

Contrast coding: reference level for sex is female. p-value ≤ 0.001‘***’, p-value ≤ 0.01‘**’, p-value ≤ 0.05‘*’, p-value ≤ 0.1‘†’. *Abbreviations*: 3q29 deletion syndrome, 3q29Del; visual-motor integration, VMI; unstandardized coefficient estimate, *b*; confidence interval, CI; degrees of freedom, DF; estimated total intracranial volume, eICV.

Table S16. Multiple linear regression results testing the relationships between ACAPULCO-based subregional cerebellar cortex volumes and cognitive abilities among 3q29Del participants. Main effects of ACAPULCO-based subregional cerebellar cortex volumes are reported in bold. Regression parameters reflect heteroskedasticity-robust estimates. Robust Wald test statistics are reported to assess the overall significance of each model. Results indicate significant inverse relationships between vermis VI-VII volumes and both standardized composite IQ scores and non-verbal IQ scores among 3q29Del participants, while correcting for age and sex (*p's* ≤ 0.05). These effects persisted after both eICV, and visual perception scores were included as additional covariates in secondary models (*p's* ≤ 0.05). In addition, a trend level positive relationship was identified between left Crus II / Lobule VIIB volumes and verbal IQ scores, while correcting for age and sex (*p's* ≤ 0.10). 3q29Del *N* = 23.

Outcome variable	Explanatory variables	*b*	CI (95%)	p-value
**Composite IQ** *(standardized score, WASI-II/ DAS-II)*	Intercept	66.44	7.62 – 125.26	0.03*
	Age (years)	0.05	-0.91 – 1.01	0.91
	Sex [Male]	-4.71	-17.58 – 8.17	0.45
	**Right Lobules I-V (cm^3)**	**1.73**	**-7.91 – 11.38**	**0.71**
	R^2 / R^2 adjusted	0.03 / -0.12		
	Robust Wald Test	0.2 on 3 and 19 DF, p-value = 0.89		
**Composite IQ** *(standardized score, WASI-II/ DAS-II)*	Intercept	74.82	27.89 – 121.76	3.47E-03**
	Age (years)	0.003	-0.89 – 0.90	0.99
	Sex [Male]	-4.11	-15.57 – 7.36	0.46
	**Left Lobules I-V (cm^3)**	**0.20**	**-6.56 – 6.96**	**0.95**
	R^2 / R^2 adjusted	0.02 / -0.13		
	Robust Wald Test	0.2 on 3 and 19 DF, p-value = 0.89		
**Composite IQ** *(standardized score, WASI-II/ DAS-II)*	Intercept	61.97	10.50 – 113.43	0.02*
	Age (years)	0.07	-0.81 – 0.94	0.88
	Sex [Male]	-4.70	-16.22 – 6.81	0.40
	**Right Lobule VI (cm^3)**	**1.55**	**-3.31 – 6.41**	**0.51**
	R^2 / R^2 adjusted	0.04 / -0.11		
	Robust Wald Test	0.4 on 3 and 19 DF, p-value = 0.77		
**Composite IQ** *(standardized score, WASI-II/ DAS-II)*	Intercept	42.14	-6.70 – 90.99	0.09†
	Age (years)	0.11	-0.64 – 0.86	0.76
	Sex [Male]	-7.06	-18.53 – 4.41	0.21
	**Left Lobule VI (cm^3)**	**4.14**	**-1.43 – 9.72**	**0.14**
	R^2 / R^2 adjusted	0.17 / 0.04		
	Robust Wald Test	1.0 on 3 and 19 DF, p-value = 0.42		
**Composite IQ** *(standardized score, WASI-II/ DAS-II)*	Intercept	56.47	-3.75 – 116.69	0.06†
	Age (years)	0.11	-0.83 – 1.04	0.82
	Sex [Male]	-4.65	-15.44 – 6.14	0.38
	**Right Crus I (cm^3)**	**1.31**	**-2.38 – 5.00**	**0.47**
	R^2 / R^2 adjusted	0.06 / -0.09		
	Robust Wald Test	0.5 on 3 and 19 DF, p-value = 0.70		
**Composite IQ** *(standardized score, WASI-II/ DAS-II)*	Intercept	45.55	-10.96 – 102.06	0.11
	Age (years)	0.17	-0.70 – 1.04	0.69
	Sex [Male]	-5.91	-16.80 – 4.99	0.27
	**Left Crus I (cm^3)**	**2.14**	**-1.53 – 5.81**	**0.24**
	R^2 / R^2 adjusted	0.10 / -0.04		
	Robust Wald Test	0.8 on 3 and 19 DF, p-value = 0.53		
**Composite IQ** *(standardized score, WASI-II/ DAS-II)*	Intercept	59.57	12.42 – 106.72	0.02*
	Age (years)	0.11	-0.79 – 1.02	0.80
	Sex [Male]	-5.23	-18.16 – 7.69	0.41
	**Right Crus II / Lobule VIIB (cm^3)**	**1.09**	**-1.92 – 4.09**	**0.46**
	R^2 / R^2 adjusted	0.05 / -0.10		
	Robust Wald Test	0.3 on 3 and 19 DF, p-value = 0.82		

**Composite IQ** *(standardized score, WASI-II/ DAS-II)*	Intercept	47.54	-7.98 – 103.07	0.09†
	Age (years)	0.13	-0.76 – 1.03	0.76
	Sex [Male]	-7.60	-20.83 – 5.63	0.24
	**Left Crus II / Lobule VIIB (cm^3)**	**1.93**	**-1.73 – 5.59**	**0.28**
	R^2 / R^2 adjusted	0.09 / -0.05		
	Robust Wald Test	0.7 on 3 and 19 DF, p-value = 0.58		
**Composite IQ** *(standardized score, WASI-II/ DAS-II)*	Intercept	56.15	7.99 – 104.31	0.03*
	Age (years)	-0.01	-0.84 – 0.82	0.98
	Sex [Male]	-6.27	-19.32 – 6.78	0.33
	**Right Lobule VIII (cm^3)**	**2.13**	**-2.74 – 7.00**	**0.37**
	R^2 / R^2 adjusted	0.08 / -0.06		
	Robust Wald Test	0.4 on 3 and 19 DF, p-value = 0.73		
**Composite IQ** *(standardized score, WASI-II/ DAS-II)*	Intercept	52.73	-3.10 – 108.55	0.06†
	Age (years)	0.08	-0.83 – 0.98	0.86
	Sex [Male]	-5.28	-17.62 – 7.05	0.38
	**Left Lobule VIII (cm^3)**	**2.32**	**-2.98 – 7.62**	**0.37**
	R^2 / R^2 adjusted	0.07 / -0.08		
	Robust Wald Test	0.4 on 3 and 19 DF, p-value = 0.76		
**Composite IQ** *(standardized score, WASI-II/ DAS-II)*	Intercept	61.98	-7.45 – 131.42	0.08†
	Age (years)	-0.002	-0.83 – 0.82	0.99
	Sex [Male]	-4.14	-15.92 – 7.63	0.47
	**Right Lobule IX (cm^3)**	**3.99**	**-14.92 – 22.90**	**0.66**
	R^2 / R^2 adjusted	0.04 / -0.12		
	Robust Wald Test	0.2 on 3 and 19 DF, p-value = 0.86		
**Composite IQ** *(standardized score, WASI-II/ DAS-II)*	Intercept	50.74	-11.18 – 112.66	0.10†
	Age (years)	0.08	-0.71 – 0.86	0.84
	Sex [Male]	-3.97	-15.43 – 7.49	0.48
	**Left Lobule IX (cm^3)**	**7.01**	**-8.93 – 22.96**	**0.37**
	R^2 / R^2 adjusted	0.08 / -0.06		
	Robust Wald Test	0.5 on 3 and 19 DF, p-value = 0.71		
**Composite IQ** *(standardized score, WASI-II/ DAS-II)*	Intercept	85.16	39.75 – 130.56	9.09E-04***
	Age (years)	-0.003	-0.82 – 0.81	0.99
	Sex [Male]	-4.27	-15.66 – 7.12	0.44
	**Right Lobule X (cm^3)**	**-15.66**	**-89.61 – 58.30**	**0.66**
	R^2 / R^2 adjusted	0.04 / -0.12		
	Robust Wald Test	0.4 on 3 and 19 DF, p-value = 0.76		
**Composite IQ** *(standardized score, WASI-II/ DAS-II)*	Intercept	77.77	41.91 – 113.63	2.24E-04***
	Age (years)	0.001	-0.84 – 0.84	0.99
	Sex [Male]	-4.06	-16.04 – 7.91	0.49
	**Left Lobule X (cm^3)**	**-3.29**	**-59.26 – 52.69**	**0.90**
	R^2 / R^2 adjusted	0.03 / -0.13		
	Robust Wald Test	1.1 on 3 and 19 DF, p-value = 0.37		
**Composite IQ** *(standardized score, WASI-II/ DAS-II))*	Intercept	66.02	4.68 – 127.36	0.04*
	Age (years)	0.04	-0.88 – 0.96	0.93
	Sex [Male]	-4.11	-15.61 – 7.39	0.46
	**Vermis I-V (cm^3)**	**4.41**	**-19.65 – 28.46**	**0.71**
	R^2 / R^2 adjusted	0.03 / -0.12		
	Robust Wald Test	0.2 on 3 and 19 DF, p-value = 0.86		
**Composite IQ** *(standardized score, WASI-II/ DAS-II)*	Intercept	127.55	87.57 – 167.52	2.19E-06***
	Age (years)	0.11	-0.48 – 0.69	0.70
	Sex [Male]	-6.16	-16.18 – 3.86	0.21
	**Vermis VI-VII (cm^3)**	**-35.80**	**-62.36 – -9.25**	**0.01****
	R^2 / R^2 adjusted	0.27 / 0.16		
	Robust Wald Test	2.9 on 3 and 19 DF, p-value = 0.06*		

**Composite IQ** *(standardized score, WASI-II/ DAS-II)*	Intercept	58.55	2.79 – 114.30	0.04*
	Age (years)	0.02	-0.83 – 0.87	0.96
	Sex [Male]	-4.91	-16.94 – 7.13	0.40
	**Vermis VIII-X (cm^3)**	**7.32**	**-14.63 – 29.26**	**0.49**
	R^2 / R^2 adjusted	0.06 / -0.09		
	Robust Wald Test	0.3 on 3 and 19 DF, p-value = 0.82		
**Supplemental test: Verbal IQ** *(standardized score, WASI-II/ DAS-II)*	Intercept	39.34	-33.96 – 112.65	0.28
	Age (years)	0.37	-0.68 – 1.43	0.47
	Sex [Male]	-8.94	-24.73 – 6.85	0.25
	**Right Lobules I-V (cm^3)**	**7.68**	**-4.71 – 20.07**	**0.21**
	R^2 / R^2 adjusted	0.11 / -0.03		
	Robust Wald Test	0.7 on 3 and 19 DF, p-value = 0.54		
**Supplemental test: Verbal IQ** *(standardized score, WASI-II/ DAS-II)*	Intercept	63.27	10.98 – 115.56	0.02*
	Age (years)	0.21	-0.71 – 1.12	0.64
	Sex [Male]	-5.87	-21.00 – 9.26	0.43
	**Left Lobules I-V (cm^3)**	**3.10**	**-5.04 – 11.25**	**0.44**
	R^2 / R^2 adjusted	0.06 / -0.09		
	Robust Wald Test	0.5 on 3 and 19 DF, p-value = 0.71		
**Supplemental test: Verbal IQ** *(standardized score, WASI-II/ DAS-II)*	Intercept	60.21	-10.46 – 130.89	0.09†
	Age (years)	0.24	-0.62 – 1.09	0.57
	Sex [Male]	-7.31	-23.57 – 8.95	0.36
	**Right Lobule VI (cm^3)**	**2.38**	**-5.35 – 10.11**	**0.53**
	R^2 / R^2 adjusted	0.06 / -0.09		
	Robust Wald Test	0.4 on 3 and 19 DF, p-value = 0.79		
**Supplemental test: Verbal IQ** *(standardized score, WASI-II/ DAS-II)*	Intercept	38.95	-37.95 – 115.85	0.30
	Age (years)	0.28	-0.59 – 1.15	0.51
	Sex [Male]	-10.14	-27.01 – 6.73	0.22
	**Left Lobule VI (cm^3)**	**5.23**	**-4.10 – 14.57**	**0.26**
	R^2 / R^2 adjusted	0.15 / 0.01		
	Robust Wald Test	0.7 on 3 and 19 DF, p-value = 0.57		
**Supplemental test: Verbal IQ** *(standardized score, WASI-II/ DAS-II)*	Intercept	87.13	-0.71 – 174.97	0.05*
	Age (years)	0.11	-0.93 – 1.14	0.83
	Sex [Male]	-6.32	-21.57 – 8.93	0.40
	**Right Crus I (cm^3)**	**-0.36**	**-6.05 – 5.33**	**0.90**
	R^2 / R^2 adjusted	0.04 / -0.12		
	Robust Wald Test	0.3 on 3 and 19 DF, p-value = 0.83		
**Supplemental test: Verbal IQ** *(standardized score, WASI-II/ DAS-II)*	Intercept	73.98	-13.39 – 161.36	0.09†
	Age (years)	0.18	-0.82 – 1.17	0.71
	Sex [Male]	-6.91	-23.14 – 9.32	0.38
	**Left Crus I (cm^3)**	**0.55**	**-5.43 – 6.53**	**0.85**
	R^2 / R^2 adjusted	0.04 / -0.11		
	Robust Wald Test	0.3 on 3 and 19 DF, p-value = 0.82		
**Supplemental test: Verbal IQ** *(standardized score, WASI-II/ DAS-II)*	Intercept	40.05	-23.71 – 103.81	0.20
	Age (years)	0.43	-0.61 – 1.46	0.40
	Sex [Male]	-9.22	-26.15 – 7.72	0.27
	**Right Crus II / Lobule VIIB (cm^3)**	**2.75**	**-1.27 – 6.78**	**0.17**
	R^2 / R^2 adjusted	0.10 / -0.04		
	Robust Wald Test	0.8 on 3 and 19 DF, p-value = 0.53		
**Supplemental test: Verbal IQ** *(standardized score, WASI-II/ DAS-II)*	Intercept	24.08	-47.34 – 95.50	0.49
	Age (years)	0.41	-0.59 – 1.42	0.40
	Sex [Male]	-13.45	-30.53 – 3.63	0.12
	**Left Crus II / Lobule VIIB (cm^3)**	**3.91**	**-0.71 – 8.54**	**0.09†**
	R^2 / R^2 adjusted	0.17 / 0.04		
	Robust Wald Test	1.3 on 3 and 19 DF, p-value = 0.29		

**Supplemental test: Verbal IQ** *(standardized score, WASI-II/ DAS-II)*	Intercept	41.73	-24.81 – 108.26	0.20
	Age (years)	0.12	-0.78 – 1.02	0.78
	Sex [Male]	-10.74	-28.95 – 7.47	0.23
	**Right Lobule VIII (cm^3)**	**4.29**	**-2.60 – 11.18**	**0.21**
	R^2 / R^2 adjusted	0.15 / 0.01		
	Robust Wald Test	0.7 on 3 and 19 DF, p-value = 0.57		
**Supplemental test: Verbal IQ** *(standardized score, WASI-II/ DAS-II)*	Intercept	26.23	-46.85 – 99.30	0.46
	Age (years)	0.32	-0.69 – 1.32	0.52
	Sex [Male]	-9.17	-24.53 – 6.19	0.23
	**Left Lobule VIII (cm^3)**	**5.53**	**-1.53 – 12.58**	**0.12**
	R^2 / R^2 adjusted	0.16 / 0.03		
	Robust Wald Test	1.2 on 3 and 19 DF, p-value = 0.34		
**Supplemental test: Verbal IQ** *(standardized score, WASI-II/ DAS-II)*	Intercept	33.38	-53.89 – 120.65	0.43
	Age (years)	0.13	-0.72 – 0.99	0.75
	Sex [Male]	-6.45	-21.70 – 8.79	0.39
	**Right Lobule IX (cm^3)**	**13.76**	**-11.33 – 38.84**	**0.26**
	R^2 / R^2 adjusted	0.11 / -0.03		
	Robust Wald Test	0.6 on 3 and 19 DF, p-value = 0.63		
**Supplemental test: Verbal IQ** *(standardized score, WASI-II/ DAS-II)*	Intercept	42.57	-24.01 – 109.14	0.20
	Age (years)	0.25	-0.50 – 1.01	0.49
	Sex [Male]	-6.19	-21.02 – 8.65	0.39
	**Left Lobule IX (cm^3)**	**10.87**	**-7.64 – 29.39**	**0.23**
	R^2 / R^2 adjusted	0.10 / -0.04		
	Robust Wald Test	0.7 on 3 and 19 DF, p-value = 0.57		
**Supplemental test: Verbal IQ** *(standardized score, WASI-II/ DAS-II)*	Intercept	68.87	15.27 – 122.47	0.01**
	Age (years)	0.14	-0.77 – 1.05	0.76
	Sex [Male]	-6.29	-21.39 – 8.82	0.40
	**Right Lobule X (cm^3)**	**22.10**	**-63.02 – 107.22**	**0.59**
	R^2 / R^2 adjusted	0.05 / -0.10		
	Robust Wald Test	0.3 on 3 and 19 DF, p-value = 0.80		
**Supplemental test: Verbal IQ** *(standardized score, WASI-II/ DAS-II)*	Intercept	68.64	25.71 – 111.58	3.39E-03**
	Age (years)	0.11	-0.78 – 1.01	0.79
	Sex [Male]	-7.08	-22.43 – 8.27	0.35
	**Left Lobule X (cm^3)**	**24.68**	**-45.95 – 95.30**	**0.47**
	R^2 / R^2 adjusted	0.06 / -0.09		
	Robust Wald Test	0.4 on 3 and 19 DF, p-value = 0.73		
**Supplemental test: Verbal IQ** *(standardized score, WASI-II/ DAS-II)*	Intercept	42.12	-16.71 – 100.95	0.15
	Age (years)	0.30	-0.62 – 1.22	0.50
	Sex [Male]	-6.31	-21.06 – 8.44	0.38
	**Vermis I-V (cm^3)**	**17.46**	**-5.63 – 40.55**	**0.13**
	R^2 / R^2 adjusted	0.09 / -0.05		
	Robust Wald Test	1.3 on 3 and 19 DF, p-value = 0.30		
**Supplemental test: Verbal IQ** *(standardized score, WASI-II/ DAS-II)*	Intercept	123.20	57.61 – 188.79	8.96E-04***
	Age (years)	0.22	-0.51 – 0.96	0.53
	Sex [Male]	-8.08	-23.83 – 7.68	0.30
	**Vermis VI-VII (cm^3)**	**-28.80**	**-71.23 – 13.63**	**0.17**
	R^2 / R^2 adjusted	0.11 / -0.03		
	Robust Wald Test	1.1 on 3 and 19 DF, p-value = 0.36		
**Supplemental test: Verbal IQ** *(standardized score, WASI-II/ DAS-II)*	Intercept	53.22	-8.52 – 114.95	0.09†
	Age (years)	0.17	-0.72 – 1.07	0.69
	Sex [Male]	-7.70	-23.41 – 8.00	0.32
	**Vermis VIII-X (cm^3)**	**11.95**	**-12.92 – 36.83**	**0.33**
	R^2 / R^2 adjusted	0.08 / -0.07		
	Robust Wald Test	0.5 on 3 and 19 DF, p-value = 0.66		

**Supplemental test: Non-verbal IQ** *(standardized score, WASI-II/ DAS-II)*	Intercept	98.27	43.47 – 153.06	1.35E-03**
	Age (years)	-0.56	-1.66 – 0.54	0.30
	Sex [Male]	-5.29	-18.32 – 7.75	0.41
	**Right Lobules I-V (cm^3)**	**-2.28**	**-11.18 – 6.62**	**0.60**
	R^2 / R^2 adjusted	0.15 / 0.01		
	Robust Wald Test	0.5 on 3 and 19 DF, p-value = 0.68		
**Supplemental test: Non-verbal IQ** *(standardized score, WASI-II/ DAS-II)*	Intercept	92.72	44.38 – 141.05	7.41E-04***
	Age (years)	-0.52	-1.61 – 0.57	0.33
	Sex [Male]	-6.25	-19.28 – 6.79	0.33
	**Left Lobules I-V (cm^3)**	**-1.18**	**-7.66 – 5.29**	**0.71**
	R^2 / R^2 adjusted	0.14 / 0.01		
	Robust Wald Test	0.3 on 3 and 19 DF, p-value = 0.73		
**Supplemental test: Non-verbal IQ** *(standardized score, WASI-II/ DAS-II)*	Intercept	74.65	13.16 – 136.14	0.02*
	Age (years)	-0.44	-1.55 – 0.67	0.42
	Sex [Male]	-6.46	-18.12 – 5.20	0.26
	**Right Lobule VI (cm^3)**	**1.21**	**-4.16 – 6.58**	**0.64**
	R^2 / R^2 adjusted	0.15 / 0.01		
	Robust Wald Test	1.3 on 3 and 19 DF, p-value = 0.31		
**Supplemental test: Non-verbal IQ** *(standardized score, WASI-II/ DAS-II)*	Intercept	57.73	7.94 – 107.52	0.03*
	Age (years)	-0.40	-1.32 – 0.53	0.38
	Sex [Male]	-8.43	-19.73 – 2.88	0.14
	**Left Lobule VI (cm^3)**	**3.42**	**-1.51 – 8.34**	**0.16**
	R^2 / R^2 adjusted	0.23 / 0.11		
	Robust Wald Test	3.1 on 3 and 19 DF, p-value = 0.05*		
**Supplemental test: Non-verbal IQ** *(standardized score, WASI-II/ DAS-II)*	Intercept	61.11	5.37 – 116.85	0.03*
	Age (years)	-0.36	-1.45 – 0.74	0.50
	Sex [Male]	-6.65	-18.00 – 4.70	0.23
	**Right Crus I (cm^3)**	**1.64**	**-1.53 – 4.82**	**0.29**
	R^2 / R^2 adjusted	0.19 / 0.06		
	Robust Wald Test	1.6 on 3 and 19 DF, p-value = 0.22		
**Supplemental test: Non-verbal IQ** *(standardized score, WASI-II/ DAS-II)*	Intercept	57.76	3.59 – 111.92	0.04*
	Age (years)	-0.33	-1.41 – 0.74	0.52
	Sex [Male]	-7.64	-18.68 – 3.41	0.16
	**Left Crus I (cm^3)**	**1.96**	**-1.24 – 5.16**	**0.22**
	R^2 / R^2 adjusted	0.19 / 0.07		
	Robust Wald Test	2.1 on 3 and 19 DF, p-value = 0.13		
**Supplemental test: Non-verbal IQ** *(standardized score, WASI-II/ DAS-II)*	Intercept	81.43	36.61 – 126.25	1.20E-03**
	Age (years)	-0.46	-1.47 – 0.55	0.35
	Sex [Male]	-6.30	-19.92 – 7.32	0.34
	**Right Crus II / Lobule VIIB (cm^3)**	**0.28**	**-2.68 – 3.24**	**0.84**
	R^2 / R^2 adjusted	0.14 / 0.001		
	Robust Wald Test	0.4 on 3 and 19 DF, p-value = 0.73		
**Supplemental test: Non-verbal IQ** *(standardized score, WASI-II/ DAS-II)*	Intercept	69.87	19.99 – 119.74	8.55E-03**
	Age (years)	-0.41	-1.46 – 0.63	0.42
	Sex [Male]	-7.94	-21.55 – 5.68	0.24
	**Left Crus II / Lobule VIIB (cm^3)**	**1.07**	**-2.21 – 4.35**	**0.50**
	R^2 / R^2 adjusted	0.16 / 0.02		
	Robust Wald Test	0.6 on 3 and 19 DF, p-value = 0.65		
**Supplemental test: Non-verbal IQ** *(standardized score, WASI-II/ DAS-II)*	Intercept	82.86	35.86 – 129.87	1.56E-03**
	Age (years)	-0.49	-1.51 – 0.53	0.33
	Sex [Male]	-6.32	-19.31 – 6.66	0.32
	**Right Lobule VIII (cm^3)**	**0.30**	**-4.35 – 4.95**	**0.89**
	R^2 / R^2 adjusted	0.14 / 0.001		
	Robust Wald Test	0.5 on 3 and 19 DF, p-value = 0.71		

**Supplemental test: Non-verbal IQ** *(standardized score, WASI-II/ DAS-II)*	Intercept	86.77	40.92 – 132.62	8.37E-04***
	Age (years)	-0.49	-1.53 – 0.54	0.33
	Sex [Male]	-5.97	-19.04 – 7.10	0.35
	**Left Lobule VIII (cm^3)**	**-0.11**	**-4.45 – 4.23**	**0.96**
	R^2 / R^2 adjusted	0.14 / -0.0004		
	Robust Wald Test	0.5 on 3 and 19 DF, p-value = 0.72		
**Supplemental test: Non-verbal IQ** *(standardized score, WASI-II/ DAS-II)*	Intercept	82.42	12.43 – 152.41	0.02*
	Age (years)	-0.49	-1.51 – 0.53	0.33
	Sex [Male]	-6.02	-18.70 – 6.66	0.33
	**Right Lobule IX (cm^3)**	**0.92**	**-17.08 – 18.92**	**0.92**
	R^2 / R^2 adjusted	0.14 / 0.0003		
	Robust Wald Test	0.5 on 3 and 19 DF, p-value = 0.69		
**Supplemental test: Non-verbal IQ** *(standardized score, WASI-II/ DAS-II)*	Intercept	58.51	-7.64 – 124.65	0.08†
	Age (years)	-0.41	-1.38 – 0.57	0.39
	Sex [Male]	-5.83	-18.26 – 6.59	0.34
	**Left Lobule IX (cm^3)**	**7.53**	**-8.28 – 23.35**	**0.33**
	R^2 / R^2 adjusted	0.20 / 0.07		
	Robust Wald Test	1.4 on 3 and 19 DF, p-value = 0.27		
**Supplemental test: Non-verbal IQ** *(standardized score, WASI-II/ DAS-II)*	Intercept	104.67	62.19 – 147.14	5.60E-05***
	Age (years)	-0.49	-1.45 – 0.47	0.30
	Sex [Male]	-6.28	-18.46 – 5.90	0.29
	**Right Lobule X (cm^3)**	**-32.58**	**-102.34 – 37.18**	**0.34**
	R^2 / R^2 adjusted	0.19 / 0.06		
	Robust Wald Test	1.0 on 3 and 19 DF, p-value = 0.41		
**Supplemental test: Non-verbal IQ** *(standardized score, WASI-II/ DAS-II)*	Intercept	88.92	54.24 – 123.59	3.52E-05***
	Age (years)	-0.48	-1.51 – 0.54	0.34
	Sex [Male]	-5.87	-18.40 – 6.66	0.34
	**Left Lobule X (cm^3)**	**-6.12**	**-53.15 – 40.91**	**0.79**
	R^2 / R^2 adjusted	0.14 / 0.002		
	Robust Wald Test	0.4 on 3 and 19 DF, p-value = 0.73		
**Supplemental test: Non-verbal IQ** *(standardized score, WASI-II/ DAS-II)*	Intercept	87.64	25.10 – 150.18	8.54E-03**
	Age (years)	-0.50	-1.61 – 0.62	0.36
	Sex [Male]	-6.03	-18.78 – 6.72	0.34
	**Vermis I-V (cm^3)**	**-0.87**	**-24.22 – 22.48**	**0.94**
	R^2 / R^2 adjusted	0.14 / -0.0002		
	Robust Wald Test	0.5 on 3 and 19 DF, p-value = 0.69		
**Supplemental test: Non-verbal IQ** *(standardized score, WASI-II/ DAS-II)*	Intercept	155.53	124.86 – 186.20	2.00E-09***
	Age (years)	-0.34	-0.89 – 0.21	0.21
	Sex [Male]	-8.75	-17.41 – -0.09	0.05*
	**Vermis VI-VII (cm^3)**	**-48.55**	**-71.35 – -25.76**	**2.70E-04*** **
	R^2 / R^2 adjusted	0.56 / 0.49		
	Robust Wald Test	13.4 on 3 and 19 DF, p-value = 6.12E-05***		
**Supplemental test: Non-verbal IQ** *(standardized score, WASI-II/ DAS-II)*	Intercept	79.15	31.78 – 126.51	2.41E-03**
	Age (years)	-0.48	-1.51 – 0.55	0.34
	Sex [Male]	-6.31	-19.01 – 6.39	0.31
	**Vermis VIII-X (cm^3)**	**2.73**	**-15.18 – 20.63**	**0.75**
	R^2 / R^2 adjusted	0.14 / 0.004		
	Robust Wald Test	0.5 on 3 and 19 DF, p-value = 0.69		

Secondary models for subregions that were found to have a significant main effect on cognitive abilities (p ≤ 0.05), after eICV is included as an additional covariate:

Outcome variable	Explanatory variables	*b*	CI (95%)	p-value
**Composite IQ** *(standardized score, WASI-II/ DAS-II)*	Intercept	84.72	-1.54 – 170.98	0.05
	Age (years)	0.21	-0.45 – 0.87	0.51
	Sex [Male]	-7.59	-18.35 – 3.17	0.16
	eICV (cm^3)	0.03	-0.02 – 0.08	0.23
	**Vermis VI-VII (cm^3)**	**-35.78**	**-61.10 – -10.45**	**8.23E-03****
	R^2 / R^2 adjusted	0.33 / 0.18		
	Robust Wald Test	2.8 on 3 and 19 DF, p-value = 0.05*		
**Supplemental test: Non-verbal IQ** *(standardized score, WASI-II/ DAS-II)*	Intercept	122.41	51.52 – 193.31	1.92E-03**
	Age (years)	-0.26	-0.84 – 0.31	0.35
	Sex [Male]	-9.86	-18.64 – -1.07	0.03*
	eICV (cm^3)	0.02	-0.02 – 0.06	0.22
	**Vermis VI-VII (cm^3)**	**-48.53**	**-71.12 – -25.94**	**2.69E-04*****
	R^2 / R^2 adjusted	0.59 / 0.50		
	Robust Wald Test	12.9 on 3 and 19 DF, p-value = 4.03E-05***		

Secondary models for subregions that were found to have a significant main effect on cognitive abilities (p ≤ 0.05), after eICV, and standardized visual perception scores *(Beery VMI-6)* are included as additional covariates:

Outcome variable	Explanatory variables	*b*	CI (95%)	p-value
**Composite IQ** *(standardized score, WASI-II/ DAS-II)*	Intercept	78.13	-4.83 – 161.09	0.06
	Age (years)	0.22	-0.44 – 0.89	0.48
	Sex [Male]	-6.26	-18.59 – 6.08	0.30
	eICV (cm^3)	0.02	-0.03 – 0.08	0.41
	Visual Perception *(standardized score, Beery VMI-6)*	0.10	-0.13 – 0.34	0.37
	**Vermis VI-VII (cm^3)**	**-30.93**	**-57.29 – -4.57**	**0.02***
	R^2 / R^2 adjusted	0.34 / 0.15		
	Robust Wald Test	2.3 on 5 and 17 DF, p-value = 0.09†		
**Supplemental test: Non-verbal IQ** *(standardized score, WASI-II/ DAS-II)*	Intercept	104.71	50.08 – 159.34	8.43E-04***
	Age (years)	-0.22	-0.80 – 0.36	0.43
	Sex [Male]	-6.27	-15.18 – 2.63	0.16
	eICV (cm^3)	0.01	-0.04 – 0.05	0.80
	Visual Perception *(standardized score, Beery VMI-6)*	0.28	0.08 – 0.48	9.23E-03**
	**Vermis VI-VII (cm^3)**	**-35.52**	**-54.41 – -16.63**	**9.96E-04*****
	R^2 / R^2 adjusted	0.67 / 0.57		
	Robust Wald Test	9.5 on 5 and 17 DF, p-value = 1.87E-04***		

Contrast coding: reference level for sex is female. p-value ≤ 0.001‘***’, p-value ≤ 0.01‘**’, p-value ≤ 0.05‘*’, p-value ≤ 0.1‘†’. *Abbreviations*: 3q29 deletion syndrome, 3q29Del; intelligence quotient, IQ; Wechsler Abbreviated Scale of Intelligence, WASI; Differential Ability Scales, DAS; unstandardized coefficient estimate, *b*; confidence interval, CI; degrees of freedom, DF; estimated total intracranial volume, eICV.

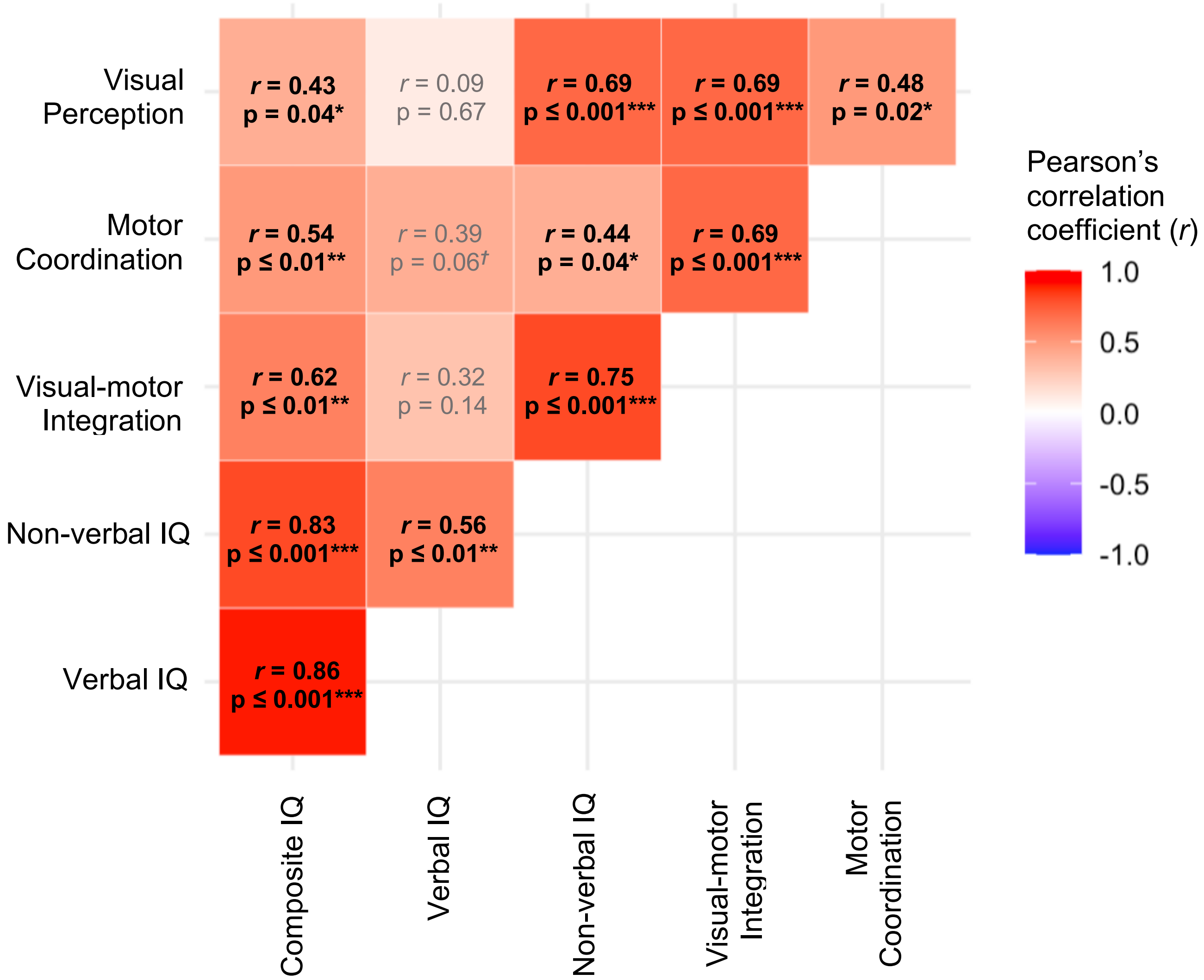

Fig. S19. Heatmap visualization of pairwise Pearson's correlations between standardized test scores for sensorimotor and cognitive abilities among 3q29Del participants. We assessed the pairwise Pearson's correlations among all behavioral measures to determine the extent to which our findings may be interrelated. In the 3q29Del sample, composite IQ, verbal IQ and non-verbal IQ scores were significantly correlated with one another (*p's* ≤ 0.01, moderate-very strong). Similarly, visual-motor integration, motor coordination, and visual perception scores showed significant pairwise correlations with each other (*p's* ≤ 0.05, moderate-strong). Composite IQ scores had significant correlations with visual-motor integration (*p* ≤ 0.01, strong), motor coordination (*p* ≤ 0.01, moderate), and visual perception scores (*p* ≤ 0.05, moderate), which were largely driven by significant correlations observed between non-verbal IQ and motor coordination scores (*p* ≤ 0.05, moderate) and between non-verbal IQ and visual perception scores (*p* ≤ 0.001, strong). Verbal IQ scores did not correlate significantly with visual-motor integration, motor coordination, or visual perception scores (*p's* > 0.05, very weak-weak). All correlation coefficients (*r*) were positive. Significant test results (*p's* ≤ 0.05) are reported in bold for clarity. The strength of the computed correlation coefficients was evaluated based on the following criteria: |*r*| = 0 – 0.19, very weak; |*r*| = 0.20 – 0.39, weak; |*r*| = 0.40 – 0.59, moderate; |*r*| = 0.60 – 0.79, strong; |*r*| = 0.80 – 1, very strong. 3q29Del *N* = 23. p-value ≤ 0.001 '***', p-value ≤ 0.01 '**', p-value ≤ 0.05 '*', p-value ≤ 0.1 '†'. *Abbreviations*: 3q29 deletion syndrome, 3q29Del; IQ, intelligence quotient.

Standardized test scores	PFAC/MCM - (*N* = 11)	PFAC/MCM + (*N* = 13)	Test statistics
**Visual-motor Integration** (*Beery VMI-6)*			
Mean ± SD	66.64 ± 16.45	68.85 ± 15.30	*t* = 0.34, DF = 22,
Median [Range]	68 [45 – 86]	70 [45 – 96]	p-value = 0.74[a]
**Supplemental test: Motor Coordination** (*Beery VMI-6)*			
Mean ± SD	64.00 ± 15.95	60.38 ± 15.79	*W* = 60.00,
Median [Range]	63 [45 – 90]	58 [45 – 83]	p-value = 0.52[b]
**Supplemental test: Visual Perception** (*Beery VMI-6)*			
Mean ± SD	69.09 ± 19.36	78.38 ± 16.41	*t* = 1.27, DF = 22,
Median [Range]	63 [45 – 98]	79 [45 – 101]	p-value = 0.22[a]
**Composite IQ** *(WASI-II/ DAS-II)*			
Mean ± SD	74.91 ± 12.83	70.77 ± 14.45	*t* = -0.74, DF = 22,
Median [Range]	75 [54 – 96]	72 [46 – 89]	p-value = 0.47[a]
**Supplemental test: Verbal IQ** *(WASI-II/ DAS-II)*			
Mean ± SD	84.73 ± 17.19	74.00 ± 20.24	*W* = 52.50,
Median [Range]	85 [57 – 106]	80 [31 – 93]	p-value = 0.28[b]
**Supplemental test: Non-verbal IQ** *(WASI-II/ DAS-II)*			
Mean ± SD	73.09 ± 12.71	75.54 ± 14.60	*t* = 0.43, DF = 22,
Median [Range]	72 [54 – 98]	79 [53 – 97]	p-value = 0.67[a]

Table S17. Standardized test scores for sensorimotor and cognitive abilities in 3q29Del participants with versus without posterior fossa arachnoid cyst or mega cisterna magna findings. There was no significant difference between PFAC/MCM positive (+) versus negative (-) 3q29Del participants in visual-motor integration, motor coordination or visual perception scores measured by the Beery-Buktenica Developmental Test of VMI, or in composite, verbal or non-verbal IQ scores measured by the WASI / DAS (*p's* > 0.05). [a]Student's two sample t-test, [b]Wilcoxon rank sum test. Non-parametric statistics are reported in cases where the data do not meet parametric assumptions. *Abbreviations*: 3q29 deletion syndrome, 3q29Del; posterior fossa arachnoid cyst, PFAC; mega cisterna magna, MCM; intelligence quotient, IQ; WASI, Wechsler Abbreviated Scale of Intelligence; DAS, Differential Ability Scales; VMI, visual-motor integration; standard deviation, SD.

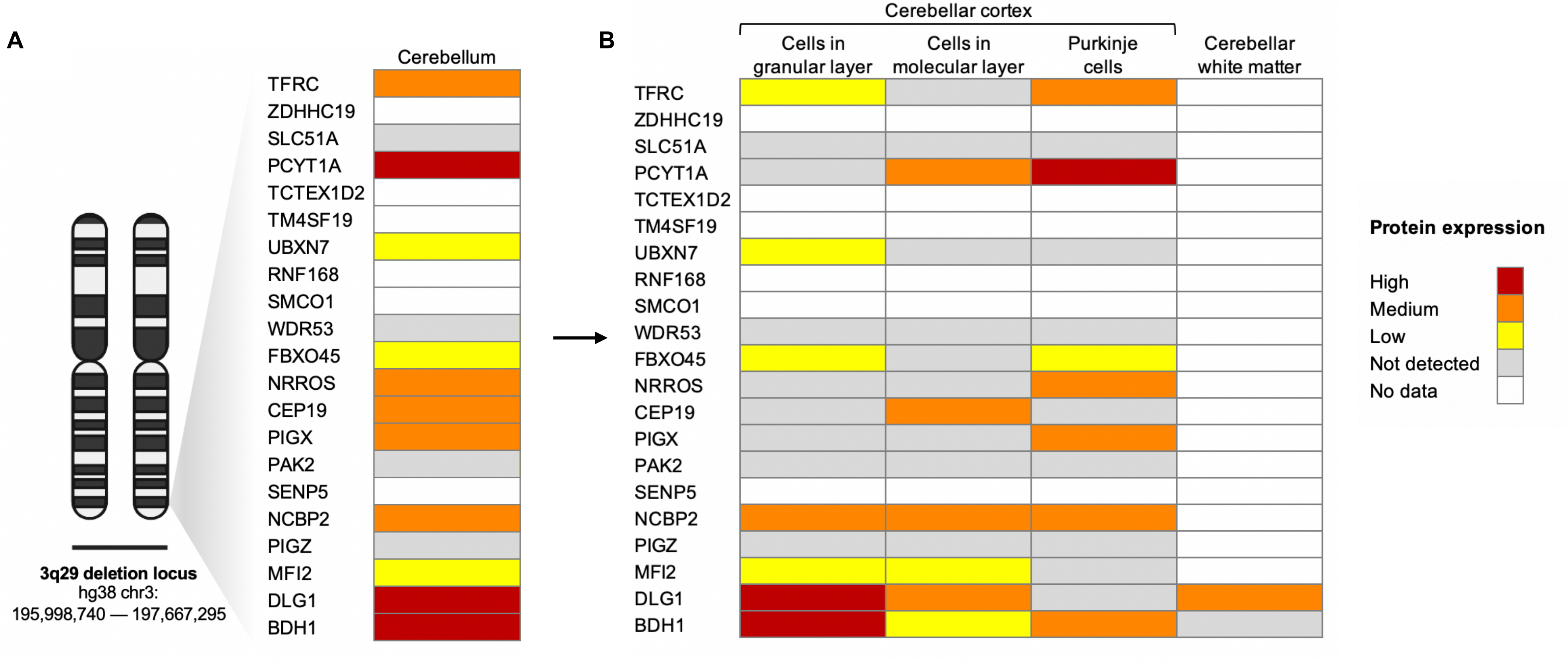

Fig. S20. Cerebellar protein expression profiles of 3q29 interval genes annotated by the Human Protein Atlas. In **A)** protein expression profiles of 21 protein coding genes located in the 3q29 interval are provided for the human cerebellum. In **B)** protein expression profiles of 3q29 interval genes are provided for annotated cerebellar subregions, using the same units. All annotations were obtained from the Human Protein Atlas (version 20.1) and were established by evaluation of immunohistochemical staining patterns, RNA-sequencing data, and available protein/gene characterization data, as described by Uhlen et al. (2010 & 2015). Protein expression profiles were characterized in normal human tissues as “High”, “Medium”, “Low” or “Not detected”. The “No data” category indicates that there was no available protein expression information on the Human Protein Atlas for a given query. The cerebellar cortex includes three layers: 1) the granular layer, which contains granule cells, golgi cells and synaptic glomeruli, 2) the molecular layer, which contains axons from the cells of the granular layer, Purkinje dendrites, astrocytes and neuronal cell bodies (e.g., stellate and basket cells), and 3) the Purkinje layer, which contains Purkinje cells (sole output of cerebellar cortex), Purkinje dendrites, interneurons, and glial cells (e.g., Bergman glia). Cerebellum is also rich in white matter, which contains myelinated axon bundles, and glial cells (e.g., oligodendrocytes). Note that, for FBXO45, RNA-based expert annotation could not be performed by the Human Protein Atlas due to inconclusive results (query date: 10.02.2021); hence the protein expression profile provided for this gene relies on immunohistochemistry findings only. The data visualized in this figure can be accessed via http://www.proteinatlas.org.

Uhlen M, Oksvold P, Fagerberg L, et al. (2010): Towards a knowledge-based human protein atlas. *Nature Biotechnology*. *28*(12):1248-1250.
Uhlen M, Fagerberg L, Hallström BM, et al. (2015): Tissue-based map of the human proteome. *Science*. 347(6220).

Supplemental methods and extended discussion

Extended methods for processing and quality control of structural MRI data

In our tissue-specific analyses dividing the cerebellum into cerebellar cortex and cerebellar white matter, the FreeSurfer software (http://surfer.nmr.mgh.harvard.edu/) was used for automated segmentation of structural MR images, based on probabilistic information estimated from a manually labeled training set (Fischl et al., 2002). This approach has been shown to yield well-defined cerebellar boundaries comparable in accuracy to manual labeling (Fischl et al., 2002; Lee et al., 2015). Prior findings also indicate that the FreeSurfer algorithm is especially suitable for multi-center data acquired on different scanners, as it demonstrates relatively low sensitivity to noise and variable image quality (Mayer et al., 2016; Dewey et al., 2010). Note that a mock scanner training protocol was used in the 3q29Del project to minimize motion artifacts and attrition; participants with a contraindication for MRI were excluded.

Since it is currently unfeasible to accurately reconstruct and segment the cortical surface of the cerebellum using this harmonized framework, we treated the cerebellum as a volumetric structure. For quality control (QC), two trained evaluators (ES, LL) inspected all cerebellar segmentations obtained from 3q29Del participants in axial, sagittal, and coronal reformats to determine whether technical problems (e.g., motion artifacts) or notable pathology (e.g., arachnoid cysts) interfered with registration or segmentation quality. One 3q29Del participant failed to pass QC due to a motion artifact and skull deformity interfering with the extraction of reliable volumetric measures (Fig. S2O). Similarly, the outputs of the structural pipeline for control participants were inspected by the HCP, as described by Marcus et al. (2013) and Elam et al. (2021); no major errors were identified. Detailed QC information for the HCP dataset is available at https://wiki.humanconnectome.org/. To facilitate joint analysis, the two trained evaluators additionally cross-compared the image quality, tissue contrast and cerebellar segmentation masks of 23 age- and sex-matched case-control pairs randomly selected from the entire dataset (see Fig. S2A-N for representative images). No systematic irregularities were identified between the two diagnostic groups and no manual intervention was performed in either group to avoid adding subjectivity to volumetric measures.

Since visual QC of eICV segmentation masks is not attainable in the atlas-based head size normalization approach used in the present study, we assessed the quality of our eICV measures by testing the correlations between eICV, total brain volume and head circumference among 3q29Del participants using Pearson product moment analysis. As expected from previous literature (McKinney et al., 2017; Koyabu et al., 2014; Buckner et al., 2004; Sanfilipo et al., 2004; Kollias et al., 1993), these correlations were positive and significant with moderate to strong effect sizes (p's ≤ 0.05) (Fig. S4), providing an indirect means of quality assurance for the eICV data.

In our lobule-specific analyses dividing the cerebellar cortex into 17 subregions, the CPU version of the open-source "Automatic cerebellum anatomical parcellation using U-Net with locally constrained optimization" (ACAPULCO) software was used for automated segmentation of T1-weighted structural MR images, based on convolutional neural networks (CNNs). Briefly, the processing steps included N4 bias field correction and MNI registration. A cascade of two 3D CNNs were used, with the first network functioning as the "locating network" that predicts a bounding box around the cerebellum to reduce the spatial size of the input that is cropped and fed into the second network. The second network functions as the "parcellating network" that divides the cerebellum into smaller subregions within the bounding box, using a modified U-Net. This approach yielded the following 17 parcellations for the cerebellar cortex consistent with commonly used transverse lobule-based anatomical boundaries: hemispheric lobules I-V (left and right), lobule VI (left and right), Crus I (left and right), Crus II / lobule VIIB (left and right), lobule VIII (left and right), lobule IX (left and right), lobule X (left and right), and vermal lobules I-V, VI-VII, VIII-X. ACAPULCO also generates a "corpus medullare" label, which was not used in the present study, since the corresponding algorithm was designed primarily for cerebellar grey matter parcellations and not optimized for white matter parcellations (Kerestes et al., 2022). The post-processed parcellations, which were performed in MNI space, were transformed back into original image space by using nearest-neighbor interpolation. Detailed methods on pre- and post-processing, training, validation, and the

precise architectures of the CNNs have been described in length by Han et al. (2020). Since our study sample primarily consisted of younger individuals (fewer adults than children and adolescents), we employed the ACAPULCO model trained on a pediatric cohort (Han et al., 2020).

We note that the low computational replicability (i.e., repetitive runs of the same MRI data not yielding the same parcellation results) previously reported by Sörös et al. (2021) has been fixed in the 3.0 version of the ACAPULCO pipeline that is used in the present study. The corresponding documentation can be found in the following GitLab page: https://gitlab.com/shuohan/acapulco/.

A multi-step process was completed for QC of the ACAPULCO-parcellated images. First, the 'QC_Images.html' file that is automatically generated by ACAPULCO was examined by two independent evaluators (ES, KD) by scrolling through the images for each subject to identify obvious parcellation failures or systematic issues. The same 3q29Del participant who failed the QC process for FreeSurfer-parcellated images similarly failed QC for ACAPULCO-parcellated images due to the quality issues described above; hence this participant was excluded from all subregional volumetric analyses. Next, using the ITK-SNAP (v.4.0) NIFTI image viewer (http://www.itksnap.org/), ACAPULCO masks were overlaid onto the original T1-weighted image of each participant to check the quality of the parcellations slice by slice in 2D axial, coronal, and sagittal planes, as well as by generating rotating reconstructions of the parcellations in 3D. Flagged parcellation errors were manually corrected by a trained neuroscientist (ES) slice by slice, in consultation with a licensed neuroradiologist (AEGY), while remaining blinded to case/control status and taking into account normal variability in each individual's cerebellar anatomy. Following manual-correction, resulting parcellations were subjected to another round of QC and anatomical boundaries were further corrected where necessary. All 17 subregions of the cerebellar cortex were retained for each participant included in this arm of the study. Volumes of each subregion were calculated in mm^3 first and converted to cm^3 for consistency with the units of measurement reported in our FreeSurfer-based analyses. See Fig. S3 for representative ACAPULCO-parcellated images post-QC along with their 3D visualizations for a subset of age- and sex-matched participants with 3q29Del and neurotypical controls.

In addition to lobule-based subregional analyses with ACAPULCO, supplemental voxel-based morphometry (VBM) analyses were performed for increased resolution and flexibility, using the Spatially Unbiased Infratentorial Template (SUIT) toolbox, which is specifically designed for VBM analysis of human cerebellum data (and the brainstem) by employing a high-resolution atlas template (Diedrichsen, 2006; Diedrichsen et al., 2009). By employing a high-resolution cerebellum template, SUIT preserves the true anatomical detail and subregional complexity of the cerebellum to a much greater extent than the commonly used whole-brain ICBM152 template, which is the standard approach for defining the MNI space in whole-brain VBM analyses. As shown by Diedrichsen (2006), the whole-brain ICBM152 template offers very little contrast for cerebellar structures and leads to poor spatial alignment of cerebellar fissures, limiting the usefulness of a voxel-based approach for investigating volumetric alterations in this region. As noted by the developers, performing VBM with SUIT has several advantages: first, the overlap of cerebellar structures across participants is improved, and second, by masking the image before re-slicing it into the atlas space, no supratentorial gray matter can bias the results. Due to its documented advantages, SUIT was employed to gain voxel-level insights into cerebellar gray matter volume in the present sample. Note that SUIT performs a separate parcellation of lobules I-IV and lobule V, whereas ACAPULCO combines these lobules under a single parcellation label. Additionally, SUIT performs separate parcellation of lobules VIIIa and VIIIb, while ACAPULCO combines these labels to generate a single parcellation for lobule VIII.

Extended methods for radiological evaluation of structural MRI data

T1- and T2-weighted images were reviewed qualitatively by a board-certified neuroradiologist (AEGY) in axial, sagittal, and coronal reformats using Horos (https://horosproject.org). The conditions that were evaluated for differential diagnosis of enlarged retrocerebellar cerebrospinal fluid (CSF) space includes Dandy-Walker malformation (DWM), Blake's pouch cyst, mega cisterna magna (MCM), posterior fossa arachnoid cyst (PFAC), and isolated inferior cerebellar vermian hypoplasia. DWM, the most common posterior fossa malformation in the general population, can occur in isolation or as part of chromosomal anomalies or Mendelian disorders (Doherty

et al., 2013). Diagnostic features of DWM on neuroimaging include hypoplasia of the cerebellar vermis, which is elevated and upwardly rotated, and dilation of the fourth ventricle, which fills and enlarges the posterior fossa (Bosemani et al., 2015). DWM is often associated with additional malformations, including callosal dysgenesis, occipital encephaloceles, polymicrogyria, and grey matter heterotropia (Parisi et al., 2003). Hydrocephalus may be present. Blake's pouch cyst occurs sporadically due to a lack of fenestration of the Blake pouch, a normal developmental structure, resulting in absence of communication between the fourth ventricle and the subarachnoid space, and leading to hydrocephalus (Tortori-Donati et al., 1996; Cornips et al., 2010). The cerebellum has a normal size and shape. Imaging demonstrates an enlarged fourth ventricle that communicates with an infravermian cyst, often resulting in hydrocephalus. MCM is an enlarged cisterna magna (≥10 mm on midsagittal images) with an intact vermis and a normal fourth ventricle. The posterior fossa may be enlarged, with scalloping of the occipital bone, without hydrocephalus. MCM may be caused by delayed fenestration of the Blake's pouch, whereas the absence of fenestration leads to a Blake's pouch cyst (Nelson et al., 2004). Arachnoid cysts result from duplication of the arachnoid membrane, with approximately 10% of arachnoid cysts in children occurring in the posterior fossa (Ali et al., 2014). Although PFACs may be asymptomatic and identified incidentally, they may present with macrocephaly, increased intracranial pressure, and developmental delay, particularly if CSF flow is obstructed (Marin-Sanabria et al., 2007). PFACs are isointense relative to CSF, well defined, can also result in scalloping of the occipital bone, and can exert mass effect on the cerebellum, with a normal appearance of the fourth ventricle and vermis. Isolated inferior vermian hypoplasia is characterized by partial absence of the inferior portion of the cerebellar vermis. More than 75% of patients with isolated inferior vermian hypoplasia have a favorable outcome, although in some patients, mild functional deficits in fine motor activity and receptive language may be present (Limperopoulos et al., 2006; Tarui et al., 2014).

Details on standardized behavioral measures

Participants were administered the *Beery-Buktenica Developmental Test of Visual-Motor Integration* (VMI, 6th edition) to assess the extent to which they can integrate their visual and fine motor abilities in a geometric design-copying task. To measure VMI, participants were asked to copy a set of geometric forms that increase in difficulty; the drawings were scored based on standard criteria outlined in the test manual according to how accurately each design was copied compared with the original.

The two supplemental tests included in the Beery VMI were additionally administered to assess visual and motor skills, separately. The visual perception supplemental test was used to assess visual analysis skills in a format requiring minimal motor input. In this task, participants were shown a series of reference geometric designs with multiple similar shapes provided below them; the task required participants to choose/match the shape that was identical to the reference design. The motor coordination supplemental test was used to assess fine motor coordination skills in a format requiring minimal visual analysis skills. In this task, participants were asked to trace a set of geometric forms with a pencil without going outside a double-lined path. Raw scores were converted to age-appropriate standard scores based on the standardization sample reported for the corresponding tests (normative mean = 100, SD = 15). Higher scores indicate better performance.

Two measures of intellectual functioning were utilized in the present study given the age range of the participants. The general conceptual ability (GCA) and full-scale intelligence quotient (FSIQ) scores from the *Differential Ability Scales* (DAS, 2nd edition) and the *Wechsler Abbreviated Scale of Intelligence* (WASI, 2nd edition) were used as composite IQ scores. The verbal reasoning and verbal comprehension domains of the two scales were used to index verbal IQ, while the non-verbal reasoning and perceptual reasoning domains indexed non-verbal IQ. The contents of these tests are sufficiently similar for combination across scales in cross-sectional analyses. Raw scores were transformed into age-appropriate standard scores based on the standardization sample for the corresponding tests (normative mean = 100, SD = 15). Higher scores indicate better performance.

Extended statistical methods for penalized cubic spline and quantile spline models

In tissue-specific analyses, the following procedure was followed to 1) build developmental trajectories for our volumetric measures of interest (VOI) and 2) to estimate normative percentile curves for our VOIs (akin to a

growth chart) as an alternative to the polynomial linear regression models presented in the main manuscript for case-control comparisons. In these supplemental analyses, penalized cubic splines were fitted to volumetric data using the *mgcv* package in *R* (version 1.8-33) (Wood, 2011; Wood, 2017) and quantile splines were fitted to volumetric data using the *fields* package in *R* (version 12.5) (Nychka et al., 2021). Given the local nature of splines, these methods are capable of capturing a wide range of nonlinear neurodevelopmental trends in our data and were incorporated into the present study to improve the statistical rigor of our central analyses. We provide a detailed summary of each approach below.

1) *Building developmental trajectories for VOIs:*

As a supplemental method, the penalized spline approach (Wahba, 1980; Eilers et al., 1996) was adopted to model volumetric changes in our brain regions of interest across age, since this method offers increased mathematical flexibility compared with parametric models that rely on delicate assumptions. As in regression splines (Eilers et al., 1996), penalized splines make use of piecewise polynomial approximation with a flexible selection of knots to effectively capture the underlying changes in a dataset, when given a fixed basis-dimension and appropriate positioning of the knots that provides fair coverage of the covariate values (Wood, 2017). This creates computational efficiency compared with smoothing splines, which place a knot at every unique sample point. Moreover, penalized splines add a roughness (or “wiggliness”) penalty to the model, as in smoothing splines (Reinsch, 1967), to control the smoothness of the fit while avoiding the problem of overfitting. In summary, penalized splines offer an effective compromise between regression splines and smoothing splines (two popular tools) for more closely approximating age-associated changes in our outcome variables of interest.

Below, we will gradually build our way towards defining the penalized cubic spline approach that was used to characterize the developmental trajectories of our VOIs.

The definition of a natural spline is as follows (given a set of knots at $k_1, k_2, \dots, k_K$):

1. $f(x)$ is a polynomial of degree p on each of the intervals $[k_1, k_2]$, $[k_2, k_3]$, ..., $[k_{K-1}, k_K]$.
2. $f(x)$ has continuous $(p-1)_{th}$ derivatives at knots $k_1, k_2, \dots, k_K$.
3. $f(x)$is a polynomial of degree $\frac{(p-1)}{2}$ on $(-\infty, k_1]$ and $[k_K, \infty)$.

The last requirement forces a lower degree to the left (or right) of the leftmost (or rightmost) knot, to reduce the variance at the boundaries of the observations. In the present study, we used natural cubic splines ($p = 3$), which are linear beyond the boundary knots.

The unique solution to the optimization problem described in *equation (1)* is a natural cubic spline with knots at every unique observation $x_1, x_2, \dots, x_n$. The solution $f(x)$ is called a smoothing spline (Reinsch, 1967).

$$\min_f \{ \frac{1}{n} \sum_{i=1}^{n} (y_i - f(x_i))^2 + \lambda \int (f''(x))^2 dx \} \qquad \text{Equation (1)}$$

where $(x_i, y_i), i = 1, \dots, n$ are a set of observations, $f(x)$ includes all that have continuous second derivatives and $\lambda > 0$ is a smoothing parameter that controls the trade-off between fidelity to the data and roughness of the function estimate.

Although smoothing splines enjoy the theoretical property of an optimizing solution (as described above), the degrees of freedom (i.e., number of knots) are comparable to the data to be smoothed and can be redundant,

as the knots are placed at every unique observation. If the penalty term in *equation (1)* is removed and $f(x)$ is restricted to a natural spline with user-defined knots (as in the definition of a natural spline where the knot positions at $k_1, k_2, \ldots, k_K$ can be completely decided by users), the solution is reduced to a regression spline. Although regression splines ease the computational redundancy of smoothing splines by removing any restrictions in knot selection, they have the potential to be over-fitted. This can be handled by a regularization term, such as the integrated square second derivative in smoothing splines. Therefore, it is natural to combine the freely chosen degrees of freedom of a regression spline and the roughness penalty of a smoothing spline, which in turn gives penalized splines (Wahba, 1980). Specifically, we use penalized cubic splines in the present study, which is the solution to the optimization problem in *equation (2)*, where the number and positions of the knots are freely chosen in advance and the number of knots is usually much smaller than the sample size:

$$\begin{cases} \min_f \{\frac{1}{n}\sum_{i=1}^{n}(y_i - f(x_i))^2 + \lambda \int (f''(x))^2 dx\} \\ f \text{ is a natural cubic spline with predefined knots at } k_1, \ldots, k_K \end{cases}$$ *Equation (2)*

To obtain a satisfying fit, the knots should be arranged nicely to cover the distribution of the covariate in the original data set (Wood, 2017).

For each VOI (i.e., total cerebellum volume, cerebellar cortex volume, cerebellar white matter volume, estimated total intracranial volume (eICV), and the eICV-adjusted versions of cerebellar volumes), we denote y_i as the corresponding volume of the i_{th} participant in the pooled dataset (i.e., data aggregated across the two diagnostic groups). It is assumed that y_i is represented by a function of the participant's age, with sex and diagnostic group added as covariates, as expressed in *equation (3).*

$$y_i = f(Age_i) + \alpha \cdot Sex_i + \beta \cdot Group_i + \epsilon_i,\ \epsilon_i \overset{\text{iid}}{\sim} N(0, \sigma^2)$$ *Equation (3)*

Sex_i is a sex indicator taking 1 for males and 0 for females. $Group_i$ is a diagnostic group indicator taking 1 for 3q29Del participants and 0 for neurotypical control participants. The residual random error ϵ_i is assumed to follow a Gaussian distribution with mean 0 and a common variance σ^2 across participants (homoscedasticity). $f(Age_i)$ is a natural cubic spline term of age. The number of knots is chosen to be 10 and they evenly cover the quantiles of the age distribution (i.e., the knots are placed at the 0%, 11.11%, 22.22%, 33.33%, 44.44%, 55.56%, 66.67%, 77.78%, 88.89%, and 100% quantiles of age in the pooled data).

Following the optimization framework of a penalized cubic spline described in *equation (2)*, the parameter estimates are obtained via the objective function:

$$\begin{cases} \min_f \{\frac{1}{n}\sum_{i=1}^{n}(y_i - f(Age_i) - \alpha \cdot Sex_i - \beta \cdot Group_i)^2 + \lambda \int (f''(x))^2 dx\} \\ f \text{ is a natural cubic spline with with 10 knots spread evenly across the age quantiles} \end{cases}$$ *Equation (4)*

where the smoothing parameter λ is selected by the restricted maximum likelihood (REML) method (Wood, 2011).

From the fitted penalized cubic splines, mean developmental trajectories for each VOI were estimated and laid onto the scatter plots of the original volumetric data points in Fig. S9. The effective degrees of freedom (EDF) reported in Table S5 represent how complex the neurodevelopmental pattern of the estimated volumetric trajectory of each VOI is across age. EDF = 1 is equivalent to a straight line, EDF = 2 is equivalent to a quadratic

curve, etc., with higher EDFs describing increased wiggliness (Wood, 2017). The EDF of the smooth term is defined as the trace of the influence matrix. The significance of the smooth term $f(Age)$, the sex indicator and the diagnostic group indicator are also reported in Table S5 in the form of p-values. Note that the significance of the smooth term is derived from an approximate F-test of whether the smooth term of age is significant in the penalized cubic spline models for each VOI ($f(Age) = 0$). The p-values are approximate in the sense that the components of the test statistic are weighted by the iterative fitting weights (Wood, 2017). A comprehensive description of how to obtain theoretical p-values is detailed by Wood (2013). The p-values for sex and diagnostic group are derived from standard t-tests of whether the indicator of sex/diagnostic group is significant in determining each VOI.

All of the aforementioned parameter estimates and p-values were obtained using the *gam()* function from the *mgcv* package in R (Wood, 2011; Wood, 2017). Using the function *gam.check()* from the same package, model diagnostics were performed, which confirmed that the required assumptions of the penalized cubic spline model in *equation (3)* were met and our basis dimension (10 knots of 9 basis dimension) was adequate to cover the age distribution of the present study sample. More specifically, QQ-plots were explored to justify the Gaussian assumption of the residuals; residuals versus linear predictors/fitted values were plotted to justify the homoscedasticity assumption; the histogram of residuals was plotted to check the normality of the residuals; and the p-value for the residual randomization test was explored to check the adequacy of the basis dimension.

2) *<u>Estimating normative percentile curves for VOIs:</u>*

Besides estimating mean volumetric changes across age in the entire study sample (i.e., data pooled across the two diagnostic groups), another interest was in estimating sex-specific normative percentiles for volumetric changes observed across age in our neurotypical control participants only. Here, our goal was to establish well-founded references (or “nomograms”) for comparison against the VOIs of individual 3q29Del participants to further characterize the degrees of neuroanatomical deviance observed within the 3q29Del sample. A secondary goal was to explore the relative distribution of 3q29Del data points in these normative charts to gain insights into potential age-windows of heightened structural vulnerability. However, the limited sample size of the 3q29Del group (especially when stratified by sex), and the paucity of data on control participants younger than age 5 precluded our ability to reach any meaningful inferences in this regard. We foresee that these estimates may become particularly useful for future studies investigating longitudinal neuroimaging outcomes in 3q29Del participants.

In the supplemental analyses described below, we characterized the 10th, 25th, 50th, 75th, and 90th percentiles of normative volumetric growth curves for each VOI, stratified by sex. Similar to modelling mean volume changes, our goal here was to model normative percentiles under a penalized spline framework, hence we used the quantile smoothing splines approach for this objective.

In quantile regression, the check loss shown in *equation (5)* is used as the objective of model fitting, where α represents the α quantile of the dependent variable y, x is the independent variable influencing y, and $1_{\{u<0\}}$ is an indicator taking 1 when $u < 0$ and 0 otherwise.

$$\rho_\alpha\big(y - g(x)\big) = (y - g(x))(\alpha - 1_{\{(y-g(x))<0\}}) \qquad \text{Equation (5)}$$

To improve computation, Nychka et al. (1995) proposed a modified check loss which rounds out the corner of the check loss in a small interval around zero by piecing in a quadratic function, in order to make the loss function differentiable at zero. The modified check loss is shown in the following equation:

$$\rho_\alpha, C\big(y - g(x)\big) = \begin{cases} \rho_\alpha\big(y - g(x)\big), & if\ |y - g(x)| > C \\ \dfrac{1-\alpha}{C}(y - g(x))^2, & if\ 0 \le y - g(x) \le C \\ \dfrac{\alpha}{C}\big(y - g(x)\big)^2, & if\ -C \le y - g(x) < 0 \end{cases} \qquad \text{Equation (6)}$$

where C is a scale factor for rounding out the absolute value function at zero to a quadratic (Nychka et al., 2021). According to Nychka et al. (1995), C should be chosen to be effectively zero relative to the magnitude of the data values.

After presenting the form of the modified check loss, we can adopt the optimization framework of smoothing splines shown in *equation (1)* by simply replacing the squared error loss to the modified check loss in *equation (6)*:

$$\min_{g}\{\frac{1}{n}\sum_{i=1}^{n}\rho_{\alpha},C(y_i - g(x_i)) + \lambda\int(g''(x))^2dx\} \quad \text{Equation (7)}$$

Similar to least square smoothing splines, the quantile smoothing splines that we adopt use the L2 roughness penalty on the integral of the squared second derivative.

For each VOI (i.e., total cerebellum volume, cerebellar cortex volume, cerebellar white matter volume, estimated total intracranial volume (eICV), we denote y_i as the corresponding volume of the i_{th} neurotypical control participant, stratified by sex. It is assumed that the α quantile of y_i is represented by a function of the participant's age $g(Age_i)$ (i.e., $P(y_i \leq g(Age_i) = \alpha)$. Following the optimization framework of a quantile spline described in *equation (7)*, the parameter estimates are obtained via the following objective over all g, such that the roughness penalty is finite:

$$\min_{g}\{\frac{1}{n}\sum_{i=1}^{n}\rho_{\alpha},C(y_i - g(Age_i)) + \lambda\int(g''(x))^2dx\} \quad \text{Equation (8)}$$

where the smoothing parameter λ is selected by the generalized cross-validation (GCV) method (Graven, 1989), and C is set to 10^{-5} of the variance of the y's.

From the fitted quantile splines for α = 0.10, 0.25, 0.50, 0.75, 0.90, normative developmental trajectories for the 10th, 25th, 50th, 75th, and 90th percentiles of the VOIs were estimated, respectively. The computation was performed using the *qsreg()* function from the *fields* package in R (Douglas Nychka et al., 2021). In Fig. S12, the original VOIs of individual 3q29Del participants are plotted against the normative percentile curves derived from male and female controls, separately. This case-by-case comparison provides a general idea of which age- and sex-specific normative percentile each 3q29Del participant's cerebellar and eICV volumes correspond to.

Spline-based age trajectories were not constructed in lobule-specific analyses dividing the cerebellar cortex into 17 subregions, due to the smaller sample size of neurotypical control participants included in this arm of the study, which resulted in reduced statistical power for detailed interrogation of the influence of age and sex on corresponding case-control differences.

List of R packages used for statistical analyses and diagnostics

Multiple linear regression analyses were performed via the standard R *lm()* function. For model comparisons, analyses of variance (ANOVA) were performed by the standard R *anova()* function. Wald statistics were calculated by the *waldtest()* function from the *lmtest* package (https://CRAN.R-project.org/package=lmtest). Heteroscedasticity-robust estimates were calculated using the *vcovHC()* function from the *sandwich* package (https://CRAN.R-project.org/package=sandwich). Permutation tests were performed using the *lmperm()* function of the *permuco* package https://CRAN.R-project.org/packages=permuco). Diagnostics plots for linear regression were created using the R base function *plot()*. Shapiro-Wilk tests were performed using the standard R *shapiro.test()* function to check for assumptions of normality. Breusch-Pagan and Levene's test were performed

using the *bptest()* function from the *lmtest* package (https://CRAN.R-project.org/package=lmtest) and the *levene_test()* function from the *rstatix* package (https://CRAN.R-project.org/package=rstatix) to check for homogeneity of variances, respectively. Fisher's exact tests, Pearson's chi-squared tests, Student's two sample t-tests and Wilcoxon signed-rank tests were performed using the standard R *fisher.test(), chisq.test(), t.test()* and *wilcox.test()* functions. Effect sizes for Wilcoxon signed-rank tests were calculated using the *wilcox_effsize()* function from the *rstatix* package (https://CRAN.R-project.org/package=rstatix). For Student's two sample t-tests, effect sizes and corresponding 95% confidence intervals by bootstrap were calculated using the *cohens_d()* function from the *rstatix* package (https://CRAN.R-project.org/package=rstatix). The standard R *cor.test()* function was used to perform Pearson's correlations. The *pcor.test()* function from the *ppcor* package (https://CRAN.R-project.org/package=ppcor) was used to calculate partial correlations. For spline modeling, the *gam()* and *gam.check()* functions from the *mgcv* package (https://cran.r-project.org/package=mgcv) and the *qsreg()* function from the *fields* package (https://cran.r-project.org/package=fields) were used. Graphics were generated by the *ggplot2* (https://CRAN.R-project.org/package=ggplot2), *ggpubr (https://cran.r-project.org/package=ggpubr)* and *jtools* (https://cran.r-project.org/package=jtools) packages.

Extended discussion of cerebellar growth trajectories

While the estimation of normative cerebellar growth trajectories across the lifespan was not the primary objective of the current study, particular attention was paid to performing detailed examinations of the linear and non-linear developmental trajectories that total cerebellum, cerebellar cortex and cerebellar white matter volumes follow. These analyses aimed to determine the appropriate functional form of age that should be used as a covariate in subsequent analyses. Especially given the wide age range of our control and 3q29Del participants (4–39 years), which encompasses dynamic stages of brain development and maturation between early childhood and middle adulthood, appropriate consideration of age effects was crucial to increase statistical rigor. The study's central findings were reinforced through several methods that further considered the influence of age, including additional sensitivity analyses that excluded a 3q29Del subject who fell outside the age range of the control group, the use of spline modeling within the Generalized Additive Model (GAM) framework, and age- and sex-matched case-control comparisons. Exploratory models testing age by diagnostic group and sex by diagnostic group interaction effects additionally allowed for preliminary investigations of the moderating influence of age and sex on identified group differences, although with reduced statistical power.

As briefly noted in the main manuscript, the cerebellum undergoes a protracted postnatal development characterized by distinct growth trajectories for its cortex and white matter, with volumetric peaks occurring at different timescales (Ostby et al., 2009; Tiemeier et al., 2010; Wu et al., 2011; Wierenga et al., 2014; Sussman et al., 2016; Wierenga et al., 2018; Romero et al., 2021). In our neurotypical control sample, cross-sectional modeling revealed developmental patterns consistent with these earlier findings.

Notably, in one of the largest reports of cerebellar growth trajectories in neurotypical participants aged 1–94 years, Romero et al. (2021) found that absolute cerebellar white matter volume exhibits a period of fast growth during youth that reaches its maximum around age 10 years. Afterward, it follows an inverted U-shaped trajectory, showing continued growth at a slower rate until approximately ages 30–40 years. In the same sample examined by Romero et al. (2021), the absolute volume of the cerebellar cortex displayed a similar pattern of fast growth during the first 10 years of postnatal life. However, in contrast to cerebellar white matter, the cerebellar cortex exhibited a constant slow decrease in volume during subsequent ages. In additional models where normalized volumes (i.e., absolute volume divided by total intracranial volume) were considered to account for individual differences in head size, cerebellar gray matter no longer exhibited an inverted U-shape, but instead showed a decreasing trend across the interrogated age range, with a steeper slope observed in early years (until approximately age 25 years), followed by a period of relative plateau, and another decline after age 60 years. In contrast, after normalization, cerebellar white matter continued to display an inverted U-shaped trajectory, but with its peak volume shifted to approximately age 50 years. In another study of neurotypical children, adolescents and young adults aged 8–30 years, Ostby et al. (2009) also found distinct developmental characteristics for gray matter and white matter within the cerebellum. Their results confirmed a general tendency of reduction for cerebellar gray matter with increasing age, although linearly for both absolute and normalized

volumes (i.e., proportion of total brain volume). In contrast, a quadratic relationship was estimated with age for both absolute and normalized cerebellar white matter volumes.

Our tissue-specific trajectory estimates for absolute and normalized cerebellar cortex and white matter volumes derived by FreeSurfer are in general agreement with these previously reported patterns, particularly for the age range overlapping 4–39 years. In our multiple linear regression analyses, testing different polynomial terms for age for model selection, the best-fitting models for total cerebellum (Table S3A), cerebellar cortex (Table S3B), and cerebellar white matter (Table S3C) volumes all included a quadratic term for age, with a later peak observed in the inverted-U shaped trajectory of cerebellar white matter compared to cerebellar cortex. Similarly, the best-fitting model for cerebellar cortex to white matter volume ratios (Table S3D) also included a quadratic term for age, but with a downward trend that reached a plateau in older ages. When these absolute volumes were adjusted for estimated total intracranial volume (eICV), the best-fitting models for total cerebellum (Table S3F) and cerebellar cortex (Table S3G) had a linear term for age. In both cases, this linear term had a negative slope, indicating a decline with increasing age; however, the cerebellar cortex showed a steeper rate of volumetric decline compared to the total cerebellum. After eICV-adjustment, cerebellar white matter still retained a quadratic form of age in its best-fitting model (Table S3H), although there was a subtle change in the curvature of its ascending slope.

Developmental trajectories for tissue-specific cerebellar volumes estimated with our penalized cubic spline approach yielded similar trends (Fig. S9, Table S5), but splines were better able to capture the non-linear effects of age in eICV-adjusted analyses, particularly for cerebellar cortex volumes. The effective degrees of freedom (EDF) were greater than 1 for the spline term of age ("s(Age)") included in all of our generalized additive models, confirming that the growth trajectories of both cerebellar cortex and cerebellar white matter are non-linear (EDF = 1 is equivalent to a straight line). However, the specific EDF values and the corresponding shape of the spline-fits differed between these two tissue-types. See Table S3 for more detailed findings on age-related volumetric changes in polynomial multiple linear regression models testing diagnostic group effects, along with accompanying trajectory visualizations in our study sample. Refer to Table S5, Figure S9, and Figure S12 for insights derived from splines, which were included as a supplementary method to better account for non-linear age patterns in volume.

In light of these findings, we highlight that studies of the cerebellum in both typically developing and clinical populations that do not differentiate between white and gray matter are at risk of overlooking differences in tissue-specific developmental trajectories. Furthermore, they may not capture the developmental moderation of pathological changes in these specific tissue types. Therefore, distinguishing between white and gray matter in cerebellar studies is crucial for a more comprehensive understanding of developmental patterns and potential pathological alterations affecting this brain region.

Note that, detailed developmental trajectories were not constructed in lobule-specific analyses dividing the cerebellar cortex into 17 subregions, due to the smaller sample size of neurotypical control participants included in this arm of the study, which resulted in reduced statistical power.

Extended discussion of subregional findings with ACAPULCO

The ACAPULCO-based subregional analyses presented in the current study, make use of the cerebellar nomenclature (lobules numbered with the Roman numerals I through X) proposed by Schmahmann et al., (1990). Prior literature indicates that distinct "syndromes" are associated with damage to distinct cerebellar subregions. In localized cerebellar lesion studies, Schmahmann and colleagues have attributed impairments in linguistic processing, visuospatial, and cognitive functions to disruptions in primarily the posterior lobe of the cerebellum, including lobules VI and VII (e.g., Schmahmann & Sherman, 1998; Schmahmann et al., 2007; Stoodley et al., 2016). These findings bear similarities to our subregional behavioral findings in study participants with 3q29Del; however larger is not better in our volumetric association findings between vermal lobules VI-VII, and standardized test scores for composite IQ, non-verbal IQ and visual perception skills. We also find significant case-control differences in bilateral hemispheric lobules I-V (anterior lobe), which are often characterized as the

“sensorimotor cerebellum”, and in bilateral hemispheric lobule VI (superior-posterior lobe), which is often characterized as part of the “cognitive cerebellum”. The location of lesion determines motor vs. cognitive consequences in patients with cerebellar damage; however, it is important to highlight that the timing of (i.e., the age at which) damage occurs is a pivotal determinant of outcome characteristics. Specifically, cognitive and social functions seem to be particularly vulnerable to impairment when cerebellar damage occurs during early life (often described as "sensitive periods", reviewed in Wang et al., 2014).

Interestingly, the cognitive and social sequela of cerebellar injury might exhibit an opposite age-dependence from its motor consequences. For example, in the case of very early preterm children with significantly reduced cerebellar volumes compared with term-born controls, no correlation has been observed between the volume of the underdeveloped cerebellum and motor functions later in life (Allin et al., 2001). However, notable associations have been found with cognitive test scores at follow-up (Allin et al., 2001). This finding, along with related findings in Tavano et al., 2007, was interpreted as indicative of the potential for functional compensation for motor but not cognitive consequences of early life damage to the cerebellum. This theorized compensation may be related to the developmental plasticity of motor-related cortico-cerebellar circuits or the existence of compensatory mechanisms for motor function elsewhere in the brain. In contrast, when motor regions of the cerebellum are damaged in adults, ensuing symptoms include ataxia of stance and gait (Timmann et al., 2008).

Gross or severe motor phenotypes were not observed in our study participants with 3q29Del, as determined previously through clinical neurological examinations conducted by a pediatric neurologist (Sanchez Russo et al., 2021). Despite significant case-control differences observed in the anterior lobules of the cerebellum, which are traditionally associated with motor functions, the absence of gross motor deficits like ataxia in our 3q29Del sample may be due to a similar compensatory mechanism at play.

Lastly, we note that the scope of our behavioral measures, which, for example, excludes outcomes for affective and social dysregulation, imposes limitations on our ability to make direct one-to-one comparisons with the extensive body of work by Schmahmann and others on structure-function relations in cerebellar disease. Future studies that incorporate a broader range of neuropsychological assessment tools will contribute to a more complete picture of how the cerebellum influences function in this genetic syndrome.

References for supplemental methods and extended discussion

Ali ZS, Lang SS, Bakar D, Storm PB, Stein SC (2014): Pediatric intracranial arachnoid cysts: comparative effectiveness of surgical treatment options. *Childs Nerv Syst*. 30:461-469.

Bosemani T, Orman G, Boltshauser E, Tekes A, Huisman TA, Poretti A (2015): Congenital abnormalities of the posterior fossa. *Radiographics*. 35:200-220.

Buckner RL, Head D, Parker J, Fotenos AF, Marcus D, Morris JC, et al. (2004): A unified approach for morphometric and functional data analysis in young, old, and demented adults using automated atlas-based head size normalization: reliability and validation against manual measurement of total intracranial volume. *Neuroimage*. 23:724-738.

Cornips EM, Overvliet GM, Weber JW, Postma AA, Hoeberigs CM, Baldewijns MM, et al. (2010): The clinical spectrum of Blake's pouch cyst: report of six illustrative cases. *Childs Nerv Syst*. 26:1057-1064.

Dewey J, Hana G, Russell T, Price J, McCaffrey D, Harezlak J, et al. (2010): Reliability and validity of MRI-based automated volumetry software relative to auto-assisted manual measurement of subcortical structures in HIV-infected patients from a multisite study. *Neuroimage*. 51:1334-1344.

Diedrichsen J (2006): A spatially unbiased atlas template of the human cerebellum. *Neuroimage*. 33(1):127-38.

Diedrichsen J, Balsters JH, Flavell J, Cussans E, Ramnani N (2009): A probabilistic MR atlas of the human cerebellum. *Neuroimage*. 46(1):39-46.

Doherty D, Millen KJ, Barkovich AJ (2013): Midbrain and hindbrain malformations: advances in clinical diagnosis, imaging, and genetics. *Lancet Neurol*. 12:381-393.

Eilers PH, Marx BD (1996): Flexible smoothing with B-splines and penalties. *Statistical science*. 11(2):89-121.

Elam JS, Glasser MF, Harms MP, Sotiropoulos SN, Andersson JLR, Burgess GC, et al. (2021): The Human Connectome Project: A retrospective. *Neuroimage*. 244:118543.

Fischl B, Salat DH, Busa E, Albert M, Dieterich M, Haselgrove C, et al. (2002): Whole brain segmentation: automated labeling of neuroanatomical structures in the human brain. *Neuron*. 33:341-355.

Graven P (1989): Smoothing noisy data with spline function: estimating the correct degree of smoothing by the method of generalized cross-validatlon. *Number. Math.* 31:377-403.

Han S, Carass A, He Y, & Prince JL (2020): Automatic cerebellum anatomical parcellation using U-Net with locally constrained optimization. *Neuroimage*, *218*: 116819.

Kerestes R, Han S, Balachander S, et al. (2022): A standardized pipeline for examining human cerebellar grey matter morphometry using structural magnetic resonance imaging. *J Vis Exp.* (180):10.3791/63340.

Kollias SS, Ball WS, Jr., Prenger EC (1993): Cystic malformations of the posterior fossa: differential diagnosis clarified through embryologic analysis. *Radiographics*. 13:1211-1231.

Koyabu D, Werneburg I, Morimoto N, Zollikofer CP, Forasiepi AM, Endo H, et al. (2014): Mammalian skull heterochrony reveals modular evolution and a link between cranial development and brain size. *Nature communications*. 5:1-9.

Lee DK, Yoon U, Kwak K, Lee JM (2015): Automated Segmentation of Cerebellum Using Brain Mask and Partial Volume Estimation Map. *Comput Math Methods Med*. 2015:167489.

Limperopoulos C, Robertson RL, Estroff JA, Barnewolt C, Levine D, Bassan H, et al. (2006): Diagnosis of inferior vermian hypoplasia by fetal magnetic resonance imaging: potential pitfalls and neurodevelopmental outcome. *Am J Obstet Gynecol*. 194:1070-1076.

Marcus DS, Harms MP, Snyder AZ, Jenkinson M, Wilson JA, Glasser MF, et al. (2013): Human Connectome Project informatics: quality control, database services, and data visualization. *Neuroimage*. 80:202-219.

Marin-Sanabria EA, Yamamoto H, Nagashima T, Kohmura E (2007): Evaluation of the management of arachnoid cyst of the posterior fossa in pediatric population: experience over 27 years. *Childs Nerv Syst*. 23:535-542.

Mayer KN, Latal B, Knirsch W, Scheer I, von Rhein M, Reich B, et al. (2016): Comparison of automated brain volumetry methods with stereology in children aged 2 to 3 years. *Neuroradiology*. 58:901-910.

McKinney AM (2017): Mega Cisterna Magna and Retrocerebellar Arachnoid Cysts. *Atlas of Normal Imaging Variations of the Brain, Skull, and Craniocervical Vasculature*: Springer, pp 19-41.

Nelson MD, Jr., Maher K, Gilles FH (2004): A different approach to cysts of the posterior fossa. *Pediatr Radiol*. 34:720-732.

Nychka D, Gray G, Haaland P, Martin D, O'connell M (1995): A nonparametric regression approach to syringe grading for quality improvement. *Journal of the American Statistical Association*. 90(432):1171–1178.

Nychka D, Furrer R, Paige J, Sain S (2021): "fields: Tools for spatial data". R package version 12.5, https://github.com/dnychka/fieldsRPackage

Ostby Y, Tamnes CK, Fjell AM, Westlye LT, Due-Tonnessen P, Walhovd KB (2009): Heterogeneity in subcortical brain development: A structural magnetic resonance imaging study of brain maturation from 8 to 30 years. *J Neurosci*. 29(38):11772-82.

Parisi MA, Dobyns WB (2003): Human malformations of the midbrain and hindbrain: review and proposed classification scheme. *Mol Genet Metab*. 80:36-53.

R Development Core Team (2021): R: A Language and Environment for Statistical Computing. Vienna, Austria: R Foundation for Statistical Computing.

Reinsch CH (1967): Smoothing by spline functions. *Numerische mathematik*. 10(3):177-183.

Romero JE, Coupe P, Lanuza E, Catheline G, Manjon JV (2021): Alzheimer's Disease Neuroimaging I. Toward a unified analysis of cerebellum maturation and aging across the entire lifespan: A MRI analysis. *Hum Brain Mapp.* 42(5):1287-303.

Sanfilipo MP, Benedict RH, Zivadinov R, Bakshi R (2004): Correction for intracranial volume in analysis of whole brain atrophy in multiple sclerosis: the proportion vs. residual method. *Neuroimage*. 22:1732-1743.

Sörös P, Wölk L, Bantel C, Bräuer A, Klawonn F, & Witt K (2021): Replicability, Repeatability, and Long-term Reproducibility of Cerebellar Morphometry. *Cerebellum. 20*(3), 439–453.

Sussman D, Leung RC, Chakravarty MM, Lerch JP, Taylor MJ (2016): The developing human brain: age-related changes in cortical, subcortical, and cerebellar anatomy. *Brain Behav*. 6(4):e00457.

Tarui T, Limperopoulos C, Sullivan NR, Robertson RL, du Plessis AJ (2014): Long-term developmental outcome of children with a fetal diagnosis of isolated inferior vermian hypoplasia. *Arch Dis Child Fetal Neonatal Ed*. 99:F54-58.

Tiemeier H, Lenroot RK, Greenstein DK, Tran L, Pierson R, Giedd JN (2010): Cerebellum development during childhood and adolescence: a longitudinal morphometric MRI study. *Neuroimage*. 49(1):63-70.

Tortori-Donati P, Fondelli MP, Rossi A, Carini S (1996): Cystic malformations of the posterior cranial fossa originating from a defect of the posterior membranous area. Mega cisterna magna and persisting Blake's pouch: two separate entities. *Childs Nerv Syst*. 12:303-308.

Wahba G (1980): Spline bases, regularization, and generalized cross validation for solving approximation problems with large quantities of noisy data. University of WISCONSIN.

Wierenga L, Langen M, Ambrosino S, van Dijk S, Oranje B, Durston S (2014): Typical development of basal ganglia, hippocampus, amygdala and cerebellum from age 7 to 24. *Neuroimage*. 96:67-72

Wierenga LM, Bos MGN, Schreuders E, Vd Kamp F, Peper JS, Tamnes CK, et al. (2018): Unraveling age, puberty and testosterone effects on subcortical brain development across adolescence. *Psychoneuroendocrinology*. 91:105-14.

Wood SN (2011): Fast stable restricted maximum likelihood and marginal likelihood estimation of semiparametric generalized linear models. *Journal of the Royal Statistical Society: Series B (Statistical Methodology).* 73(1):3-36.

Wood SN (2013): On p-values for smooth components of an extended generalized additive model. *Biometrika*. 100(1), 221-228.

Wood SN (2017): Generalized additive models: an introduction with R. CRC press.

Wu KH, Chen CY, Shen EY (2011): The cerebellar development in chinese children-a study by voxel-based volume measurement of reconstructed 3D MRI scan. *Pediatr Res.* 69(1):80-3
